# Sequential C–O decarboxylative vinylation/C–H arylation of cyclic oxalates *via* a nickel-catalyzed multicomponent radical cascade†

**DOI:** 10.1039/d0sc01471k

**Published:** 2020-04-14

**Authors:** Huan Li, Lei Guo, Xiaoliang Feng, Liping Huo, Shengqing Zhu, Lingling Chu

**Affiliations:** State Key Laboratory for Modification of Chemical Fibers and Polymer Materials, College of Chemistry, Chemical Engineering and Biotechnology, Center for Advanced Low-Dimension Materials, Donghua University Shanghai 201620 China Lingling.chu1@dhu.edu.cn

## Abstract

A selective, sequential C–O decarboxylative vinylation/C–H arylation of cyclic alcohol derivatives enabled by visible-light photoredox/nickel dual catalysis is described. This protocol utilizes a multicomponent radical cascade process, *i.e.* decarboxylative vinylation/1,5-HAT/aryl cross-coupling, to achieve efficient, site-selective dual-functionalization of saturated cyclic hydrocarbons in one single operation. This synergistic protocol provides straightforward access to sp^3^-enriched scaffolds and an alternative retrosynthetic disconnection to diversely functionalized saturated ring systems from the simple starting materials.

## Abstract 2

A selective, sequential C–O decarboxylative vinylation/C–H arylation of cyclic alcohol derivatives enabled by visible-light photoredox/nickel dual catalysis has been described.

## Graphical abstract

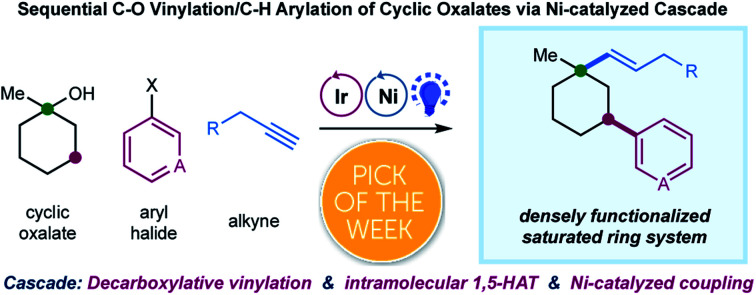

## Introduction

**Fig. 1:**
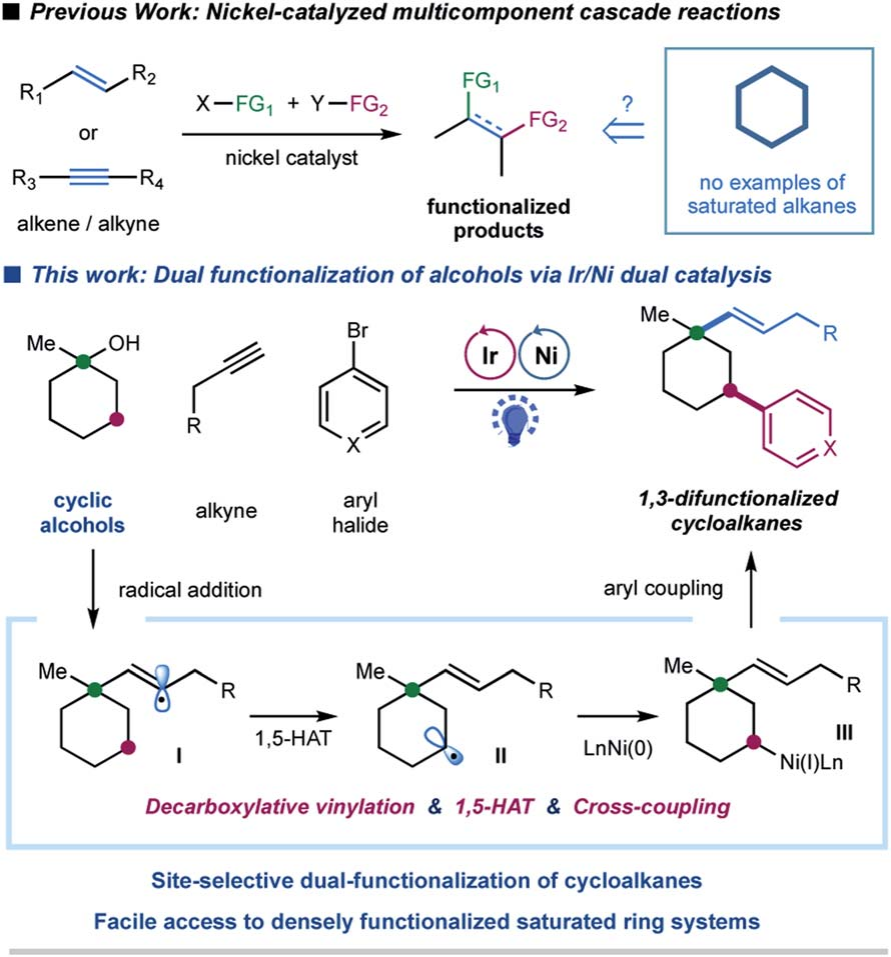
Multicomponent cascade reaction of cycloalkanes enabled by photoredox/nickel dual catalysis.

In the last decade, the nickel-catalyzed cross-coupling reaction has emerged as a powerful technique to construct C–C bonds in chemical synthesis.^1^ Notably, nickel-catalyzed multicomponent reactions, that allow for the formation of multiple C–C and/or C–heteroatom bonds thus enabling the streamlined synthesis of complex molecular scaffolds in a single operation, are particularly attractive due to their good compatibility and unique selectivity.^2^ Significant progress has been achieved in the area of catalytic multicomponent difunctionalization of unsaturated systems, enabling the simultaneous, one-pot installation of two functionalities over double bonds or triple bonds *via* nickel catalysis (Fig. 1).^3,4^ In contrast, there has been a lack of reports in which saturated hydrocarbons have been manipulated for cross-coupling at more than one reaction site in one single operation.^5^ This catalytic strategy would simultaneously install two sp^3^ C–C bonds in saturated hydrocarbons, readily available and abundant building blocks in organic synthesis; nevertheless, the realization of such a strategy requires the ability to overcome the relative inertness of saturated hydrocarbons and to control the selectivity in the presence of multiple similar chemical bonds.

Alcohols have been widely employed as salient synthetic building blocks in chemical synthesis. Notable advances have been made in selective functionalization of sp^3^ C–H bonds of alcohols through the hydrogen-atom-transfer (HAT) process.^6,7^ Moreover, alcohols can function as latent alkylating agents through transition-metal-catalyzed *ipso*-C–O activation^8^ or homolytic C–O cleavage.^4*j*,*k*,9,10^ These catalytic approaches enable the facile construction of diverse structural motifs from abundant alcohols. Nevertheless, one single transformation that combines *ipso*-C–O functionalization and remote C–H functionalization of alcohols, which would provide an attractive platform for the synthesis of highly functionalized sp^3^-rich scaffolds,^11^ has not been reported yet. We herein report a selective, cascade C–O bond vinylation/C–H bond arylation of alcohols that achieves site-selective dual carbofunctionalization of simple abundant aliphatic alcohols *via* a photoredox/nickel^12^-enabled multicomponent radical cascade process. This strategy relies on a synergistic combination of alkyl oxalate decarboxylation, 1,5-hydrogen atom transfer (1,5-HAT) of the vinyl radical, and C(sp^3^)–C(sp^2^) cross-coupling. Our original design for this radical cascade strategy is outlined in Fig. 1. Specifically, we envisioned that a radical addition of alkylalkyne would give rise to a σ-type vinyl radical **I**, which would prefer to undergo an intramolecular 1,5-HAT process to yield a nucleophilic alkyl radical species **II**.^13^ Subsequent nickel-mediated coupling of alkyl radial **II** with aryl halides would forge a C(sp^3^)–Ar bond,^12^ and finally lead to a sequential two-site functionalization of oxalates, *i.e.* C–O decarboxylative vinylation & C–H arylation (Fig. 1). We are particularly interested in exploring cyclic alkyl oxalates to construct highly functionalized cycloalkanes,^14^ due to two considerations: (i) this synergistic cascade process would undoubtedly expedite the synthesis of challenging aliphatic rings, and more importantly, would provide a novel retrosynthetic disconnection for complex saturated ring systems from simple starting materials; (ii) conformation of cyclic substrates might be beneficial for the intramolecular 1,5-HAT migration of vinyl radical species.^15^

## Results and discussion

We began our investigations by employing *tertiary* cyclic oxalate **1** as a model substrate to test the possibilities (Table 1). Pleasingly, we found that irradiation of a DMSO solution of **1**, aliphatic alkyne **2**, and 4-bromobenzonitrile **3** did afford the desired 1,3-vinylarylation product **4** in 84% yield in the presence of catalytic Ir[dF(CF_3_)ppy]_2_(dtbbpy)PF_6_, NiCl_2_(Py)_4_, 4,4′-di-*tert*-butyl-2,2′-dipyridyl (dtbbpy), and bis(4-methoxyphenyl)methanone (Table 1, entry 1). The countercation of oxalates impacted the reaction efficiency to a small extent, while the use of cesium oxalates afforded higher yields than the corresponding Li, Na, K salts (entries 2–4). Further evaluation indicated that both Ni(ii) and Ni(0) catalysts were able to promote the desired transformation, with the precatalyst NiCl_2_(Py)_4_ proving optimal (entries 5–9). Control experiments demonstrated that the photocatalyst, the nickel catalyst, and visible light were all essential to this synergistic cascade process, as the desired 1,3-vinylarylation products were not observed in the absence of any of these components (entries 10–12). Nevertheless, low conversion (27% yield) was still observed in the absence of the bipyridine ligand (entry 13). The use of bis(4-methoxyphenyl)methanone as an additive proved to be slightly beneficial to the reaction efficiency (entry 14).^13^

**Table 1:** Reaction optimization^a^

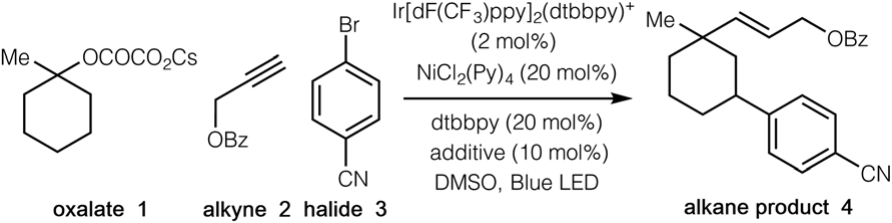		
Entry	Variations from standard conditions	Yield^b^
1	None	84%
2	ROCOCO_2_Li	55%
3	ROCOCO_2_Na	71%
4	ROCOCO_2_K	62%
5	NiCl_2_·DME	70%
6	NiCl_2_·(PPh_3_)_2_	63%
7	NiBr_2_·dtbbpy	52%
8	NiI_2_	40%
9	Ni(COD)_2_	34%
10	w/o photocatalyst	0
11	w/o nickel catalyst	0
12	w/o visible light	0
13	w/o ligand	27%
14	w/o bis(4-methoxyphenyl)methanone	78%

a Reaction conditions: **Ir-1** (2 mol%), NiCl_2_(Py)_4_ (20 mol%), dtbbpy (20 mol%), alkene **2** (0.1 mmol), bromide **3** (2.0 equiv.), oxalate **1** (3.0 equiv.), bis(4-methoxyphenyl)methanone (additive) (10 mol%), DMSO [0.05 M], 37 °C, 90W blue LED.

b Yields were determined by ^1^H NMR analysis of the crude reaction mixtures.

**Scheme 1:**
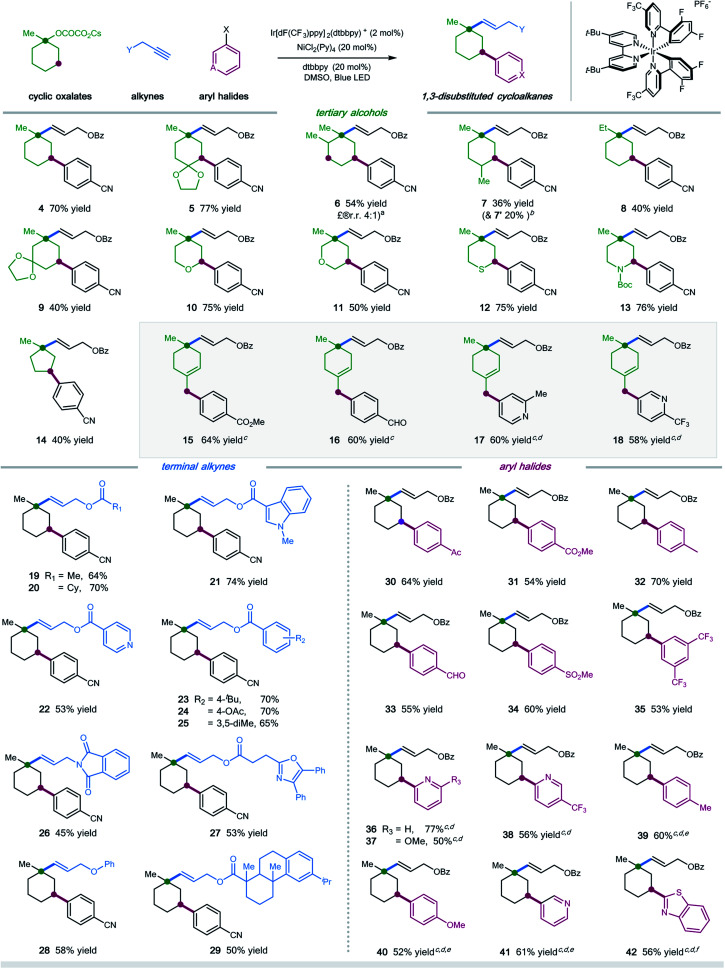
Substrate scope. Ir[dF(CF_3_)ppy]_2_(dtbbpy)PF_6_ (2 mol%), NiCl_2_(Py)_4_ (20 mol%), dtbbpy (20 mol%), bis(4-methoxyphenyl)methanone (10 mol%), alkyne (0.1 mmol), oxalate (3.0 equiv.), aryl halide (2.0 equiv.), DMSO [0.05 M], 37 °C, 90W blue LED. Isolated yields, ratios of diastereoisomers determined by ^1^H NMR analysis are between 1 : 1 and 1 : 1.2. See the ESI† for experimental details. ^*a*^Ratio of regioisomers was determined *via* HPLC; ^*b*^molecular structure of **7′** is shown in the ESI;†^*c*^w/o bis(4-methoxyphenyl)methanone as additive; ^*d*^DMSO/EA (4 : 1); ^*e*^employing aryl iodides; ^*f*^employing aryl chlorides.

With the optimal conditions in hand, we next turned our attention to evaluating the applicability of substrates as well as the potential limitations of this dual functionalization protocol. As shown in Scheme 1, a number of tertiary cyclic oxalates, readily prepared from the corresponding alcohols, could undergo the sequential C–O vinylation/C–H arylation with excellent site-selectivity, installing both vinyl and aryl functionalities onto the skeletons of alcohols under redox-neutral and mild conditions (products **4–14**, 36–77% yields). A number of substituents including alkyl, ketal, ketone, and alkene on the cyclohexyl alcohols were tolerated in this dual catalytic cascade system, furnishing multi-substituted cyclic alkanes in a single operation under mild conditions (products **5–9**, 36–77% yields). Nonetheless, steric hindrance of the substituents was found to have a considerable effect on the reaction efficiency: installing a methyl group at the α- or γ-position, or replacing methyl with ethyl at the *ipso* position of cyclohexyl oxalates resulted in decreased efficiency (products **6–8**, 36–54% yields). Pleasingly, saturated O-, S-, and N-heterocyclic oxalates turned out to be viable substrates, yielding the vinyl/aryl-disubstituted saturated heterocycles with good efficiency (products **10–13**, 50–76% yields). Moreover, cyclopentyl oxalates were also competent substrates, delivering the 1,3-difunctionalized cyclopentane products in synthetically useful yields (product **14**, 40% yield).

Unfortunately, cyclic oxalates with larger or smaller ring sizes (*e.g.* 4- and 7-membered cyclic oxalates), bicyclic oxalates, and linear oxalates were unsuccessful substrates for this cascade protocol, probably due to the less favorable conformation (for unsuccessful oxalates, see Section 5, page S62 in the ESI†). Interestingly, the reaction of 4-methylenecyclohexyl oxalate with aryl bromides under optimal conditions afforded the 1,5-vinylarylation products in moderate yields (products **15–18**, 58–64% yields). Excellent chemoselectivity was observed in this case, with no observations of 1,3-difunctionalized products. We reasoned that allyl-Ni species, generated *via* intramolecular 1,5-HAT followed by nickel trapping, underwent a selective coupling with aryl bromide at the terminal position, probably due to steric hindrance, to afford the cyclohexene product. This protocol represents a new and efficient platform to construct highly functionalized saturated heterocycles, important structural scaffolds for bioactive molecules, from simple starting materials.

Next, we examined the scope with respect to the alkyne component (Scheme 1). Pleasingly, a wide range of terminal alkylalkynes could be efficiently employed in this cascade protocol, yielding the corresponding *trans*-alkenes in moderate to good yields (products **19–29**, 45–74% yields). Notably, aliphatic alkynes tethered with complex molecules, exemplified by oxaprozin and dehydroabietic acid, also worked with moderate efficiency, further demonstrating the amenability of this synergistic strategy for late-stage manipulations (products **27** and **29**, 53% and 50% yield, respectively). Nevertheless, internal alkynes proved to be inefficient substrates, most of which remained intact under the standard conditions.

Finally, we explored the scope of aryl halides in this multicomponent transformation (Scheme 1). Aryl bromides containing electron-withdrawing substituents, including aldehydes, ketones, esters, nitriles, sulfones, and trifluoromethylates, are competent coupling partners under optimal conditions, delivering the desired products with good efficiency (products **30–35**, 53–70% yields). The mild conditions allow for the good tolerance of these important functionalities. This reaction is amenable to heteroaryl halides, selectively installing pyridines and benzothiazoles into cyclohexanes with moderate yields (products **36–38** and **41–42**, 50–77% yields). Additionally, (hetero)aryl iodides/chlorides also participated in this sequential C–O/C–H dual functionalization process smoothly (products **39–42**, 52–61% yields). Electron-rich aryl halides were applicable coupling partners, albeit with decreased efficiency (products **39–40**).

**Scheme 2:**
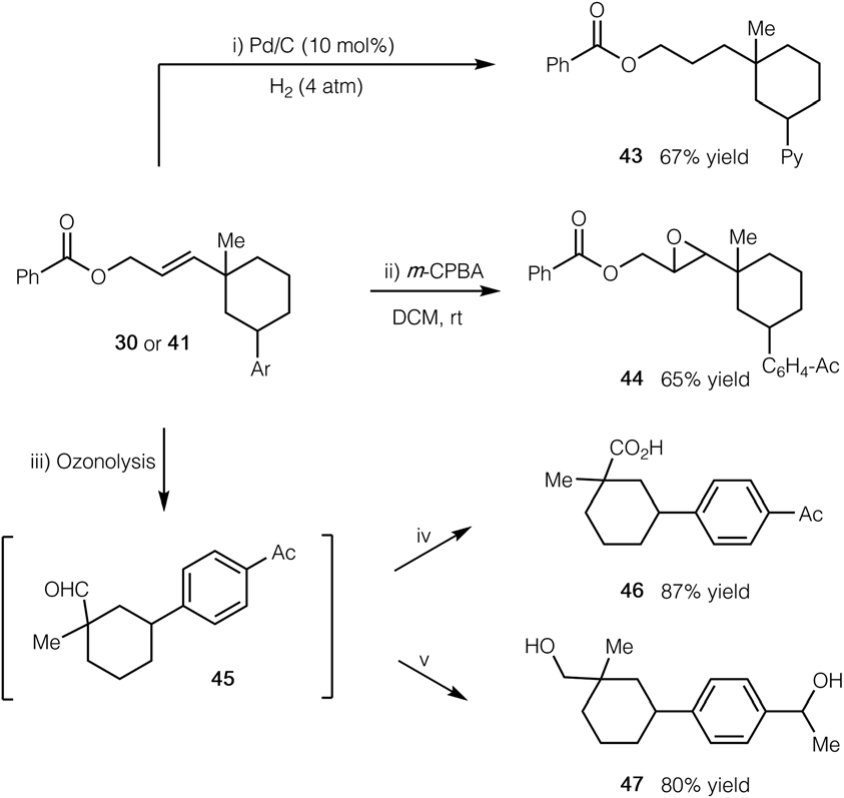
Synthetic manipulations of products. (i) Pd/C (10 mol%), H_2_ (4 atm), THF, rt; (ii) *m*-CPBA (2 equiv.), CH_2_Cl_2_, rt; (iii) O_3_, CH_2_Cl_2_, −78 °C; Me_2_S (10 equiv.), rt; (iv) NaClO_2_ (5 equiv.), H_2_O_2_ (4 equiv.), MeCN, 0 °C to rt; (v) NaBH_4_ (10 equiv.), MeOH, 0 °C to rt. See the ESI† for experimental details.

To further demonstrate the synthetic application of our cascade protocol, we have performed several transformations by utilizing the alkene functionality (Scheme 2). The double bond of compound **41** readily underwent selective hydrogenation with H_2_ in the presence of a Pd/C catalyst (product **43**). Epoxidation of compound **30** with *m*-CPBA gave epoxide **44** in 65% yield. Furthermore, ozonolysis of **30** led to the formation of aldehyde **45**, which could subsequently be oxidized to the corresponding carboxylic acid **46**, or be reduced to the related alcohol **47** in good yield.

**Scheme 3:**
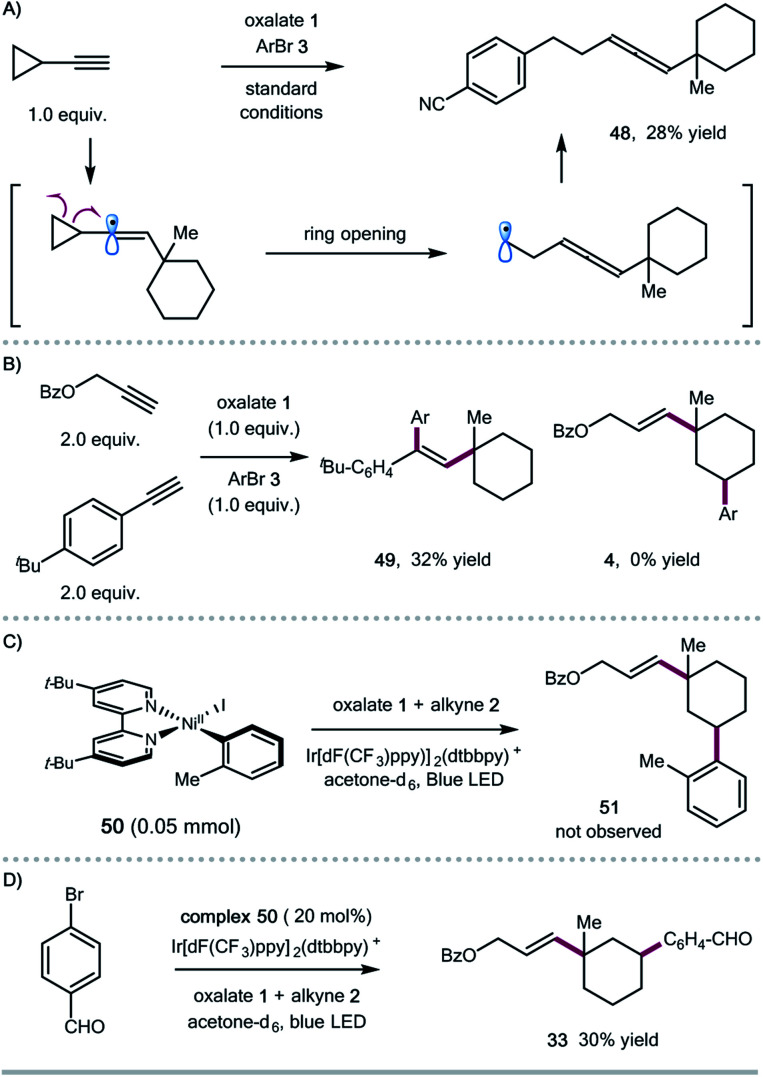
Mechanistic studies: (A) competitive experiments between arylalkyne and alkylalkyne; (B) radical clock experiment; (C) stoichiometric reaction of the Ar–Ni(ii) complex; (D) reaction in the presence of a catalytic amount of the Ar–Ni(ii) complex.

To shed some light on the potential reaction pathway, we have conducted several mechanistic experiments (Scheme 3). Reaction of ethynylcyclopropane with oxalate **1** and aryl bromide **3** gave the allene product **48**, presumably generated *via* a ring-opening/cross-coupling process, indicating the involvement of vinyl radical species (Scheme 3A). Initially, we assumed that the 1,5-HAT process could be related to the subtle conformation of oxalates. We also found that the expected 1,5-HAT process is reliant on the nature of the vinyl radicals (σ-type *vs.* π-type). For instance, competitive experiments between aryl and aliphatic alkynes showed that arylalkynes exhibited higher reactivity to afford the exclusive formation of 1,2-alkylarylation products,^4*j*^ and the 1,5-HAT/coupling product was not observed in this case (Scheme 3B). Regarding the coupling step, we prepared the ligated Ar–Ni(ii) complex **50**,^16^ and found that the stoichiometric reaction of the Ar–Ni(ii) complex with alkyne and oxalate didn't form the desired 1,3-disubstituted cycloalkane product **51**, suggesting that Ar–Ni(ii) might not be a reactive intermediate for this cascade transformation (Scheme 3C). Nevertheless, the Ar–Ni(ii) complex was able to catalyze the synergistic cascade reaction, giving the 1,3-disubstituted cyclohexane **33** in 30% yield in acetone (Scheme 3D).

**Scheme 4:**
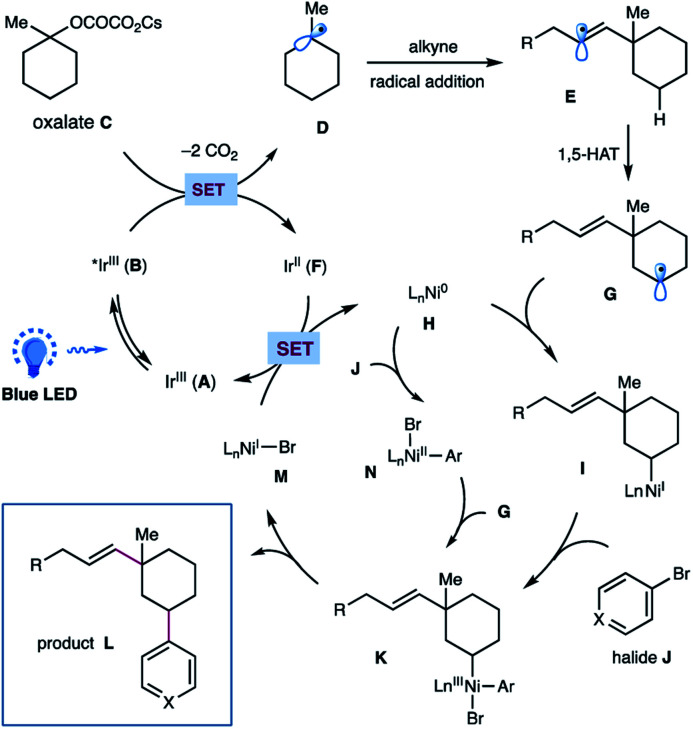
Proposed mechanism for this metallaphotoredox-catalyzed dual functionalization of cyclic oxalates.

On the basis of these experimental results as well as literature precedents,^12,17^ a plausible mechanism for this photoredox/nickel-catalyzed dual functionalization is depicted in Scheme 4. A thermodynamically feasible SET event between the photoexcited Ir-catalyst **B** and oxalate **C** would generate a tertiary alkyl radical **D***via* decarboxylation, followed by a radical addition of alkyne to give rise to a σ-type vinyl radical **E**. The resulting vinyl radical **E** would go through an intramolecular 1,5-HAT to selectively activate the sp^3^ C–H of oxalates and to afford a nucleophilic, secondary alkyl radical species **G**. Subsequent interception of alkyl radical **G** by Ni(0) **H** would generate an alkyl-Ni(i) species **I**, which then undergoes an oxidative addition with aryl halide **J** to yield an (alkyl)(aryl)Ni(iii) intermediate **K**.^17^ This high-valent Ni(iii) complex **K** would undergo a feasible reductive elimination to forge the C(sp^3^)–Ar bond and furnish the final product **L** as well as Ni(i) species **M**. Finally, a SET event between Ni(i) **M** and the reduced Ir(ii) **F** would regenerate Ni(0) **H** and the ground state Ir(iii) **A** to close these two catalytic cycles. At this stage, we could not preclude another pathway that proceeds *via* oxidative addition of aryl bromide to Ni(0) followed by interception of Ar–Ni(ii) **N** by alkyl radical species to afford the same (alkyl)(aryl)Ni(iii) **K**.

## Conclusions

In summary, we have developed a sequential C–O decarboxylative vinylation/C–H arylation of cyclic oxalates *via* photoredox/nickel dual catalysis. This synergistic protocol enables efficient and selective assembly of both vinyl and aryl functionalities onto saturated cyclic hydrocarbons in one single operation under mild and redox-neutral conditions, providing a new and complementary retrosynthetic method for densely functionalized saturated cyclic hydrocarbons. The mild conditions allow for excellent compatibility of functional groups and substrate scope in the oxalates, alkynes, and (hetero)aryl halides.

## Conflicts of interest

There are no conflicts to declare.

## Supplementary Material

SC-011-D0SC01471K-s001
