# Supplementary material for: Sequential C–O decarboxylative vinylation/C–H arylation of cyclic oxalates via a nickel-catalyzed multicomponent radical cascade
Source: Chem Sci. 2020 Apr 14;11(19):4904–10. doi: 10.1039/d0sc01471k (PMC8159219; doi:10.1039/d0sc01471k)

Electronic Supplementary Material (ESI) for Chemical Science.
This journal is © The Royal Society of Chemistry 2020

Supporting Information for

# Sequential C-O Decarboxylative Vinylation/C-H Arylation of Cyclic Oxalates via Nickel-Catalyzed Multicomponent Radical Cascade

Huan Li, Lei Guo, Xiaoliang Feng, Liping Huo, Shengqing Zhu, and Lingling Chu*

# Table of Contents


1. **General Information……………………………………………......3**

2. **Substrate Preparations and Characterizations…………………...4**

3. **Experimental procedure and Characterization of products…....16**

4. **Transformations of Products…………………………………...…57**

5. **Unsuccessful Oxalates and Mechanistic Studies…………………62**

6. **Spectral Data……………………………………………………….69**

# 1. General Information

Commercial reagents were purchased from Adamas, Aldrich, TCI, Energy Chemical and J&K chemical, and were used as received. All reactions were carried out in oven-dried glassware under an atmosphere of nitrogen unless otherwise noted. Chromatographic purification of products was accomplished by flash chromatography using silica gel. Thin-layer chromatography (TLC) was performed on Silicycle 250 mm silica gel F-254 plates. $^{1}H$, $^{19}F$ and $^{13}C$ NMR spectra were recorded on Bruker 400 (400 and 100 MHz) and Bruker 600 (600 and 150 MHz), and are internally referenced to residual solvent signals (for $CDCl_3$, δ 7.26 ppm and 77.0 ppm; DMSO-$d_6$, 2.50 ppm and 39.52 ppm, $CD_2Cl_2$, 5.35 ppm). Data for $^{1}H$ NMR are reported as follows: chemical shift (δ ppm), multiplicity (s = singlet, d = doublet, t = triplet, m = multiplet, br = broad), integration, coupling constant (Hz). $^{13}C$ spectra were reported as chemical shifts in ppm and multiplicity where appropriate. High resolution mass spectra were obtained at Shanghai Institute of Organic Chemistry mass spectrometry facilities.

## 2. Substrate Preparations and Characterizations

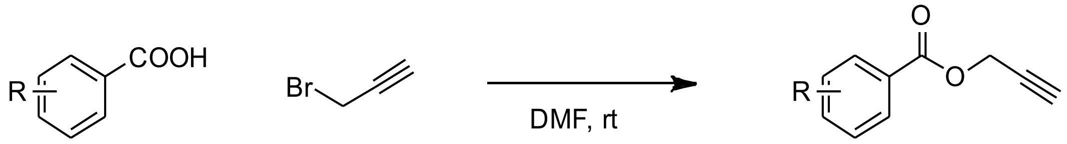


**General procedure for the synthesis of Propargyl benzoate derivative[1]:**

To a solution of commercially available Benzoic acid derivative (5.00 mmol, 1.0 equiv.) in dry DMF (12.5 mL, 0.4 M) was added $K_2CO_3$ (1.38 g, 10.0 mmol, 2.0 equiv.) and propargyl bromide (568 μL, 7.50 mmol, 1.5 eq.) at 0 °C. After stirring at rt for 3 h, the reaction was quenched with sat. $NH_4Cl$ and the mixture was extracted with EtOAc. The organic layer was washed with $H_2O$, brine, dried over anhydrous $Na_2SO_4$ and concentrated in reduced pressure. The crude product was purified by silica gel chromatography (hexane/EtOAc = 20:1) to afford propargyl benzoate.

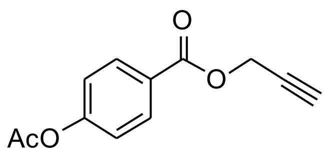


**Prop-2-yn-1-yl 4-acetoxybenzoate (S1):**

Colorless oil (600 mg, 55% yield). $^1$H NMR (600 MHz, $CDCl_3$) δ 8.09 (d, *J* = 8.6 Hz, 2H), 7.18 (d, *J* = 8.6 Hz, 2H), 4.93 – 4.89 (m, 2H), 2.52 (t, *J* = 2.4 Hz, 1H), 2.31 (s, 3H).$^{13}$C NMR (150 MHz, $CDCl_3$) δ 168.77, 164.97, 154.62, 131.42, 126.93, 121.72, 77.63, 75.13, 52.55, 21.15.

**HRMS (EI):** m/z calcd. for $C_{12}H_{10}O_4^+$ ($[M]^+$) 218.0579, found 218.0571.

---

[1] Takagi, K., Fukuda, H., Shuto, S., Otaka, A., & Arisawa, M. Safe Removal of the Allyl Protecting Groups of Allyl Esters using a Recyclable, Low-Leaching and Ligand-Free Palladium Nanoparticle Catalyst. *Adv. Synth. Catal.,* **2015**, *357*(9): 2119.

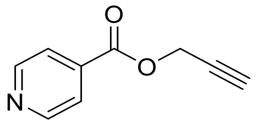


**Prop-2-yn-1-yl isonicotinate (S2):**

Yellow solid (400 mg, 50% yield).$^{1}$H NMR (600 MHz, $CDCl_3$) δ 8.78 (d, *J* = 6.0 Hz, 2H), 7.85 (d, *J* = 6.0 Hz, 2H), 4.93 (d, *J* = 2.4 Hz, 2H), 2.55 (t, *J* = 2.4 Hz, 1H). $^{13}$C NMR (150 MHz, $CDCl_3$) δ 164.38, 150.70, 136.54, 122.90, 76.95, 75.69, 53.16.

**HRMS (EI):** m/z calcd. for $C_9H_7NO_2^+$ $[M]^+$: 161.0477; Found: 161.0477.

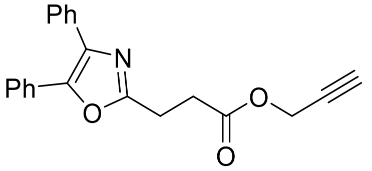


**prop-2-yn-1-yl 3-(4,5-diphenyloxazol-2-yl) propanoate (S3):**

White solid (861 mg, 52% yield). $^{1}$H NMR (600 MHz, $CDCl_3$) δ 7.68 – 7.64 (m, 2H), 7.61 – 7.58 (m, 2H), 7.41 – 7.32 (m, 6H), 4.77 (d, *J* = 2.4 Hz, 2H), 3.23 (t, *J* = 7.5 Hz, 2H), 3.00 (t, *J* = 7.5 Hz, 2H), 2.49 (t, *J* = 2.4 Hz, 1H). $^{13}$C NMR (150 MHz, $CDCl_3$) δ 171.28, 161.43, 145.49, 135.14, 132.41, 128.95, 128.65, 128.56, 128.48, 128.08, 127.90, 126.50, 77.49, 75.05, 52.31, 30.86, 23.38.

**HRMS (ESI):** m/z calcd. for $C_{21}H_{18}O_3N^+$ $[M+H]^+$: 332.1287; Found: 332.1280.

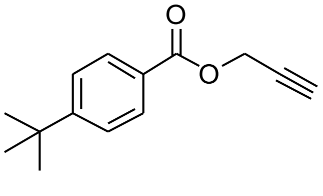


**Prop-2-yn-1-yl 4-(tert-butyl)benzoate (S4):**

Colorless liquid (654 mg, 60% yield).$^{1}$H NMR (600 MHz, $CDCl_3$) δ 8.00 (d, *J* = 8.4 Hz, 2H), 7.46 (d, *J* = 8.4 Hz, 2H), 4.91 (d, *J* = 2.4 Hz, 2H), 2.51 (t, *J* = 2.4 Hz, 1H), 1.33 (s, 9H). $^{13}$C NMR (150 MHz, $CDCl_3$) δ 165.75, 157.01, 129.72, 126.65, 125.42, 77.94, 74.92, 52.25, 35.11, 31.10.

**HRMS (ESI+):** m/z calcd. for $C_{14}H_{17}O_2{}^+$ $[M+H]^+$: 217.1229; Found: 217.1223.

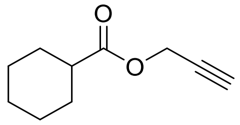

**Prop-2-yn-1-yl cyclohexanecarboxylate (S5):**

Colorless liquid (700mg, 84% yield). $^{1}$H NMR (600 MHz, $CDCl_3$) δ 4.62 (d, $J$ = 2.5 Hz, 2H), 2.43 (t, $J$ = 2.5 Hz, 1H), 2.31 (tt, $J$ = 11.3, 3.6 Hz, 1H), 1.92 – 1.84 (m, 2H), 1.71 (dt, $J$ = 22.9, 8.5 Hz, 2H), 1.65 – 1.56 (m, 1H), 1.48 – 1.38 (m, 2H), 1.31 – 1.16 (m, 3H). $^{13}$C NMR (150 MHz, $CDCl_3$) δ 175.08, 77.90, 74.61, 51.66, 42.85, 28.82, 25.65, 25.31.

**HRMS (EI):** m/z calcd. for $C_{10}H_{14}O_2^+$ ($[M]^+$) :166.0994; Found: 166.0994.

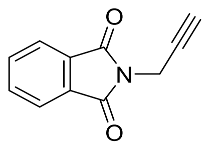

**2-(Prop-2-yn-1-yl)isoindoline-1,3-dione (S6).**

White solid (647.5 mg, 70% yield). $^{1}$H NMR (600 MHz, $CDCl_3$) δ 7.88 (dd, $J$ = 5.1, 3.1 Hz, 2H), 7.74 (dd, $J$ = 5.2, 3.0 Hz, 2H), 4.45 (d, $J$ = 2.0 Hz, 2H), 2.22 (s, 1H). $^{13}$C NMR (150 MHz, $CDCl_3$) δ 166.98, 134.23, 131.99, 123.59, 77.17, 71.49, 27.00.

**HRMS (ESI+):** m/z calcd. for $C_{11}H_8O_2N^+$ $[M+H]^+$:186.0555； Found: 186.0550.

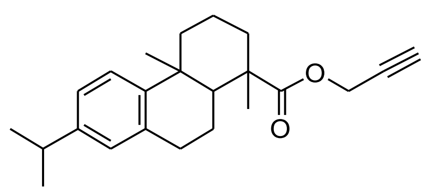

**Prop-2-yn-1-yl-7-isopropyl-1,4a-dimethyl-1,2,3,4,4a,9,10,10a-octahy drophenan-threne -1-carboxylate (S7).**

Colorless liquid (1547 mg, 91% yield). $^{1}$H NMR (600 MHz, $CDCl_3$) δ 7.17 (d, $J$ = 8.2 Hz, 1H), 7.01 (dd, $J$ = 8.1, 1.6 Hz, 1H), 6.89 (s, 1H), 4.71 (dt, $J$ = 15.6, 3.3 Hz, 1H), 4.64 – 4.59 (m, 1H), 2.96 – 2.80 (m, 3H), 2.43 (t, $J$ = 2.4 Hz, 1H), 2.35 – 2.25 (m, 2H), 1.88 – 1.79 (m, 2H), 1.79 – 1.69 (m, 2H), 1.69 – 1.65 (m, 1H), 1.54 – 1.48 (m, 1H), 1.47 – 1.43 (m, 1H), 1.29 (d, $J$ = 5.8 Hz, 3H), 1.23 (s, 3H), 1.22 (d, $J$ = 2.2 Hz, 6H). $^{13}$C

NMR (150 MHz, $CDCl_3$) δ 177.72, 146.78, 145.76, 134.71, 126.90, 124.18, 123.94, 77.96, 74.46, 52.03, 47.67, 44.82, 37.94, 36.97, 36.34, 33.47, 30.01, 25.16, 23.99, 21.66, 18.55, 16.49.

**HRMS (ESI+):** m/z calcd. for $C_{23}H_{31}O_2^+$ $[M+H]^+$: 339.2313; Found: 339.2319.

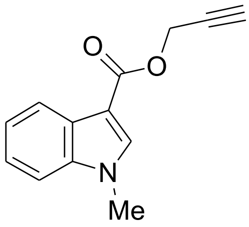


**Prop-2-yn-1-yl 1-methyl-1H-indole-3-carboxylate (S8).**

White solid (960 mg, 90% yield). $^1$H NMR (400 MHz, $CDCl_3$) δ 8.20 (dd, $J$ = 5.0, 4.0 Hz, 1H), 7.83 (s, 1H), 7.37-7.30 (m, 3H), 4.93 (d, $J$ = 2.0 Hz, 2H), 3.84 (s, 3H), 2.50 (t, $J$ = 2.0 Hz, 1H). $^{13}$C NMR (100 MHz, $CDCl_3$) δ 163.88, 137.23, 135.63, 126.65, 122.97, 122.12, 121.69, 109.81, 106.06, 78.56, 74.43, 51.21, 33.48.

**HRMS (EI):** m/z calcd. for $C_{13}H_{11}NO_2^+$ ([M]+): 213.0790; Found: 213.0790.

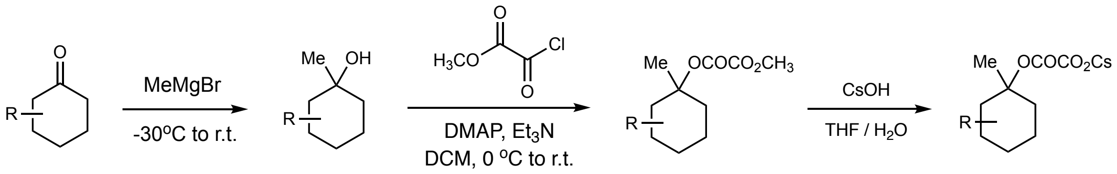


**General procedure for the synthesis of methyl alcohols[2]:** To a 3 M methyl magnesium bromide (1.3 equiv) in diethyl ether was added dropwise cyclobutanol (1.0 equiv) neat -30 °C under an atmosphere of nitrogen. The reaction mixture was stirred for an additional 2 h and then quenched with aqueous ammonium chloride. The mixture was extracted with ether and the combined organic layers were washed with a sodium bicarbonate solution and brine. Combined organic layer was dried with anhydrous sodium sulphate, evaporated to dryness under reduced pressure and was directly used for next reaction. A round-bottom flask was charged with tertiary alcohol (1.0 equiv.) and $CH_2Cl_2$ [0.1M]. Triethylamine (1.2 equiv.) and DMAP (0.1 equiv.)

[2]were added followed by drop-wise addition of methyl chlorooxoacetate (1.2 equiv.). The reaction was stirred for 4 h at room temperature, then quenched with sat. $NH_4Cl$ (aq). The aqueous phase was extracted with DCM, and the organic extracts were dried over $Na_2SO_4$ and concentrated. The crude material was purified by flash column chromatography on silica gel.

A round-bottom flask was charged with methyl oxalate (1.2 equiv.) followed by the addition of THF [1 M]. To this solution, 1 N aq. CsOH (1 equiv.) was added dropwise. The mixture was stirred vigorously for 1 h at room temperature, then extracted with hexane three times, the combined water layers were concentrated under reduced pressure to give the target product.

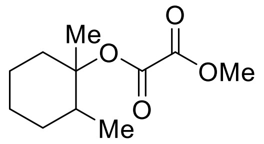


**(1S,2S)-1,2-dimethylcyclohexyl methyl oxalate (S9):**

Colorless solid (1033.7 mg, 64% yield).$^1$H NMR (600 MHz, $CDCl_3$) δ 3.86 (s, 3H), 2.72 – 2.63 (m, 1H), 1.72 – 1.63 (m, 1H), 1.56 (s, 3H), 1.53 – 1.42 (m, 4H), 1.38 – 1.22 (m, 3H), 0.99 (d, $J$ = 6.4 Hz, 3H). $^{13}$C NMR (150 MHz, $CDCl_3$) δ 159.09, 156.80, 87.95, 53.17, 41.81, 34.43, 30.08, 25.31, 23.83, 21.79, 15.18.

**HRMS (EI):** m/z calcd. for $C_{11}H_{18}O_4^+$ [M]$^+$: 214.1205; Found: 214.1205.

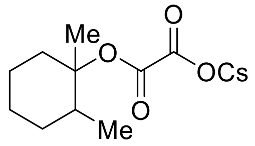


**Cesium 2-(((1S,2S)-1,2-dimethylcyclohexyl)oxy)-2-oxoacetate (S10):**

Colorless solid (224 mg, 67% yield).$^1$H NMR (600 MHz, DMSO) δ 2.57 – 2.52 (m, 1H), 1.57 (d, $J$ = 11.8 Hz, 1H), 1.41 (s, 3H), 1.41 – 1.32 (m, 5H), 1.29 – 1.13 (m, 2H),

[2] Nawrat, C. C., Jamison, C. R., Slutskyy, Y., MacMillan, D. W., & Overman, L. E. Oxalates as activating groups for alcohols in visible light photoredox catalysis: formation of quaternary centers by redox-neutral fragment coupling. *J. Am. Chem. Soc.*, **2015**, *137*, 11270.

0.88 (d, $J$ = 5.9 Hz, 3H).$^{13}$C NMR (150 MHz, DMSO) δ 168.14, 164.15, 81.10, 41.69, 34.67, 30.15, 25.45, 24.47, 21.76, 15.62.

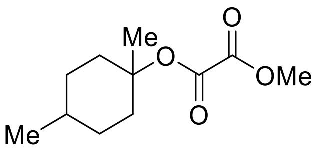


**1,4-dimethylcyclohexyl methyl oxalate (S11):**

Colorless solid (2.14 g, 77% yield, dr = 5:1). $^1$H NMR (600 MHz, $CDCl_3$) δ 3.87 (s, 0.5H), 3.85 (s, 2.5H), 2.40 – 2.07 (m, 2H), 1.80 (tt, $J$ = 28.1, 14.0 Hz, 2H), 1.73 – 1.63 (m, 2H), 1.59 (s, 2.5H), 1.54 (s, 0.5H), 1.53-1.48 (m, 1H), 1.17 – 1.05 (m, 2H), 0.93 – 0.89 (m, 3H). $^{13}$C NMR (150 MHz, $CDCl_3$) δ 159.09, 156.80, 87.95, 53.17, 41.81, 34.43, 30.08, 25.31, 23.83, 21.79, 15.18. *m/z* Calculated for $C_{13}H_{11}NO_2$ $[M+H]^+$: 215.1283; Found: 215.1280.

**HRMS (EI):** m/z calcd. for $C_{11}H_{18}O_4^+$ ($[M]^+$): 214.1205; Found: 214.1205.

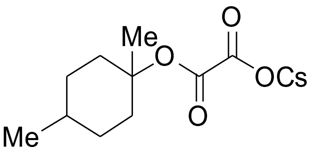


**Cesium 2-((1,4-dimethylcyclohexyl)oxy)-2-oxoacetate (S12):**

Preparation of **S12** using **S11** in a single configuration was colorless solid (266 mg, 80% yield). $^1$H NMR (400 MHz, DMSO) δ 2.27 (d, $J$ = 12.1 Hz, 2H), 1.55 – 1.17 (m, 10H), 0.96 (t, $J$ = 9.0 Hz, 3H). $^{13}$C NMR (100 MHz, DMSO) δ 167.55, 163.55, 78.51, 35.92, 31.16, 29.71, 26.00, 22.17.

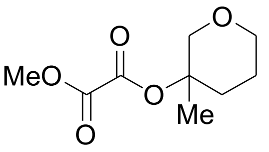


**Methyl (3-methyltetrahydro-2H-pyran-3-yl) oxalate (S13)**

Yellow oil (2.82 g, 70% yield).$^1$H NMR (600 MHz, $CDCl_3$) δ 4.13 (t, $J$ = 9.9 Hz, 1H), 3.87 (s, 3H), 3.83 – 3.78 (m, 1H), 3.53 – 3.47 (m, 1H), 3.39 (d, $J$ = 12.1 Hz, 1H), 2.34 – 2.26 (m, 1H), 1.90 – 1.84 (m, 1H), 1.77 – 1.69 (m, 1H), 1.57 – 1.52 (m, 1H), 1.51 (s,

3H). $^{13}C$ NMR (150 MHz, $CDCl_3$) δ 158.64, 156.65, 82.33, 72.82, 67.86, 53.41, 33.81, 22.18, 21.25.

**HRMS (ESI+):** m/z calcd. for $C_9H_{15}O_5{}^+$ $[M+Na]^+$: 225.0733, found225.0733.

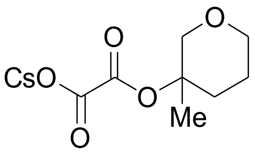


**Cesium 2-((3-methyltetrahydro-2H-pyran-3-yl)oxy)-2-oxoacetate (S14):** colorless solid (317 mg, 99% yield).$^1H$ NMR (600 MHz, DMSO) δ 3.93 – 3.84 (m, 1H), 3.71 (s, 2H), 3.45 – 3.40 (m, 1H), 2.20 (s, 1H), 1.83 – 1.72 (m, 2H), 1.59 – 1.50 (m, 1H), 1.45 (s, 3H).$^{13}C$ NMR (150 MHz, DMSO) δ 163.50, 73.50, 67.36, 33.72, 22.45, 21.77.

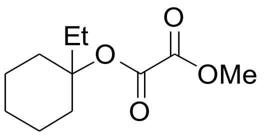


**1-Ethylcyclohexyl methyl oxalate (S15):** Colorless oil (1.77 g, 77% yield). $^1H$ NMR (400 MHz, Chloroform-d) δ 3.86 (d, J = 1.1 Hz, 3H), 2.24 (dd, J = 13.2, 4.3 Hz, 2H), 2.02 – 1.88 (m, 2H), 1.68 – 1.57 (m, 1H), 1.56 – 1.37 (m, 6H), 1.32 – 1.20 (m, 1H), 0.89 – 0.82 (m, 3H). $^{13}C$ NMR (100 MHz, CDCl3) δ 159.12, 156.62, 89.53, 53.17, 33.78, 29.87, 25.35, 21.70, 7.33.

**HRMS (EI):** m/z calcd. For $C_{11}H_{18}O_4{}^+$ $[M+NH^4]^+$: 232.1544; Found: 232.1543.

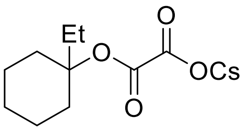


**Cesium 2-((1-ethylcyclohexyl)oxy)-2-oxoacetate (S16):**

White solid (268.1 mg, 81% yield). $^1H$ NMR (400 MHz, $D_2O$) δ 2.18 (s, 2H), 1.95 (q, J = 7.4, 6.4 Hz, 2H), 1.53 (t, J = 11.0 Hz, 7H), 1.33 (s, 1H), 0.95 – 0.82 (m, 3H).$^{13}C$ NMR (100 MHz, $D_2O$) δ 165.43, 164.43, 88.90, 33.64, 29.58, 24.97, 21.48, 6.73.

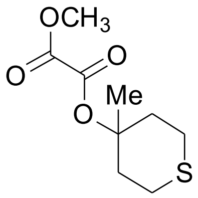


**Methyl (4-methyltetrahydro-2H-thiopyran-4-yl) oxalate (S17):**

Yellow oil (3.2 g, 72% yield).$^1$H NMR (600 MHz, $CDCl_3$) δ 3.85 (s, 3H), 2.84 (t, $J$ = 13.0 Hz, 2H), 2.56 (d, $J$ = 14.3 Hz, 2H), 2.42 (d, $J$ = 14.0 Hz, 2H), 1.79 – 1.70 (m, 2H), 1.54 (s, 3H). $^{13}$C NMR (150 MHz, $CDCl_3$) δ 158.58, 156.34, 84.32, 53.39, 37.06, 25.91, 23.81.

**HRMS (EI):** m/z calcd. For $C_9H_{14}O_4S^+[M]^+$: 218.0613; Found: 218.0613.

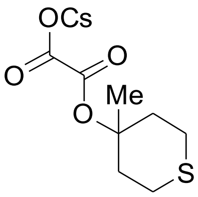


**Cesium 2-((4-methyltetrahydro-2H-thiopyran-4-yl)oxy)-2-oxoacetate (S18):**

White solid (258.7 mg, 77% yield).$^1$H NMR $^1$H NMR (600 MHz, DMSO) δ 2.77 (t, $J$ = 11.5 Hz, 2H), 2.38 (t, $J$ = 8.1 Hz, 4H), 1.61 (t, $J$ = 11.7 Hz, 2H), 1.41 (s, 3H). $^{13}$C NMR (150 MHz, DMSO) δ 167.59, 163.69, 78.16, 37.50, 26.49, 23.52.

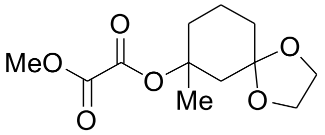


**Methyl (7-methyl-1,4-dioxaspiro[4.5]decan-7-yl) oxalate (S19):** To a 3 M methyl magnesium bromide (1.3 equiv) in diethyl ether was added dropwise 1,4-dioxaspiro[4.5]decan-7-one (2.09 g，5 mmol，1.0 equiv) neat -30 °C under an atmosphere of nitrogen. The reaction mixture was stirred for an additional 2 h and then quenched with aqueous ammonium chloride. The mixture was extracted with ether and the combined organic layers were washed with a sodium bicarbonate solution and brine.

The organic extract was dried over sodium sulfate and concentrated under vacuum to afford crude product 7-methyl-1,4-dioxaspiro[4.5]decan-7-ol for next step.

A round-bottom flask was charged with 7-methyl-1,4-dioxaspiro[4.5]decan-7-ol (1.0 equiv) and THF (0.25 M) under an atmosphere of argon. The solution was stirredand cooled to –78 ºC before a 2.5 M solution of n-BuLi in hexanes (2 mL, 1.0 equiv) was added drop-wise. The solution was stirred for 15 min, then methyl chlorooxoacetate (0.69 mL, 7.5 mmol, 1.5 equiv) was added drop-wise. The reaction was stirred for 2 h at -78ºC, then at cryogenic temperature as the dry ice bath slowly warmed to room temperature (4 h).Then quenched with sat. $NH_4Cl$ (aq). The aqueous phase was extracted with DCM, and the organic extracts were dried over $Na_2SO_4$ and concentrated to afford crude product. The crude material was purified by flash column chromatography on silica gel (PE: EA= 10:1) to give the target product as yellow oil (774 mg, 60% yield). $^{1}$H NMR (600 MHz, $CDCl_3$) δ 3.89 – 3.78 (m, 7H), 2.83 – 2.72 (m, 1H), 1.96 – 1.85 (m, 2H), 1.68 – 1.57 (m, 3H), 1.54 (dd, $J$ = 6.3, 0.9 Hz, 3H), 1.53 – 1.41 (m, 2H).$^{13}$C NMR (150 MHz, $CDCl_3$) δ 158.88, 156.93, 108.30, 85.70, 64.76, 64.01, 53.24, 40.37, 37.35, 34.55, 25.45, 19.20.

**HRMS (ESI+):** m/z calcd. for $C_{12}H_{22}O_6N^+$ [M+Na]$^+$: 281.0993, found: 281.0996.

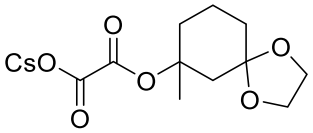


**Cesium 2-((7-methyl-1,4-dioxaspiro[4.5]decan-7-yl)oxy)-2-oxoacetate (S20):**. Yellow solid (318 mg, 99% yield).$^{1}$H NMR (600 MHz, DMSO) δ 3.90 – 3.70 (m, 4H), 2.05 – 1.90 (m, 2H), 1.72 (d, $J$ = 10.8 Hz, 1H), 1.67 – 1.57 (m, 2H), 1.57 – 1.50 (m, 1H), 1.43 (s, 3H), 1.42 – 1.31 (m, 2H).$^{13}$C NMR (150 MHz, DMSO) δ 167.86, 163.68, 108.58, 64.19, 63.91, 43.87, 36.79, 34.58, 24.43, 19.66.

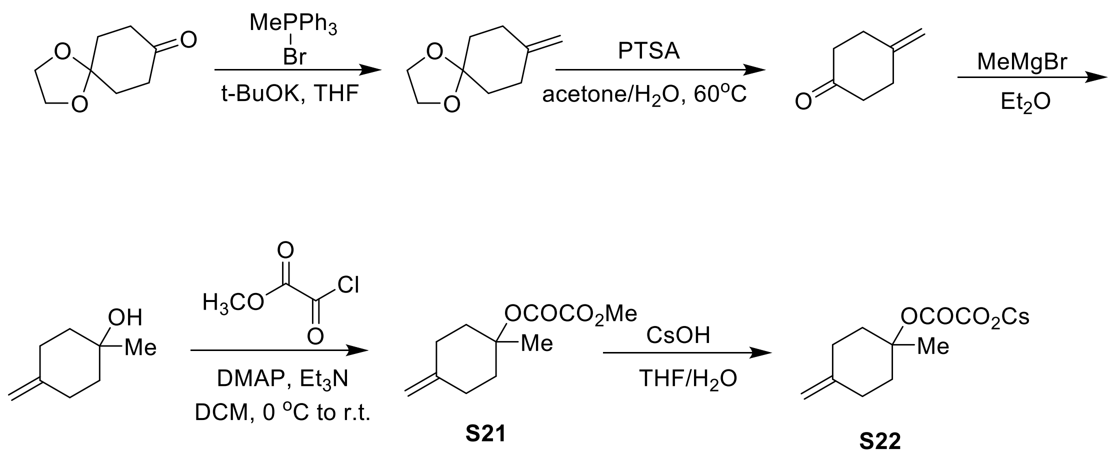


**Methyl (1-methyl-4-methylenecyclohexyl) oxalate (S21)** [2,3]

To a suspension of methyl triphenylphosphonium bromide (28.5 g, 80 mmol, 1.6 equiv.) in dry THF (160 mL) was added potassium tertiary butoxide (14.6 g, 130 mmol, 1.6 equiv.) at 0°C under nitrogen atmosphere and resulting yellow coloured suspension was stirred for one hour. A solution of 1,4-Dioxaspiro[4.5]decan-8-one(7.8 g, 50 mmol) in THF (20 mL) was added and the reaction mixture was stired for 12 h. After completion of the reaction (confirmed by TLC), saturated solution of ammonium chloride (50 mL) was added followed by workup with $CH_2Cl_2$ (3 × 80 mL). Combined organic layer was dried with anhydrous sodium sulphate, evaporated to dryness under reduced pressure and was directly used for next reaction.

The crude reaction mixture was re-dissolved in acetone/$H_2O$ (100 mL/8 mL) at room temperature followed by addition of PTSA (6.2 g, 65 mmol, 1.3 equiv.) and stired for 8 h at 60 °C under nitrogen atmosphere. After completion of reaction, saturated solution of sodium bicarbonate (50 mL) was added for quenching the reaction. Workup was done with $CH_2Cl_2$ (3 × 60 mL) and combined organic layer was dried with sodium sulphate and evaporated to dryness under reduced pressure. Column chromatography of the crude mixture was done with ethyl acetate / hexanes (10:90) and 4-methylenecyclohexan-1-one was obtained as a colorless liquid (2.2 g, 40%).The product was confirmed by GC-MS.

[3] Fairhurst, R. A., Marsilje, T. H., Stutz, S., Boos, A., Niklaus, M., Chen, B. & Stauffer, F. Optimisation of a 5-[3-phenyl-(2-cyclic-ether)-methyl-ether]-4-aminopyrrolopyrimidine series of IGF-1R inhibitors. *Bioorg. Med. Chem. Lett.*,**2016**, *26*, 2057.

To a 3 M methyl magnesium bromide (1.3 equiv) in diethyl ether was added dropwise 4-methylenecyclohexan-1-one (2.2 g，20 mmol，1.0 equiv) neat -30 °C under an atmosphere of nitrogen. The reaction mixture was stirred for an additional 2 h and then quenched with aqueous ammonium chloride. The mixture was extracted with ether and the combined organic layers were washed with a sodium bicarbonate solution and brine. The organic extract was dried over sodium sulfate and concentrated under vacuum to afford crude product 1-methyl-4-methylenecyclohexan-1-ol for next step.

A round-bottom flask was charged with 1-methyl-4-methylenecyclohexan-1-ol and $CH_2Cl_2$ [0.1M]. Triethylamine (3.3 mL, 24 mmol, 1.2 equiv.) and DMAP (488.5 mg, 2 mmol, 0.1 equiv.) were added followed by drop-wise addition of methyl chlorooxoacetate (2.2 mL, 24 mmol, 1.2 equiv.). The reaction was stirred for 4 h at room temperature, then quenched with sat. $NH_4Cl$ (aq). The aqueous phase was extracted with DCM, and the organic extracts were dried over $Na_2SO_4$ and concentrated. The crude material was purified by flash column chromatography on silica gel (PE) to give the target product

A round-bottom flask was charged with methyl oxalate (1.2 equiv.) followed by the addition of THF [1 M]. To this solution, 1 N aq. CsOH (1 equiv.) was added dropwise. The mixture was stirred vigorously for 1 h at room temperature, then extracted with hexane three times, the combined water layers were concentrated under reduced pressure to give the target product.

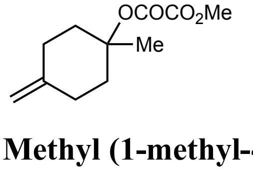


**Methyl (1-methyl-4-methylenecyclohexyl) oxalate (S21)**

Colorless solid (2.1 g, 50% yield).$^{1}$H NMR (600 MHz, $CDCl_3$) δ 4.67 (s, 2H), 3.87 (s, 3H), 2.39-2.36 (m, 2H), 2.28 (td, $J$ = 13.7, 4.2 Hz, 2H), 2.16 (dt, $J$ = 14.0, 4.2 Hz, 2H), 1.58 (s, 3H), 1.58 – 1.53 (m, 2H). $^{13}$C NMR (150 MHz, $CDCl_3$) δ 158.91, 156.72, 146.68, 108.12, 85.84, 53.28, 37.22, 30.14, 24.73.

**HRMS (ESI+):** m/z calcd. for $C_{11}H_{16}O_4N^+$ $[M+Na]^+$: 235.0940, found: 235.0941.

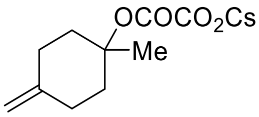


**Cesium 2-((1-methyl-4-methylenecyclohexyl)oxy)-2-oxoacetate(S22)**

Colorless solid (264 mg, 80% yield).$^{1}$H NMR (400 MHz, DMSO) δ 4.72 (s, 2H), 2.36 – 2.22 (m, 4H), 2.20 – 2.10 (m, 2H), 1.58 – 1.44 (m, 5H). $^{13}$C NMR (100 MHz, DMSO) δ 167.56, 163.35, 147.49, 107.41, 78.59, 37.08, 29.76, 24.89.

## 3. Experimental Procedure and Characterization of Products

**General procedure for the Sequential Vinylation/Arylation of *ipso*-C-O and *meta*-C-H Bonds of Cyclic Oxalates :** To a flame-dried 8 mL reaction vial was charged with $NiCl_2(Py)_4$ (0.02 mmol, 20 mol%), dtbbpy (0.02 mmol, 20 mol%), $Ir[dF(CF_3)ppy]_2(dtbbpy)PF_6$ (0.002 mmol, 2 mol%), aryl bromide (0.2 mmol, 2.0 equiv., if solid) and cesium salt (0.3 mmol, 3 equiv.), 4,4'-dimethoxybenzophenone (0.01 mmol, 10 mol%.), the vial was capped. DMSO [0.05 M] was added via a syringe, nitrogen bubbling for fifteen minutes, followed by the addition of alkyne (0.1 mmol, 1.0 equiv.). The reaction mixture was then irradiated with a 90 W blue LED lamp (at approximately 1.5 cm away from the light source) with cooling from a fan for 18 h. The reaction was quenched with $H_2O$, extracted with ethyl acetate. The combined organic layers were dried with $MgSO_4$, filtered, and concentrated in vacuo. The crude material was purified by flash chromatography to afford the product.

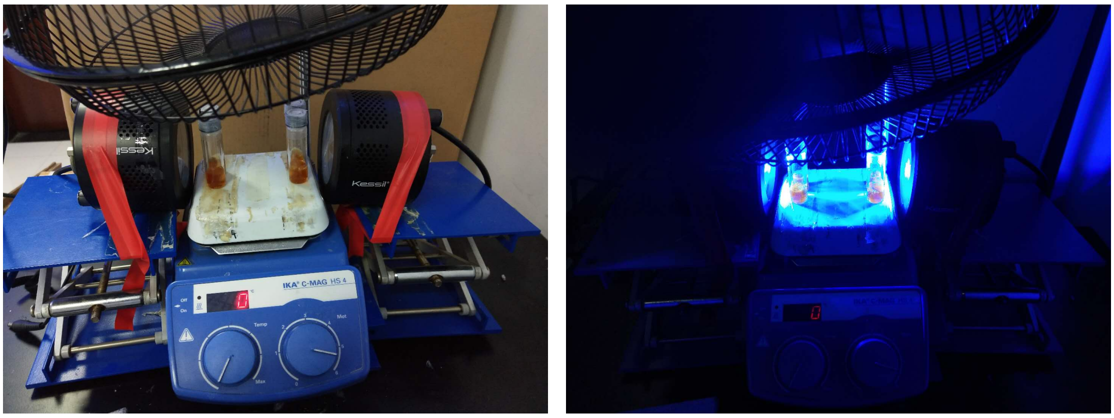


**Figure S1. Setup of this metallaphotoredox manifold.**

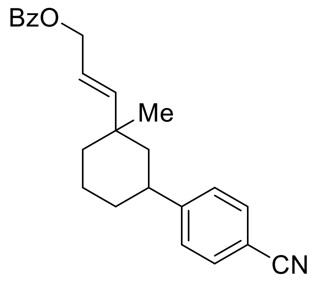


**(E)-3-(6-(4-cyanophenyl)-8-methyl-1,4-dioxaspiro[4.5]decan-8-yl)allyl benzoate (4)**

To a flame-dried 8 mL reaction vial was charged with $NiCl_2(Py)_4$ (0.02 mmol, 8.9 mg, 20 mol%), dtbbpy (0.02 mmol, 5.4 mg, 20 mol%), $Ir[dF(CF_3)ppy]_2(dtbbpy)PF_6$ (0.002 mmol, 2.2 mg, 2 mol%), 4-Bromobenzonitrile (0.2 mmol, 36.4 mg, 2.0 equiv.) and cesium 2-((1-methylcyclohexyl)oxy)-2-oxoacetate (0.3 mmol, 95.4 mg, 3 equiv.), 4,4'-dimethoxybenzophenone (0.01 mmol, 2.42 mg, 10 mol%.) , the vial was capped. DMSO (2 mL) was added via a syringe, nitrogen bubbling for fifteen minutes, followed by the addition of prop-2-yn-1-yl benzoate (14.5 uL, 0.1 mmol, 1.0 equiv.). The reaction mixture was then irradiated with a 90 W blue LED lamp (at approximately 1.5 cm away from the light source) with cooling from a fan for 18 h. The reaction was quenched with $H_2O$, extracted with ethyl acetate. The combined organic layers were dried with MgSO4, filtered, and concentrated in vacuo. The crude material was purified by flash chromatography PE: EA=20:1) as a pale yellow oil (25.1 mg, 70%, *dr* = 1:1.2). Separate one of the single structures， the nuclear magnetic data of one of the configurations is as follows.$^1$H NMR (600 MHz, $CDCl_3$) δ 8.07 (dd, *J* = 8.2, 1.1 Hz, 2H), 7.56 (d, *J* = 8.4 Hz,3H), 7.46 (d, *J* = 7.9 Hz, 2H), 7.28 (d, *J* = 8.2 Hz, 2H), 5.87 (d, *J* = 16.0 Hz, 1H), 5.72 (dd, *J* = 14.3, 8.0 Hz, 1H), 4.85 (d, *J* = 6.3 Hz, 2H), 2.81 – 2.59 (m, 1H), 1.93 – 1.79 (m,3H), 1.75 – 1.67 (m, 1H), 1.63 – 1.54 (m, 1H), 1.44 – 1.39 (m, 1H), 1.37-1.31 (m, 2H), 1.05 (s, 3H). $^{13}$C NMR (150 MHz, $CDCl_3$) δ 166.42, 152.85, 143.29, 132.95, 132.22, 130.39, 129.61, 128.38, 127.69, 122.49, 119.12, 109.73, 66.07, 45.61, 40.36, 37.17, 37.08, 33.39, 31.71, 22.62.

**HRMS (ESI+):** m/z calcd. for $C_{24}H_{26}O_2^+$ $[M+H]^+$: 360.1964; Found: 360.1958.

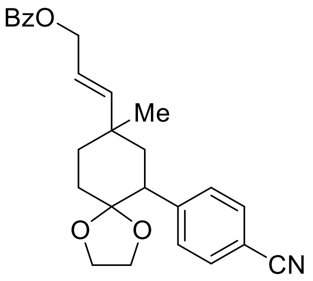

**(E)-3-(6-(4-cyanophenyl)-8-methyl-1,4-dioxaspiro[4.5]decan-8-yl)allylbenzoate (5)**

To a flame-dried 8 mL reaction vial was charged with $NiCl_2(Py)_4$ (0.02 mmol, 8.9 mg, 20mol%), dtbbpy (0.02 mmol, 5.4 mg, 20 mol%), $Ir[dF(CF_3)ppy]_2(dtbbpy)PF_6$ (0.002 mmol, 2.2 mg, 2 mol%), 4-Bromobenzonitrile (0.2 mmol, 36.4 mg, 2.0 equiv.) and cesium-2-((8-methyl-1,4-dioxaspiro[4.5]decan-8-yl)oxy)-2-oxoacetate (0.3 mmol, 112.8 mg, 3 equiv.), 4,4'-dimethoxybenzophenone (0.01 mmol, 2.42 mg, 10 mol%.) , the vial was capped. DMSO (2 mL) was added via a syringe, nitrogen bubbling for fifteen minutes, followed by the addition of prop-2-yn-1-yl benzoate (14.5 uL, 0.1 mmol, 1.0 equiv.). The reaction mixture was then irradiated with a 90 W blue LED lamp (at approximately 1.5 cm away from the light source) with cooling from a fan for 18 h. The reaction was quenched with $H_2O$, extracted with ethyl acetate. The combined organic layers were dried with $MgSO_4$, filtered, and concentrated in vacuo. The product was isolated by flash chromatography (PE: EA= 20:1) as a pale yellow oil (32.1 mg, 77%, *dr* = 1:1). $^1$H NMR (600 MHz, $CDCl_3$) δ 8.10 (t, *J* = 18.5 Hz, 2H), 7.69 – 7.53 (m, 3H), 7.52 – 7.36 (m, 4H), 5.88 (dt, *J* = 25.5, 12.6 Hz, 1H), 5.77 (dt, *J* = 16.0, 6.2 Hz, 0.5H), 5.70 (dt, *J* = 15.7, 6.3 Hz, 0.5H), 4.98 – 4.74 (m, 2H), 3.82 – 3.65 (m, 2H), 3.51 – 3.38 (m, 1H), 3.19 (dd, *J* = 13.7, 3.3 Hz, 0.5H), 3.06 (dt, *J* = 14.7, 7.4 Hz,0.5H), 3.03 – 2.94 (m, 1H), 2.19-2.13 (m, 1H), 1.89 – 1.68 (m, 4H), 1.60 – 1.52 (m, 1H), 1.23 (s, 1.5H), 1.13 (s, 1.5H).$^{13}$C NMR (150 MHz, $CDCl_3$) δ 166.41, 146.51, 146.37, 146.32, 142.15, 132.99, 132.94, 131.32, 130.72, 130.58, 130.33, 130.29, 129.64, 128.39, 128.36, 122.88, 119.91, 119.16, 119.13, 110.36, 110.29, 110.00, 109.76, 65.90, 65.88, 64.93, 64.75, 64.71, 47.63, 47.05, 41.31, 40.46, 36.49, 35.55, 34.72, 34.25, 33.79, 32.86, 32.26, 30.92, 29.71, 21.75.

**HRMS (ESI+):** m/z calcd. for $C_{26}H_{27}O_4NNa^+$ $[M+Na]^+$: 440.1838; Found: 440.1832.

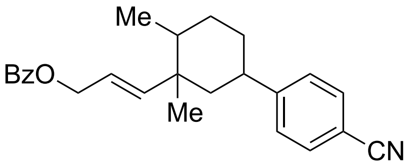


**6a**

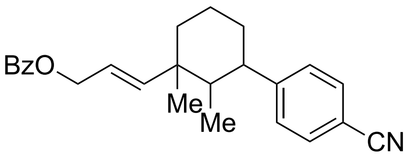


**6b**

**(E)-3-(5-(4-cyanophenyl)-1,2-dimethylcyclohexyl)allyl benzoate and (E)-3-(3-(4-cyanophenyl)-1,2-dimethylcyclohexyl)allyl benzoate (6)**

To a flame-dried 8 mL reaction vial was charged with $NiCl_2(Py)_4$ (0.02 mmol, 8.9 mg, 20mol%), dtbbpy (0.02 mmol, 5.4 mg, 20 mol%), $Ir[dF(CF_3)ppy]_2(dtbbpy)PF_6$ (0.002 mmol, 2.2 mg, 2 mol%), 4-Bromobenzonitrile (0.2 mmol, 36.4 mg, 2.0 equiv.) and cesium cesium 2-((1,2-dimethylcyclohexyl)oxy)-2-oxoacetate (0.3 mmol, 99.6 mg, 3 equiv.), 4,4'-dimethoxybenzophenone (0.01 mmol, 2.42 mg, 10 mol%.) ,the vial was capped. DMSO (2 mL) was added via a syringe, nitrogen bubbling for fifteen minutes, followed by the addition of prop-2-yn-1-yl benzoate (14.5 uL, 0.1 mmol, 1.0 equiv.). The reaction mixture was then irradiated with a 90 W blue LED lamp (at approximately 1.5 cm away from the light source) with cooling from a fan for 18 h. The reaction was quenched with $H_2O$, extracted with ethyl acetate. The combined organic layers were dried with MgSO4, filtered, and concentrated in vacuo. The product was isolated by flash chromatography (PE: EA= 20:1) as a pale yellow oil (20.1 mg, 54%, $dr_{6a}$ = 1：1.2；$dr_{6b}$ =1：4.7； rr = 4:1).$^1$H NMR (600 MHz, $CDCl_3$) δ 8.14 – 8.02 (m, 2H), 7.61-7.58 (m, 3H), 7.47 (dt, *J* = 15.5, 7.7 Hz, 2H), 7.32 (dd, *J* = 18.3, 8.2 Hz, 1.8H), 7.24 (d, *J* = 8.1 Hz, 0.2H), 5.80 – 5.65 (m, 1.6H), 5.59 (dt, *J* = 15.8, 6.4 Hz, 0.4H), 4.92 – 4.90 (m, 0.8H), 4.89 (d, *J* = 5.4 Hz, 0.2H), 4.82 (d, *J* = 6.4 Hz,1H), 2.84 – 2.71 (m, 0.8H), 2.55 (dt, *J* = 19.8, 8.0 Hz, 0.2H), 1.87 (t, *J* = 13.3 Hz, 2H), 1.82 – 1.70 (m, 2H), 1.63 – 1.57 (m, 1H), 1.38-1.34 (m, 2H), 1.28 (s, 3H), 0.82 (td, *J* = 7.5, 2.3 Hz, 3H). $^{13}$C NMR (150 MHz, $CDCl_3$) δ 166.43, 153.06, 152.85, 149.10, 149.05, 145.54, 141.99, 132.97, 132.91, 132.27, 132.25, 132.15, 130.40, 129.61, 129.60, 129.17, 128.41, 128.35, 127.79, 127.72, 124.22, 123.22, 120.72, 119.13, 109.81, 109.71, 66.07, 43.70, 41.68, 40.51, 40.27, 39.35, 39.19, 37.03, 35.79, 34.87, 33.81, 33.76, 33.31, 30.39, 29.72, 26.70, 26.38, 22.49, 22.15, 21.55, 14.04, 8.07, 7.68.

(Chiralpak IC column with guard, 10% IPA, 90% hexane, 1 mL/min, 25ºC, λ= 254 nm, 5μL injection)

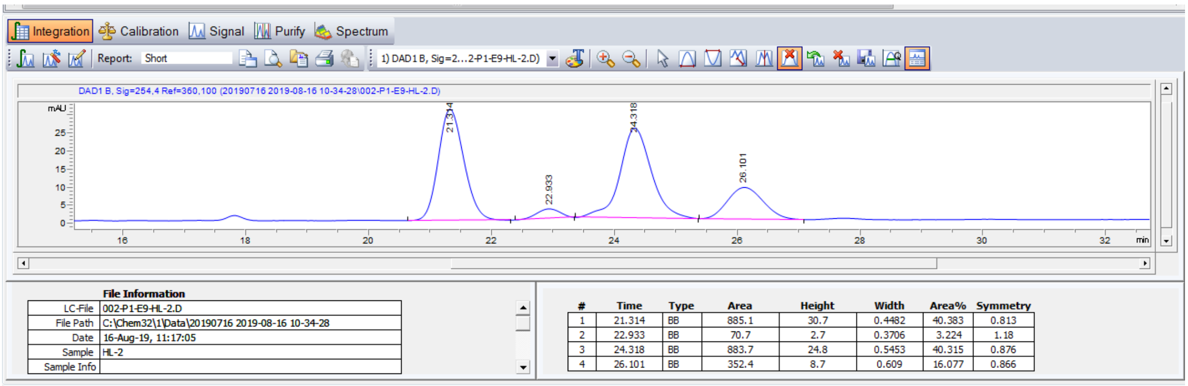


| # | Time | Type | Area | Height | Width | Area% | Symmetry |
|---|---|---|---|---|---|---|---|
| 1 | 21.314 | BB | 885.1 | 30.7 | 0.4482 | 40.383 | 0.813 |
| 2 | 22.933 | BB | 70.7 | 2.7 | 0.3706 | 3.224 | 1.18 |
| 3 | 24.318 | BB | 883.7 | 24.8 | 0.5453 | 40.315 | 0.876 |
| 4 | 26.101 | BB | 352.4 | 8.7 | 0.609 | 16.077 | 0.866 |

**HRMS (ESI+):** m/z calcd. for $C_{25}H_{27}O_2NNa^+$ $[M+Na]^+$: 396.1939; Found: 396.1934.

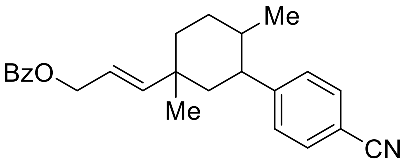


**(*E*)-3-(3-(4-cyanophenyl)-1,4-dimethylcyclohexyl)allyl benzoate (7)**

To a flame-dried 8 mL reaction vial was charged with $NiCl_2$•$Py_4$ (0.02 mmol, 8.9 mg, 20 mol%), dtbbpy (0.02 mmol, 5.4 mg, 20 mol%), Ir[dF($CF_3$)ppy]$_2$(dtbbpy)$PF_6$ (0.002 mmol, 2.2 mg, 2 mol%), 4-Bromobenzonitrile (0.2 mmol, 36.4 mg, 2.0 equiv.) and cesium 2-((1,4-dimethylcyclohexyl)oxy)-2-oxoacetate (0.3 mmol, 99.6 mg, 3 equiv.), 4,4'-dimethoxybenzophenone (0.01 mmol, 2.42 mg, 10 mol%.) ,the vial was capped. DMSO/DME = 4:1 (2.5mL) was added via a syringe, nitrogen bubbling for fifteen minutes, followed by the addition of prop-2-yn-1-yl benzoate (14.5 uL, 0.1 mmol, 1.0 equiv.). The reaction mixture was then irradiated with a 90 W blue LED lamp (at approximately 1.5 cm away from the light source) for 18h. The reaction was quenched with $H_2O$, extracted with ethyl acetate. The combined organic layers were dried with $MgSO_4$, filtered, and concentrated in vacuo. **7** was isolated by flash chromatography (PE: EA= 20:1) as a pale yellow oil (13.4 mg, 36%, *dr* = 1:1.1). Separate one of the single structures, the nuclear magnetic data of one of the configurations is as follows. $^1H$ NMR (600 MHz, $CDCl_3$) δ 8.09 – 8.06 (m, 2H), 7.59 – 7.54 (m, 3H), 7.49 – 7.43

(m, 2H), 7.23 (d, $J$ = 8.2 Hz, 2H), 5.86 (d, $J$ = 16.0 Hz, 1H), 5.70 (dt, $J$ = 16.0, 6.4 Hz, 1H), 4.88 – 4.84 (m, 2H), 2.37 – 2.31 (m, 1H), 1.84 – 1.79 (m, 1H), 1.76 – 1.72 (m, 1H), 1.67 – 1.64 (m, 1H), 1.45 – 1.40 (m, 2H), 1.03 (s, 3H), 0.88 (dd, $J$ = 9.6, 4.5 Hz, 2H), 0.63 (d, $J$ = 6.5 Hz, 3H). $^{13}$C NMR (150 MHz, $CDCl_3$) δ 166.44, 151.93, 143.31, 132.97, 132.25, 130.39, 129.60, 128.40, 128.37, 122.46, 119.15, 109.75, 66.07, 48.26, 46.70, 37.69, 37.21, 37.03, 31.48, 31.37, 20.10.

**HRMS (ESI+):** m/z calcd. for $C_{25}H_{27}O_2NNa^+$ $[M+Na]^+$: 396.1939; Found:396.1937 .

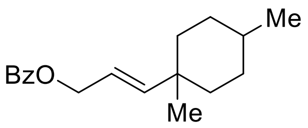


**3-(1,4-dimethylcyclohexyl)allyl benzoate (7')**

**7'** was isolated by flash chromatography (PE: EA= 20:1) as a pale yellow oil (5.4 mg, 20%, Z/E = 2:1). $^1$H NMR (600 MHz, $CDCl_3$) δ 8.13 – 8.01 (m, 2H), 7.55 (t, $J$ = 7.3 Hz, 1H), 7.48 – 7.39 (m, 2H), 5.99 – 5.74 (m, 0.65H), 5.73 – 5.32 (m, 1.35H), 4.93 – 4.65 (m, 2H), 1.86 – 1.62 (m, 2H), 1.58 – 1.31 (m, 3H), 1.24 (d, $J$ = 12.4 Hz, 2H), 1.22 – 1.02 (m, 3H), 1.02 – 0.94 (m, 2H), 0.94 – 0.57 (m, 3H). $^{13}$C NMR (150 MHz, CDCl3) δ 166.47, 143.77, 140.71, 132.86, 130.42, 129.62, 128.35, 128.33, 123.49, 121.91, 118.92, 66.21, 65.76, 44.60, 44.25, 37.78, 36.65, 36.45, 31.41, 31.16, 29.72, 20.33.

**HRMS (ESI+):** m/z calcd. for $C_{18}H_{24}O_2Na^+$ $[M+Na]^+$:295.1674 ; Found: 295.1677.

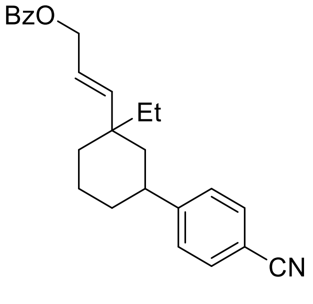


**(E)-3-(3-(4-cyanophenyl)-1-ethylcyclohexyl)allyl benzoate (8)**

To a flame-dried 8 mL reaction vial was charged with $NiCl_2(Py)_4$ (0.02 mmol, 8.9 mg, 20 mol%), dtbbpy (0.02 mmol, 5.4 mg, 20 mol%), $Ir[dF(CF_3)ppy]_2(dtbbpy)PF_6$ (0.002 mmol, 2.2 mg, 2 mol%), 4-Bromobenzonitrile (0.2 mmol, 36.4 mg, 2.0 equiv.) and

cesium 2-((1-ethylcyclohexyl)oxy)-2-oxoacetate (0.3 mmol, 99.6 mg, 3 equiv.), 4,4'-dimethoxybenzophenone (0.01 mmol, 2.42 mg, 10 mol%.) ,the vial was capped. DMSO (2 mL) was added via a syringe, nitrogen bubbling for fifteen minutes, followed by the addition of prop-2-yn-1-yl benzoate (14.5 uL, 0.1 mmol, 1.0 equiv.). The reaction mixture was then irradiated with a 90 W blue LED lamp (at approximately 1.5 cm away from the light source) with cooling from a fan for 18 h. The reaction was quenched with $H_2O$, extracted with ethyl acetate. The combined organic layers were dried with $MgSO_4$, filtered, and concentrated in vacuo. The product was isolated by flash chromatography (PE: EA= 20:1) as a pale yellow oil (14.9 mg, 40%, *dr* = 1:3.3).$^1$H NMR (600 MHz, $CDCl_3$) δ 8.08 (dt, *J* = 8.4, 4.1 Hz, 2H), 7.62 – 7.51 (m, 3H), 7.50 – 7.44 (m, 2H), 7.30 (d, *J* = 8.3 Hz, 0.45H), 7.20 (d, *J* = 8.1 Hz, 1.5H), 6.22 (d, *J* = 15.9 Hz, 0.23H), 5.86 (d, *J* = 15.8 Hz, 0.77H), 5.79 – 5.71 (m, 1H), 5.01 – 4.76 (m, 2H), 2.95 – 2.87 (m, 0.24H), 2.86 – 2.78 (m, 0.77H), 2.17 – 2.02 (m, 1H), 1.71 – 1.65 (m, 1H), 1.59 – 1.51 (m, 1H), 1.50 – 1.36 (m, 5H), 1.20 – 1.14 (m, 1H), 1.12 (s,0.7H), 1.08 (s, 2.3H), 0.92 – 0.89 (m, 1H). $^{13}$C NMR (150 MHz, $CDCl_3$) δ 166.44, 153.06, 149.09, 143.59, 141.99, 132.96, 132.24, 132.14, 130.42, 129.60, 129.17, 128.40, 127.72, 124.22, 123.23, 119.16, 109.72, 109.45, 66.06, 66.02, 43.70, 40.51, 40.27, 39.31, 37.03, 35.79, 34.87, 33.76, 30.39, 26.38, 22.49, 22.15, 7.67.

**HRMS (ESI+):** *m/z* calcd. for $C_{25}H_{27}O_2NNa^+$ $[M+Na]^+$: 396.1939; Found: 396.1934.

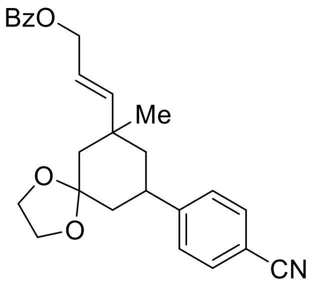


**(*E*)-3-(9-(4-cyanophenyl)-7-methyl-1,4-dioxaspiro[4.5]decan-7-yl)allyl benzoate(9)**

To a flame-dried 8 mL reaction vial was charged with $NiCl_2(Py)_4$ (0.02 mmol, 8.9 mg, 20 mol%), dtbbpy (0.02 mmol, 5.4 mg, 20 mol%), $Ir[dF(CF_3)ppy]_2(dtbbpy)PF_6$ (0.002 mmol, 2.2 mg, 2 mol%), 4-Bromobenzonitrile (0.2 mmol, 36.4 mg, 2.0 equiv.) and

cesium 2-((7-methyl-1,4-dioxaspiro[4.5]decan-7-yl)oxy)-2-oxoacetate (0.3 mmol, 112.8 mg, 3 equiv.), 4,4'-dimethoxybenzophenone (0.01 mmol, 2.42 mg, 10 mol%.) , the vial was capped. DMSO (2 mL) was added via a syringe, nitrogen bubbling for fifteen minutes, followed by the addition of prop-2-yn-1-yl benzoate (14.5 uL, 0.1 mmol, 1.0 equiv.). The reaction mixture was then irradiated with a 90 W blue LED lamp (at approximately 1.5 cm away from the light source) with cooling from a fan for 18 h. The reaction was quenched with $H_2O$, extracted with ethyl acetate. The combined organic layers were dried with $MgSO_4$, filtered, and concentrated in vacuo. The product was isolated by flash chromatography (PE: EA= 20:1) as a pale yellow oil (14.9 mg, 40%, *dr* = 1:1.86). $^1$HNMR (400 MHz, ) δ 8.05 (dd, *J* = 14.3, 7.7 Hz, 2H), 7.61 – 7.52 (m, 3H), 7.43 (dd, *J* = 13.9, 7.0 Hz, 2H), 7.30 (t, *J* = 7.7 Hz, 2H), 6.31 (d, *J* = 15.9 Hz,0.35H), 5.80 (d, *J* = 15.6 Hz, 0.65H), 5.73 – 5.58 (m, 1H), 4.87 – 4.83 (m, 0.7H), 4.77 (d, *J* = 6.2 Hz, 1.3H), 4.04 – 3.93 (m, 2H), 3.91 (d, *J* = 6.6 Hz, 2H), 3.17-3.08 (m, 1H), 1.99 – 1.80 (m, 2H), 1.65 (dd, *J* = 13.0, 8.9 Hz, 3H), 1.49 (dd, *J* = 19.3, 6.4 Hz, 1H), 1.29 (s, 1.95H), 1.11 (s, 1.05H).$^{13}$C NMR (101 MHz, $CDCl_3$) δ 166.42, 166.37, 151.15, 151.05, 146.54, 142.93, 132.96, 132.92, 132.38, 130.49, 130.29, 129.62, 128.36, 127.86, 127.77, 122.73, 120.63, 119.64, 118.99, 110.15, 110.09, 108.98, 108.88, 66.03, 65.74, 64.89, 64.69, 63.91, 63.75, 45.97, 45.29, 45.20, 44.22, 43.62, 41.54, 41.41, 37.96, 37.77, 37.68, 37.46, 29.97, 29.71, 23.63.

**HRMS (ESI+):** *m/z* calcd. for $C_{26}H_{27}O_4NNa^+$ $[M+Na]^+$: 440.1838 ; Found: 440.1841

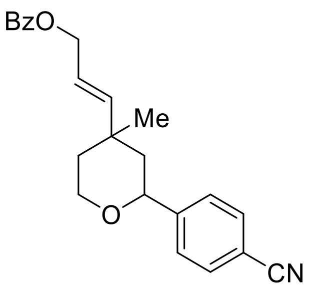


**(*E*)-3-(2-(4-cyanophenyl)-4-methyltetrahydro-2H-pyran-4-yl)allyl benzoate (10)**

To a flame-dried 8 mL reaction vial was charged with $NiCl_2(Py)_4$ (0.02 mmol, 8.9 mg, 20mol%), dtbbpy (0.02 mmol, 5.4 mg, 20 mol%), $Ir[dF(CF_3)ppy]_2(dtbbpy)PF_6$ (0.002 mmol, 2.2 mg, 2 mol%), 4-Bromobenzonitrile (0.2 mmol, 36.4 mg, 2.0 equiv.) and

cesium 2-((4-methyltetrahydro-2H-pyran-4-yl)oxy)-2-oxoacetate (0.3 mmol, 96 mg, 3 equiv.), 4,4'-dimethoxybenzophenone (0.01 mmol, 2.42 mg, 10 mol%.) ,the vial was capped. DMSO (2 mL) was added via a syringe, nitrogen bubbling for fifteen minutes, followed by the addition of prop-2-yn-1-yl benzoate (14.5 uL, 0.1 mmol, 1.0 equiv.). The reaction mixture was then irradiated with a 90 W blue LED lamp (at approximately 1.5 cm away from the light source) with cooling from a fan for 18 h. The reaction was quenched with $H_2O$, extracted with ethyl acetate. The combined organic layers were dried with $MgSO_4$, filtered, and concentrated in vacuo. The product was isolated by flash chromatography (PE: EA= 10:1) as a pale yellow oil (27.08 mg,75%, *dr* = 1:1.1). Separation causes the dr ratio (*dr* = 1:1.1) to become 1:4. $^1$H NMR (600 MHz, $CDCl_3$) δ 8.07 (d, *J* = 7.5 Hz, 1.6H), 8.04 (d, *J* = 7.8 Hz, 0.4H), 7.61 (d, *J* = 8.2 Hz, 2H), 7.59 – 7.54 (m, 1H), 7.48 – 7.40 (m, 4H), 5.93 (d, *J* = 16.0 Hz, 0.8H), 5.82 – 5.74 (m, 1H), 5.65 (tt, *J* = 15.7, 6.3 Hz, 0.2H), 4.91 – 4.86 (m, 1.6H), 4.81 – 4.74 (m, 0.4H), 4.59 (dt, *J* = 23.4, 11.7 Hz, 0.2H), 4.55 – 4.47 (m, 0.8H), 4.06 (dd, *J* = 11.8, 4.5 Hz, 0.2H), 4.00 (dt, *J* = 6.3, 3.4 Hz, 0.8H), 3.86 – 3.81 (m, 0.2H), 3.76 (ddd, *J* = 15.1, 12.7, 7.7 Hz, 0.8H), 1.91 – 1.77 (m, 1H), 1.75 – 1.68 (m, 1.7H), 1.63 – 1.57 (m, 0.3H), 1.56 – 1.44 (m, 1H), 1.30 (s, 0.6H), 1.09 (s, 2.4H). $^{13}$C NMR (151 MHz, $CDCl_3$) δ 166.60, 166.57, 148.63, 146.16, 142.04, 133.29, 133.20, 132.39, 130.41, 129.83, 128.65, 128.58, 126.57, 126.54, 123.83, 120.35, 119.16, 111.24, 111.19, 75.26, 74.58, 66.00, 65.87, 65.09, 64.29, 45.98, 44.87, 37.09, 36.12, 35.51, 34.77, 31.53, 21.89. **HRMS (ESI+):** *m/z* calcd. for $C_{23}H_{23}O_3NNa^+$ $[M+Na]^+$: 384.1576; Found: 384.1570.

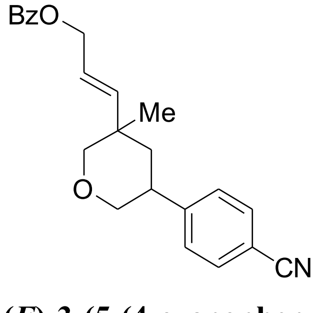


**(*E*)-3-(5-(4-cyanophenyl)-3-methyltetrahydro-2H-pyran-3-yl)allyl benzoate (11)**

To a flame-dried 8 mL reaction vial was charged with $NiCl_2(Py)_4$ (0.02 mmol, 8.9 mg, 20mol%), dtbbpy (0.02 mmol, 5.4 mg,20 mol%), $Ir[dF(CF_3)ppy]_2(dtbbpy)PF_6$ (0.002

mmol, 2.2 mg, 2 mol%), 4-Bromobenzonitrile (0.2 mmol, 36.4 mg, 2.0 equiv.) andcesium 2-((3-methyltetrahydro-2H-pyran-3-yl)oxy)-2-oxoacetate (0.3 mmol, 96 mg, 3 equiv.), 4,4'-dimethoxybenzophenone (0.01 mmol, 2.42 mg, 10 mol%.) ,the vial was capped. DMSO (2 mL) was added via a syringe, nitrogen bubbling for fifteen minutes, followed by the addition of prop-2-yn-1-yl benzoate (14.5 uL, 0.1 mmol, 1.0 equiv.). The reaction mixture was then irradiated with a 90 W blue LED lamp (at approximately 1.5 cm away from the light source) with cooling from a fan for 18h. The reaction was quenched with $H_2O$, extracted with ethyl acetate. The combined organic layers were dried with $MgSO_4$, filtered, and concentrated in vacuo. The product was isolated by flash chromatography (PE: EA= 10:1) as a pale yellow oil (18 mg ,50 %, *dr* = 1:1.3).$^1$H NMR (400 MHz, $CDCl_3$) δ 8.03 (dd, *J* = 28.1, 21.0 Hz, 2H), 7.73 – 7.51 (m, 3H), 7.45 (dd, *J* = 11.0, 4.2 Hz, 2H), 7.34 – 7.25 (m, 2H), 6.17 – 5.67 (m, 2H), 5.01 – 4.67 (m, 2H), 4.05 – 3.60 (m, 2H), 3.36 – 3.03 (m, 3H), 2.07 – 1.67 (m, 2H), 1.34 (s, 1.3H), 0.99 (s, 1.7H).$^{13}$C NMR (100 MHz, $CDCl_3$) δ 166.39, 147.33, 147.27, 141.49, 140.66, 133.03, 132.97, 132.41, 132.38, 130.35, 130.21, 129.64, 128.38, 128.27, 128.20, 123.63, 122.67, 118.77, 110.78, 110.70, 76.16, 75.86, 73.04, 72.95, 65.82, 65.53, 42.53, 41.10, 39.28, 38.82, 36.43, 25.38, 21.61.

**HRMS** (ESI): *m/z* calcd. for $C_{23}H_{23}NO_3Na^+$ $[M+Na]^+$: 384.1569. Found: 384.1570

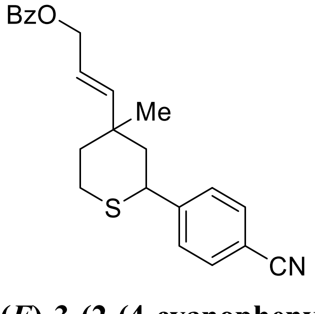


**(*E*)-3-(2-(4-cyanophenyl)-4-methyltetrahydro-2H-thiopyran-4-yl)allyl benzoate(12)**

To a flame-dried 8 mL reaction vial was charged with $NiCl_2(Py)_4$ (0.02 mmol, 8.9 mg, 20 mol%), dtbbpy (0.02 mmol, 5.4 mg, 20 mol%), $Ir[dF(CF_3)ppy]_2(dtbbpy)PF_6$ (0.002 mmol, 2.2 mg, 2 mol%), 4-Bromobenzonitrile (0.2 mmol,36.4 mg, 2.0 equiv.) and cesium 2-((4-methyltetrahydro-2H-thiopyran-4-yl)oxy)-2-oxoacetate (0.3 mmol, 100.8

mg, 3 equiv.), 4,4'-dimethoxybenzophenone (0.01 mmol, 2.42 mg, 10 mol%.) ,the vial was capped. DMSO (2 mL) was added via a syringe, nitrogen bubbling for fifteen minutes, followed by the addition of prop-2-yn-1-yl benzoate (14.5 uL, 0.1 mmol, 1.0 equiv.). The reaction mixture was then irradiated with a 90 W blue LED lamp (at approximately 1.5 cm away from the light source) with cooling from a fan for 18 h. The reaction was quenched with $H_2O$, extracted with ethyl acetate. The combined organic layers were dried with $MgSO_4$, filtered, and concentrated in vacuo. The product was isolated by flash chromatography (PE: EA= 10:1) as a pale yellow oil (28.3 mg, 75%, dr=1:1.2). Separate one of the single structures，the nuclear magnetic data of one of the configurations is as follows.$^1$H NMR (600 MHz, $CDCl_3$) δ 8.09 (d, *J* = 7.7 Hz, 2H), 7.61 (t, *J* = 9.2 Hz, 3H), 7.49 (t, *J* = 7.7 Hz, 2H), 7.45 (d, *J* = 8.1 Hz, 2H), 5.83 (d, *J* = 16.1 Hz, 1H), 5.72 (dt, *J* = 16.1, 6.2 Hz, 1H), 4.88 (d, *J* = 6.1 Hz, 2H), 4.11 (d, *J* = 12.1 Hz, 1H), 3.11 (td, *J* = 13.5, 1.9 Hz, 1H), 2.57 (dt, *J* = 13.9, 3.3 Hz, 1H), 2.12 (d, *J* = 13.5 Hz, 2H), 1.89 (t, *J* = 13.0 Hz, 1H), 1.72 (td, *J* = 13.6, 3.4 Hz, 1H), 1.13 (s, 3H). $^{13}$C NMR (150 MHz, $CDCl_3$) δ 166.37, 147.72, 141.66, 133.10, 132.45, 130.18, 129.62, 128.45, 128.35, 123.90, 118.75, 111.16, 65.78, 46.33, 42.58, 37.60, 36.93, 32.08, 26.57. **HRMS (ESI+):** *m/z* calcd. for $C_{23}H_{23}O_2NNaS^+$ $[M+Na]^+$: 400.1347; Found: 400.1342.

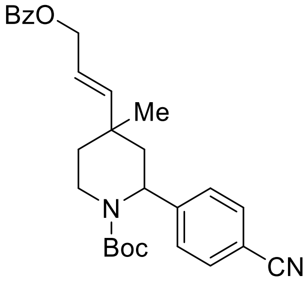


***Tert*-butyl (E)-4-(3-(benzoyloxy)prop-1-en-1-yl)-2-(4-cyanophenyl)-4-ethyl piperidine-1-carboxylate (13)**

To a flame-dried 8 mL reaction vial was charged with $NiCl_2(Py)_4$ (0.02 mmol, 8.9 mg, 20 mol%), dtbbpy (0.02 mmol, 5.4 mg, 20 mol%), $Ir[dF(CF_3)ppy]_2(dtbbpy)PF_6$ (0.002 mmol, 2.2 mg, 2 mol%), 4-Bromobenzonitrile (0.2 mmol, 36.4 mg, 2.0 equiv.) and cesium 2-((1-(tert-butoxycarbonyl)-4-methylpiperidin-4-yl)oxy)-2-oxoacetate (0.3 mmol, 125.7 mg, 3 equiv.), 4,4'-dimethoxybenzophenone (0.01 mmol, 2.42 mg, 10

mol%.) ,the vial was capped. DMSO (2 mL) was added via a syringe, nitrogen bubbling for fifteen minutes, followed by the addition of prop-2-yn-1-yl benzoate (14.5 uL, 0.1 mmol, 1.0 equiv.). The reaction mixture was then irradiated with a 90 W blue LED lamp (at approximately 1.5 cm away from the light source) with cooling from a fan for 18h. The reaction was quenched with $H_2O$, extracted with ethyl acetate. The combined organic layers were dried with $MgSO_4$, filtered, and concentrated in vacuo. The product was isolated by flash chromatography (PE: EA= 10:1) as a pale yellow oil (34.5 mg, 76%, *dr* = 1:1.4). $^1$H NMR (600 MHz, $CDCl_3$) δ 8.11 – 8.02 (m, 1.17H), 8.00 – 7.95 (m, 0.83H), 7.62 (d, *J* = 8.3 Hz, 1H), 7.59 – 7.54 (m, 1H), 7.51 (d, *J* = 8.3 Hz, 0.85H), 7.45 (dt, *J* = 15.7, 7.9 Hz, 2H), 7.30 (d, *J* = 8.1 Hz, 1.15H), 7.24 (d, *J* = 8.1 Hz, 1H), 5.80 (d, *J* = 15.8 Hz, 0.6H), 5.65 (dt, *J* = 15.8, 6.2 Hz, 0.6H), 5.41 (t, *J* = 3.4 Hz, 0.8H), 5.20 (dt, *J* = 10.8, 5.5 Hz, 1H), 4.80 (dd, *J* = 6.2, 0.9 Hz, 1.1H), 4.54 – 4.38 (m, 0.9H), 4.15 – 4.10 (m, 0.59H), 4.09 – 4.04 (m, 0.41H), 3.27 – 3.18 (m, 0.41H), 3.15 (ddd, *J* = 13.8, 12.4, 3.5 Hz, 0.59H), 2.09 (dd, *J* = 13.8, 5.0 Hz, 0.41H), 1.98 (d, *J* = 6.0 Hz, 1H), 1.96 – 1.80 (m, 0.59H), 1.78 – 1.71 (m, 1H), 1.56 (dd, *J* = 13.0, 7.9 Hz, 0.41H), 1.44 (dd, *J* = 9.9, 2.7 Hz,0.59H), 1.35 (d, *J* = 3.5 Hz, 9H), 1.09 (s, 1.22H), 0.75 (s, 1.68H).$^{13}$C NMR (150MHz, $CDCl_3$) δ 166.37, 166.11, 155.38, 155.30, 148.85, 148.75, 144.98, 142.17, 133.05, 133.02, 132.40, 132.17, 130.21, 130.08, 129.63, 129.58, 128.42, 128.39, 126.31, 126.10, 121.06, 120.83, 118.89, 118.82, 110.23, 110.13, 80.32, 80.30, 65.65, 65.32, 53.39, 53.06, 41.83, 40.34, 37.91, 37.40, 35.32, 35.01, 34.25, 34.03, 29.71, 29.36, 28.27, 25.11.

HRMS (ESI+): *m/z* calcd. for $C_{28}H_{32}O_4N_2Na^+$ $[M+Na]^+$: 483.2260; Found: 483.2254.

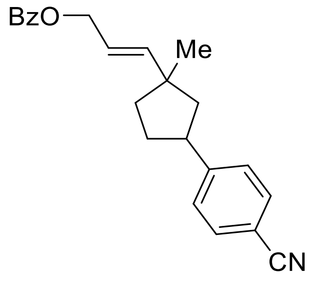


**(*E*)-3-(3-(4-cyanophenyl)-1-methylcyclopentyl)allyl benzoate (14)**

To a flame-dried 8 mL reaction vial was charged with $NiCl_2(Py)_4$ (0.02 mmol, 8.9 mg, 20 mol%), dtbbpy (0.02 mmol, 5.4 mg, 20 mol%), Ir[dF($CF_3$)ppy]$_2$(dtbbpy)$PF_6$ (0.002 mmol, 2.2 mg, 2 mol%), 4-Bromobenzonitrile (0.2 mmol, 36.4 mg, 2.0 equiv.) and cesium 2-((1-methylcyclopentyl)oxy)-2-oxoacetate (0.3 mmol, 91.2 mg, 3 equiv.), 4,4'-dimethoxybenzophenone (0.01 mmol, 2.42 mg, 10 mol%.) ,the vial was capped. DMSO (2 mL) was added via a syringe, nitrogen bubbling for fifteen minutes, followed by the addition of prop-2-yn-1-yl benzoate (14.5 uL, 0.1 mmol, 1.0 equiv.). The reaction mixture was then irradiated with a 90 W blue LED lamp (at approximately 1.5 cm away from the light source) with cooling from a fan for 18h. The reaction was quenched with $H_2O$, extracted with ethyl acetate. The combined organic layers were dried with $MgSO_4$, filtered, and concentrated in vacuo. The product was isolated by flash chromatography (PE: EA= 20:1) as a pale yellow oil (13.8 mg, 40 %, *dr* = 1:1.3). $^1$H NMR (600 MHz, $CDCl_3$) δ 8.15 – 8.05 (m, 2H), 7.60 (dt, *J* = 12.7, 10.4 Hz, 3H), 7.52 – 7.46 (m, 2H), 7.38 – 7.32 (m, 2H), 6.00 (dd, *J* = 20.6, 15.7 Hz, 1H), 5.71 (ddt, *J* = 18.1, 15.6, 6.4 Hz, 1H), 4.84 (dt, *J* = 18.9, 9.5 Hz, 2H), 3.40 – 3.32 (m,0.57H), 3.29 – 3.21 (m,0.43H), 2.30 – 1.62 (m, 6H), 1.26 (s, 1.3H), 1.24 (s, 1.7H). $^{13}$C NMR (150 MHz, $CDCl_3$) δ 166.43, 166.41, 151.83, 151.76, 145.47, 144.85, 132.94, 132.19, 132.17, 130.36, 130.30, 129.62, 128.36, 127.91, 127.86, 119.97, 119.85, 119.11, 109.66, 65.88, 65.85, 47.99, 47.94, 44.80, 44.70, 44.66, 44.46, 39.44, 38.99, 33.22, 33.21, 27.70, 26.24. **HRMS (ESI+)**: *m/z* calcd. for $C_{23}H_{23}O_2N^+$ [M+Na]$^+$: 368.1626; Found: 368.1621.

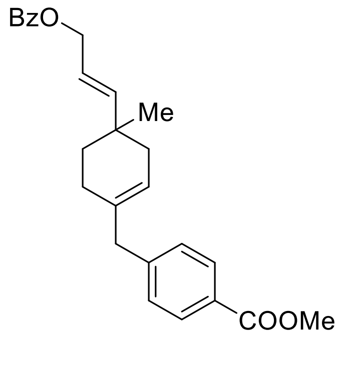


**Methyl (*E*)-4-((4-(3-(benzoyloxy)prop-1-en-1-yl)-4-methylcyclohex-1-en-1-yl)methyl)benzoate (15)**

To a flame-dried 8 mL reaction vial was charged with $NiCl_2(Py)_4$ (0.02 mmol, 8.9 mg,20 mol%), dtbbpy (0.02 mmol, 5.4 mg,20 mol%), $Ir[dF(CF_3)ppy]_2(dtbbpy)PF_6$ (0.002 mmol, 2.2 mg, 2 mol%), ethyl 4-bromobenzoate (0.2 mmol, 43.0 mg, 2.0 equiv.) and cesium 2-((1-methyl-4-methylenecyclohexyl)oxy)-2-oxoacetate (0.2 mmol, 65.8 mg, 2 equiv.), the vial was capped. DMSO (2 mL) was added via a syringe, nitrogen bubbling for fifteen minutes, followed by the addition of prop-2-yn-1-yl benzoate (14.5 uL, 0.1 mmol, 1.0 equiv.). The reaction mixture was then irradiated with a 90 W blue LED lamp (at approximately 1.5 cm away from the light source) with cooling from a fan for 24 h. The reaction was quenched with $H_2O$, extracted with ethyl acetate. The combined organic layers were dried with $MgSO_4$, filtered, and concentrated in vacuo. The product was isolated by flash chromatography (PE: EA= 20:1) as a pale yellow oil (25.9 mg, 64%).$^1$H NMR (400 MHz, $CDCl_3$) δ 8.05 (d, $J$ = 7.5 Hz, 2H), 7.93 (d, $J$ = 8.0 Hz, 2H), 7.56 (t, $J$ = 7.4 Hz, 1H), 7.44 (t, $J$ = 7.6 Hz, 2H), 7.21 (d, $J$ = 8.0 Hz, 2H), 5.80 (d, $J$ = 15.7 Hz, 1H), 5.61 (dt, $J$ = 15.7, 6.3 Hz, 1H), 5.45 (s, 1H), 4.84 – 4.72 (m, 2H), 3.89 (s, 3H), 3.29 (s, 2H), 2.08 (t, $J$ = 14.5 Hz, 1H), 1.94 – 1.75 (m, 3H), 1.54 – 1.38 (m, 2H), 1.00 (s, 3H). $^{13}$C NMR (100 MHz, $CDCl_3$) δ 167.16, 166.44, 145.86, 144.58, 135.49, 132.85, 130.47, 129.61, 129.59, 128.81, 128.33, 128.01, 121.97, 120.38, 66.07, 51.95, 44.17, 36.93, 34.14, 33.98, 25.87, 25.71.

**HRMS** (ESI+): *m/z* calcd. for $C_{26}H_{28}O_4Na^+$ $[M+Na]^+$: 427.1876 Found: 427.1880.

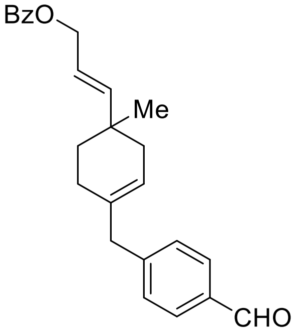


**(*E*)-3-(4-(4-formylbenzyl)-1-methylcyclohex-3-en-1-yl)allylbenzoate (16)**

To a flame-dried 8 mL reaction vial was charged with $NiCl_2(Py)_4$ (0.02 mmol, 8.9 mg, 20 mol%), dtbbpy (0.02 mmol, 5.4 mg, 20 mol%), $Ir[dF(CF_3)ppy]_2(dtbbpy)PF_6$ (0.002 mmol, 2.2mg, 2 mol%), 4-Bromobenzaldehyde (0.2 mmol, 37.0 mg, 2.0 equiv.) and

cesium 2-((1-methyl-4-methylenecyclohexyl)oxy)-2-oxoacetate (0.2 mmol, 65.8 mg, 2 equiv.), the vial was capped. DMSO (2 mL) was added via a syringe, nitrogen bubbling for fifteen minutes, followed by the addition of prop-2-yn-1-yl benzoate (14.5 uL, 0.1 mmol, 1.0 equiv.). The reaction mixture was then irradiated with a 90 W blue LED lamp (at approximately 1.5 cm away from the light source) with cooling from a fan for 24 h. The reaction was quenched with $H_2O$, extracted with ethyl acetate. The combined organic layers were dried with $MgSO_4$, filtered, and concentrated in vacuo. The product was isolated by flash chromatography (PE: EA= 20:1) as a pale yellow oil (22.4 mg, 60%).$^1$H NMR (400 MHz, $CDCl_3$) δ 9.94 (s, 1H), 8.05 (d, *J* = 7.3 Hz, 2H), 7.76 (d, *J* = 8.0 Hz, 2H), 7.56 (t, *J* = 7.2 Hz, 1H), 7.44 (t, *J* = 7.7 Hz, 2H), 7.31 (d, *J* = 8.0 Hz, 2H), 5.80 (d, *J* = 15.7 Hz, 1H), 5.62 (dt, *J* = 15.7, 6.3 Hz, 1H), 5.47 (s, 1H), 4.87 – 4.68 (m, 2H), 3.32 (s, 2H), 2.11 (d, *J* = 17.6 Hz, 1H), 2.00 – 1.78 (m, 3H), 1.456-1.42 (m, Hz, 2H), 1.01 (s, 3H). $^{13}$C NMR (100 MHz, $CDCl_3$) δ 191.98, 166.43, 147.82, 144.46, 135.23, 134.69, 132.90, 130.46, 129.83, 129.62, 129.43, 128.36, 122.33, 120.46, 66.06, 44.35, 36.89, 34.15, 33.96, 26.02, 25.78.

**HRMS** (ESI+): *m/z* calcd. for $C_{25}H_{26}O_3Na^+$ $[M+Na]^+$: 397.1775. Found: 397.1774.

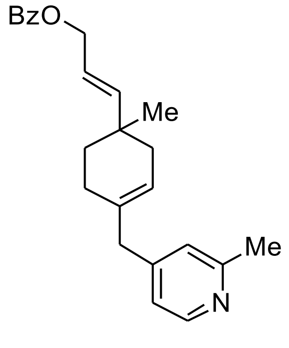


**(*E*)-3-(1-methyl-4-((2-methylpyridin-4-yl)methyl)cyclohex-3-en-1-yl)allyl benzoate (17)**

To a flame-dried 8 mL reaction vial was charged with $NiCl_2(Py)_4$ (0.02 mmol, 8.9 mg, 20 mol%), dtbbpy (0.02 mmol, 5.4 mg, 20 mol%), $Ir[dF(CF_3)ppy]_2(dtbbpy)PF_6$ (0.002 mmol, 2.2 mg, 2 mol%), 4-Bromo-2-methylpyridine (0.2 mmol, 34.4 mg, 2.0 equiv.) and cesium 2-((1-methyl-4-methylenecyclohexyl)oxy)-2-oxoacetate (0.2 mmol, 65.8 mg, 2 equiv.), the vial was capped. DMSO (2 mL) was added via a syringe, nitrogen

bubbling for fifteen minutes, followed by the addition of prop-2-yn-1-yl benzoate (14.5 uL, 0.1 mmol, 1.0 equiv.). The reaction mixture was then irradiated with a 90 W blue LED lamp (at approximately 1.5 cm away from the light source) with cooling from a fan for 24h. The reaction was quenched with $H_2O$, extracted with ethyl acetate. The combined organic layers were dried with $MgSO_4$, filtered, and concentrated in vacuo. The product was isolated by flash chromatography (PE: EA= 10:1) as a pale yellow oil (21.6 mg, 60%).$^1$H NMR (400 MHz, $CDCl_3$) δ 8.34 (d, *J* = 5.1 Hz, 1H), 8.10 – 8.00 (m, 2H), 7.55 (t, *J* = 7.4 Hz, 1H), 7.43 (t, *J* = 7.6 Hz, 2H), 6.98 – 6.84 (m, 2H), 5.80 (d, *J* = 15.7 Hz, 1H), 5.61 (dt, *J* = 15.7, 6.3 Hz, 1H), 5.45 (s, 1H), 4.82 – 4.71 (m, 2H), 3.18 (s, 2H), 2.51 (s, 3H), 2.10 (d, *J* = 17.5 Hz, 1H), 1.94 – 1.80 (m, 3H), 1.55 – 1.40 (m, 2H), 1.00 (s, 3H). $^{13}$C NMR (100 MHz, $CDCl_3$) δ 166.41, 158.17, 149.61, 148.92, 144.50, 134.61, 132.88, 130.44, 129.61, 128.34, 123.73, 122.47, 121.29, 120.43, 66.04, 43.44, 36.90, 34.10, 33.92, 25.88, 25.70, 24.33.

**HRMS** (ESI+): *m/z* calcd. for $C_{24}H_{27}NO_2^+$ $[M+H]^+$: 362.2111 ; Found: 362.2115.

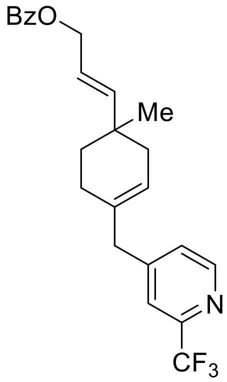


**(*E*)-3-(1-methyl-4-((2-(trifluoromethyl)pyridin-4-yl)methyl)cyclohex-3-en-1-yl)allyl benzoate (18)**

To a flame-dried 8 mL reaction vial was charged with $NiCl_2(Py)_4$ (0.02 mmol, 8.9 mg, 20 mol%), dtbbpy (0.02 mmol, 5.4 mg, 20 mol%), Ir[dF($CF_3$)ppy]$_2$(dtbbpy)$PF_6$ (0.002 mmol, 2.2 mg, 2 mol%), 4-bromo-2-(trifluoromethyl)pyridine, (0.2 mmol, 45.0 mg, 2.0 equiv.) and cesium 2-((1-methyl-4-methylenecyclohexyl)oxy)-2-oxoacetate (0.2 mmol, 65.8 mg, 2 equiv.), the vial was capped. DMSO (2 mL) was added via a syringe, nitrogen bubbling for fifteen minutes, followed by the addition of prop-2-yn-1-yl

benzoate (14.5 uL, 0.1 mmol, 1.0 equiv.). The reaction mixture was then irradiated with a 90 W blue LED lamp (at approximately 1.5 cm away from the light source) with cooling from a fan for 24 h. The reaction was quenched with $H_2O$, extracted with ethyl acetate. The combined organic layers were dried with $MgSO_4$, filtered, and concentrated in vacuo. The product was isolated by flash chromatography (PE: EA= 10:1) as a pale yellow oil (24 mg, 58%). $^1$H NMR (400 MHz, $CDCl_3$) δ 8.51 (s, 1H), 8.05 (d, $J$ = 7.5 Hz, 2H), 7.62 (d, $J$ = 8.1 Hz, 1H), 7.56 (t, $J$ = 7.3 Hz, 2H), 7.44 (t, $J$ = 7.7 Hz, 2H), 5.79 (d, $J$ = 15.8 Hz, 1H), 5.61 (dt, $J$ = 15.7, 6.3 Hz, 1H), 5.46 (s, 1H), 4.85 – 4.73 (m, 2H), 3.31 (s, 2H), 2.10 (d, $J$ = 17.4 Hz, 1H), 1.95 – 1.78 (m, 3H), 1.60 – 1.40 (m, 2H), 1.01 (s, 3H). $^{19}$F NMR (377 MHz, $CDCl_3$) δ -67.67 (s,3F). $^{13}$C NMR (100 MHz, $CDCl_3$) δ 166.42, 150.43, 146.17 (d, $J$ = 34.6 Hz), 144.23, 139.01, 137.27, 134.42, 132.91, 130.42, 129.60, 128.36, 121.85 (d, $J$ = 252.2 Hz), 120.36, 120.08 (q, $J$ = 2.7 Hz). 65.97, 40.90, 36.80, 34.10, 33.86, 26.01, 25.79.

**HRMS** (ESI+): *m/z* calcd. for $C_{24}H_{24}F_3NO_2N^+$ $[M+H]^+$: 416.1828; Found:416.1832.

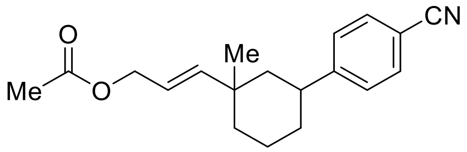


**(E)-3-(3-(4-cyanophenyl)-1-methylcyclohexyl)allyl acetate (19)**

To a flame-dried 8 mL reaction vial was charged with $NiCl_2(Py)_4$ (0.02 mmol, 8.9 mg, 20 mol%), dtbbpy (0.02 mmol, 5.4 mg, 20 mol%), $Ir[dF(CF_3)ppy]_2(dtbbpy)PF_6$ (0.002 mmol, 2.2mg, 2 mol%), 4-Bromobenzonitrile (0.2 mmol, 36.4 mg, 2.0 equiv.) and cesium 2-((1-methylcyclohexyl)oxy)-2-oxoacetate (0.3 mmol, 95.4 mg, 3 equiv.), 4,4'-dimethoxybenzophenone (0.01 mmol, 2.42mg, 10 mol%.) ,the vial was capped. DMSO (2 mL) was added via a syringe, nitrogen bubbling for fifteen minutes, followed by the addition of prop-2-yn-1-yl acetate (9.8 mg, 0.1 mmol, 1.0 equiv.). The reaction mixture was then irradiated with a 90 W blue LED lamp (at approximately 1.5 cm away from the light source) with cooling from a fan for 18 h. The reaction was quenched with $H_2O$, extracted with ethyl acetate. The combined organic layers were dried with $MgSO_4$, filtered, and concentrated in vacuo. The product was isolated by flash chromatography

(PE: EA= 20:1) as a pale yellow oil (19 mg, 54%, *dr* = 1:1).$^{1}$H NMR (400 MHz, $CDCl_3$) δ 7.58 (t, *J* = 10.2 Hz, 2H), 7.32 (dd, *J* = 8.2, 3.9 Hz, 2H), 5.78 (dd, *J* = 15.8, 9.7 Hz, 1H), 5.67 – 5.45 (m, 1H), 4.61 (d, *J* = 6.5 Hz, 1H), 4.54 (dd, *J* = 6.4, 0.8 Hz, 1H), 2.97 – 2.67 (m, 1H), 2.11 (s, 1.5H), 2.07 (d, 5.6 Hz, 1.5H), 1.97 – 1.74 (m, 3H), 1.68-1.53 (m, 1H), 1.50 – 1.25 (m, 4H), 1.16 (s, 1.5H), 1.05 (s, 1.5H). $^{13}$C NMR (100 MHz, $CDCl_3$) δ 170.88, 152.85, 152.71, 147.54, 143.07, 132.24, 127.77, 127.69, 122.40, 119.21, 119.13, 119.10, 109.81, 109.74, 65.65, 65.57, 45.49, 44.32, 40.31, 39.75, 37.15, 37.02, 36.28, 36.13, 33.40, 31.71, 22.59, 22.23, 21.92, 21.10, 21.06.

**HRMS (ESI+)**: calcd. for $C_{19}H_{23}O_2NNa^+$ $[M+Na]^+$: 320.1597, found 320.1621.

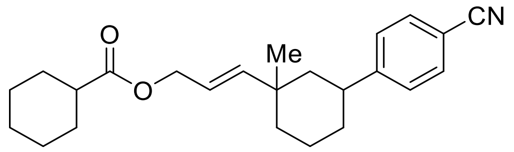


**(*E*)-3-(3-(4-cyanophenyl)-1-methylcyclohexyl)allyl cyclohexanecarboxylate (20)**

To a flame-dried 8 mL reaction vial was charged with $NiCl_2(Py)_4$ (0.02 mmol, 8.9 mg, 20 mol%), dtbbpy (0.02 mmol, 5.4 mg, 20 mol%), $Ir[dF(CF_3)ppy]_2(dtbbpy)PF_6$ (0.002 mmol, 2.2 mg, 2 mol%), 4-Bromobenzonitrile (0.2 mmol, 36.4 mg, 2.0 equiv.) and cesium 2-((1-methylcyclohexyl)oxy)-2-oxoacetate (0.3 mmol, 95.4 mg, 3 equiv.), 4,4'-dimethoxybenzophenone (0.01 mmol, 2.42 mg, 10 mol%.) ,the vial was capped. DMSO (2 mL) was added via a syringe, nitrogen bubbling for fifteen minutes, followed by the addition of prop-2-yn-1-yl cyclohexanecarboxylate (16.6 mg, 0.1 mmol, 1.0 equiv.). The reaction mixture was then irradiated with a 90 W blue LED lamp (at approximately 1.5 cm away from the light source) with cooling from a fan for 12h. The reaction was quenched with $H_2O$, extracted with ethyl acetate. The combined organic layers were dried with $MgSO_4$, filtered, and concentrated in vacuo. The product was isolated by flash chromatography (PE: EA= 20:1) as a pale yellow oil (25.5mg, 70%, *dr* = 1:1.2). Separate one of the single structures，the nuclear magnetic data of one of the configurations is as follows.$^{1}$H NMR (600 MHz, $CDCl_3$) δ 7.60 (d, *J* = 8.3 Hz, 2H), 7.33 (d, *J* = 8.2 Hz, 2H), 5.77 (t, *J* = 16.3 Hz, 1H), 5.51 (dt, *J* = 15.7, 6.3 Hz, 1H), 4.55 (dd, *J* = 9.4, 8.6 Hz, 2H), 2.84 (tt, *J* = 12.5, 3.3 Hz, 1H), 2.36 – 2.28 (m, 1H), 1.92 (d,

$J$ = 10.1 Hz, 3H), 1.81 – 1.75 (m, 3H), 1.71 – 1.64 (m, 2H), 1.54 (d, $J$ = 13.1 Hz, 1H), 1.49 – 1.42 (m, 3H), 1.40 – 1.31 (m, 3H), 1.28 – 1.22 (m, 3H), 1.16 (s, 3H). $^{13}$C NMR (150 MHz, $CDCl_3$) δ 175.93, 152.74, 147.13, 132.24, 127.78, 119.45, 119.11, 109.80, 65.14, 44.35, 43.24, 39.78, 36.25, 36.17, 33.41, 29.04, 25.76, 25.46, 22.26, 21.94.
**HRMS (ESI+)**: calcd for $C_{24}H_{31}O_2NNa^+$ $[M+Na]^+$: 388.2252, found 388.2247.

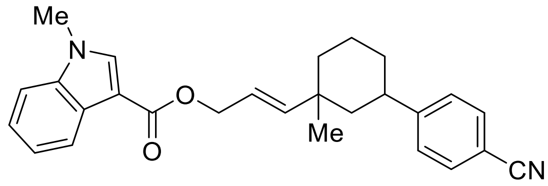


**(*E*)-3-(3-(4-cyanophenyl)-1-methylcyclohexyl)allyl 1-methyl-1H-indole-3-carboxylate(21)**

To a flame-dried 8 mL reaction vial was charged with $NiCl_2(Py)_4$ (0.02 mmol, 8.9 mg, 20 mol%), dtbbpy (0.02 mmol, 5.4 mg, 20 mol%), Ir[dF($CF_3$)ppy]$_2$(dtbbpy)$PF_6$ (0.002 mmol, 2.2 mg, 2 mol%), 4-Bromobenzonitrile (0.2 mmol, 36.4 mg, 2.0 equiv.) and cesium 2-((1-methylcyclohexyl)oxy)-2-oxoacetate (0.3 mmol, 95.4 mg, 3 equiv.), 4,4'-dimethoxybenzophenone (0.01 mmol, 2.42 mg, 10 mol%.) ,the vial was capped. DMSO (2 mL) was added via a syringe, nitrogen bubbling for fifteen minutes, followed by the addition of prop-2-yn-1-yl 1-methyl-1H-indole-3-carboxylate (21.3mg, 0.1 mmol, 1.0 equiv.). The reaction mixture was then irradiated with a 90 W blue LED lamp (at approximately 1.5 cm away from the light source) for with cooling from a fan 18 h. The reaction was quenched with $H_2O$, extracted with ethyl acetate. The combined organic layers were dried with $MgSO_4$, filtered, and concentrated in vacuo. The product was isolated by flash chromatography (PE: EA= 20:1) as a pale yellow oil (30.4 mg, 74%, *dr* = 1:1.8). $^1$H NMR (600 MHz, $CDCl_3$) δ 8.21 (dd, $J$ = 12.1, 7.6 Hz, 1H), 7.84 (t, $J$ = 12.3 Hz, 1H), 7.58 (dt, $J$ = 20.0, 10.0 Hz, 2H), 7.39 (t, $J$ = 9.1 Hz, 1H), 7.35 – 7.29 (m, 4H), 5.88 (dd, $J$ = 26.9, 15.9 Hz, 1H), 5.79 (tt, $J$ = 12.4, 6.1 Hz, 0.65H), 5.69 (dt, $J$ = 15.7, 6.3 Hz, 0.35H), 4.88 (d, $J$ = 6.2 Hz, 1.3H), 4.84 – 4.78 (m, 0.7H), 3.87 (s, 1.9H), 3.86 (s, 1.1H), 2.90 – 2.77 (m, 1H), 1.94 – 1.63 (m, 4H), 1.52 – 1.29 (m, 4H), 1.19 (s, 1.1H), 1.08 (s, 1.9H). $^{13}$C NMR (150 MHz, $CDCl_3$) δ 164.84, 164.80, 152.97,

152.80, 146.87, 142.59, 137.23, 135.28, 135.24, 132.24, 132.21, 127.80, 127.72, 126.62, 123.21, 122.83, 122.79, 121.87, 121.69, 121.67, 120.04, 119.18, 109.85, 109.77, 109.66, 107.07, 64.80, 64.72, 45.68, 44.40, 40.37, 39.81, 37.27, 37.04, 36.29, 36.23, 33.48, 33.44, 31.77, 22.64, 22.33, 21.97 .

HRMS (ESI+): calcd. for $C_{27}H_{28}N_2O_2Na^+[M+Na]^+$: 435.2043, found: 435.2043.

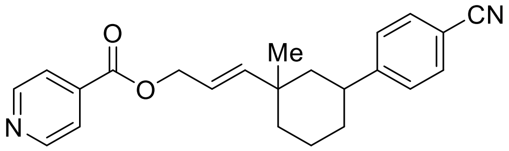


**(*E*)-3-(3-(4-cyanophenyl)-1-methylcyclohexyl)allyl isonicotinate (22)**

To a flame-dried 8 mL reaction vial was charged with $NiCl_2(Py)_4$ $(PPh_3)_2$(0.02 mmol,13.08 mg, 20 mol%), dtbbpy (0.02 mmol, 5.4 mg, 20 mol%), $Ir[dF(CF_3)ppy]_2(dtbbpy)PF_6$ (0.002 mmol, 2.2 mg, 2 mol%), 4-Bromobenzonitrile (0.2 mmol, 36.4 mg, 2.0 equiv.) and cesium 2-((1-methylcyclohexyl)oxy)-2-oxoacetate (0.3 mmol, 95.4 mg, 3 equiv.), $NH_4Cl$(0.2 mmol, 2 equiv.),the vial was capped. DMSO (2 mL) [0.05 M] was added via a syringe, nitrogen bubbling for fifteen minutes, followed by the addition of prop-2-yn-1-yl isonicotinate (16.1 mg, 0.1 mmol, 1.0 equiv.). The reaction mixture was then irradiated with a 90 W blue LED lamp (at approximately 1.5 cm away from the light source) for 18h. The reaction was quenched with $H_2O$, extracted with ethyl acetate. The combined organic layers were dried with $MgSO_4$, filtered, and concentrated in vacuo. The product was isolated by flash chromatography (PE: EA= 20:1) as a pale yellow oil (19 mg, 53%, *dr* = 1:1). $^1H$ NMR (600 MHz, $CDCl_3$) δ 8.79 (d, *J* = 5.0 Hz, 1H), 8.77 (d, *J* = 5.0 Hz, 1H), 7.87 (d, *J* = 5.1 Hz, 1H), 7.85 (d, *J* = 5.0 Hz, 1H), 7.57 (d, *J* = 8.1 Hz, 2H), 7.28 (dt, *J* = 12.1, 6.1 Hz, 2H), 5.87 (dd, *J* = 25.6, 15.8 Hz, 1H), 5.70 (dt, *J* = 15.9, 6.5 Hz, 0.5H), 5.61 (dt, *J* = 15.5, 6.5 Hz,0.5H), 4.88 (d, *J* = 6.5 Hz, 1H), 4.81 (d, *J* = 6.5 Hz, 1H), 2.82-2.72 (m, 0.5H), 2.71 (dd, *J* = 17.2, 7.8 Hz, 0.5H), 1.95 – 1.79 (m, 2H), 1.72 – 1.53 (m, 3H), 1.49 – 1.29 (m, 3H), 1.16 (s, 1.5H), 1.05 (s, 1.5H). $^{13}C$ NMR (150 MHz, $CDCl_3$) δ 164.97, 164.94, 152.71, 152.62, 150.63, 150.58, 148.52, 144.32, 137.52, 132.26, 127.77, 127.67, 122.90, 122.89,

121.72, 119.10, 118.58, 109.84, 109.78, 66.94, 66.84, 45.48, 44.28, 40.38, 39.73, 37.16, 37.08, 36.42, 36.10, 33.38, 33.34, 31.66, 22.61, 22.22, 21.89.

**HRMS (ESI+)**: calcd. for $C_{23}H_{24}N_2O_2^+$[M+H] $^+$361.1916, found 361.1911.

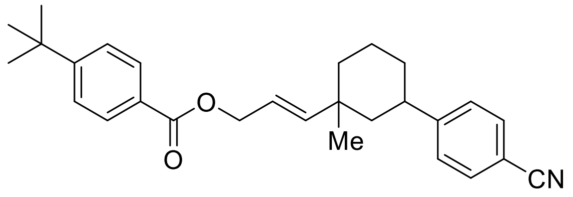

**(*E*)-3-(3-(4-cyanophenyl)-1-methylcyclohexyl)allyl 4-(tert-butyl)benzoate (23)**

To a flame-dried 8 mL reaction vial was charged with $NiCl_2(Py)_4$ (0.02 mmol,8.9 mg,20 mol%), dtbbpy (0.02 mmol, 5.4 mg, 20 mol%), Ir[dF($CF_3$)ppy]$_2$(dtbbpy)$PF_6$ (0.002 mmol, 2.2 mg, 2 mol%), 4-Bromobenzonitrile (0.2 mmol, 36.4 mg, 2.0 equiv.) and cesium 2-((1-methylcyclohexyl)oxy)-2-oxoacetate (0.3 mmol, 95.4 mg, 3 equiv.), 4,4'-dimethoxybenzophenone (0.01 mmol, 2.42 mg, 10 mol%.) ,the vial was capped. DMSO (2 mL) was added via a syringe, nitrogen bubbling for fifteen minutes, followed by the addition of prop-2-yn-1-yl 4-(tert-butyl)benzoate (21.6 mg, 0.1 mmol, 1.0 equiv.). The reaction mixture was then irradiated with a 90 W blue LED lamp (at approximately 1.5 cm away from the light source) with cooling from a fan for 18h. The reaction was quenched with $H_2O$, extracted with ethyl acetate. The combined organic layers were dried with $MgSO_4$, filtered, and concentrated in vacuo. The product was isolated by flash chromatography (PE: EA= 20:1) as a pale yellow oil (29 mg, 70%, *dr* = 1:1.2). Separation causes the dr ratio (*dr* = 1:1.2) to become 1:19. The nuclear magnetic data of configurations is as follows.$^1$H NMR (600 MHz, $CDCl_3$) δ 8.00 (d, *J* = 8.5 Hz, 2H), 7.60 (d, *J* = 8.3 Hz, 2H), 7.47 (d, *J* = 8.5 Hz, 2H),7.33 (d, *J* = 8.2 Hz, 2H), 5.85 (d, *J* = 15.7 Hz, 1H), 5.73 (dt, *J* = 16.0, 6.3 Hz, 0.05H), 5.64 (dt, *J* = 15.7, 6.3 Hz, 0.95H), 4.86 (d, *J* = 6.3 Hz, 0.1H), 4.79 (d, *J* = 0.8 Hz, 1.9H), 2.85 (tt, *J* = 12.5, 3.3 Hz, 0.95H), 2.77 (t, *J* = 10.7 Hz, 0.05H), 1.91 (d, *J* = 12.7 Hz, 1H), 1.80 - 1.78(m, 1H), 1.74 – 1.53 (m, 4H), 1.47 (dd, *J* = 15.1, 10.6 Hz, 1H), 1.43 – 1.38 (m, 1H), 1.36 (s, 9H), 1.18 (s, 3H). $^{13}$C NMR (150 MHz, $CDCl_3$) δ 166.41, 156.57, 152.74, 147.32, 132.24,

129.48, 127.79, 127.70, 127.59, 125.30, 119.46, 119.11, 109.80, 65.73, 44.36, 39.79, 36.31, 36.19, 35.08, 33.41, 31.13, 22.29, 21.95.

**HRMS (ESI+):** calcd. for $C_{28}H_{33}O_2NNa^+$ $[M+Na]^+$ 438.2409 , found 438.2404.

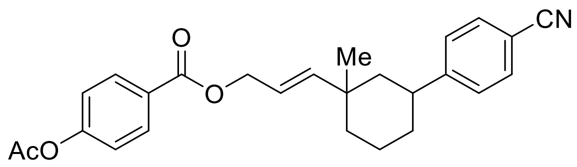


**(*E*)-3-(3-(4-cyanophenyl)-1-methylcyclohexyl)allyl 4-acetoxybenzoate (24)**

To a flame-dried 8 mL reaction vial was charged with $NiCl_2(Py)_4$ (0.02 mmol,8.9mg,20 mol%), dtbbpy (0.02 mmol, 5.4 mg, 20 mol%), $Ir[dF(CF_3)ppy]_2(dtbbpy)PF_6$ (0.002 mmol, 2.2 mg, 2 mol%), 4-Bromobenzonitrile (0.2 mmol, 36.4 mg, 2.0 equiv.) and cesium 2-((1-methylcyclohexyl)oxy)-2-oxoacetate (0.3 mmol, 95.4 mg, 3 equiv.), 4,4'-dimethoxybenzophenone (0.01 mmol, 2.42 mg, 10 mol%.) ,the vial was capped. DMSO (2 mL) was added via a syringe, nitrogen bubbling for fifteen minutes, followed by the addition of prop-2-yn-1-yl 4-acetoxybenzoate (21.8 mg, 0.1 mmol, 1.0 equiv.). The reaction mixture was then irradiated with a 90 W blue LED lamp (at approximately 1.5 cm away from the light source) with cooling from a fan for 18 h. The reaction was quenched with $H_2O$, extracted with ethyl acetate. The combined organic layers were dried with $MgSO_4$, filtered, and concentrated in vacuo. The product was isolated by flash chromatography (PE: EA= 20:1) as a pale yellow oil (27 mg, 65%, *dr* = 1:1.5). $^1H$ NMR (400 MHz, $CDCl_3$) δ 8.09 (t, *J* = 8.8 Hz, 2H), 7.57 (d, *J* = 8.1 Hz, 2H), 7.29 (dd, *J* = 11.3, 9.4 Hz, 2H), 7.18 (t, *J* = 8.2 Hz, 2H), 5.84 (t, *J* = 15.6 Hz, 2H), 5.70 (dt, *J* = 16.0, 6.3 Hz, 0.4H), 5.61 (dt, *J* = 15.7, 6.3 Hz, 0.6H), 4.84 (d, *J* = 6.2 Hz, 0.8H), 4.78 (d, *J* = 6.2 Hz, 0.2H), 2.78 (dt, *J* = 37.1, 12.5 Hz, 1H), 2.32 (d, *J* = 2.6 Hz, 3H), 1.81 (dt, *J* = 36.0, 16.5 Hz, 3H), 1.65 – 1.52 (m, 2H), 1.46 – 1.28 (m, 3H), 1.16 (s, 1.8H), 1.05 (s, 1.2H). $^{13}C$ NMR (100 MHz, $CDCl_3$) δ 168.88, 165.61, 165.59, 154.34, 154.30, 152.85, 152.73, 147.69, 143.45, 132.26, 131.21, 127.95, 127.94, 127.81, 127.71, 122.37, 121.66, 121.60, 119.20, 119.12, 109.81, 109.73, 66.17, 66.07, 45.56, 44.33, 40.36, 39.77, 37.15, 37.09, 36.35, 36.16, 33.40, 31.72, 22.62, 22.26, 21.93, 21.16.

**HRMS (ESI+):** calcd. for $C_{26}H_{27}NO_4$ $Na^+$ $[M+Na]^+$ 440.1838 , found 440.1837.

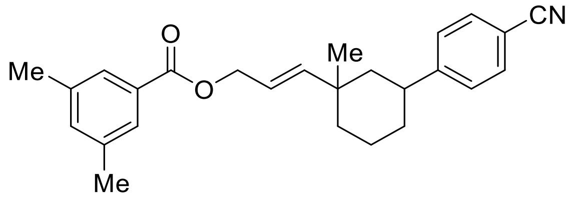


**(*E*)-3-(3-(4-cyanophenyl)-1-methylcyclohexyl)allyl 3,5-dimethylbenzoate (25)**

To a flame-dried 8 mL reaction vial was charged with $NiCl_2(Py)_4$ (0.02 mmol, 8.9 mg, 20 mol%), dtbbpy (0.02 mmol, 5.4 mg, 20 mol%), $Ir[dF(CF_3)ppy]_2(dtbbpy)PF_6$ (0.002 mmol, 2.2 mg, 2 mol%), 4-Bromobenzonitrile (0.2 mmol, 36.4 mg, 2.0 equiv.) and cesium 2-((1-methylcyclohexyl)oxy)-2-oxoacetate (0.3 mmol, 95.4 mg, 3 equiv.), 4,4'-dimethoxybenzophenone (0.01 mmol, 2.42 mg, 10 mol%.) ,the vial was capped. DMSO (2 mL) was added via a syringe, nitrogen bubbling for fifteen minutes, followed by the addition of pprop-2-yn-1-yl 3,5-dimethylbenzoatee (18.8 mg, 0.1 mmol, 1.0 equiv.). The reaction mixture was then irradiated with a 90 W blue LED lamp (at approximately 1.5 cm away from the light source) with cooling from a fan for 18 h. The reaction was quenched with $H_2O$, extracted with ethyl acetate. The combined organic layers were dried with $MgSO_4$, filtered, and concentrated in vacuo. The product was isolated by flash chromatography (PE: EA= 20:1) as a pale yellow oil (25.1 mg, 65%, *dr* = 1:19). Separation causes the dr ratio (*dr* = 1:1) to become 1:19. The nuclear magnetic data of configurations is as follows.$^1$H NMR (600 MHz, $CDCl_3$) δ 7.68 (s, 2H), 7.59 (d, *J* = 8.2 Hz, 2H), 7.33 (d, *J* = 8.2 Hz, 2H), 7.21 (d, *J* = 11.0 Hz, 1H), 5.84 (d, *J* = 15.7 Hz, 1H), 5.67 – 5.63 (m, 0.95H), 5.45 (dt, *J* = 15.7, 6.2 Hz, 0.05H), 4.79 (d, *J* = 6.4 Hz, 1.9H), 4.55 (d, *J* = 6.2 Hz, 0.1H), 2.85-2.82 (m, 1H), 2.38 (s, 6H), 1.91 (d, *J* = 12.5 Hz, 1H), 1.82 – 1.68 (m, 2H), 1.63 (d, *J* = 12.7 Hz, 1H), 1.57 (d, *J* = 13.3 Hz, 1H), 1.47 (t, *J* = 12.5 Hz, 1H), 1.42 – 1.34 (m, 2H), 1.18 (s, 2.85H), 1.10 (s, 0.15H). $^{13}$C NMR (150 MHz, $CDCl_3$) δ 166.76, 152.75, 147.50, 138.00, 134.58, 132.25, 130.19, 127.80, 127.32, 119.42, 119.13, 109.78, 65.96, 44.32, 39.78, 36.34, 36.16, 33.41, 22.28, 21.94, 21.18.

**HRMS (ESI+):** calcd. for $C_{26}H_{29}NO_2Na^+$ $[M+Na]^+$ 410.2096 , found 410.2093.

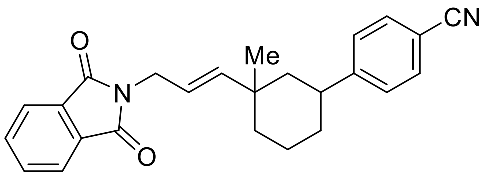

**(*E*)-4-(3-(3-(1,3-dioxoisoindolin-2-yl)prop-1-en-1-yl)-3-methyl cyclohexyl) benzonitrile(26)**

To a flame-dried 8 mL reaction vial was charged with $NiCl_2(Py)_4$ (0.02 mmol, 8.9 mg, 20 mol%), dtbbpy (0.02 mmol, 5.4 mg, 20 mol%), $Ir[dF(CF_3)ppy]_2(dtbbpy)PF_6$ (0.002 mmol, 2.2 mg, 2 mol%), 4-Bromobenzonitrile (0.2 mmol, 36.4 mg, 2.0 equiv.) and cesium 2-((1-methylcyclohexyl)oxy)-2-oxoacetate (0.3 mmol, 95.4 mg, 3 equiv.), 4,4'-dimethoxybenzophenone (0.01 mmol, 2.42 mg, 10 mol%.) ,the vial was capped. DMSO (2 mL) was added via a syringe, nitrogen bubbling for fifteen minutes, followed by the addition of 2-(prop-2-yn-1-yl)isoindoline-1,3-dione (18.5 mg, 0.1 mmol, 1.0 equiv.). The reaction mixture was then irradiated with a 90 W blue LED lamp (at approximately 1.5 cm away from the light source) with cooling from a fan for 18h. The reaction was quenched with $H_2O$, extracted with ethyl acetate. The combined organic layers were dried with $MgSO_4$, filtered, and concentrated in vacuo. The product was isolated by flash chromatography (PE: EA= 20:1) as a pale yellow oil (17.28 mg, 45%, *dr* = 1:1.2). Separation causes the dr ratio (*dr* = 1:1.2) to become *dr* = 1:4.6. The nuclear magnetic data of configurations is as follows.$^1$H NMR (600 MHz, $CDCl_3$) δ 7.92 – 7.84 (m, 2H), 7.76 – 7.71 (m, 2H), 7.58 (d, *J* = 8.2 Hz, 2H), 7.30 (d, *J* = 8.3 Hz, 2H), 5.78 (dd, *J* = 22.1, 15.9 Hz, 1H), 5.54 (dt, *J* = 15.8, 6.3 Hz, 0.18H), 5.46 (dt, *J* = 15.6, 6.3 Hz, 0.83H), 4.34 (d, *J* = 6.3 Hz, 0.35H), 4.26 (d, *J* = 6.3 Hz, 1.63H), 2.81 (t, *J* = 11.8 Hz,0.83H), 2.73 (t, *J* = 12.4 Hz, 0.17H), 1.92 – 1.64 (m, 3H), 1.53 (dd, *J* = 32.0, 13.3 Hz, 2H), 1.43 – 1.30 (m, 3H), 1.12 (s, 2.48H), 1.01 (s, 0.53H). $^{13}$C NMR (150 MHz, $CDCl_3$) δ 167.99, 152.75, 146.64, 133.91, 132.21, 127.78, 127.71, 123.26, 119.72, 119.13, 118.53, 109.74, 44.33, 39.89, 39.77, 36.25, 36.18, 33.35, 22.27, 21.91.

**HRMS (ESI+):** calcd. for $C_{25}H_{24}N_2O2Na^+$ $[M+Na]^+$ :407.1728 , found:407.1730.

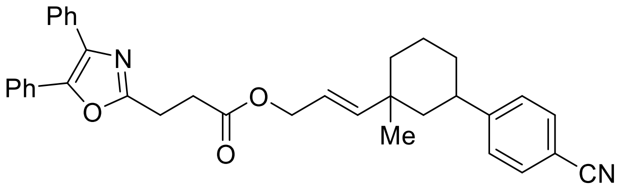


**(*E*)-3-(3-(4-cyanophenyl)-1-methylcyclohexyl)allyl 3-(4,5-diphenyloxazol-2-yl) propanoate(27)**

To a flame-dried 8 mL reaction vial was charged with $NiCl_2(Py)_4$ (0.02 mmol, 8.9 mg, 20 mol%), dtbbpy (0.02 mmol, 5.4 mg, 20 mol%), $Ir[dF(CF_3)ppy]_2(dtbbpy)PF_6$ (0.002 mmol, 2.2 mg, 2 mol%), 4-Bromobenzonitrile (0.2 mmol, 36.4 mg, 2.0 equiv.) and cesium 2-((1-methylcyclohexyl)oxy)-2-oxoacetate (0.3 mmol, 95.4 mg, 3 equiv.), 4,4'-dimethoxybenzophenone (0.01 mmol, 2.42 mg, 10 mol%.) ,the vial was capped. DMSO (2 mL) was added via a syringe, nitrogen bubbling for fifteen minutes, followed by the addition of prop-2-yn-1-yl 3-(4,5-diphenyloxazol-2-yl)propanoate (33.1 mg, 0.1 mmol, 1.0 equiv.). The reaction mixture was then irradiated with a 90 W blue LED lamp (at approximately 1.5 cm away from the light source) with cooling from a fan for 12h. The reaction was quenched with $H_2O$, extracted with ethyl acetate. The combined organic layers were dried with $MgSO_4$, filtered, and concentrated in vacuo. The product was isolated by flash chromatography (PE: EA= 20:1) as a pale yellow oil (28.09 mg, 53%, *dr* = 1:1.2). $^1$H NMR (600 MHz, $CDCl_3$) δ 7.65 (dd, *J* = 10.0, 8.5 Hz, 2H), 7.61 – 7.57 (m, 4H), 7.39 – 7.32 (m, 6H), 7.30 (dd, *J* = 7.1, 5.0 Hz, 2H), 5.78 (dd, *J* = 19.0, 16.0 Hz, 1H), 5.61 (dt, *J* = 16.0, 6.4 Hz, 0.55H), 5.52 (dt, *J* = 15.7, 6.4 Hz, 0.45H), 4.68 (d, *J* = 6.4 Hz, 1.1H), 4.61 (d, *J* = 6.3 Hz, 0.9H), 3.22 (dt, *J* = 12.6, 7.5 Hz, 2H), 2.96 (dt, *J* = 19.6, 7.6 Hz, 2H), 2.83-2.80 (m,0.45H), 2.75 – 2.68 (m, 0.55H), 1.80-1.70 (m, 4H), 1.59 – 1.47 (m, 1H), 1.38 – 1.32 (m, 3H), 1.13 (s, 1.35H), 1.03 (s, 1.65H). $^{13}$C NMR (150 MHz, $CDCl_3$) δ 171.82, 171.78, 161.71, 152.81, 152.69, 147.63, 145.43, 145.40, 143.31, 135.14, 135.12, 132.45, 132.43, 132.24, 132.23, 128.98, 128.96, 128.65, 128.56, 128.48, 128.46, 128.08, 128.06, 127.87, 127.78, 127.68, 126.45, 122.20, 119.14, 119.12, 119.03, 109.79, 109.71, 99.99, 65.96, 65.84, 45.47, 44.26, 40.31, 39.74, 37.11, 37.03, 36.27, 36.10, 33.38, 31.69, 31.21, 23.57, 22.58, 22.19, 21.91. **HRMS (ESI+):** calcd. for $C_{35}H_{35}NO_3N_2{}^+$ $[M+H]^+$ 531.2648, found 531.2642.

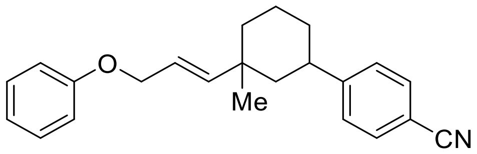

**(*E*)-4-(3-methyl-3-(3-phenoxyprop-1-en-1-yl)cyclohexyl)benzonitrile(28)**

To a flame-dried 8 mL reaction vial was charged with $NiCl_2(Py)_4$ (0.02 mmol, 8.9 mg, 20 mol%), dtbbpy (0.02 mmol, 5.4 mg, 20 mol%), $Ir[dF(CF_3)ppy]_2(dtbbpy)PF_6$ (0.002 mmol, 2.2 mg, 2 mol%), 4-Bromobenzonitrile (0.2 mmol, 36.4 mg, 2.0 equiv.) and cesium 2-((1-methylcyclohexyl)oxy)-2-oxoacetate (0.3 mmol, 95.4 mg, 3 equiv.), $NH_4Cl$(0.2mmol, 2 equiv.),the vial was capped. DMSO/EA = 4:1[0.04 M] was added via a syringe, nitrogen bubbling for fifteen minutes, followed by the addition of(prop-2-yn-1-yloxy)benzene (13.2 mg, 0.1 mmol, 1.0 equiv.). The reaction mixture was then irradiated with a 90 W blue LED lamp (at approximately 1.5 cm away from the light source) for 18 h. The reaction was quenched with $H_2O$, extracted with ethyl acetate. The combined organic layers were dried with $MgSO_4$, filtered, and concentrated in vacuo. The product was isolated by flash chromatography (PE: EA= 20:1) as a pale yellow oil (19.2mg, 58%, *dr* = 1:1.2). Separate one of the single structures， the nuclear magnetic data of one of the configurations is as follows.$^1$H NMR (600 MHz, $CDCl_3$) δ 7.60 (d, $J$ = 8.2 Hz, 2H), 7.32 (dd, $J$ = 15.1, 7.9 Hz, 4H), 7.32 (dd, $J$ = 15.1, 7.9 Hz, 3H), 5.85 (d, $J$ = 15.8 Hz, 1H), 5.67 (dt, $J$ = 15.8, 5.9 Hz, 1H), 4.52 (d, $J$ = 3.6 Hz, 2H), 2.85 (tt, $J$ = 12.5, 3.3 Hz, 1H), 1.91 (d, $J$ = 12.7 Hz, 1H), 1.82 – 1.76 (m, 1H), 1.72 – 1.62 (m, 2H), 1.57 (d, $J$ = 7.3 Hz, 1H), 1.49 (t, $J$ = 12.8 Hz, 1H), 1.45 – 1.34 (m, 2H), 1.19 (s, 3H). $^{13}$C NMR (150 MHz, $CDCl_3$) δ 158.71, 152.77, 146.55, 132.25, 129.41, 127.78, 120.73, 120.25, 119.12, 114.73, 109.79, 69.08, 44.36, 39.80, 36.31, 36.19, 33.46, 22.30, 21.97.

**HRMS (ESI+):** calcd. for $C_{23}H_{25}NONa^+[M+Na]^+$ 354.1827 , found 354.1828.

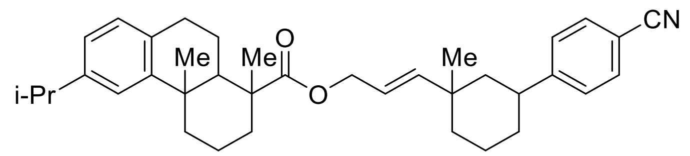


**(*E*)-3-(3-(4-cyanophenyl)-1-methylcyclohexyl)allyl 6-isopropyl-1,4a-dimethyl-1,2,3,4,4a,9,10,10a-octahydrophenanthrene-1-carboxylate (29)**

To a flame-dried 8 mL reaction vial was charged with $NiCl_2(Py)_4$ (0.02 mmol, 8.9 mg,20 mol%), dtbbpy (0.02 mmol, 5.4 mg, 20 mol%), $Ir[dF(CF_3)ppy]_2(dtbbpy)PF_6$ (0.002 mmol, 2.2 mg, 2 mol%), 4-Bromobenzonitrile (0.2 mmol, 36.4 mg, 2.0 equiv.) and cesium 2-((1-methylcyclohexyl)oxy)-2-oxoacetate (0.3 mmol, 95.4 mg, 3 equiv.), 4,4'-dimethoxybenzophenone (0.01 mmol, 2.42 mg, 10 mol%.) ,the vial was capped. DMSO (2 mL) was added via a syringe, nitrogen bubbling for fifteen minutes, followed by the addition of prop-2-yn-1-yl 6-isopropyl-1,4a-dimethyl-1,2,3,4,4a,9,10,10a-octahydrophenanthrene-1-carboxylate (33.8 mg, 0.1 mmol, 1.0 equiv.). The reaction mixture was then irradiated with a 90 W blue LED lamp (at approximately 1.5 cm away from the light source) with cooling from a fan for 18 h. The reaction was quenched with $H_2O$, extracted with ethyl acetate. The combined organic layers were dried with $MgSO_4$, filtered, and concentrated in vacuo. The product was isolated by flash chromatography (PE: EA= 20:1) as a pale yellow oil (26.5 mg, 50%, *dr* = 1:1.2). Separate one of the single structures， the nuclear magnetic data of one of the configurations is as follows. $^1H$ NMR (600 MHz, $CDCl_3$) δ 7.57 (t, $J$ = 10.4 Hz, 2H), 7.31 (d, $J$ = 8.2 Hz, 2H), 7.16 (dd, $J$ = 8.8, 5.1 Hz, 1H), 7.01 (d, $J$ = 8.0 Hz, 1H), 6.84 (s, 1H), 5.75 (t, $J$ = 19.4 Hz, 1H), 5.52 – 5.44 (m, 1H), 4.61 – 4.55 (m, 1H), 4.51 – 4.43 (m, 1H), 2.91 – 2.80 (m, 4H), 2.32 – 2.22 (m, 2H), 1.90 – 1.74 (m, 6H), 1.7-1.61 (m, 4H), 1.44 – 1.33 (m, 5H), 1.26 (s, 3H), 1.22 (dd, $J$ = 6.9, 1.3 Hz, 6H), 1.20 (s, 3H), 1.12 (s, 3H). $^{13}C$ NMR (150 MHz, $CDCl_3$) δ 178.28, 152.74, 147.49, 146.94, 145.76, 134.64, 132.25, 127.79, 126.87, 124.25, 123.96, 119.45, 119.42, 119.09, 109.82, 65.54, 47.61, 44.79, 44.39, 44.35, 39.80, 39.78, 37.98, 36.96, 36.45, 36.28, 36.26, 36.19, 36.16, 33.47, 33.43, 33.39, 30.16, 29.71, 25.18, 24.00, 23.97, 22.30, 22.27, 21.94, 21.62, 18.62, 16.54.

**HRMS (ESI+)**: calcd. for $C_{37}H_{48}NO_2^+$ $[M+H]^+$ 538.3685, found 538.3690.

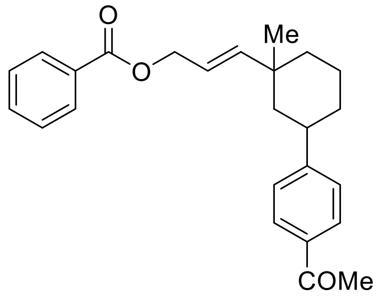


**(*E*)-3-(3-(4-acetylphenyl)-1-methylcyclohexyl)allyl benzoate (30)**

To a flame-dried 8 mL reaction vial was charged with $NiCl_2(Py)_4$ (0.02 mmol, 8.9 mg, 20 mol%), dtbbpy (0.02 mmol, 5.4 mg, 20 mol%), $Ir[dF(CF_3)ppy]_2(dtbbpy)PF_6$ (0.002 mmol, 2.2 mg, 2 mol%), 4'-Bromoacetophenone (0.2 mmol, 39.8 mg, 2.0 equiv.) and cesium 2-((1-methylcyclohexyl)oxy)-2-oxoacetate (0.3 mmol, 95.4 mg, 3 equiv.), 4,4'-dimethoxybenzophenone (0.01 mmol, 2.42 mg, 10 mol%.) ,the vial was capped. DMSO (2 mL) was added via a syringe, nitrogen bubbling for fifteen minutes, followed by the addition of prop-2-yn-1-yl benzoate (14.5 uL, 0.1 mmol, 1.0 equiv.). The reaction mixture was then irradiated with a 90 W blue LED lamp (at approximately 1.5 cm away from the light source) with cooling from a fan for 18 h. The reaction was quenched with $H_2O$, extracted with ethyl acetate. The combined organic layers were dried with $MgSO_4$, filtered, and concentrated in vacuo. The product was isolated by flash chromatography (PE: EA= 20:1) as a pale yellow oil (24.06 mg, 64%, *dr* = 1:1.3). $^1$H NMR (400 MHz, $CDCl_3$) δ 8.19 – 8.04 (m, 2H), 7.91 (dd, *J* = 8.3, 1.6 Hz, 2H), 7.64 – 7.54 (m, 1H), 7.52 – 7.43 (m, 2H), 7.32 (dd, *J* = 15.8, 7.2 Hz, 2H), 5.89 (t, *J* = 16.8 Hz, 1H), 5.80 – 5.68 (m, 0.55H), 5.65 (dt, *J* = 15.7, 6.3 Hz, 0.44H), 4.93 – 4.84 (m, 1.1H), 4.81 (dd, *J* = 6.3, 0.9 Hz, 0.9H), 2.94 – 2.72 (m, 1H), 2.61 (s, 3H), 1.96 – 1.65 (m, 4H), 1.58 – 1.31 (m, 4H), 1.19 (s, 1.31H), 1.08 (s, 1.69H). $^{13}$C NMR (100 MHz, $CDCl_3$) δ 197.84, 166.43, 153.11, 152.96, 147.82, 143.52, 135.23, 135.17, 132.93, 132.88, 130.42, 130.39, 129.62, 128.59, 128.38, 128.33, 127.16, 127.06, 122.34, 119.15, 66.16, 66.07, 45.73, 44.46, 40.24, 39.64, 37.28, 37.10, 36.37, 36.26, 33.52, 31.76, 26.57, 22.72, 22.31, 22.04.

**HRMS (ESI+):** calcd. for $C_{25}H_{29}O_3^+$ $[M+H]^+$377.2117 , found 377.2111

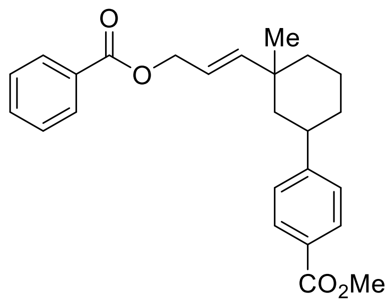


**Methyl (*E*)-4-(3-(3-(benzoyloxy)prop-1-en-1-yl)-3-methylcyclohexyl)Benzoate (31)**

To a flame-dried 8 mL reaction vial was charged with $NiCl_2(Py)_4$ (0.02 mmol,8.9mg,20 mol%), dtbbpy (0.02 mmol, 5.4 mg, 20 mol%), Ir[dF($CF_3$)ppy]$_2$(dtbbpy)$PF_6$ (0.002 mmol, 2.2 mg, 2 mol%), methyl methyl 4-bromobenzoate (0.2 mmol, 43 mg, 2.0 equiv.) and cesium 2-((1-methylcyclohexyl)oxy)-2-oxoacetate (0.3 mmol, 95.4 mg, 3 equiv.), 4,4'-dimethoxybenzophenone (0.01 mmol, 2.42 mg, 10 mol%.) ,the vial was capped. DMSO (2 mL) was added via a syringe, nitrogen bubbling for fifteen minutes, followed by the addition of prop-2-yn-1-yl benzoate (14.5 uL, 0.1 mmol, 1.0 equiv.). The reaction mixture was then irradiated with a 90 W blue LED lamp (at approximately 1.5 cm away from the light source) with cooling from a fan for 18 h. The reaction was quenched with $H_2O$, extracted with ethyl acetate. The combined organic layers were dried with $MgSO_4$, filtered, and concentrated in vacuo. The product was isolated by flash chromatography (PE: EA= 20:1) as a pale yellow oil (21.16 mg, 54%, *dr* = 1:1.2). Separate one of the single structures， the nuclear magnetic data of one of the configurations is as follows. $^1$H NMR (600 MHz, $CDCl_3$) δ 8.15 – 8.03 (m, 2H), 7.99 (t, *J* = 10.7 Hz, 2H), 7.60 – 7.54 (m, 1H), 7.50 – 7.41 (m, 2H), 7.32 – 7.29 (m, 2H), 5.87 (d, *J* = 15.7 Hz, 1H), 5.65 (dt, *J* = 15.7, 6.4 Hz, 1H), 4.81 (dd, *J* = 6.4, 0.9 Hz, 2H), 3.93 (d, *J* = 12.8 Hz, 3H), 2.86 (tt, *J* = 12.5, 3.3 Hz, 1H), 1.94 – 1.63 (m, 4H), 1.59 – 1.37 (m, 4H), 1.19 (s, 3H). $^{13}$C NMR (150 MHz, $CDCl_3$) δ 167.18, 166.48, 152.70, 147.89, 132.90, 130.38, 129.76, 129.64, 128.34, 127.93, 126.99, 119.10, 66.11, 52.00, 44.46, 39.62, 36.39, 36.26, 33.56, 22.29, 22.05.

**HRMS (ESI+)**: calcd. for $C_{25}H_{29}O_4^+$[M+H]$^+$ 393.2066 , found 393.2060

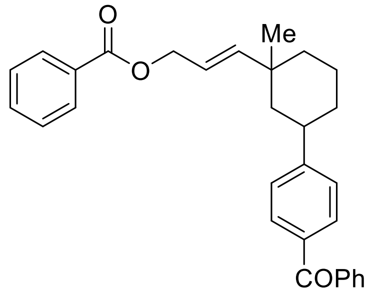


**Methyl (*E*)-4-(3-(3-(benzoyloxy)prop-1-en-1-yl)-3-methylcyclohexyl)benzoate**

**(*E*)-3-(3-(4-benzoylphenyl)-1-methylcyclohexyl)allyl benzoate(32)**

To a flame-dried 8 mL reaction vial was charged with $NiCl_2(Py)_4$ (0.02 mmol,8.9mg,20 mol%), dtbbpy (0.02 mmol, 5.4 mg, 20 mol%), $Ir[dF(CF_3)ppy]_2(dtbbpy)PF_6$ (0.002 mmol, 2.2 mg, 2 mol%), 4-bromobenzoylbenzene (0.2 mmol, 52.2 mg, 2.0 equiv.) and cesium 2-((1-methylcyclohexyl)oxy)-2-oxoacetate (0.3 mmol, 95.4 mg, 3 equiv.), 4,4'-dimethoxybenzophenone (0.01 mmol, 2.42 mg, 10 mol%.) ,the vial was capped. DMSO (2 mL) was added via a syringe, nitrogen bubbling for fifteen minutes, followed by the addition of prop-2-yn-1-yl benzoate (14.5 uL, 0.1 mmol, 1.0 equiv.). The reaction mixture was then irradiated with a 90 W blue LED lamp (at approximately 1.5 cm away from the light source) with cooling from a fan for 18 h. The reaction was quenched with $H_2O$, extracted with ethyl acetate. The combined organic layers were dried with $MgSO_4$, filtered, and concentrated in vacuo. The product was isolated by flash chromatography (PE: EA= 20:1) as a pale yellow oil (30.66 mg, 70%, *dr* = 1:1.2). Separate one of the single structures， the nuclear magnetic data of one of the configurations is as follows. $^1$H NMR (600 MHz, $CDCl_3$) δ 8.08 (d, *J* = 7.5 Hz, 2H), 7.82 (d, *J* = 7.4 Hz, 2H), 7.77 (d, *J* = 8.1 Hz, 2H), 7.60 (tt, *J* = 14.6, 7.4 Hz, 2H), 7.48 (dt, *J* = 23.0, 7.7 Hz, 4H), 7.34 (d, *J* = 8.1 Hz, 2H), 5.88 (d, *J* = 15.7 Hz, 1H), 5.66 (dt, *J* = 15.7, 6.4 Hz, 1H), 4.82 (d, *J* = 6.4 Hz, 2H), 2.89 (ddd, *J* = 12.5, 7.9, 3.2 Hz, 1H), 1.96 (d, *J* = 12.6 Hz, 1H), 1.85 – 1.78 (m, 1H), 1.74 – 1.65 (m, 2H), 1.65 – 1.56 (m, 2H), 1.42 (tdd, *J* = 12.8, 9.2, 3.8 Hz, 2H), 1.20 (s, 3H). $^{13}$C NMR (150 MHz, $CDCl_3$) δ 196.48, 166.45, 152.33, 147.88, 137.91, 135.41, 132.90, 132.20, 130.44, 130.38, 129.99, 129.63, 128.34, 128.22, 126.90, 119.12, 66.11, 44.54, 39.67, 36.41, 36.27, 33.56, 22.33, 22.06.

**HRMS (ESI+):** calcd. for $C_{30}H_{31}O_3^+$ $[M+H]^+$ 439.2273 , found 439.2268

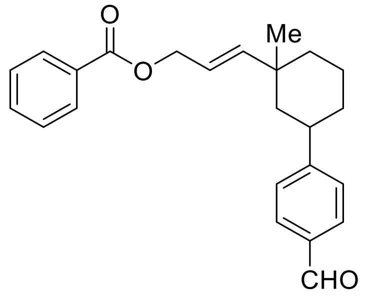

**(*E*)-3-(3-(4-formylphenyl)-1-methylcyclohexyl)allyl benzoate(33)**

To a flame-dried 8 mL reaction vial was charged with $NiCl_2(Py)_4$ (0.02 mmol, 8.9 mg , 20 mol%), dtbbpy (0.02 mmol, 5.4 mg, 20 mol%), $Ir[dF(CF_3)ppy]_2(dtbbpy)PF_6$ (0.002 mmol, 2.2 mg, 2 mol%), 4-bromobenzaldehyde(0.2 mmol, 37 mg, 2.0 equiv.) and cesium 2-((1-methylcyclohexyl)oxy)-2-oxoacetate (0.3 mmol, 95.4 mg, 3 equiv.), 4,4'-dimethoxybenzophenone (0.01 mmol, 2.42 mg, 10 mol%.) ,the vial was capped. DMSO (2 mL) was added via a syringe, nitrogen bubbling for fifteen minutes, followed by the addition of prop-2-yn-1-yl benzoate (14.5 uL, 0.1 mmol, 1.0 equiv.). The reaction mixture was then irradiated with a 90 W blue LED lamp (at approximately 1.5 cm away from the light source) with cooling from a fan for 18 h. The reaction was quenched with $H_2O$, extracted with ethyl acetate. The combined organic layers were dried with $MgSO_4$, filtered, and concentrated in vacuo. The product was isolated by flash chromatography (PE: EA= 20:1) as a pale yellow oil (19.9 mg, 55%, *dr* = 1:1.5). $^1$H NMR (600 MHz, $CDCl_3$) δ 9.97 (s, 1H), 8.08 (dd, *J* = 8.2, 1.2 Hz, 0.8H), 8.05 (dd, *J* = 8.2, 1.1 Hz, 0.2H), 7.80 (dd, *J* = 8.2, 3.3 Hz, 2H), 7.60 – 7.53 (m, 1H), 7.45 (dt, *J* = 15.5, 7.8 Hz, 2H), 7.36 (dd, *J* = 14.6, 8.1 Hz, 2H), 5.87 (dd, *J* = 26.4, 15.9 Hz, 1H), 5.73 (dt, *J* = 15.6, 6.2 Hz, 0.4H), 5.63 (dt, *J* = 15.7, 6.4 Hz, 0.6H), 4.86 (d, *J* = 6.3 Hz, 0.8H), 4.79 (d, *J* = 6.4 Hz, 1.2H), 2.86 (tt, *J* = 12.5, 3.3 Hz, 0.6H), 2.78 (tt, *J* = 12.0, 2.9 Hz, 0.4H), 1.92 – 1.63 (m, 4H), 1.60 – 1.36 (m, 4H), 1.17 (s, 1.8H), 1.06 (s, 1.2H).$^{13}$C NMR (150 MHz, $CDCl_3$) δ 192.01, 191.99, 166.42, 154.72, 154.57, 147.72, 143.44, 134.68, 134.62, 132.94, 132.89, 130.41, 130.38, 130.00, 129.62, 128.38,

128.33, 127.65, 127.56, 122.40, 119.21, 76.82, 66.13, 66.05, 45.68, 44.41, 40.48, 39.88, 37.24, 37.10, 36.37, 36.24, 33.48, 31.75, 22.69, 22.30, 22.01.

**HRMS (ESI+):** calcd. for $C_{24}H_{26}O_3^+$[M+H] $^+$363.1960 , found 363.1955

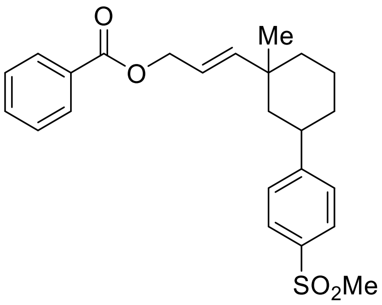


**(*E*)-3-(1-methyl-3-(4-(methylsulfonyl)phenyl)cyclohexyl)allyl benzoate (34)**

To a flame-dried 8 mL reaction vial was charged with $NiCl_2(Py)_4$ (0.02 mmol, 8.9 mg, 20 mol%), dtbbpy (0.02 mmol, 5.4 mg, 20 mol%), Ir[dF($CF_3$)ppy]$_2$(dtbbpy)$PF_6$ (0.002 mmol, 2.2 mg, 2 mol%), 4-bromobenzyl sulfone(0.2 mmol, 47 mg, 2.0 equiv.) and cesium 2-((1-methylcyclohexyl)oxy)-2-oxoacetate (0.3 mmol, 95.4 mg, 3 equiv.), 4,4'-dimethoxybenzophenone (0.01 mmol, 2.42 mg, 10 mol%.) ,the vial was capped. DMSO (2 mL) was added via a syringe, nitrogen bubbling for fifteen minutes, followed by the addition of prop-2-yn-1-yl benzoate (14.5 uL, 0.1 mmol, 1.0 equiv.). The reaction mixture was then irradiated with a 90 W blue LED lamp (at approximately 1.5 cm away from the light source) with cooling from a fan for 18 h. The reaction was quenched with $H_2O$, extracted with ethyl acetate. The combined organic layers were dried with $MgSO_4$, filtered, and concentrated in vacuo. The product was isolated by flash chromatography (PE: EA= 20:1) as a pale yellow oil (24.7 mg, 60%, *dr* = 1:1.5). $^1$H NMR (600 MHz, $CDCl_3$) δ 8.13 – 8.03 (m, 2H), 7.90 – 7.82 (m, 2H), 7.63 – 7.55 (m, 1H), 7.50 – 7.38 (m, 4H), 5.88 (dd, *J* = 23.9, 15.9 Hz, 1H), 5.75 (dt, *J* = 16.0, 6.3 Hz, 0.6H), 5.65 (dt, *J* = 15.7, 6.4 Hz, 0.4H), 4.88 (d, *J* = 6.3 Hz, 1.2H), 4.81 (dd, *J* = 6.4, 0.9 Hz, 0.8H), 3.06 (s, 3H), 2.92 – 2.86 (m, 0.4H), 2.83-1.98 (m, 0.6H), 1.94 – 1.66 (m, 4H), 1.60 – 1.32 (m, 4H), 1.19 (s, 1.2H), 1.08 (s, 1.8H). $^{13}$C NMR (150 MHz, $CDCl_3$) δ 166.44, 153.86, 153.73, 147.58, 143.31, 138.09, 132.98, 132.92, 130.40, 130.37, 129.62, 128.40, 128.35, 127.97, 127.87, 127.54, 127.52, 122.51, 119.32, 66.09, 66.01,

45.71, 44.61, 44.49, 40.27, 39.67, 37.17, 37.10, 36.36, 36.19, 33.53, 33.48, 31.73, 22.64, 22.30, 21.96.

**HRMS (ESI+)**: calcd. for $C_{24}H_{28}O_4SNa^+$ $[M+Na]^+$ 435.1606, found 435.1601.

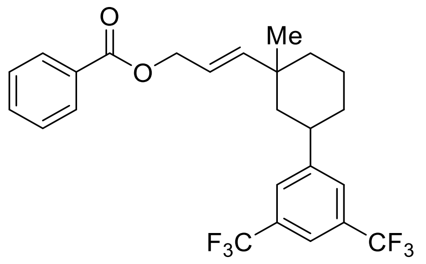

**(*E*)-3-(3-(3,5-bis(trifluoromethyl)phenyl)-1-methylcyclohexyl)allyl benzoate(35)**

To a flame-dried 8 mL reaction vial was charged with $NiCl_2(Py)_4$ (0.02 mmol, 8.9 mg, 20 mol%), dtbbpy (0.02 mmol, 5.4 mg, 20 mol%), $Ir[dF(CF_3)ppy]_2(dtbbpy)PF_6$ (0.002 mmol, 2.2 mg, 2 mol%), 1-bromo-3,5-bis(trifluoromethyl)benzene (58.2 mg, 0.2 mmol, 2.0 equiv.) and cesium 2-((1-methylcyclohexyl)oxy)-2-oxoacetate (0.3 mmol, 95.4 mg, 3 equiv.), 4,4'-dimethoxybenzophenone (0.01 mmol, 2.42 mg, 10 mol%.) ,the vial was capped. DMSO (2 mL) was added via a syringe, nitrogen bubbling for fifteen minutes, followed by the addition of prop-2-yn-1-yl benzoate (14.5 uL, 0.1 mmol, 1.0 equiv.). The reaction mixture was then irradiated with a 90 W blue LED lamp (at approximately 1.5 cm away from the light source) with cooling from a fan for 18 h. The Aeaction was quenched with $H_2O$, extracted with ethyl acetate. The combined organic layers were dried with $MgSO_4$, filtered, and concentrated in vacuo. The product was isolated by flash chromatography (PE: EA= 20:1) as a pale yellow oil (24.9 mg, 53%, *dr* = 1:1.2). Separate one of the single structures，the nuclear magnetic data of one of the configurations is as follows. $^1H$ NMR (600 MHz, $CDCl_3$) δ 8.06 (d, *J* = 7.9 Hz, 2H), 7.71 (s, 1H), 7.64 (d, *J* = 15.6 Hz, 2H), 7.55 (t, *J* = 7.4 Hz, 1H), 7.44 (t, *J* = 7.7 Hz, 2H), 5.85 (d, *J* = 15.7 Hz, 1H), 5.64 (dt, *J* = 15.7, 6.3 Hz, 1H), 4.83 (dd, *J* = 42.3, 6.3 Hz, 2H), 3.02 – 2.84 (m, 1H), 1.95 – 1.63 (m, 4H), 1.53 – 1.37 (m, 3H), 1.26 (dd, *J* = 8.1, 5.9 Hz, 1H), 1.21 (s, 3H). $^{19}F$ NMR (377 MHz, $CDCl_3$) δ -65.26(s, 6F). $^{13}C$ NMR (150 MHz, $CDCl_3$) δ 163.89, 147.01, 144.77, 130.38, 128.99(q, *J* = 33.2 Hz),127.79, 127.09,

125.80, 124.67, 120.94 (d, $J$ = 272.1 Hz), 117.56, 116.91, 63.44, 41.96, 36.93, 33.87, 33.49, 30.96, 19.70, 19.34.

**HRMS (ESI+)**: calcd. for $C_{24}H_{24}O_2F_6Na^+$ $[M+Na]^+$493.1578, found 493.1573.

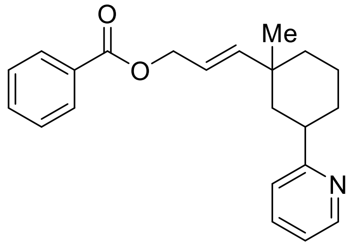

**(*E*)-3-(1-methyl-3-(pyridin-2-yl)cyclohexyl)allyl benzoate(36).** To a flame-dried 8 mL reaction vial was charged with $NiCl_2(Py)_4$ (0.02 mmol, 8.9 mg, 20 mol%), dtbbpy (0.02 mmol, 5.4 mg, 20 mol%), $Ir[dF(CF_3)ppy]_2(dtbbpy)PF_6$ (0.002 mmol, 2.2 mg, 2 mol%), 2-Bromopyridine (31.6 mg, 0.2 mmol, 2.0 equiv.) and cesium 2-((1-methylcyclohexyl)oxy)-2-oxoacetate (0.3 mmol, 95.4 mg, 3 equiv.), the vial was capped. DMSO/EA=4:1 (2.5 mL) was added via a syringe, nitrogen bubbling for fifteen minutes, followed by the addition of prop-2-yn-1-yl benzoate (14.5 uL, 0.1 mmol, 1.0 equiv.). The reaction mixture was then irradiated with a 90 W blue LED lamp (at approximately 1.5 cm away from the light source) for 18 h. The reaction was quenched with $H_2O$, extracted with ethyl acetate. The combined organic layers were dried with $MgSO_4$, filtered, and concentrated in vacuo. The product was isolated by flash chromatography (PE: EA= 20:1) as a pale yellow oil (31 mg, 77%, *dr* = 1:1.2). $^1H$ NMR (600 MHz, $CDCl_3$) δ 8.61 – 8.50 (s, 1H), 8.14 – 8.01 (m, 2H), 7.61 – 7.53 (m, 2H), 7.44 (dd, $J$ = 16.0, 8.2 Hz, 2H), 7.16 – 7.09 (m, 2H), 5.87 (dt, $J$ = 29.0, 14.6 Hz, 1H), 5.73 (dt, $J$ = 15.9, 6.4 Hz, 0.46H), 5.63 (dt, $J$ = 15.7, 6.4 Hz, 0.54H), 4.87 – 4.81 (m, 0.9H), 4.77 (dt, $J$ = 7.5, 3.7 Hz, 1.1H), 2.97 (tt, $J$ = 12.5, 3.4 Hz, 0.54H), 2.88 (tt, $J$ = 12.5, 3.4 Hz, 0.46H), 1.97 – 1.71 (m, 3H), 1.67 – 1.55 (m, 2H), 1.55 – 1.35 (m, 3H), 1.17 (s, 1.66H), 1.06 (s, 1.34H). $^{13}C$ NMR (150 MHz, $CDCl_3$) δ 166.46, 166.44, 165.95, 165.89, 149.07, 148.03, 143.71, 136.45, 132.85, 132.83, 130.46, 130.42, 129.64, 129.63, 128.33, 128.30, 122.11, 121.37, 121.23, 121.22, 121.16, 118.97, 66.28, 66.15, 44.16, 43.06, 42.19, 41.64, 37.20, 36.93, 36.33, 36.26, 32.60, 32.51, 31.72, 22.56, 22.27, 21.90.

**HRMS (ESI+)**: calcd. for $C_{22}H_{26}O_2N^+$ [M+H]$^+$ 336.1956 , found 336.1958.

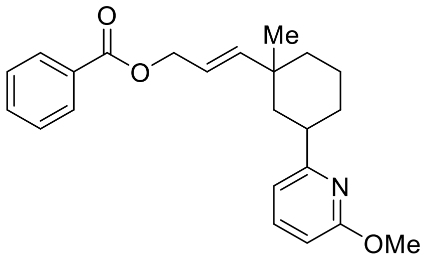

**(*E*)-3-(3-(6-methoxypyridin-2-yl)-1-methylcyclohexyl)allyl benzoate (37)**

To a flame-dried 8 mL reaction vial was charged with $NiCl_2(Py)_4$ (0.02 mmol, 8.9 mg, 20 mol%), dtbbpy (0.02 mmol, 5.4 mg, 20 mol%), Ir[dF($CF_3$)ppy]$_2$(dtbbpy)$PF_6$ (0.002 mmol, 2.2 mg, 2 mol%), 2-bromo-6-(methyloxy)pyridine (37.6 mg, 0.2 mmol, 2.0 equiv.) and cesium 2-((1-methylcyclohexyl)oxy)-2-oxoacetate (0.3 mmol, 95.4 mg, 3 equiv.),) ,the vial was capped. DMSO/EA=4:1 (2.5 mL) was added via a syringe, nitrogen bubbling for fifteen minutes, followed by the addition of prop-2-yn-1-yl benzoate (14.5 uL, 0.1 mmol, 1.0 equiv.). The reaction mixture was then irradiated with a 90 W blue LED lamp (at approximately 1.5 cm away from the light source) for 18h. The reaction was quenched with $H_2O$, extracted with ethyl acetate. The combined organic layers were dried with $MgSO_4$, filtered, and concentrated in vacuo. The product was isolated by flash chromatography (PE: EA= 20:1) as a pale yellow oil (18.2mg, 50%, *dr* = 1:1). Separation causes the dr ratio (*dr* = 1:1) to become *dr* = 3:7. The nuclear magnetic data of configurations is as follows. $^1$H NMR (600 MHz, $CDCl_3$) δ 8.10 – 8.03 (m, 2H), 7.56 (dd, $J$ = 14.1, 7.1 Hz, 1H), 7.49 – 7.40 (m, 3H), 6.69 (dd, $J$ = 12.1, 7.3 Hz, 1H), 6.53 (d, $J$ = 8.2 Hz, 1H), 5.88 (dd, $J$ = 23.3, 15.9 Hz, 1H), 5.73 (dt, $J$ = 15.9, 6.4 Hz, 0.3H), 5.63 (dt, $J$ = 15.7, 6.4 Hz, 0.7H), 4.85 (d, $J$ = 6.3 Hz, 0.6H), 4.79 (d, $J$ = 6.3 Hz, 1.4H), 3.91 (d, $J$ = 2.8 Hz, 3H), 2.84 (tt, $J$ = 12.0, 3.2 Hz, 0.7H), 2.77 (tt, $J$ = 12.3, 3.1 Hz, 0.3H), 1.92 (d, $J$ = 13.7 Hz, 1H), 1.79 – 1.62 (m, 3H), 1.59 – 1.32 (m, 4H), 1.16 (s, 2.1H), 1.05 (s, 0.9H). $^{13}$C NMR (150 MHz, $CDCl_3$) δ 166.48, 164.03, 163.95, 163.53, 148.35, 143.92, 138.77, 138.75, 132.87, 132.85, 130.47, 130.43, 129.63, 128.34, 128.32, 122.01, 118.83, 113.52, 113.47, 107.28, 107.18, 66.33, 66.23, 53.19,

53.15, 44.01, 42.89, 41.70, 41.14, 37.48, 36.91, 36.38, 36.24, 32.22, 32.20, 31.80, 22.59, 22.33, 21.89.

**HRMS (ESI+)**: calcd. for $C_{23}H_{27}NO_3^+$ [M+H]$^+$ 366.2061, found 366.2064.

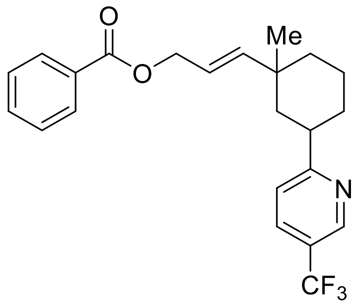


**(*E*)-3-(1-methyl-3-(5-(trifluoromethyl)pyridin-2-yl)cyclohexyl)allyl benzoate (38)**

To a flame-dried 8 mL reaction vial was charged with $NiCl_2(Py)_4$ (0.02 mmol, 8.9 mg, 20 mol%), dtbbpy (0.02 mmol, 5.4 mg, 20 mol%), Ir[dF($CF_3$)ppy]$_2$(dtbbpy)$PF_6$ (0.002 mmol, 2.2 mg, 2 mol%), 2-Bromo-5-(trifluoromethyl)pyridine (45.2 mg, 0.2 mmol, 2.0 equiv.) and cesium 2-((1-methylcyclohexyl)oxy)-2-oxoacetate (0.3 mmol, 95.4 mg, 3 equiv.), the vial was capped. DMSO/EA=4:1 (2.5 mL) was added via a syringe, nitrogen bubbling for fifteen minutes, followed by the addition of prop-2-yn-1-yl benzoate (14.5 uL, 0.1 mmol, 1.0 equiv.). The reaction mixture was then irradiated with a 90 W blue LED lamp (at approximately 1.5 cm away from the light source) for 18h. The reaction was quenched with $H_2O$, extracted with ethyl acetate. The combined organic layers were dried with $MgSO_4$, filtered, and concentrated in vacuo. The product was isolated by flash chromatography (PE: EA= 20:1) as a pale yellow oil (22.5 mg, 56%, *dr* = 1:1).H NMR (600 MHz, $CDCl_3$) δ 8.81 (s, 1H), 8.08 (dd, *J* = 9.3, 4.2 Hz, 2H), 7.92 – 7.80 (m, 1H), 7.58 (q, *J* = 7.6 Hz, 1H), 7.46 (dd, *J* = 12.2, 4.3 Hz, 2H), 7.32 – 7.29 (m, 1H), 5.89 (dt, *J* = 25.5, 12.9 Hz, 1H), 5.76 (dt, *J* = 15.9, 6.4 Hz, 0.5H), 5.68 – 5.64 (m, 0.5H), 4.90 – 4.84 (m, 1H), 4.80 (d, *J* = 6.4 Hz, 1H), 3.07 (tt, *J* = 12.5, 3.5 Hz, 0.5H), 2.98 (ddd, *J* = 12.4, 7.8, 3.3 Hz, 0.5H), 2.00 – 1.69 (m, 4H), 1.60 – 1.34 (m, 4H), 1.20 (s, 1.5H), 1.09 (d, *J* = 4.6 Hz, 1.5H). $^{13}C$ NMR (150 MHz, $CDCl_3$) δ 169.95, 169.90, 166.47, 166.44, 147.65, 146.09 (q, *J* = 4.0 Hz).143.37, 133.56, 133.53, 133.51, 133.48, 132.92, 132.89, 130.37 (d, *J* = 3.8 Hz), 129.64, 129.63, 128.37, 128.33, 122.36,

121.15, 120.13 (d, $J$ = 272.9 Hz), 66.19, 66.07, 43.86, 42.80, 42.34, 41.81, 37.06, 36.89, 36.19, 32.36, 32.27, 31.70, 22.71, 22.42, 22.24, 21.74.

**HRMS (ESI+):** calcd. for $C_{23}H_{25}F_3O_2N^+$ $[M+H]^+$ 404.1837, found 404.1832.

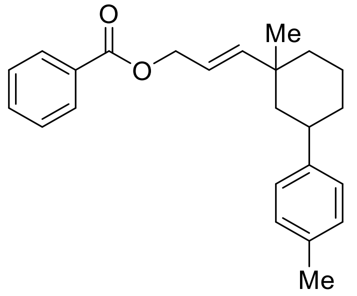


**(*E*)-3-(1-methyl-3-(p-tolyl)cyclohexyl)allyl benzoate (39)**

To a flame-dried 8 mL reaction vial was charged with $NiCl_2(Py)_4$ (0.02 mmol, 8.9 mg, 20 mol%), dtbbpy (0.02 mmol, 5.4 mg, 20 mol%), $Ir[dF(CF_3)ppy]_2(dtbbpy)PF_6$ (0.002 mmol, 2.2 mg, 2 mol%), 1-iodo-4-methylbenzene (43.6 mg, 0.2 mmol, 2.0 equiv.) and cesium 2-((1-methylcyclohexyl)oxy)-2-oxoacetate (0.3 mmol, 95.4 mg, 3 equiv.), the vial was capped. DMSO/EA=4:1 (2.5 mL) was added via a syringe, nitrogen bubbling for fifteen minutes, followed by the addition of prop-2-yn-1-yl benzoate (14.5 uL, 0.1 mmol, 1.0 equiv.). The reaction mixture was then irradiated with a 90 W blue LED lamp (at approximately 1.5 cm away from the light source) for 18h. The reaction was quenched with $H_2O$, extracted with ethyl acetate. The combined organic layers were dried with $MgSO_4$, filtered, and concentrated in vacuo. The product was isolated by flash chromatography (PE: EA= 20:1) as a pale yellow oil (20.88 mg, 60%, *dr* = 1:1.1). $^1$H NMR (600 MHz, $CDCl_3$) δ 8.15 – 8.03 (m, 2H), 7.61 – 7.53 (m, 1H), 7.49 – 7.41 (m, 2H), 7.16 – 7.05 (m, 4H), 5.95 – 5.81 (m, 1H), 5.72 (dt, $J$ = 15.9, 6.4 Hz, 0.47H), 5.63 (dt, $J$ = 15.7, 6.4 Hz,0.53H), 4.86 (d, $J$ = 6.4 Hz, 0.95H), 4.79 (d, $J$ = 6.4 Hz, 1.05H), 2.74 (tt, $J$ = 12.5, 3.4 Hz, 0.53H), 2.67 (tt, $J$ = 12.5, 3.2 Hz, 0.47H), 2.32 (s, 3H), 1.94 – 1.62 (m, 4H), 1.58 – 1.48 (m, 1H), 1.45 – 1.29 (m, 3H), 1.16 (s, 1.56H), 1.04 (d, $J$ = 9.1 Hz, 1.44H).$^{13}$C NMR (150 MHz, $CDCl_3$) δ 148.29, 144.48, 144.35, 143.93, 135.45, 135.38, 132.91, 132.87, 130.47, 130.44, 129.66, 129.06, 129.05,

128.38, 128.34, 126.79, 126.70, 122.05, 118.85, 66.30, 66.21, 44.97, 39.62, 39.01, 37.45, 37.18, 36.47, 36.38, 33.98, 33.95, 31.83, 22.91, 22.37, 22.23, 21.01.

**HRMS (ESI+):** calcd. for $C_{24}H_{28}O_2\,Na^+$ $[M+Na]^+$ 371.1986, found 371.1982.

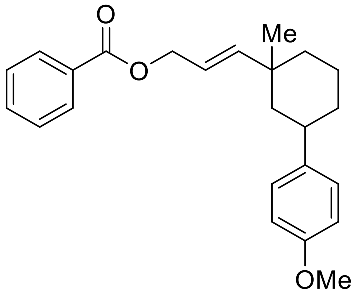


**(*E*)-3-(3-(4-methoxyphenyl)-1-methylcyclohexyl)allyl benzoate (40)**

To a flame-dried 8 mL reaction vial was charged with $NiCl_2(Py)_4$ (0.02 mmol, 8.9 mg, 20 mol%), dtbbpy (0.02 mmol, 5.4 mg, 20 mol%), $Ir[dF(CF_3)ppy]_2(dtbbpy)PF_6$ (0.002 mmol, 2.2 mg, 2 mol%), 1-iodo-4-methoxybenzene (46.6 mg, 0.2 mmol, 2.0 equiv.) and cesium 2-((1-methylcyclohexyl)oxy)-2-oxoacetate (0.3 mmol, 95.4 mg, 3 equiv.), the vial was capped. DMSO/EA=4:1 (2.5 mL) was added via a syringe, nitrogen bubbling for fifteen minutes, followed by the addition of prop-2-yn-1-yl benzoate (14.5 uL, 0.1 mmol, 1.0 equiv.). The reaction mixture was then irradiated with a 90 W blue LED lamp (at approximately 1.5 cm away from the light source) for 18 h. The reaction was quenched with $H_2O$, extracted with ethyl acetate. The combined organic layers were dried with $MgSO_4$, filtered, and concentrated in vacuo. The product was isolated by flash chromatography (PE: EA= 20:1) as a pale yellow oil (18.9 mg, 52%, *dr* = 1:1). $^1$H NMR (600 MHz, $CDCl_3$) δ 8.09 (dd, *J* = 14.4, 7.5 Hz, 2H), 7.58 (dd, *J* = 16.3, 7.5 Hz, 1H), 7.47 (dt, *J* = 11.4, 7.8 Hz, 2H), 7.18 – 7.12 (m, 2H), 6.86 (d, *J* = 8.5 Hz, 2H), 5.89 (dd, *J* = 27.5, 15.8 Hz, 1H), 5.73 (dt, *J* = 15.7, 6.3 Hz, 0.5H), 5.64 (dt, *J* = 15.7, 6.4 Hz, 0.5H), 4.87 (d, *J* = 6.4 Hz, 1H), 4.80 (d, *J* = 6.4 Hz, 1H), 3.81 (s, 3H), 2.76-2.72 (m, 0.5H), 2.69 – 2.63 (m, 0.5H), 1.90 – 1.63 (m, 4H), 1.56 – 1.40 (m, 2H), 14-1.32 (m, 2H), 1.17 (s, 1.5H), 1.06 (s, 1.5H).$^{13}$C NMR (150 MHz, $CDCl_3$) δ 166.42, 157.72, 148.27, 143.91, 139.67, 139.52, 132.89, 132.85, 130.41, 129.63, 128.36, 128.32, 127.73, 127.64, 122.01, 118.82, 113.72, 113.71, 66.29, 66.20, 55.28, 46.39,

45.09, 39.15, 38.54, 37.41, 37.18, 36.47, 36.35, 34.13, 34.10, 31.81, 22.89, 22.35, 22.22, 0.02.

**HRMS (ESI+):** calcd. for $C_{24}H_{28}O_3Na^+$ $[M+Na]^+$387.1936, found 387.1931.

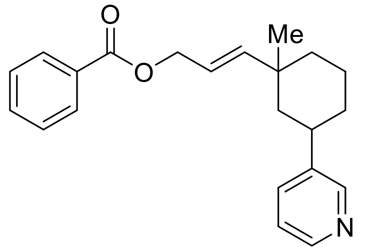


**(*E*)-3-(1-methyl-3-(pyridin-3-yl)cyclohexyl)allyl benzoate (41)**

To a flame-dried 8 mL reaction vial was charged with $NiCl_2(Py)_4$ (0.02 mmol, 8.9 mg, 20 mol%), dtbbpy (0.02 mmol, 5.4 mg, 20 mol%), Ir[dF($CF_3$)ppy]$_2$(dtbbpy)$PF_6$ (0.002 mmol, 2.2 mg, 2 mol%), 3-iodopyridine (41 mg, 0.2 mmol, 2.0 equiv.) and cesium 2-((1-methylcyclohexyl)oxy)-2-oxoacetate (0.3 mmol, 95.4 mg, 3 equiv.), the vial was capped. DMSO/EA=4:1 (2.5 mL) was added via a syringe, nitrogen bubbling for fifteen minutes, followed by the addition of prop-2-yn-1-yl benzoate (14.5 uL, 0.1 mmol, 1.0 equiv.). The reaction mixture was then irradiated with a 90 W blue LED lamp (at approximately 1.5 cm away from the light source) for 18h. The reaction was quenched with $H_2O$, extracted with ethyl acetate. The combined organic layers were dried with $MgSO_4$, filtered, and concentrated in vacuo. The product was isolated by flash chromatography (PE: EA= 20:1) as a pale yellow oil (20.4mg, 61%, *dr* = 1:1.2). $^1$H NMR (600 MHz, $CDCl_3$) δ 8.50 (dd, *J* = 24.6, 8.7 Hz, 2H), 8.13 – 8.06 (m, 2H), 7.63 – 7.55 (m, 2H), 7.47 (dt, *J* = 12.4, 7.8 Hz, 2H), 7.29 – 7.22 (m, 1H), 5.88 (dd, *J* = 23.6, 15.9 Hz, 1H), 5.75 (dt, *J* = 16.0, 6.3 Hz, 0.45H), 5.65 (dt, *J* = 15.7, 6.4 Hz, 0.55H), 4.93 – 4.85 (m, 0.86H), 4.81 (dd, *J* = 6.4, 0.9 Hz, 1.14H), 2.84 (tt, *J* = 12.6, 3.4 Hz, 0.54H), 2.75 (tt, *J* = 12.5, 3.2 Hz, 0.46H), 2.00 – 1.56 (m, 5H), 1.53 – 1.29 (m, 3H), 1.19 (s, 1.65H), 1.08 (s, 1.35H). $^{13}$C NMR (150 MHz, $CDCl_3$) δ 166.45, 166.44, 148.58, 148.44, 147.66, 147.04, 146.97, 143.29, 142.61, 142.50, 134.68, 132.94, 132.90, 132.26, 130.37, 130.35, 129.63, 128.39, 128.33, 123.56, 123.54, 122.44, 119.22, 66.13, 66.06,

45.61, 44.41, 37.71, 37.14, 37.11, 37.08, 36.37, 36.15, 33.60, 33.57, 31.74, 22.67, 22.25, 21.98.

**HRMS (ESI+):** calcd. for $C_{22}H_{26}O_2N^+$ [M+H] $^+$336.1964 , found 336.1958.

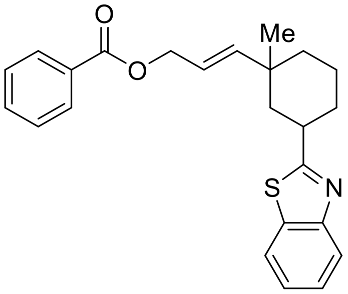


**(*E*)-3-(3-(benzo[d]thiazol-2-yl)-1-methylcyclohexyl)allyl benzoate(42)**

To a flame-dried 8 mL reaction vial was charged with $NiCl_2(Py)_4$ (0.02 mmol, 8.9 mg, 20 mol%), dtbbpy (0.02 mmol,5.4 mg, 20 mol%), $Ir[dF(CF_3)ppy]_2(dtbbpy)PF_6$ (0.002 mmol, 2.2 mg, 2 mol%), 2-chlorobenzo[d]thiazolee (33.9 mg, 0.2 mmol, 2.0 equiv.) and cesium 2-((1-methylcyclohexyl)oxy)-2-oxoacetate (0.3 mmol, 95.4 mg, 3 equiv.), 4,4'-dimethoxybenzophenone (0.01 mmol, 2.42 mg, 10 mol%.) ,the vial was capped. DMSO (2.0 mL) was added via a syringe, nitrogen bubbling for fifteen minutes, followed by the addition of prop-2-yn-1-yl benzoate (14.5 uL, 0.1 mmol, 1.0 equiv.). The reaction mixture was then irradiated with a 90 W blue LED lamp (at approximately 1.5 cm away from the light source) with cooling from a fan for 18 h. The reaction was quenched with $H_2O$, extracted with ethyl acetate. The combined organic layers were dried with $MgSO_4$, filtered, and concentrated in vacuo. The product was isolated by flash chromatography (PE: EA= 20:1) as a pale yellow oil (21.9 mg, 56%, *dr* = 1:1.2). $^1$H NMR (600 MHz, $CDCl_3$) δ 8.13 – 8.04 (m, 2H), 8.00 (dd, *J* = 8.1, 4.1 Hz, 1H), 7.87 (d, *J* = 8.0 Hz, 1H), 7.58 (dd, *J* = 13.6, 7.3 Hz, 1H), 7.50 – 7.44 (m, 3H), 7.37 (t, *J* = 7.6 Hz, 1H), 5.90 (dd, *J* = 15.7, 13.2 Hz, 1H), 5.78 (dt, *J* = 16.0, 6.3 Hz, 0.46H), 5.69 (dt, *J* = 15.7, 6.3 Hz, 0.54H), 4.90 – 4.84 (m, 0.91H), 4.82 (d, *J* = 6.3 Hz, 1.1H), 3.38-3.37 (m, 0.55H), 3.4-3.28 m, 0.45H), 2.25 – 1.82 (m, 3H), 1.75 – 1.65 (m, 2H), 1.59 – 1.56 (m, 1H), 1.49 – 1.33 (m, 2H), 1.21 (s, 1.59H), 1.12 (s, 1.41H). $^{13}$C NMR (150 MHz, $CDCl_3$) δ 177.26, 166.45, 153.04, 147.16, 142.84, 134.47, 132.93, 132.90, 130.36,

129.68, 129.66, 128.37, 128.35, 125.91, 125.89, 124.63, 124.61, 122.66, 122.58, 121.60, 119.51, 66.08, 66.00, 44.36, 43.40, 39.68, 39.16, 37.01, 36.97, 36.30, 36.11, 33.30, 33.23, 31.59, 29.73, 22.18, 21.62.

**HRMS (ESI+):** calcd. for $C_{24}H_{26}NO_2S^+$ [M+H] $^+$ 392.1684 , found 392.1679

## 4. Transformations of products

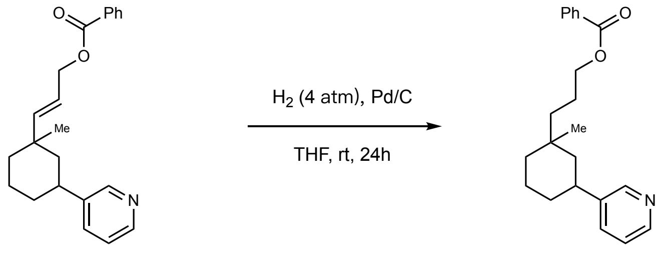


30 43

**3-(1-methyl-3-(pyridin-3-yl)cyclohexyl)propyl benzoate compound with methane (1:1) (43)**

To a stirred solution of (E)-3-(1-methyl-3-(pyridin-3-yl)cyclohexyl)allyl benzoate (33.5 mg, 0.1 mmol, 1.0 equiv, *dr* = 1:1.2) in THF (5.0 mL), Pd/C (10%, 10 mol%) was added at rt. Reaction was placed in hydrogen autoclave of 4 atmospheres for 24 hours. After the reaction is completed, the reaction mixture was poured into aq. NaCl (sat) with diethyl ether. The aqueous layer was extracted with two portions of diethyl ether. The combined extract was washed with brine, dried over MgSO4 and concentrated under reduced pressure. The residue was purified by column chromatography on silica gel with hexanes: ethyl acetate = 10: 1 to give 23.8mg colorless oil liquid, in 67% yield. Separation causes the dr ratio (*dr* = 1:1.2) to become *dr* = 1:2. The nuclear magnetic data of configurations is as follows. $^1$H NMR (600 MHz, $CDCl_3$) δ 8.51 (dd, *J* = 29.7, 6.7 Hz, 2H), 8.13 (d, *J* = 7.1 Hz, 0.5H), 8.06 (dd, *J* = 8.2, 1.2 Hz, 0.5H), 8.03 (dd, *J* = 8.2, 1.1 Hz, 1H), 7.60 – 7.54 (m, 2H), 7.44 (dt, *J* = 13.1, 5.2 Hz, 2H), 7.30 – 7.26 (m, 1H), 4.38 – 4.32 (m, 0.65H), 4.30 (q, *J* = 6.8 Hz, 1.35H), 2.83 – 2.70 (m, 1H), 1.89 (d, *J* = 12.6 Hz, 1H), 1.83 – 1.43 (m, 7H), 1.38 – 1.30 (m, 3H), 1.24 – 1.18 (m, 1H), 1.04 (s, 2H), 0.93 (s, 1H).$^{13}$C NMR (150 MHz, $CDCl_3$) δ 207.01, 166.66, 148.61, 148.54, 146.99, 146.97, 142.92, 134.76, 132.90, 132.84, 130.44, 130.01, 129.53, 128.39, 128.34, 128.24, 123.56, 65.77, 65.72, 45.45, 45.19, 42.35, 37.30, 36.93, 33.90, 33.63, 33.53, 31.96, 31.94, 30.94, 29.71, 22.80, 22.70, 22.19, 21.97, 21.88, 14.13.

**HRMS (ESI+):** calcd. for $C_{22}H_{27}O_2N^+$ [M+H]$^+$: 338.2111 , found 338.2115.

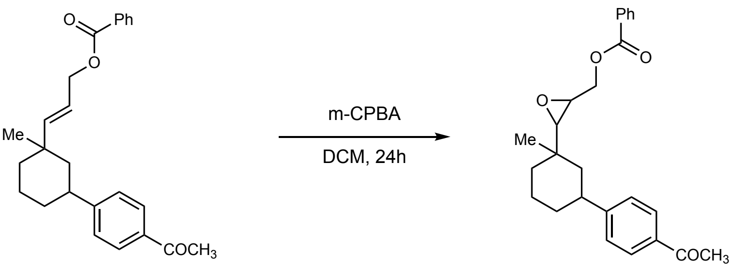


**41** **44**

**(3-(3-(4-acetylphenyl)-1-methylcyclohexyl)oxiran-2-yl)methyl benzoate (44)[4]**

To a stirred solution of olefin (Z)-3-(3-(4-acetylphenyl)-1-methylcyclohexyl)allyl benzoate (50 mg, 0.133 mmol, 1.0 equiv, *dr* = 1:1.3) in DCM (2.0 mL), m-CPBA (49.2 mg, 0.15 mmol, 1.5 equiv) was added at rt under $N_2$ atmosphere. After being stirred for 24 h, the reaction mixture was poured into aq. $NaHCO_3$ (sat) with diethyl ether. The aqueous layer was extracted with two portions of diethyl ether. The combined extract was washed with brine, dried over $MgSO_4$ and concentrated under reduced pressure. The residue was purified by column chromatography on silica gel with hexanes : ethyl acetate = 10 : 1 to give 33.8 mg white solid in 65% yield and *dr* = 1.1:1.1:0.42:0.38.$^{1}$H NMR (600 MHz, $CDCl_3$) δ 8.10 (dd, *J* = 18.1, 7.3 Hz, 2H), 7.91 (dt, *J* = 10.8, 5.4 Hz, 2H), 7.60 (dd, *J* = 11.8, 4.3 Hz, 1H), 7.48 (dt, *J* = 15.4, 7.7 Hz, 2H), 7.31 (dt, *J* = 8.4, 4.3 Hz, 2H), 4.68 – 4.58 (m, 1H), 4.33 – 4.27 (m, 0.27H), 4.23 – 4.14 (m, 0.83H), 3.35-3.30 (m, 1H), 3.15 – 2.75 (m, 2H), 2.60 (d, *J* = 2.2 Hz, 2.8H), 2.31 (d, *J* = 1.7 Hz, 0.2H), 1.96 – 1.69 (m, 3H), 1.62 – 1.31 (m, 5H), 1.11 (s, 1.1H), 1.10 (s, 1.1H), 0.93 (s, 0.42H), 0.91 (s,0.38H). $^{13}$C NMR (150 MHz, $CDCl_3$) δ 197.86, 166.32, 152.65, 152.58, 135.29, 135.25, 133.29, 133.22, 129.74, 129.70, 128.62, 128.58, 128.48, 128.43, 127.80, 127.76, 127.16, 127.12, 127.07, 127.03, 121.34, 121.32, 65.77, 65.63, 65.56, 65.30, 65.27, 62.25, 62.16, 52.54, 52.08, 51.71, 51.67, 45.10, 43.06, 41.76, 40.18, 39.81, 39.75, 39.22, 39.19, 36.46, 34.96, 34.21, 34.14, 33.68, 33.63, 33.57, 33.50, 33.08, 33.03, 31.67, 26.71, 26.61, 26.30, 22.85, 22.49, 21.57, 21.54, 21.17, 19.53, 19.38.

**HRMS (EI):** calcd. for $C_{25}H_{28}O_4^+$ $[M+Na]^+$:415.1876 , found 415.1880.

[4]Chuentragool, P., Yadagiri, D., Morita, T., Sarkar, S., Parasram, M., Wang, Y., & Gevorgyan, V. (2019). Aliphatic Radical Relay Heck Reaction at Unactivated C (sp3)– H Sites of Alcohols. *Angewandte Chemie*, **2019**,*131*, 1808.

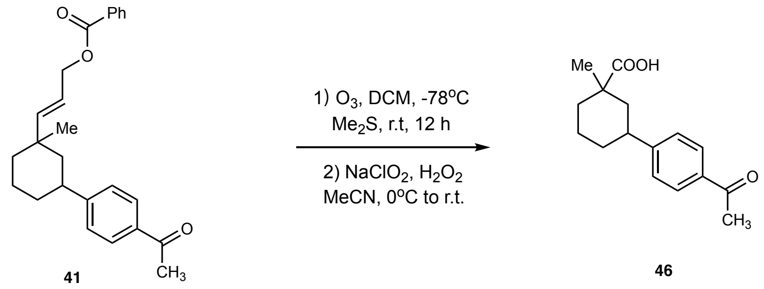


**3-(4-acetylphenyl)-1-methylcyclohexane-1-carboxylic acid (46)[5],[6]**

To a stirred solution of olefin (Z)-3-(3-(4-acetylphenyl)-1-methylcyclohexyl)allyl benzoate (75.3 mg, 0.2 mmol, 1.0 equiv, *dr* = 1:1.3). DCM was added (5 mL) in a 25 mL round bottom flask. The mixture was cooled to – 78 °C (dry ice/acetone). Ozone was bubbled through the reaction mixture until the color of the solution changes to blue. Then, $O_2$ gas was bubbled through the reaction mixture over 10min, dimethylsulfide was added (0.1mL, 10 equiv), and the reaction mixture was warmed to room temperature over 12 h. The reaction was quenched with water (5 mL), extract with $CH_2Cl_2$, and washed with brine. The combined extract was washed with brine, dried over $MgSO_4$ and concentrated under reduced pressure. The reaction mixture was dissolved in MeCN (1 mL) and cool to 0°C, and under rigorous stirring a solution of 1.6 mg $KHSO_4$ in 1ml water and 20 uL of $H_2O_2$ (30%) were added. After addition of a solution of 40 mg of $NaClO_2$ (technical, 80%) in 168 uL water ,the reaction solution until ending of gas evolution (ca.2 h).Then, 20 mg of $NaS_2O_3$ was added to destroy excess $NaClO_2$ and the solution for 1h. The aqueous layer was acidified to pH = 1 by addition of cold HCl (1 N). The combined extract were dried over $MgSO_4$ and concentrated under reduced pressure. The residue was purified by column chromatography on silica gel with hexane : ethyl acetate = 1:1 to give 1-(4-(3-(hydroxymethyl)-3-methylcyclohexyl)phenyl)ethan-1-ol (45 mg, 87%) for a white

---

[5]Chuentragool, P., Yadagiri, D., Morita, T., Sarkar, S., Parasram, M., Wang, Y., & Gevorgyan, V. Aliphatic Radical Relay Heck Reaction at Unactivated C (sp3)− H Sites of Alcohols. *Angew. Chem., Int. Ed.*, **2019**, *131*(6), 1808.

[6] Beumer, R., Bubert, C., Cabrele, C., Vielhauer, O., Pietzsch, M., & Reiser, O. The synthesis of diastereo-and enantiomerically pure β-aminocyclopropanecarboxylic acids. *J. Org. Chem.*, **2000**,*65*(26), 8960.

solid. Separate one of the single structures, the nuclear magnetic data of one of the configurations is as follows.$^1$H NMR (600 MHz, $CDCl_3$) δ 7.93 (d, $J$ = 8.2 Hz, 2H), 7.35 (d, $J$ = 8.2 Hz, 2H), 2.88 – 2.80 (m, 1H), 2.61 (s, 3H), 2.36 (dd, $J$ = 38.0, 13.5 Hz, 2H), 1.91-1.84 (m, 2H), 1.60-1.58(m, 1H), 1.43 (qd, $J$ = 12.8, 3.5 Hz, 1H), 1.32 (s, 3H), 1.27-1.21 (m, 2H). $^{13}$C NMR (150 MHz, $CDCl_3$) δ 197.98, 183.40, 152.47, 135.25, 128.61, 127.07, 44.20, 42.96, 41.31, 35.34, 32.62, 28.72, 26.58, 23.89.

**HRMS (ESI+):** calcd. for $C_{16}H_{20}O_3^+$ $[M+H]^+$ : 261.1491, found:261.1495.

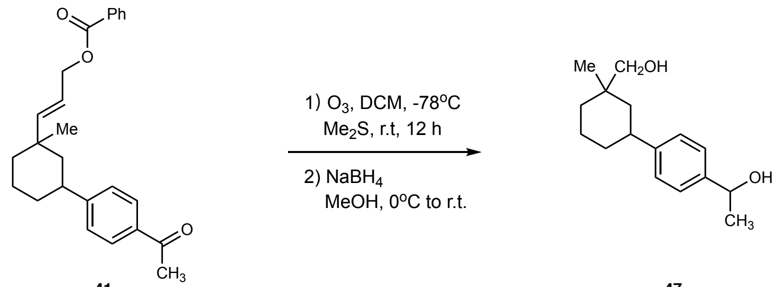


**1-(4-(3-(hydroxymethyl)-3-methylcyclohexyl)phenyl)ethan-1-ol(47)[4]**

To a stirred solution of olefin (Z)-3-(3-(4-acetylphenyl)-1-methylcyclohexyl)allyl benzoate (75.25 mg, 0.2 mmol, 1.0 equiv, *dr* = 1:1.3). DCM was added (5 mL) in a 25 mL round bottom flask. The mixture was cooled to – 78 °C (dry ice/acetone). Ozone was bubbled through the reaction mixture until the color of the solution changes to blue or purple. Then, $O_2$ gas was bubbled through the reaction mixture over 10min, dimethylsulfide was added (0.1mL, 10 equiv), and the reaction mixture was warmed to room temperature over 12h. The reaction was quenched with water (5 mL), extract with DCM, and washed with brine. The combined extract was washed with brine, dried over $MgSO_4$ and concentrated under reduced pressure. The reaction mixture was dissolved in MeOH (4 mL) and cool to 0°C, and $NaBH_4$ (75.66 mg, 2 mmol,10 equiv) was added .The reaction mixture was warmed to room temperature for overnight. The combined extract was washed with brine, dried over $MgSO_4$ and concentrated under reduced pressure. The residue was purified by column chromatography on silica gel with hexanes : ethyl acetate = 5 : 1 to give 44 mg white solid, in 80% yield. Separation causes the dr ratio (*dr* = 1:1.3) to become *dr* = 1:1. $^1$H NMR (600 MHz, $CDCl_3$) δ 7.32

(d, $J$ = 8.0 Hz, 2H), 7.22 (dd, $J$ = 8.0, 4.5 Hz, 2H), 4.90 (q, $J$ = 6.4 Hz, 1H), 3.64 (d, $J$ = 11.0 Hz, 1H), 3.35 (s, 1H), 2.77 (tt, $J$ = 12.5, 3.4 Hz, 0.5H), 2.68 (tt, $J$ = 12.6, 3.4 Hz, 0.5H), 1.92 – 1.69 (m, 4H), 1.51 (d, $J$ = 6.5 Hz, 3H), 1.45 – 1.28 (m, 5H), 1.06 (s, 1.5H), 0.98 (s, 1.5H). $^{13}$C NMR (150 MHz, $CDCl_3$) δ 146.86, 146.68, 143.42, 127.05, 126.99, 125.47, 74.78, 70.28, 67.21, 42.58, 41.90, 39.17, 38.97, 36.28, 35.69, 34.23, 34.02, 33.81, 33.14, 27.79, 25.01, 22.33, 21.96, 19.93.

**HRMS (ESI+):** calcd. for $C_{16}H_{24}O_2Na^+$ [M+Na]$^+$: 271.1666, found 276.1669.

# 5. Unsuccessful Oxalates and Mechanistic Studies

## 5.1 Unsuccessful oxalates

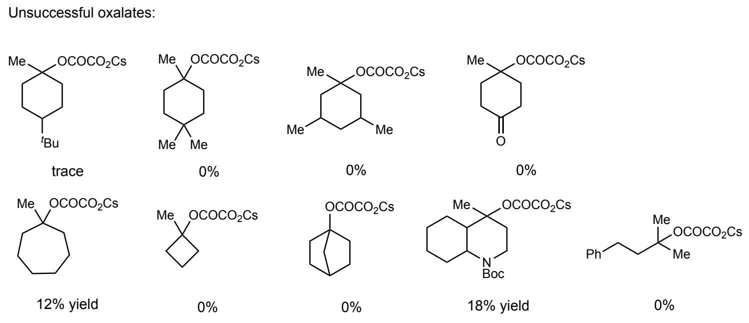


## 5.2 Preliminary mechanistic studies

### a) Radical inhibition experiment

1) Free radical trapping experiment

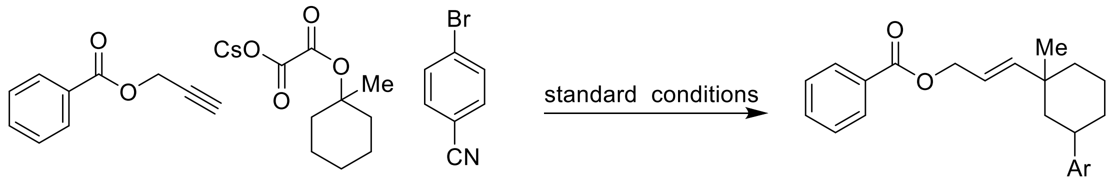


0.1 mmol 3 equiv. 2 equiv. **4**

To a flame-dried 8 mL reaction vial was charged with $NiCl_2(Py)_4$ (0.02 mmol, 8.9 mg, 20 mol%), dtbbpy (0.02 mmol, 5.4 mg, 20 mol%), $Ir[dF(CF_3)ppy]_2(dtbbpy)PF_6$ (0.002 mmol, 2.2 mg, 2 mol%), 4-Bromobenzonitrile (0.2 mmol, 36.4 mg, 2.0 equiv.) and cesium 2-((1-methylcyclohexyl)oxy)-2-oxoacetate (0.3 mmol, 95.4 mg, 3 equiv.), 4,4'-dimethoxybenzophenone (0.01 mmol, 2.42 mg, 10 mol%.) , TEMPO (0.3 mmol, 46.9 mg, 3 equiv.) the vial was capped. DMSO (2 mL) was added via a syringe, nitrogen bubbling for fifteen minutes, followed by the addition of prop-2-yn-1-yl benzoate (14.5 uL, 0.1 mmol, 1.0 equiv.). The reaction mixture was then irradiated with a 90 W blue LED lamp (at approximately 1.5 cm away from the light source) with cooling from a fan for 18 h. No product was detected from the hydrogen spectrum and TLC of the reaction solution.

2) Radical Clock Experiments

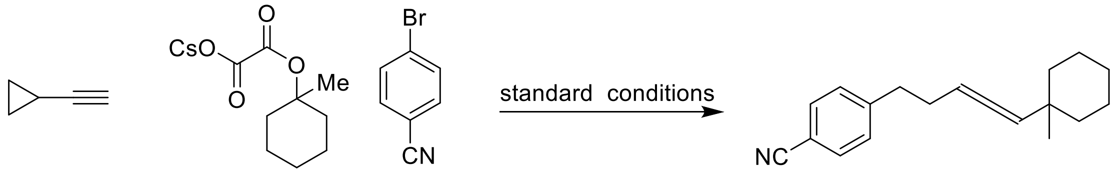


To a flame-dried 8 mL reaction vial was charged with $NiCl_2(Py)_4$ (0.02 mmol, 8.9 mg, 20 mol%), dtbbpy (0.02 mmol, 5.4 mg, 20 mol%), $Ir[dF(CF_3)ppy]_2(dtbbpy)PF_6$ (0.002 mmol, 2.2 mg, 2 mol%), 4-Bromobenzonitrile (0.2 mmol, 36.4 mg, 2.0 equiv.) and cesium 2-((1-methylcyclohexyl)oxy)-2-oxoacetate (0.3 mmol, 95.4 mg, 3 equiv.), 4,4'-dimethoxybenzophenone (0.01 mmol, 2.42 mg, 10 mol%.) .DMSO (2 mL) was added via a syringe, nitrogen bubbling for fifteen minutes, followed by the addition of ethynylcyclopropane (8.5 uL, 0.1 mmol, 1.0 equiv.). The reaction mixture was then irradiated with a 90 W blue LED lamp (at approximately 1.5 cm away from the light source) with cooling from a fan for 8 h. The reaction was quenched with $H_2O$, extracted with ethyl acetate. The combined organic layers were dried with MgSO4, filtered, and concentrated in vacuo. The crude material was purified by flash chromatography (PE) as a pale yellow oil (7.5 mg, 28%).

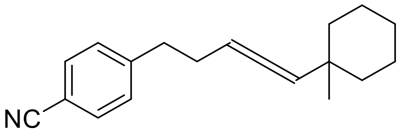


**4-(5-(1-methylcyclohexyl)penta-3,4-dien-1-yl)benzonitrile(S48):** $^1$H NMR (600 MHz, $CDCl_3$) δ 7.57 (d, *J* = 8.2 Hz, 2H), 7.29 (d, *J* = 8.1 Hz, 2H), 5.14 (q, *J* = 6.4 Hz, 1H), 5.04 (dt, *J* = 6.2, 3.0 Hz, 1H), 2.78 (ddd, *J* = 12.7, 8.5, 4.4 Hz, 2H), 2.37 – 2.27 (m, 2H), 1.44 – 1.38 (m, 6H), 1.29 (dd, *J* = 8.9, 4.8 Hz, 1H), 1.25 – 1.15 (m, 3H), 0.94 (s, 3H). $^{13}$C NMR (151 MHz, $CDCl_3$) δ 202.31, 147.62, 132.12, 129.36, 119.14, 109.71, 102.31, 91.08, 38.34, 38.14, 35.64, 34.93, 30.32, 26.18, 22.61, 22.58. **HRMS (EI+):** calcd. for C19H23N $[M+NH_4]^+$: 283.2169, found 283.2169.

**b) Competitive experiments between alkylalkyne and arylalkyne**

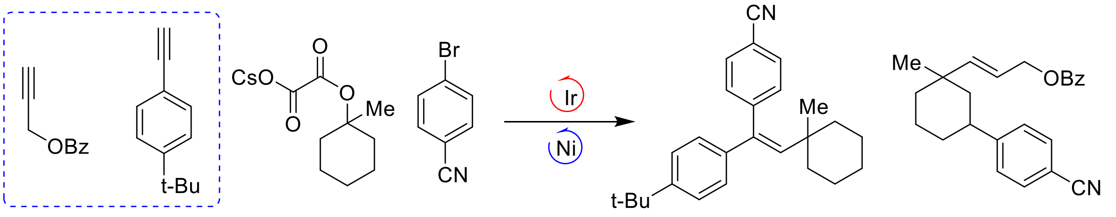


**4-(1-(4-(tert-butyl)phenyl)-2-(1-methylcyclohexyl)vinyl)benzonitrile(49)**

To a flame-dried 8 mL reaction vial was charged with $NiCl_2(Py)_4$ (0.02 mmol, 8.9 mg, 20 mol%), dtbbpy (0.02 mmol, 5.4 mg, 20 mol%), $Ir[dF(CF_3)ppy]_2(dtbbpy)PF_6$ (0.002 mmol, 2.2 mg, 2 mol%), 4-Bromobenzonitrile (0.1 mmol, 18.2 mg, 1.0 equiv.) and cesium 2-((1-methylcyclohexyl)oxy)-2-oxoacetate (0.3 mmol, 96 mg, 3 equiv.), 4,4'-dimethoxybenzophenone (0.01 mmol, 2.42 mg, 10 mol%.), the vial was capped. DMSO (2 mL) was added via a syringe, nitrogen bubbling for fifteen minutes, followed by the addition ofprop-2-yn-1-yl benzoate (29 uL, 0.2 mmol, 2.0 equiv.). 1-(tert-butyl)-4-ethynylbenzene (36.3 uL, 0.2 mmol, 2.0 equiv.). The 1.5 cm away from the light source with cooling from fan for 18 h. he combined extract was washed with brine, dried over $MgSO_4$ and concentrated under reduced pressure. The residue was purified by column chromatography on silica gel with hexanes : ethyl acetate = 15 : 1 to give 11.4 mg colorless oil liquid, in 32% yield (*Z/E* = 10:1). $^1$H NMR (600 MHz, $CDCl_3$) δ 7.67 – 7.62 (m, 1.82H), 7.50 (d, *J* = 8.6 Hz, 0.18H), 7.36 – 7.32 (m, 2H), 7.29 – 7.27 (m, 2H), 7.08 – 7.04 (m, 2H), 6.11 (s, 0.08H), 6.08 (s, 0.92H), 1.47 (d, *J* = 3.6 Hz, 1H), 1.43 – 1.38 (m, 6H), 1.35 (s, 0.8H), 1.29 (s, 8.2H), 1.26 (s, 1H), 1.11 – 1.05 (m, 2H), 0.99 (s, 0.26H), 0.96 (s, 2.74H). $^{13}$C NMR (150 MHz, $CDCl_3$) δ 150.19, 146.56, 140.18, 139.74, 138.30, 131.81, 131.67, 130.85, 129.44, 127.39, 126.38, 125.16, 124.92, 119.00, 110.74, 39.49, 39.33, 37.56, 37.22, 34.60, 34.45, 31.41, 31.28, 26.06, 26.01, 23.03, 22.87.

**HRMS (ESI+):** calcd. for $C26H31N^+$ $[M+]^+$: 357.2452, found 357.2457.

**c) Divalent nickel complex experiment**

1) Synthesis of (dtbbpy)NiII(2-tolyl)I**[50]**:[7]

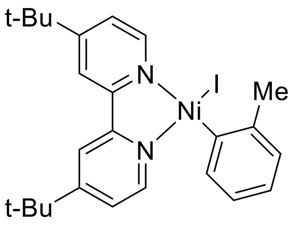


A solution of (dtbbpy)Ni(cod) was generated by stirring 138 mg of Ni(cod)$_2$ (0.5 mmol) and 134 mg of 4,4'-di-tert-butyl-2,2'-bipyridine (0.5 mmol) in 5 mL of THF overnight at room temperature in an argon-filled glovebox. Upon dissolution of the solids, the reaction mixture became dark purple. To this purple mixture was added 0.076 mL of 2-iodotoluene (131 mg, 0.6 mmol) and the color rapidly changed to red, indicating formation of 6. The solution was stirred for an additional 2 h before the solvent was removed under vacuum. The solid was triturated with dry, degassed pentane three times to remove residual cyclooctadiene and 2-iodotoluene and then dried under vacuum overnight to provide 230 mg of 6 (85% yield). $^1$H NMR (400 MHz, Acetone) δ 9.56 (d, $J$ = 5.9 Hz, 1H), 8.45 – 8.32 (m, 2H), 7.65 (dd, $J$ = 5.9, 1.7 Hz, 1H), 7.56 (dd, $J$ = 7.4, 1.1 Hz, 1H), 7.37 (dt, $J$ = 24.7, 12.4 Hz, 1H), 6.83 (d, J = 6.1 Hz, 1H), 6.75 (d, J = 7.3 Hz, 1H), 6.70 (t, J = 6.7 Hz, 1H), 6.62 (dt, J = 7.2, 3.6 Hz, 1H), 2.95 (s, 3H), 1.42 (s, 9H), 1.36 (s, 9H).

2) (dtbbpy)NiII(2-tolyl)I Catalyzed three-component reaction of alkyne

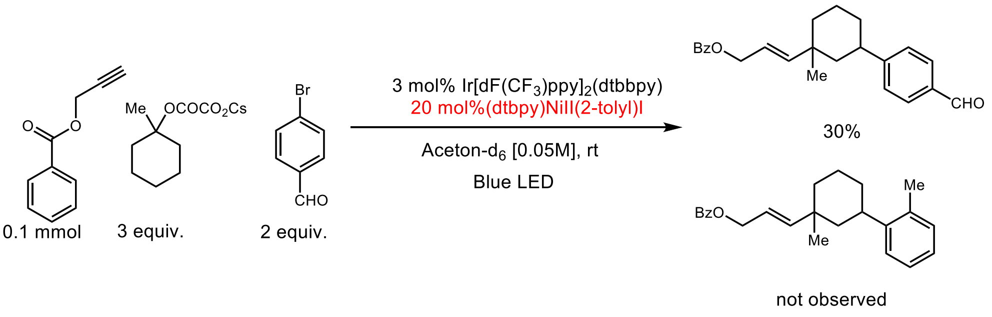


---

[7]K. M. M. Huihui, J. A. Caputo, Z. Melchor, A. M. Olivares, A. M. Spiewak, K. A. Johnson, T. A. DiBenedetto, S. Kim, L. K. G. Ackerman, D. J. Weix, *J. Am. Chem. Soc.* **2016**, *138*, 5016.

To a flame-dried 8 mL reaction vial was charged Ir[dF($CF_3$)ppy]$_2$(dtbbpy)$PF_6$ (0.003 mmol, 3.3 mg, 3 mol%), 4-Bromobenzaldehyde (0.2 mmol, 37 mg, 2.0 equiv.) and cesium 2-((1,4-dimethylcyclohexyl)oxy)-2-oxoacetate (0.3 mmol, 99.6 mg, 3 equiv.), (dtbpy)NiII(2-tolyl)I (0.02 mmol, 10.88 mg, 20 mol%), 2 mL aceton-d6 was added in an argon-filled glovebox, followed by the addition of prop-2-yn-1-yl benzoate (14.5 uL, 0.1 mmol, 1.0 equiv.). The 1.5 cm away from the light source with cooling from fan for 8 h. The combined extract was washed with brine, dried over $MgSO_4$ and concentrated under reduced pressure. The residue was purified by column chromatography on silica gel with hexanes : ethyl acetate = 20 : 1 to give 10.8 mg colorless oil liquid, in 30% yield.

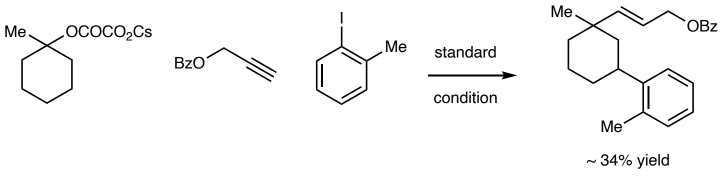


**Note:** The reaction of 2-tolyl iodide (0.2 mmol, 2.0 equiv.) with prop-2-yn-1-yl benzoate (0.1 mmol, 1.0 equiv.) and cesium 2-((1,4-dimethylcyclohexyl)oxy)-2-oxoacetate (0.3 mmol, 3 equiv.) under the standard condition afforded the desired 1,3-vinylarylation product in about 34% yield.

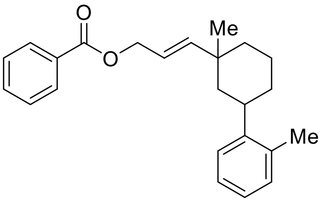


**(E)-3-(1-methyl-3-(o-tolyl)cyclohexyl)allyl benzoate [51]**

A pale yellow oil (11.8 mg, 34%, *dr* = 1:1). $^1$H NMR (400 MHz, $CDCl_3$) δ 8.06 (t, *J* = 7.7 Hz, 2H), 7.63 – 7.50 (m, 3H), 7.17 – 7.04 (m, 4H), 5.93 (d, *J* = 15.9 Hz, 0.5H), 5.84 (d, *J* = 15.8 Hz, 0.5H), 5.74 (dt, *J* = 16.0, 6.4 Hz, 0.5H), 5.61 (dt, *J* = 15.7, 6.4 Hz, 0.5H), 4.86 (t, *J* = 6.9 Hz, 1H), 4.77 (d, *J* = 6.4 Hz, 1H), 3.01 (t, *J* = 12.2 Hz, 0.5H), 2.90 (t, *J* = 12.2 Hz, 0.5H), 2.34 (s, 1.5H), 2.26 (s, 1.5H), 1.81 – 1.61 (m, 4H), 1.44-1.31 (m, 4H), 1.19 (s, 1.5H), 1.04 (s, 1.5H). **HRMS (ESI+):** calcd. for $C_{24}H_{28}O_2Na^+$ $[M+Na]^+$ 371.1986, found 371.1986.

3) Equivalent (dtbbpy)NiII(2-tolyl)I involved in the three-component reaction of alkyne

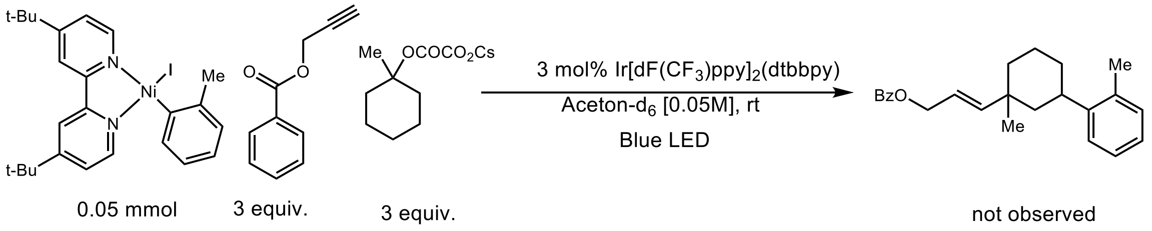


To a flame-dried 8 mL reaction vial was charged Ir[dF($CF_3$)ppy]$_2$(dtbbpy)$PF_6$ (0.003 mmol, 3.3 mg, 3 mol%), and cesium 2-((1,4-dimethylcyclohexyl)oxy)-2-oxoacetate (0.15 mmol, 49.8 mg, 3 equiv.), (dtbpy)NiII(2-tolyl)I (0.05 mmol, 27.2 mg, 1 equiv ), 1 mL aceton-d6 was added in an argon-filled glovebox, followed by the addition of prop-2-yn-1-yl benzoate (21.75 uL, 0.15 mmol, 3.0 equiv.). The 1.5 cm away from the light source with cooling from fan for 12 h. No product was detected via 1H NMR analysis of the crude mixture and preparative TLC.

**d) Decarboxylative radical addition of alkynes**

1) Three-component reaction

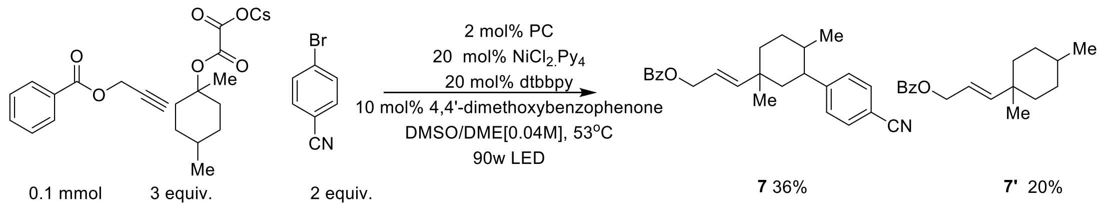


To a flame-dried 8 mL reaction vial was charged with $NiCl_2(Py)_4$ (0.02 mmol, 8.9 mg, 20 mol%), dtbbpy (0.02 mmol, 5.4 mg, 20 mol%), Ir[dF($CF_3$)ppy]$_2$(dtbbpy)$PF_6$ (0.002 mmol, 2.2 mg, 2 mol%), 4-Bromobenzonitrile (0.2 mmol, 36.4 mg, 2.0 equiv.) and cesium 2-((1,4-dimethylcyclohexyl)oxy)-2-oxoacetate (0.3 mmol, 99.6 mg, 3 equiv.), 4,4'-dimethoxybenzophenone (0.01 mmol, 2.42 mg, 10 mol%.) ,the vial was capped. DMSO/DME = 4:1 (2.5mL) was added via a syringe, nitrogen bubbling for fifteen minutes, followed by the addition of prop-2-yn-1-yl benzoate (14.5 uL, 0.1 mmol, 1.0 equiv.). The reaction mixture was then irradiated with a 90 W blue LED lamp (at approximately 1.5 cm away from the light source) for 18h. The reaction was quenched with $H_2O$, extracted with ethyl acetate. The combined organic layers were dried with $MgSO_4$, filtered, and concentrated in vacuo.

2) Two-component reaction

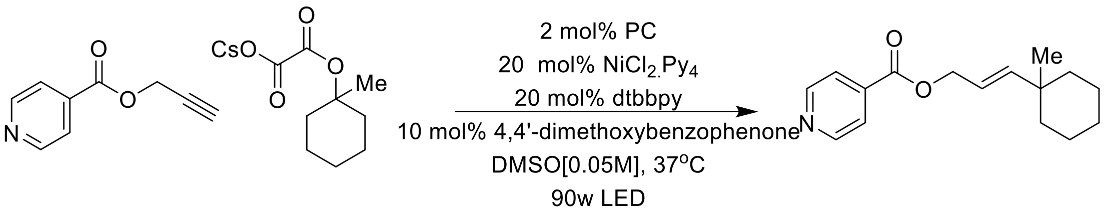


0.1 mmol 3 equiv. **S23** 10%, E/Z=93:7

To a flame-dried 8 mL reaction vial was charged with NiCl2(Py)4 (0.02 mmol, 8.9 mg, 20 mol%), dtbbpy (0.02 mmol,5.4 mg,20 mol%), Ir[dF($CF_3$)ppy]$_2$(dtbbpy)$PF_6$ (0.002 mmol, 2.2 mg, 2 mol%), cesium 2-((1-methylcyclohexyl)oxy)-2-oxoacetate (0.3 mmol, 95.4 mg, 3 equiv.), 4,4'-dimethoxybenzophenone (0.01 mmol, 2.42 mg, 10 mol%.) ,the vial was capped. DMSO (2 mL) was added via a syringe, nitrogen bubbling for fifteen minutes, followed by the addition of prop-2-yn-1-yl isonicotinate (16.1 mg, 0.1 mmol, 1.0 equiv.). The 1.5 cm away from the light source with cooling from fan for 18 h. The reaction was quenched with $H_2O$, extracted with ethyl acetate. The combined organic layers were dried with MgSO4, filtered, and concentrated in vacuo. The crude material was purified by flash chromatography (PE: EA= 10:1) as a pale yellow oil (5 mg, 10%, *Z/E* = 93:7).

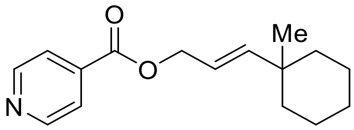


**(E)-3-(1-methylcyclohexyl)allyl isonicotinate (S23):** $^1$H NMR (600 MHz, $CDCl_3$) δ8.78 (d, *J* = 5.8 Hz, 2H), 7.86 (d, *J* = 5.9 Hz, 2H), 5.84 (d, *J* = 15.8 Hz, 1H), 5.60 (dt, *J* = 15.8, 6.6 Hz, 0.93H), 5.52 (d, *J* = 6.2 Hz, 0.07H), 5.01 (d, *J* = 5.2 Hz, 0.13H) ,4.82 (d, *J* = 6.6 Hz,1.87H), 1.50 – 1.44 (m, 5H), 1.39 (d, *J* = 4.5 Hz, 2H), 1.34 – 1.29 (m, 3H), 1.12 (s, 0.23H), 1.00 (s, 2.78H). $^{13}$C NMR (151 MHz, $CDCl_3$) δ 179.57, 169.50, 165.00, 150.57, 122.93, 119.42, 67.20, 37.59, 35.97, 29.73, 26.22, 22.27.

**HRMS (EI):** m/z calcd. for $C_{16}H_{21}O_2N^+$ $[M]^+$:259.1572 ; Found: 259.1477.

# 6. Spectral Data

## Prop-2-yn-1-yl 4-acetoxybenzoate (S1)

$^{1}H$ NMR (600 MHz, $CDCl_3$)

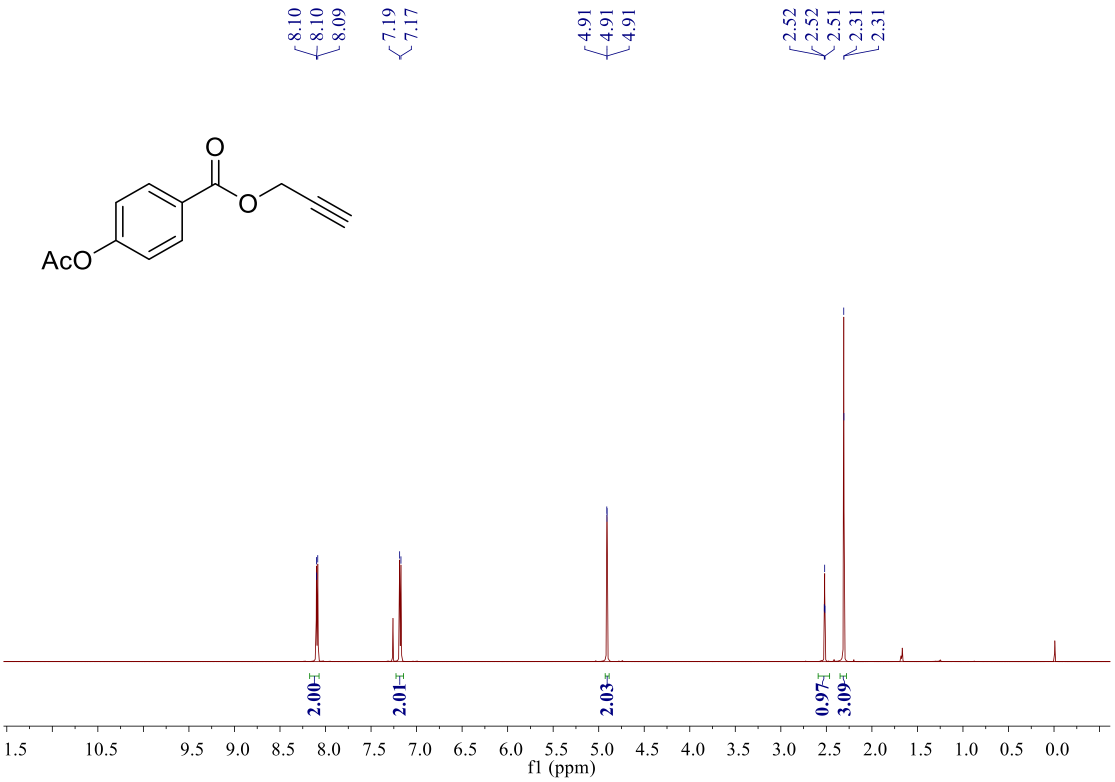


$^{13}C$ NMR (150 MHz, $CDCl_3$)

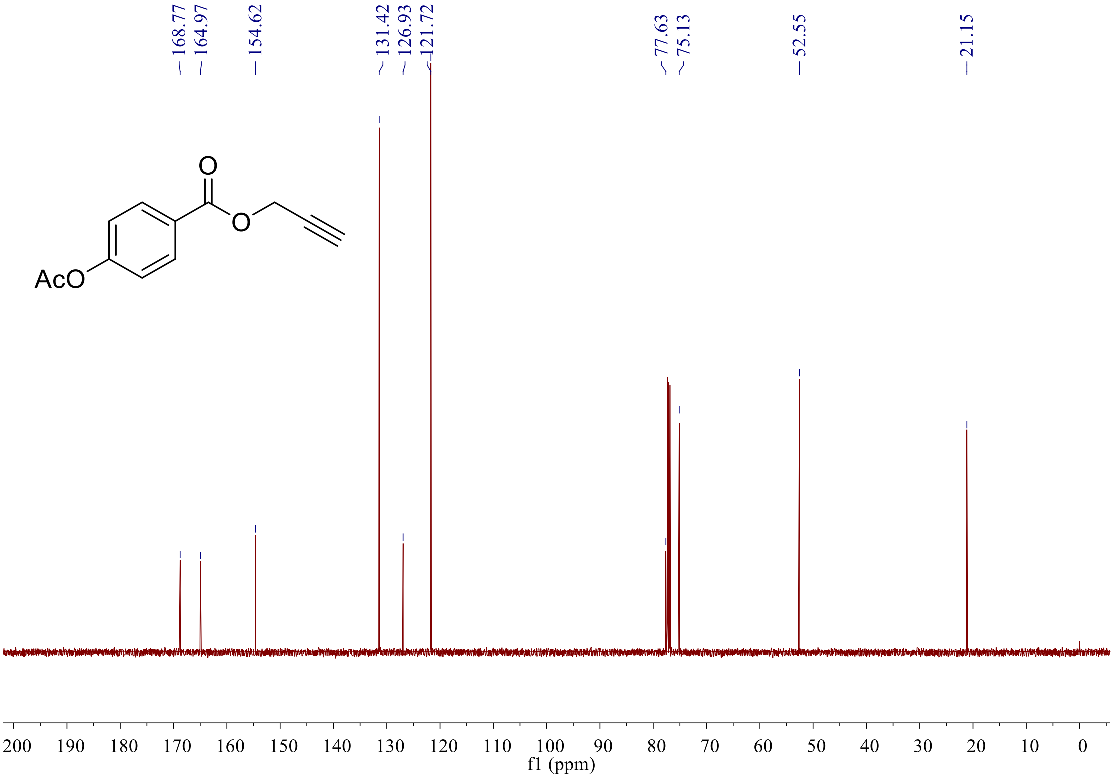

**Prop-2-yn-1-yl isonicotinate (S2)**

$^{1}H$ NMR (600 MHz, $CDCl_3$)

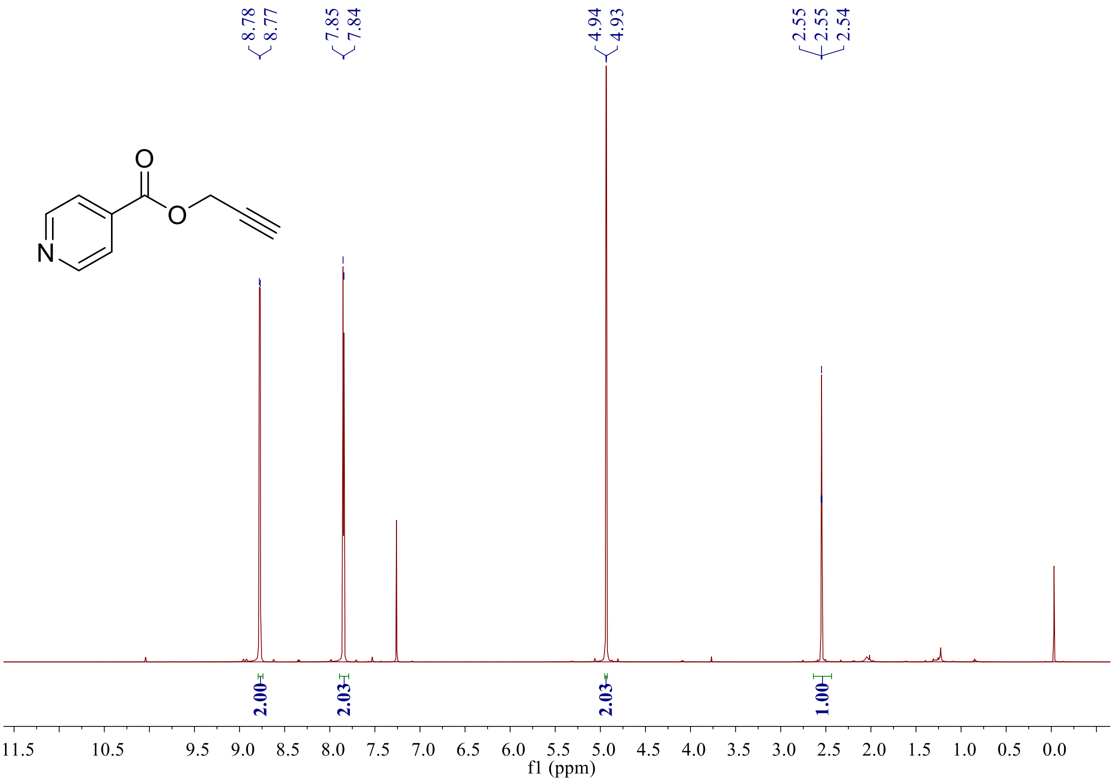


$^{13}C$ NMR (150 MHz, $CDCl_3$)

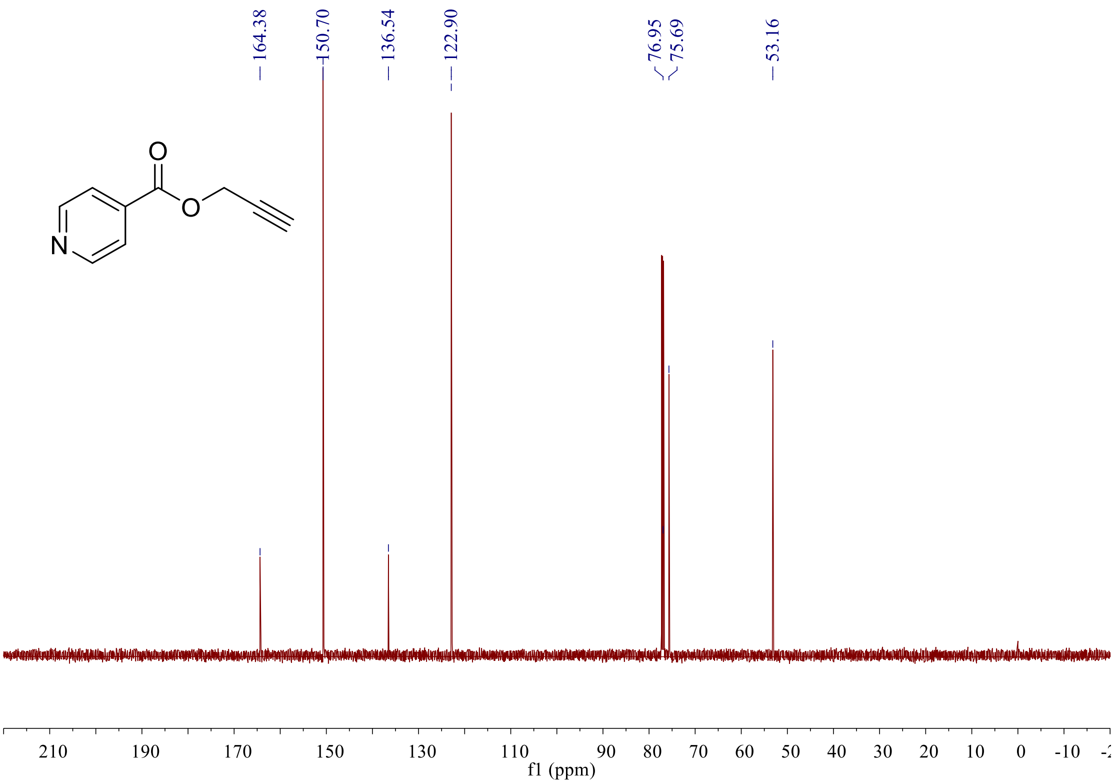

**prop-2-yn-1-yl 3-(4,5-diphenyloxazol-2-yl)propanoate (S3)**

$^{1}$H NMR (600 MHz, $CDCl_3$)

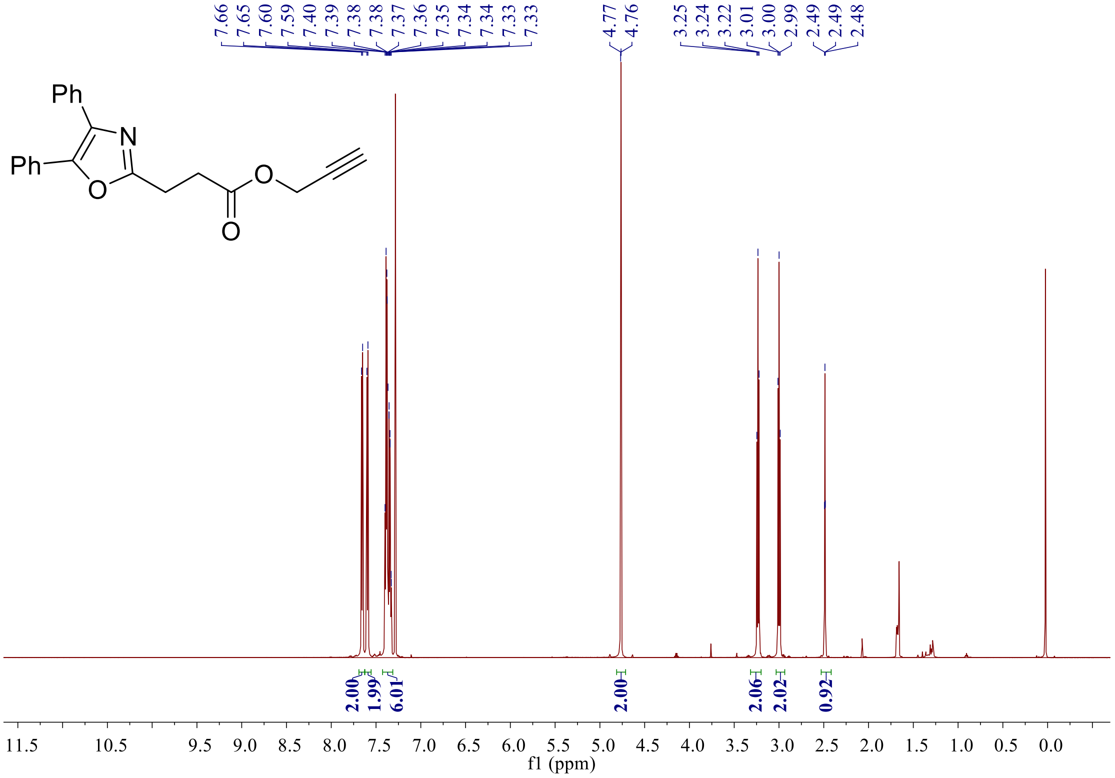


$^{13}$C NMR (150 MHz, $CDCl_3$)

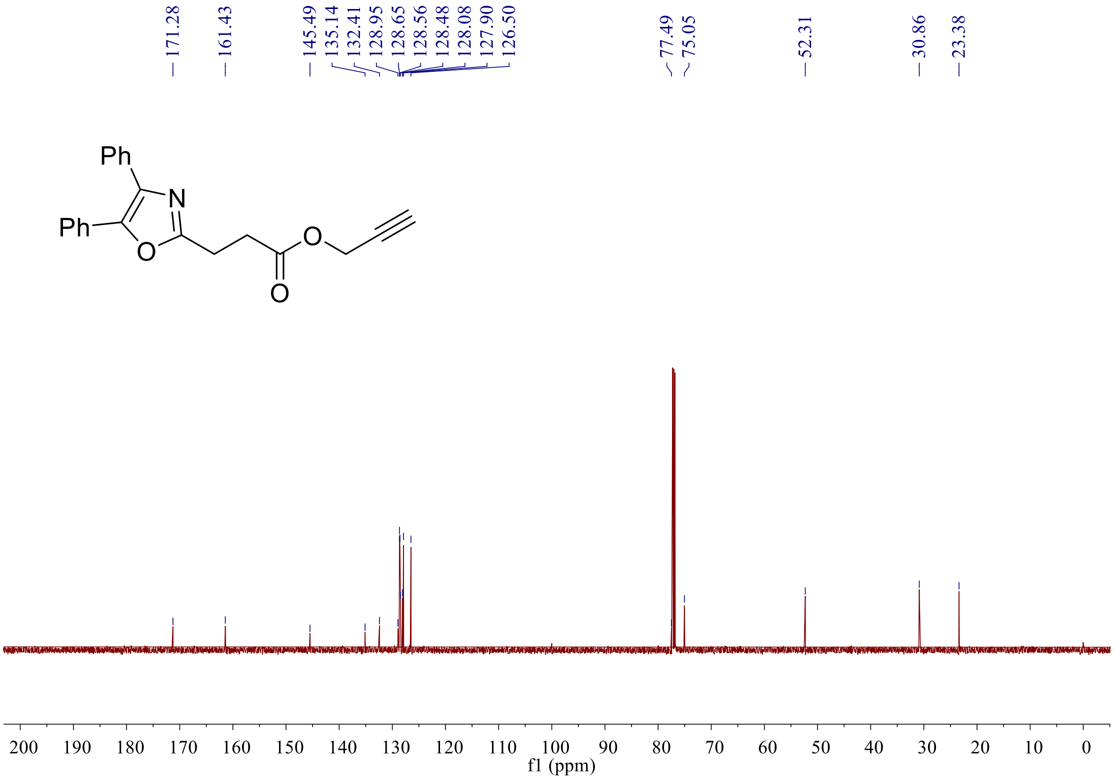

**Prop-2-yn-1-yl 4-(tert-butyl)benzoate (S4)**

$^{1}H$ NMR (600 MHz, $CDCl_3$)

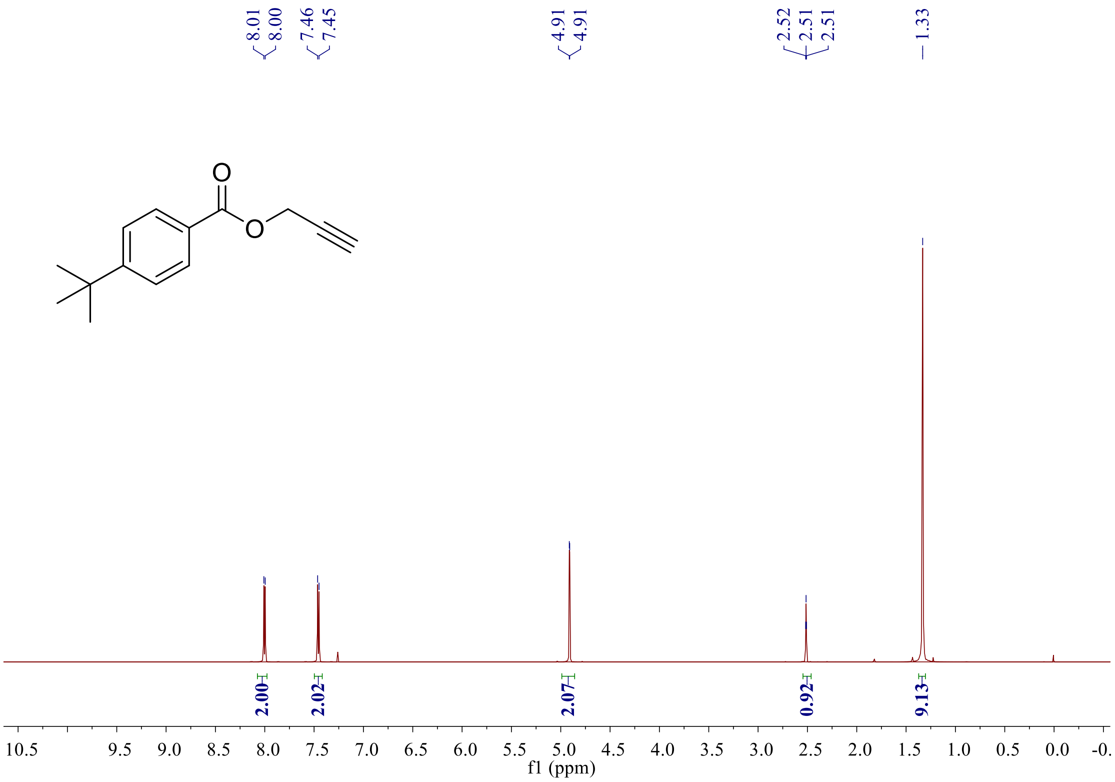


$^{13}C$ NMR (150 MHz, $CDCl_3$)

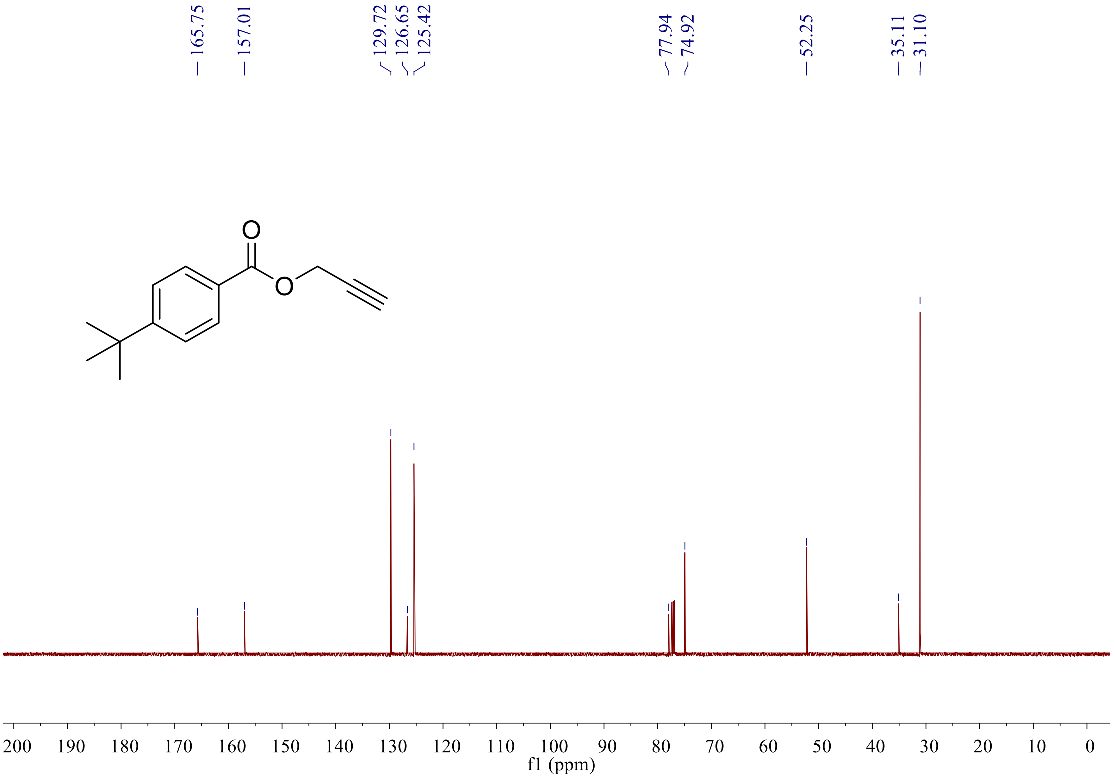

**Prop-2-yn-1-yl cyclohexanecarboxylate(S5):**

$^{1}$H NMR (600 MHz, $CDCl_3$)

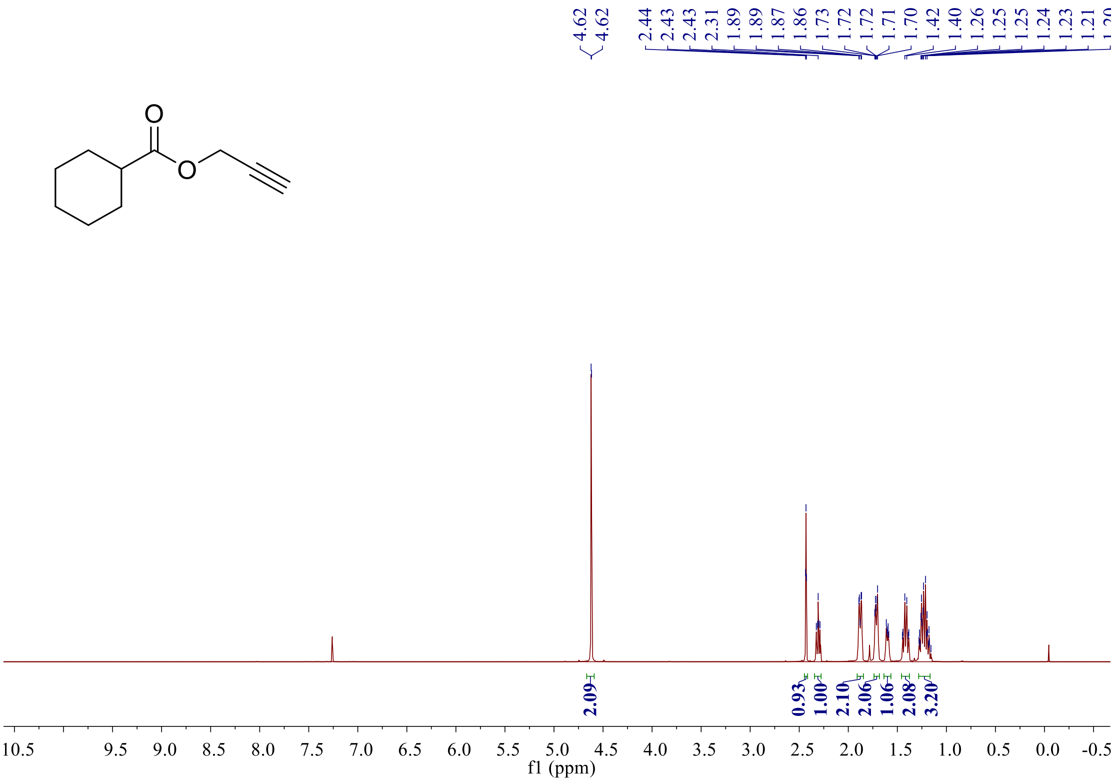


$^{13}$C NMR (150 MHz, $CDCl_3$)

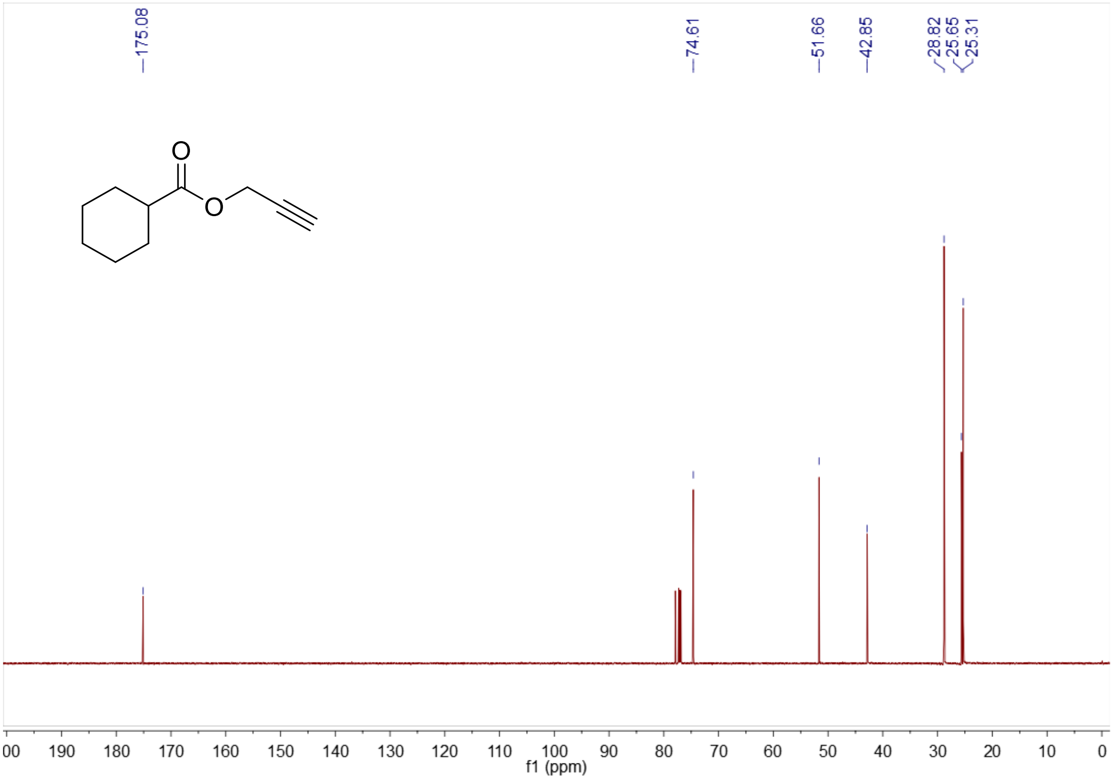

**2-(Prop-2-yn-1-yl)isoindoline-1,3-dione(S6):**

$^{1}$H NMR (600 MHz, CDCl$_3$)

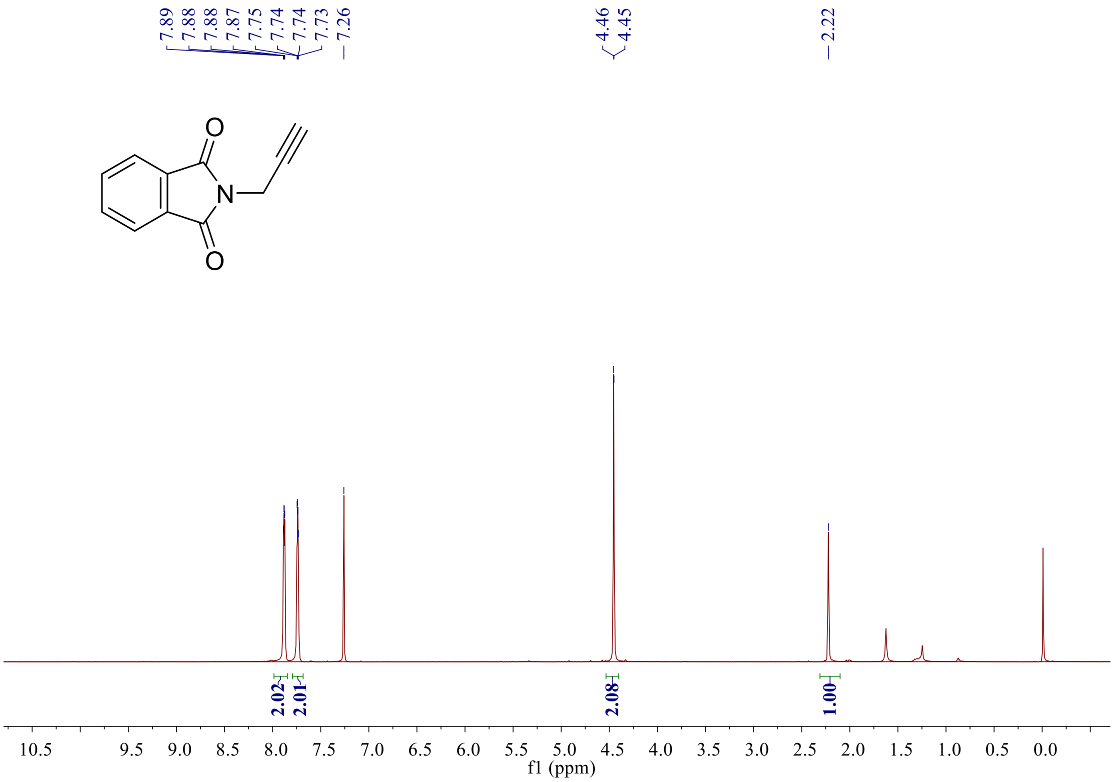

$^{13}$C NMR (150 MHz, CDCl$_3$)

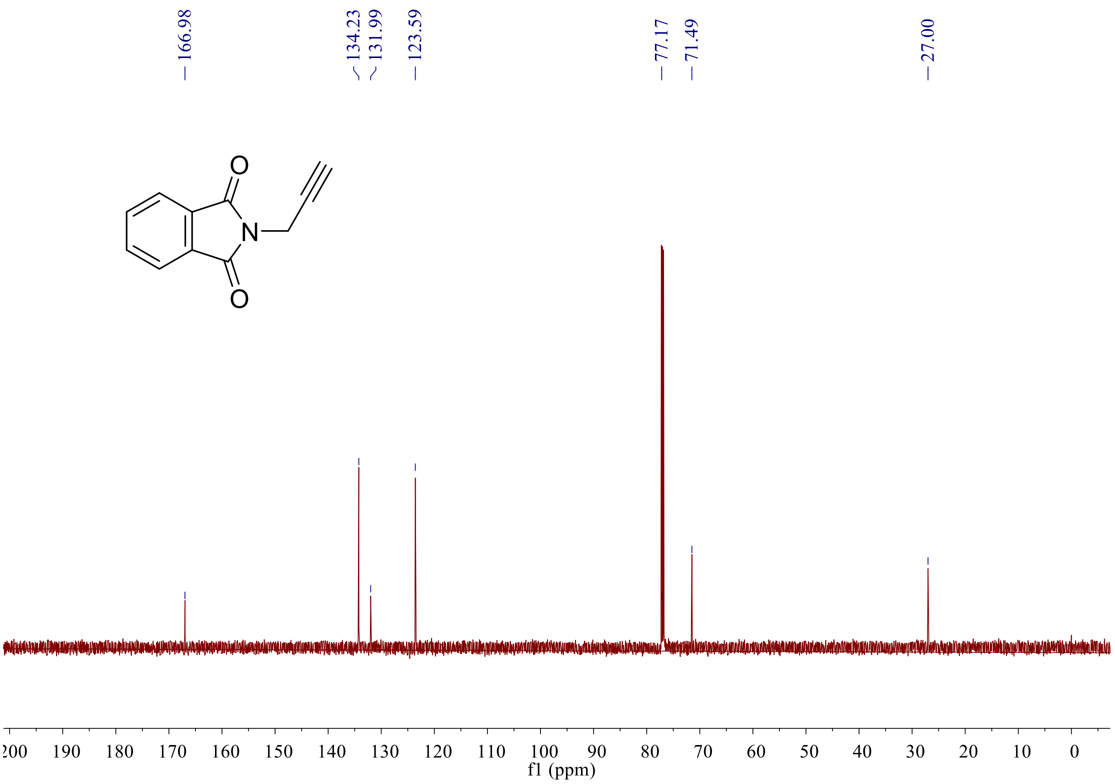

**Prop-2-yn-1-yl-7-isopropyl-1,4a-dimethyl-1,2,3,4,4a,9,10,10a-octahy drop henan-threne -1-carboxylate(S7):**

$^{1}H$ NMR (600 MHz, $CDCl_3$)

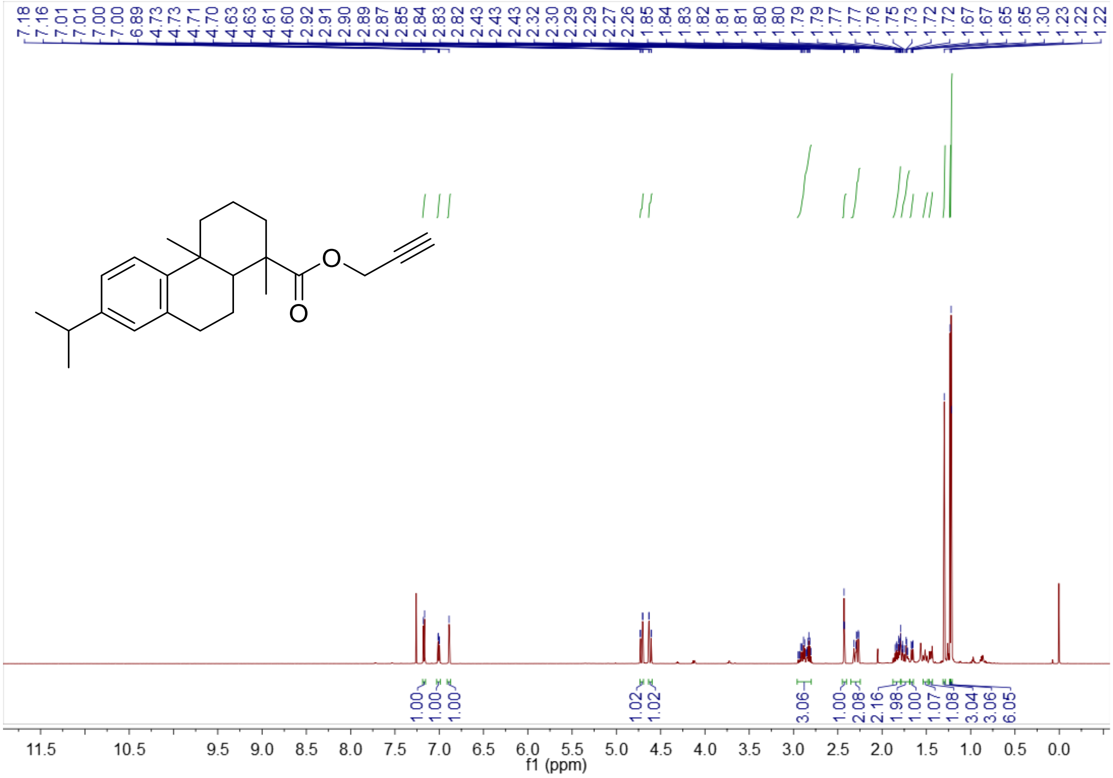

$^{13}C$ NMR (150 MHz, $CDCl_3$)

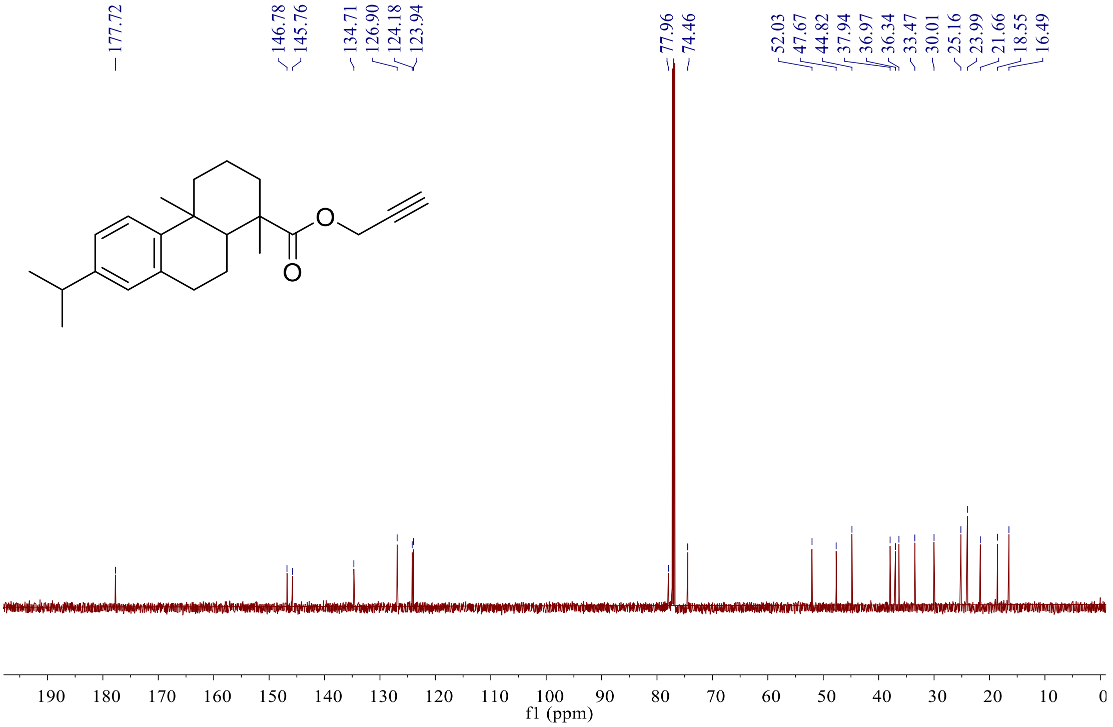

**Prop-2-yn-1-yl 1-methyl-1H-indole-3-carboxylate(S8):**

$^{1}H$ NMR (400 MHz, $CDCl_3$)

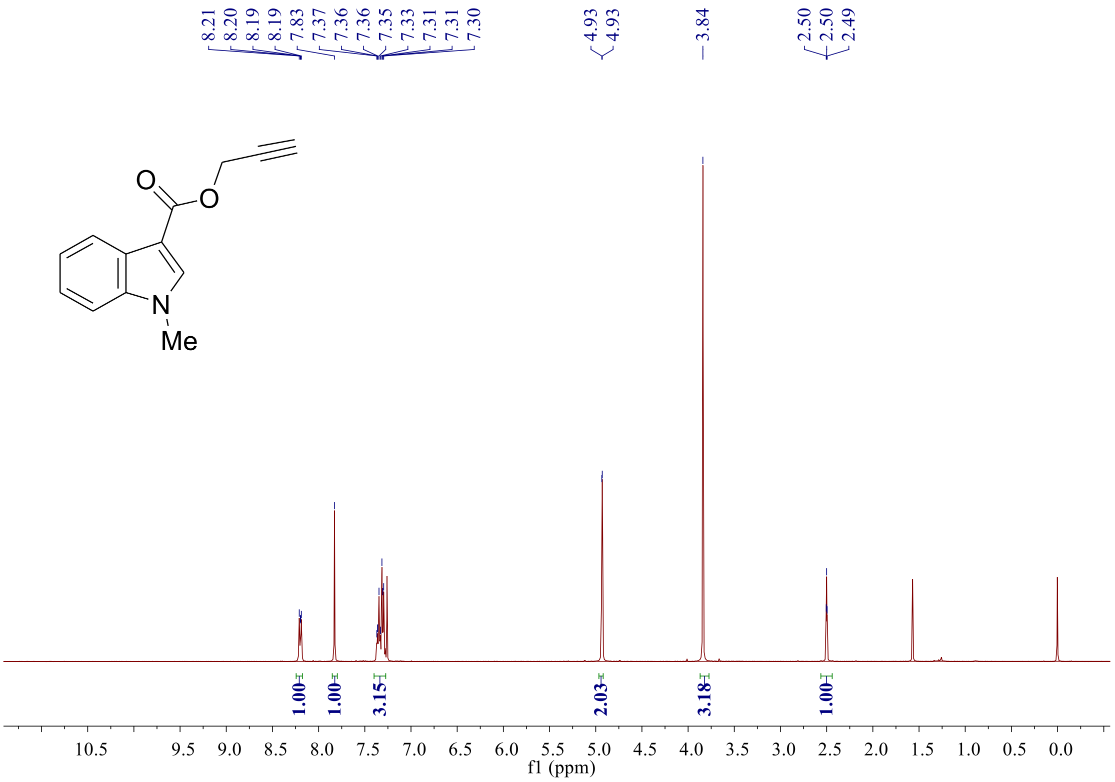


$^{13}C$ NMR (100 MHz, $CDCl_3$)

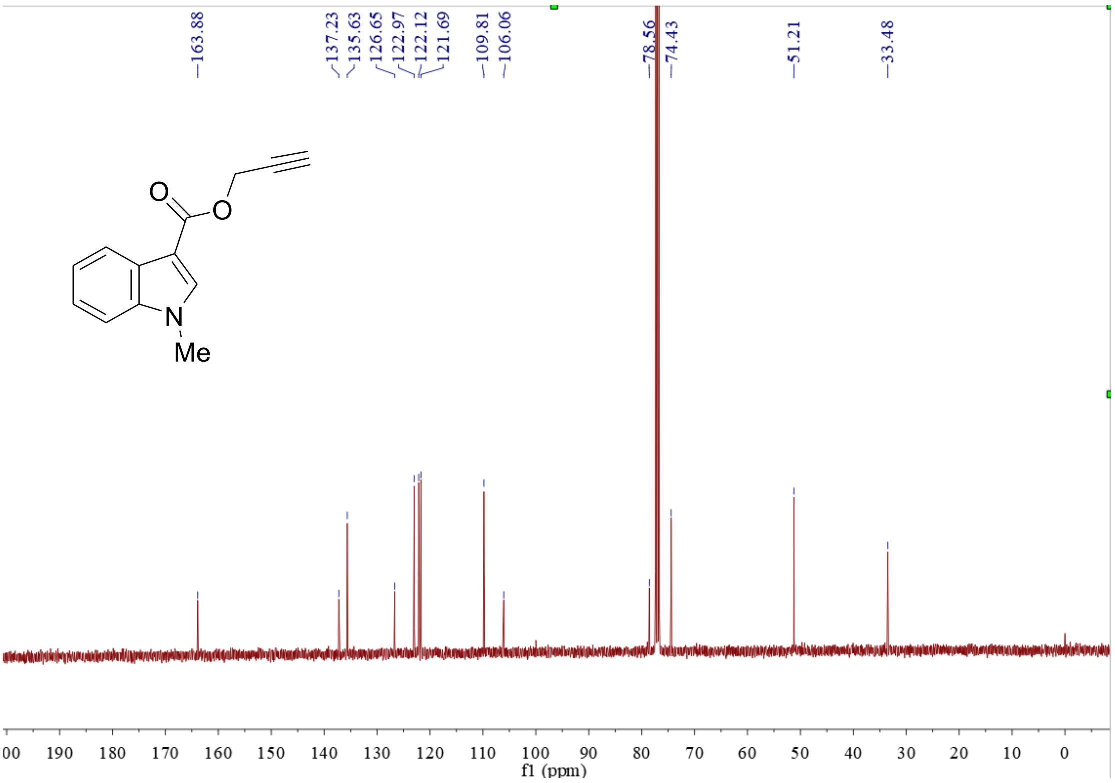

**(1S,2S)-1,2-dimethylcyclohexyl methyl oxalate(S9):**

$^{1}$H NMR (600 MHz, $CDCl_3$)

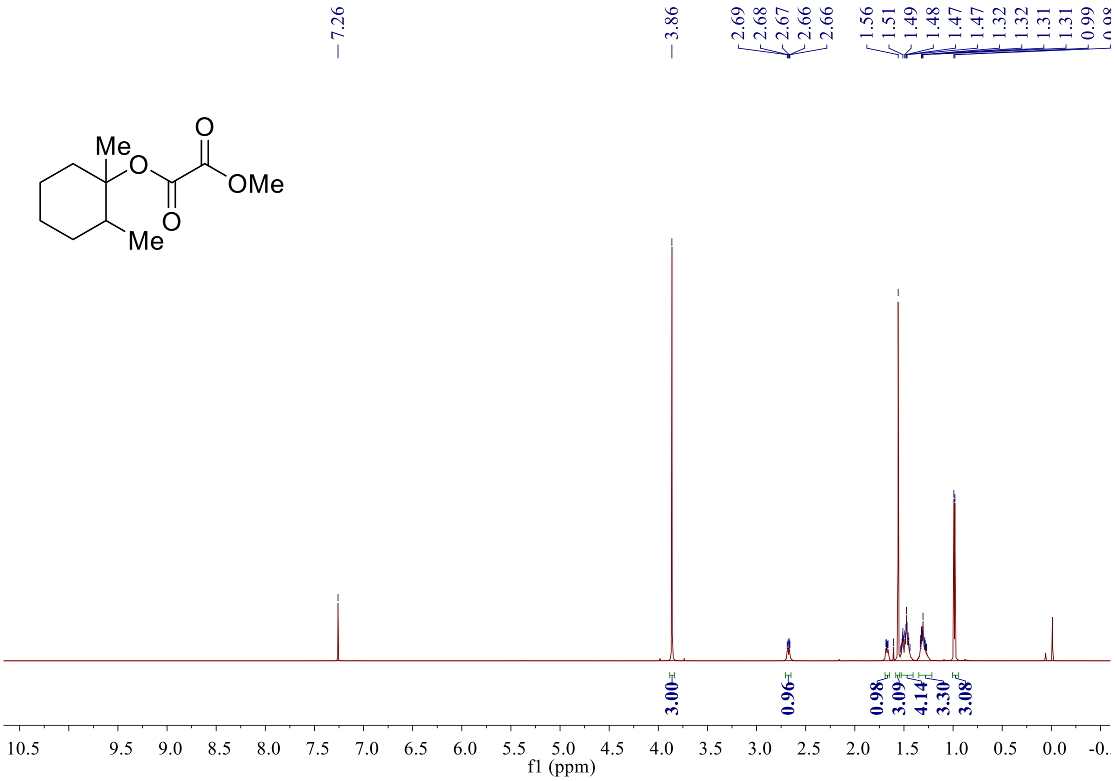


$^{13}$C NMR (150MHz, $CDCl_3$)

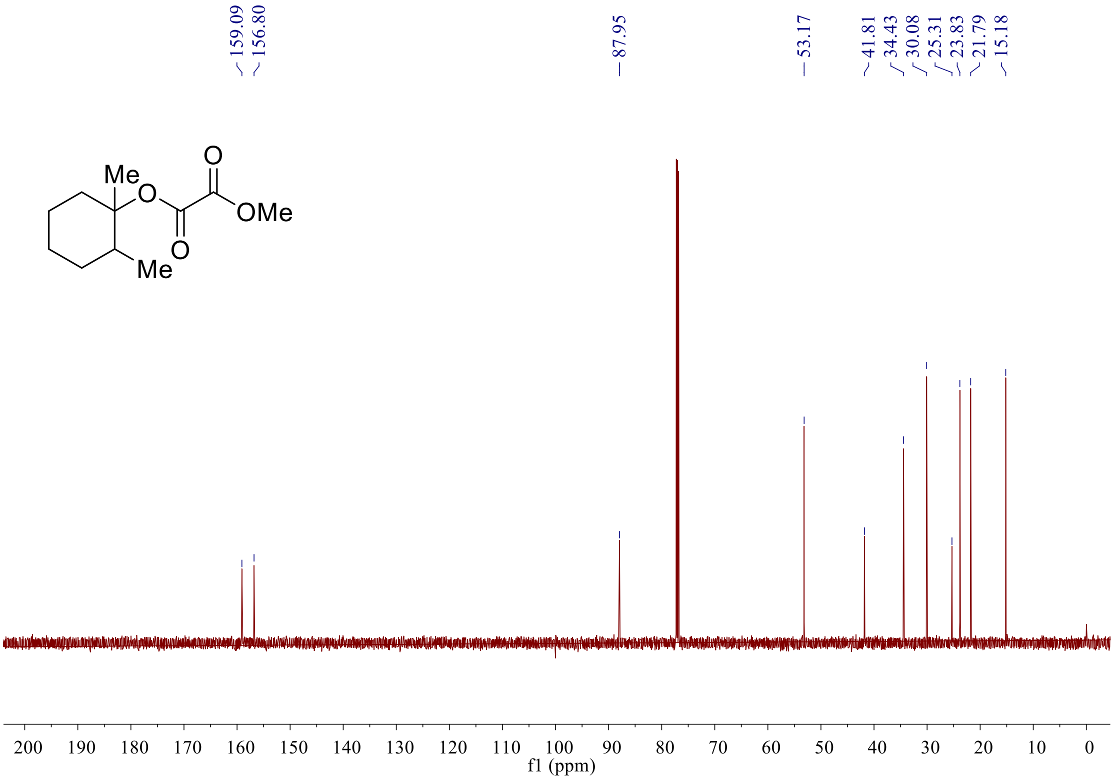

**Cesium 2-(((1S,2S)-1,2-dimethylcyclohexyl)oxy)-2-oxoacetate (S10):**

$^{1}H$ NMR (600 MHz, DMSO)

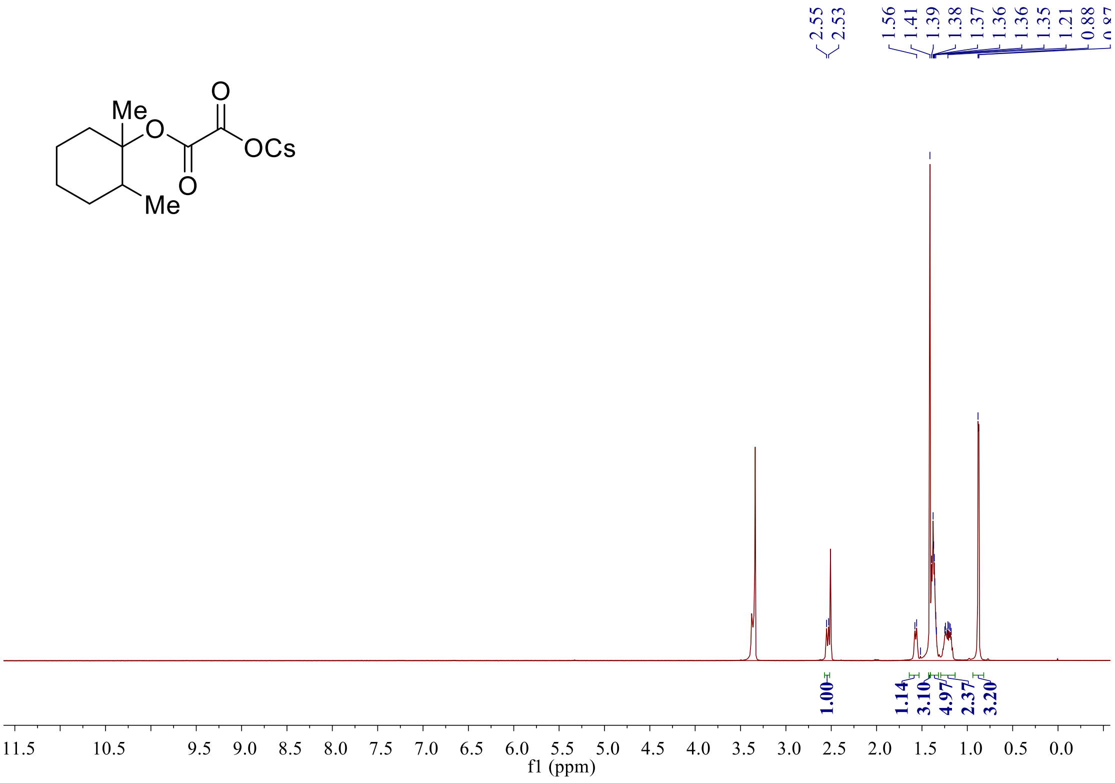


$^{13}C$ NMR (150 MHz, DMSO)

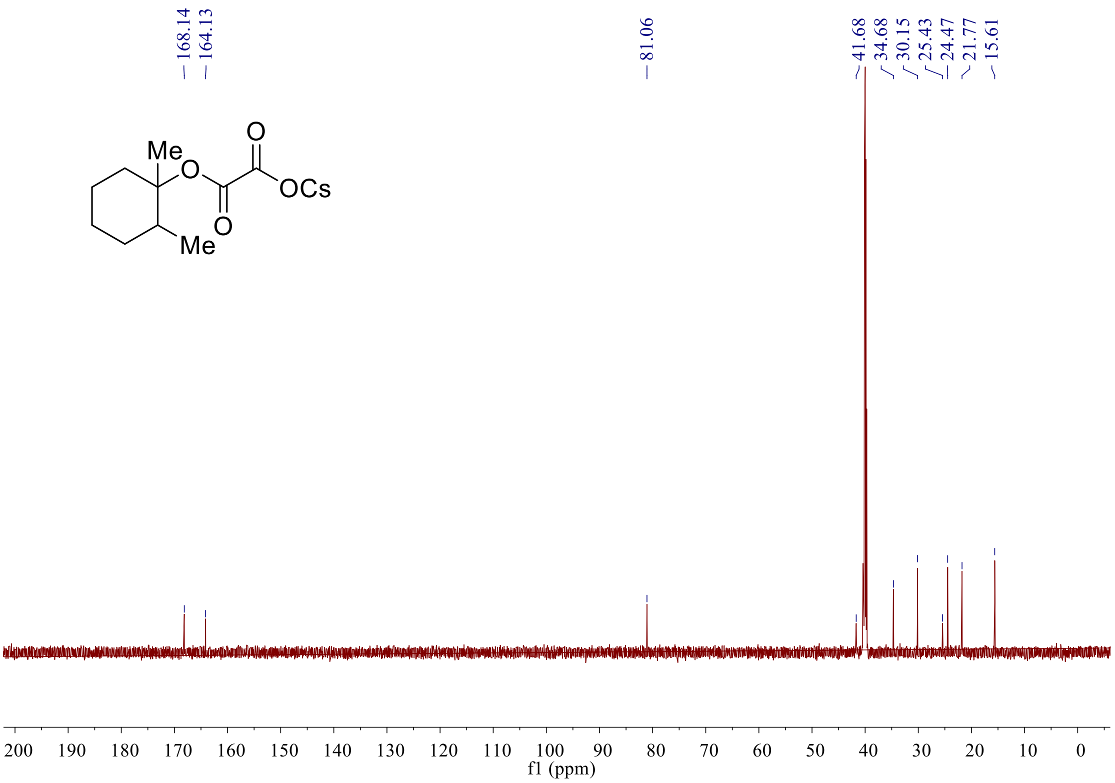

**1,4-dimethylcyclohexyl methyl oxalate (S11):**

$^1$H NMR (600 MHz, $CDCl_3$)

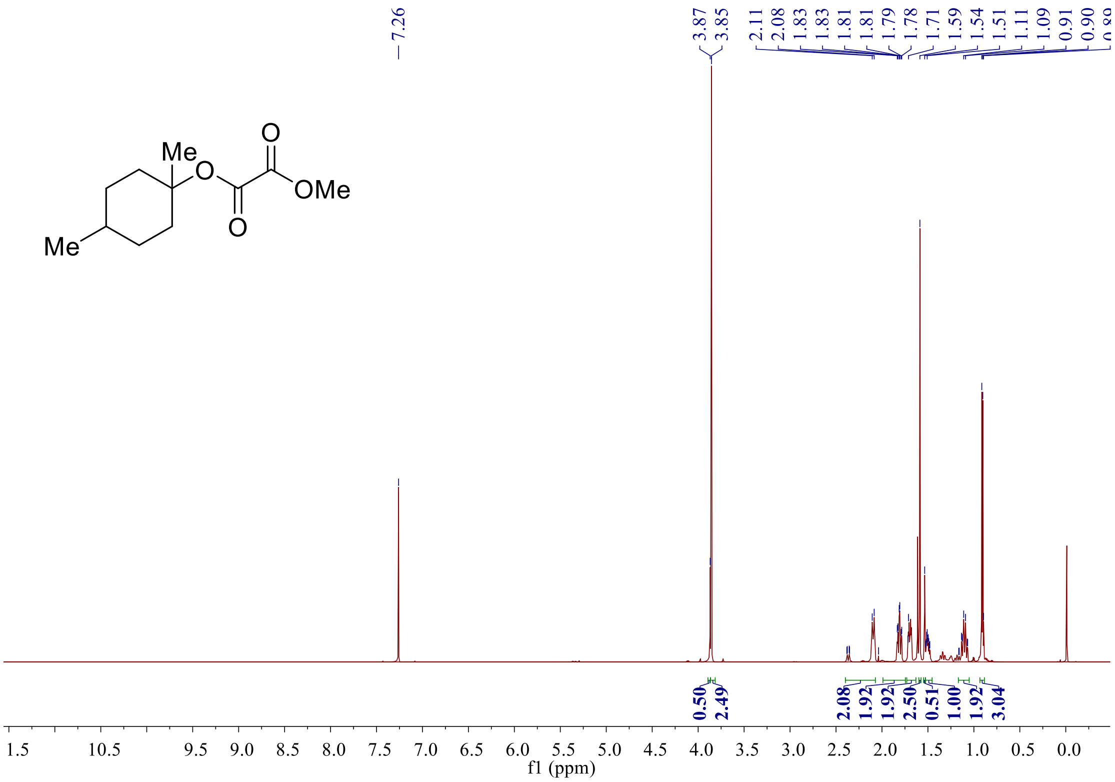

$^{13}$C NMR (150 MHz, $CDCl_3$)

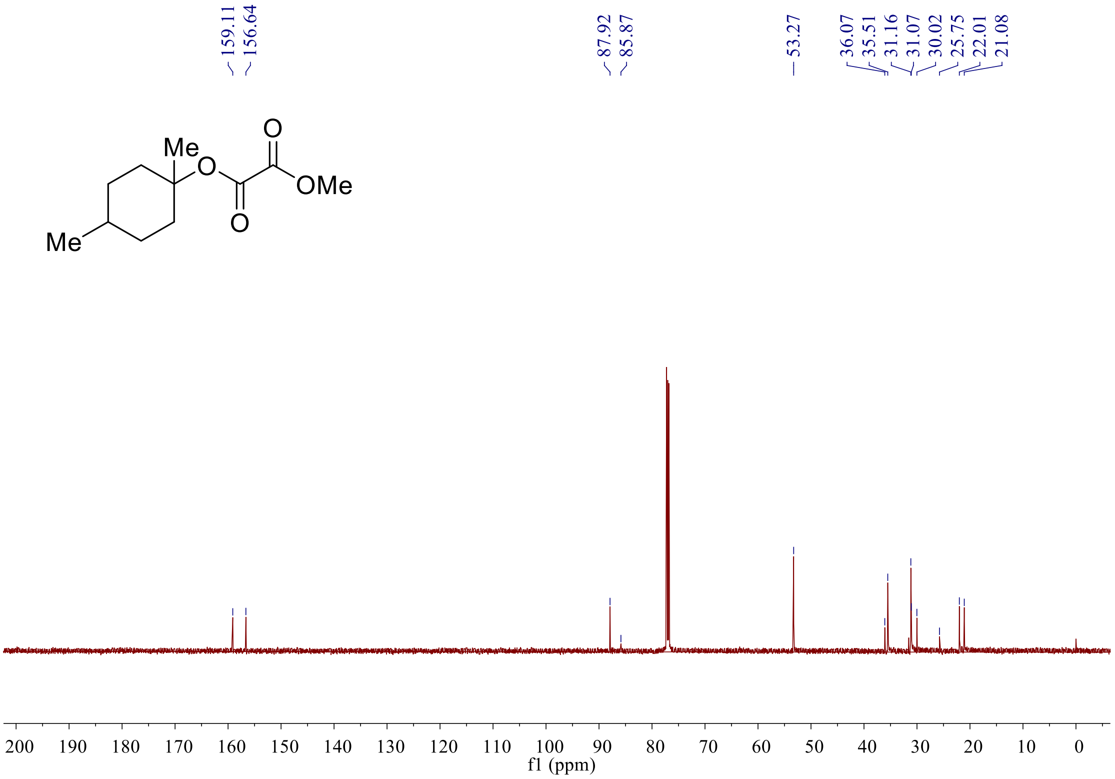

**Cesium 2-((1,4-dimethylcyclohexyl)oxy)-2-oxoacetate (S12):**

$^{1}$H NMR (400 MHz, DMSO)

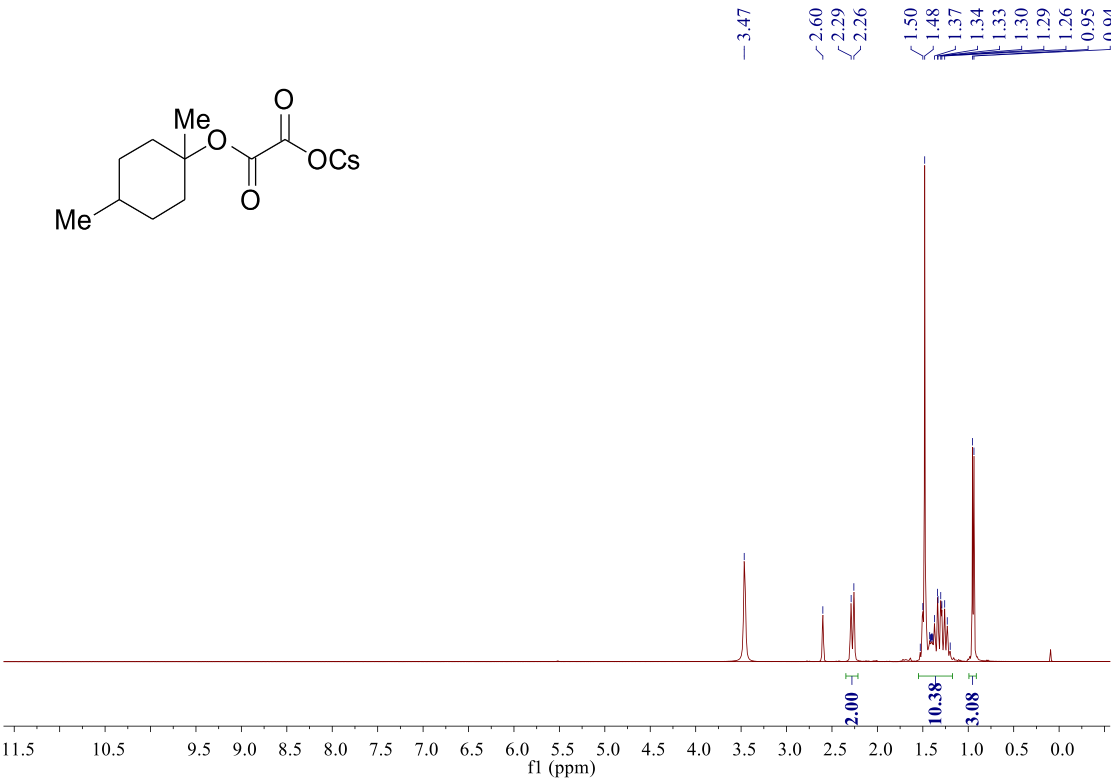


$^{13}$C NMR (100 MHz, DMSO)

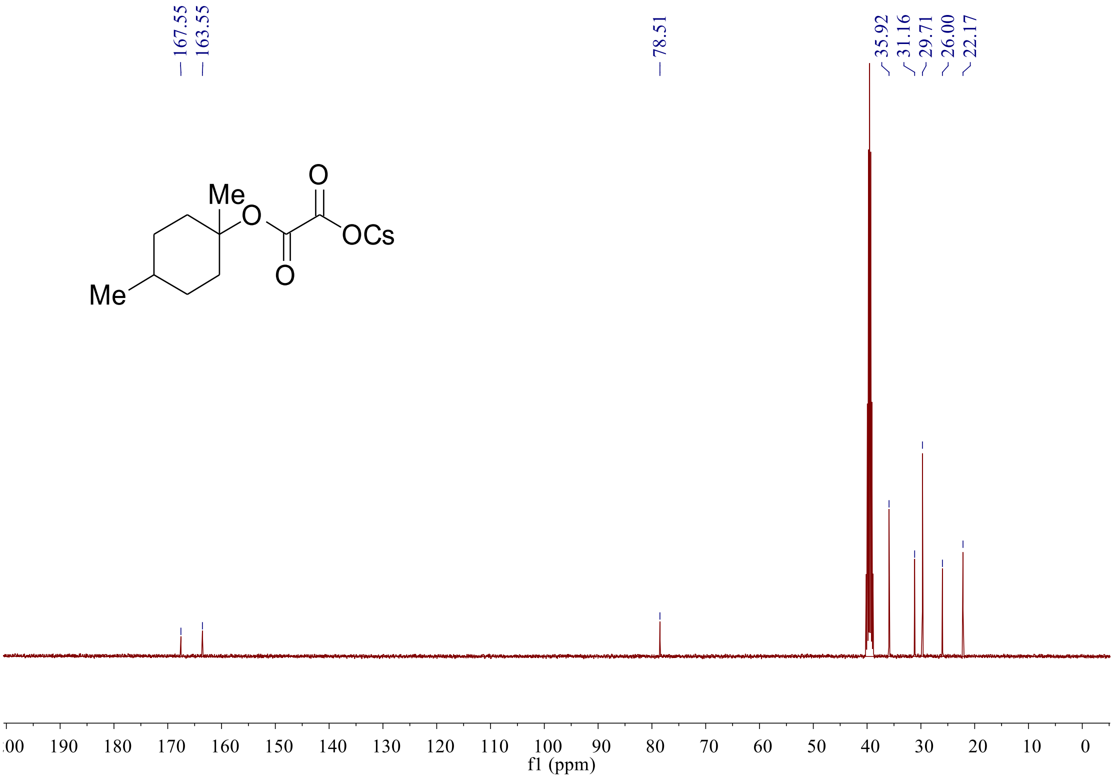

**Methyl (3-methyltetrahydro-2H-pyran-3-yl) oxalate (S13):**

$^{1}H$ NMR (600 MHz, $CDCl_3$)

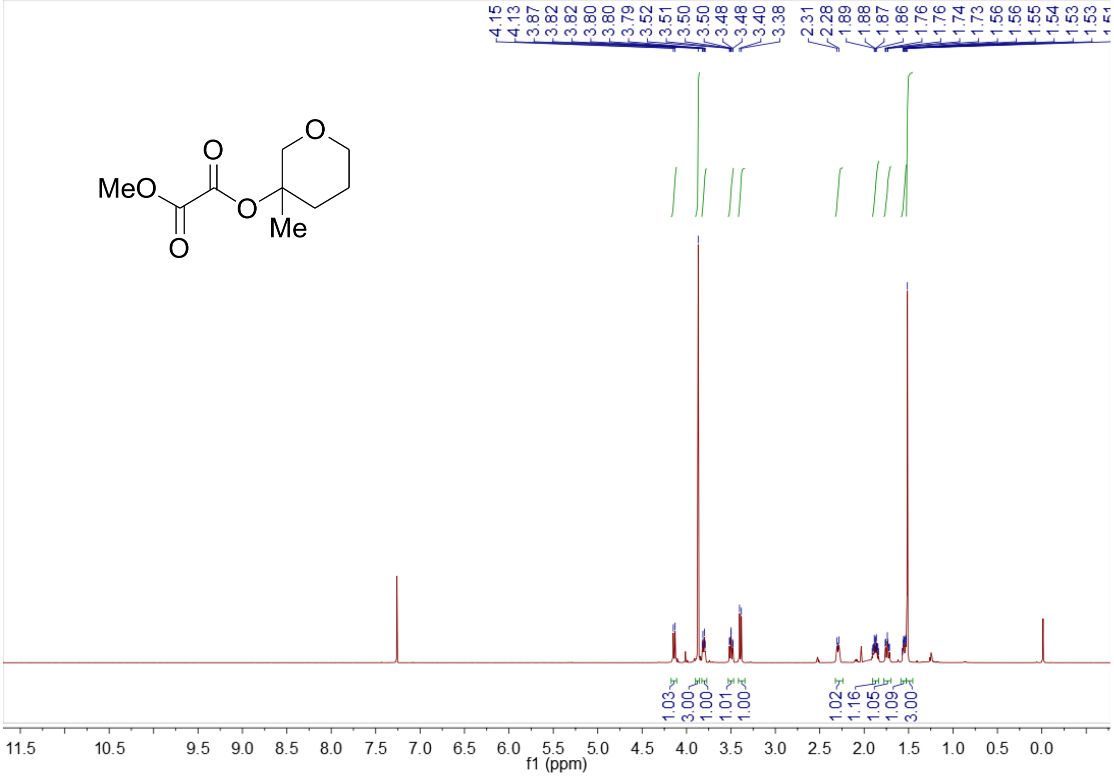


$^{13}C$ NMR (150 MHz, $CDCl_3$)

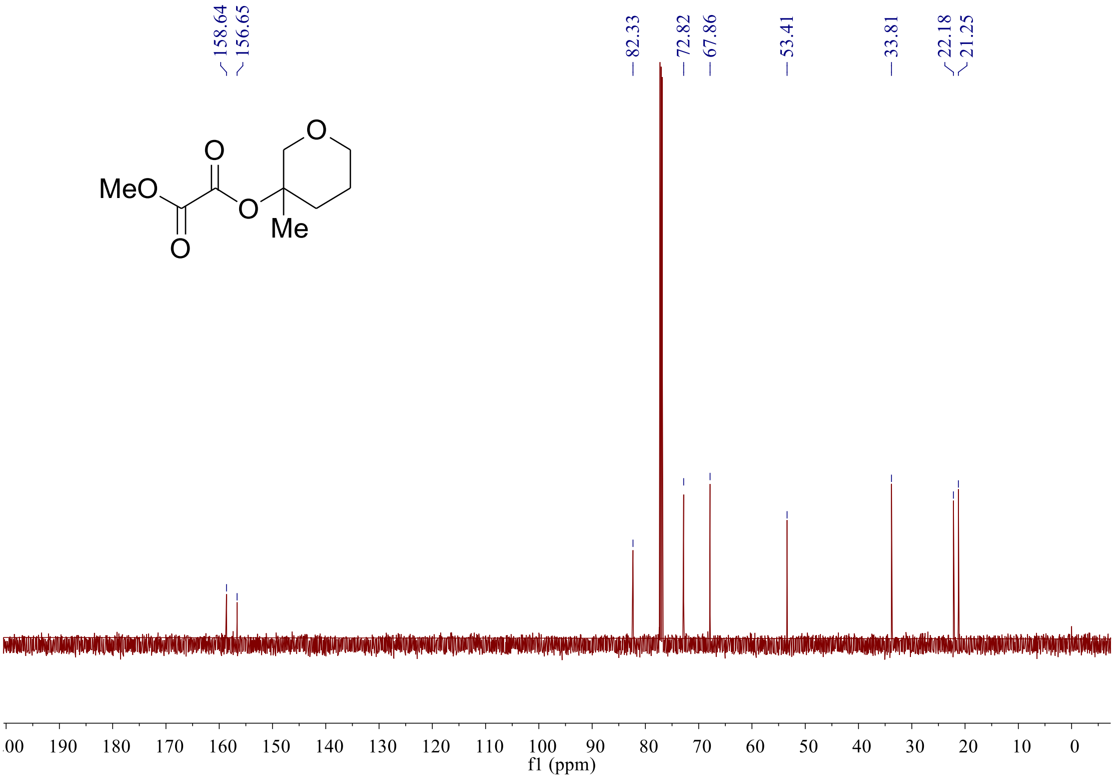

**Cesium 2-((3-methyltetrahydro-2H-pyran-3-yl)oxy)-2-oxoacetate (S14):**

$^{1}$H NMR (600 MHz, $CDCl_3$)

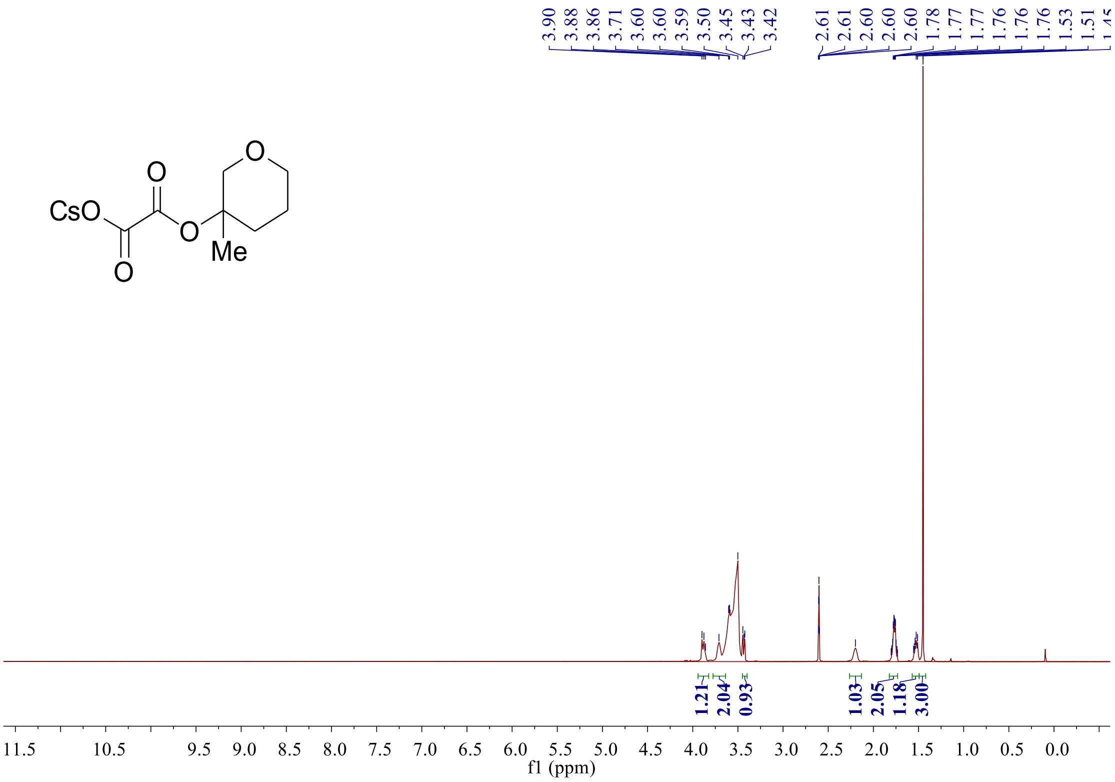


$^{13}$C NMR (150 MHz, DMSO)

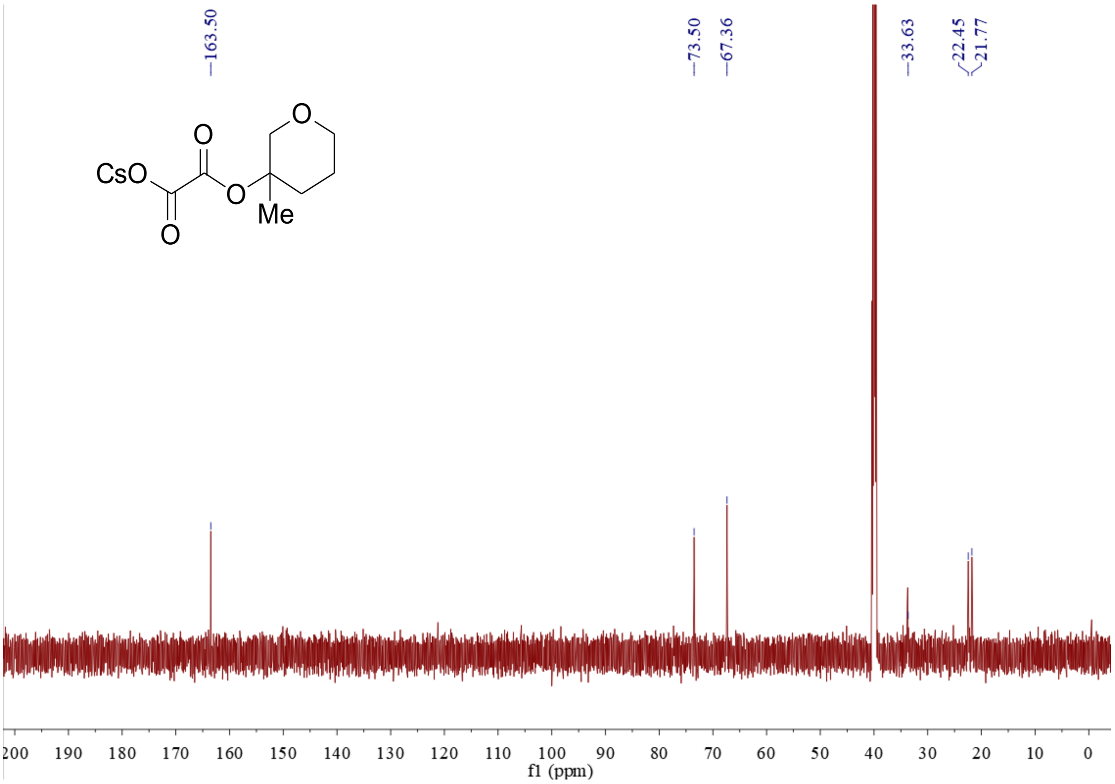

## 1- Ethylcyclohexyl methyl oxalate (S15):

$^{1}$H NMR (600 MHz, $CDCl_3$)

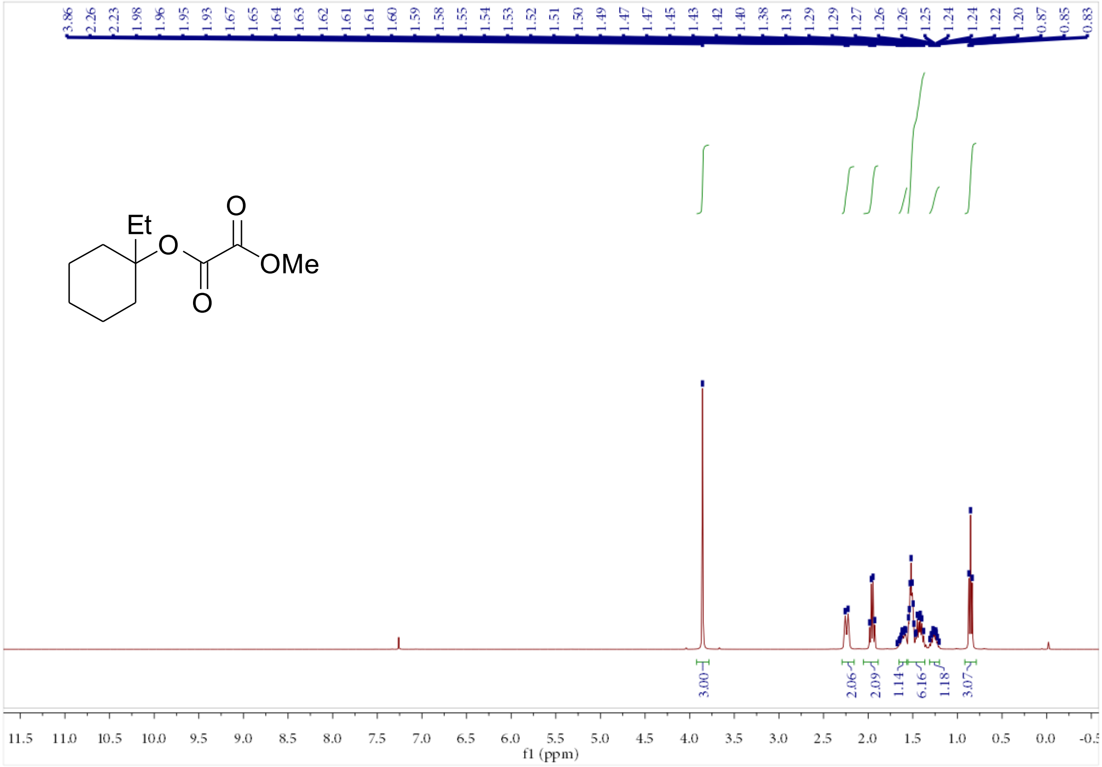

$^{13}$C NMR (150 MHz, $CDCl_3$)

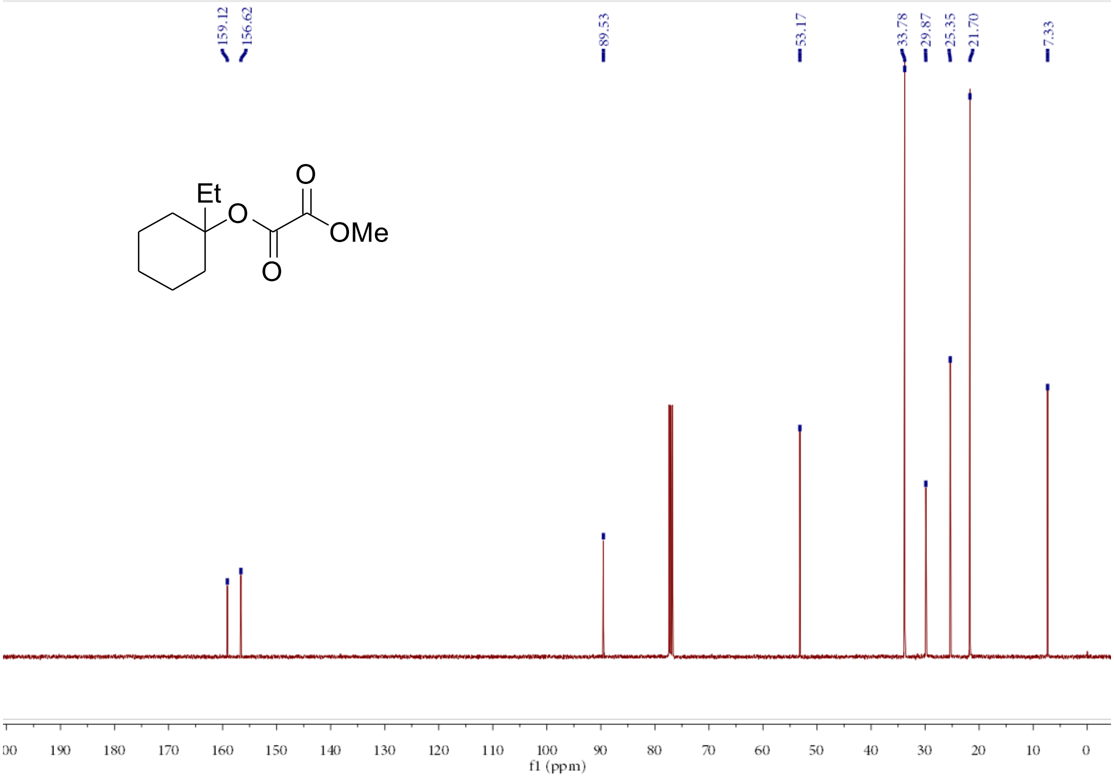

**Cesium 2-((1-ethylcyclohexyl)oxy)-2-oxoacetate (S16):**

$^{1}$H NMR (400 MHz, $D_2O$)

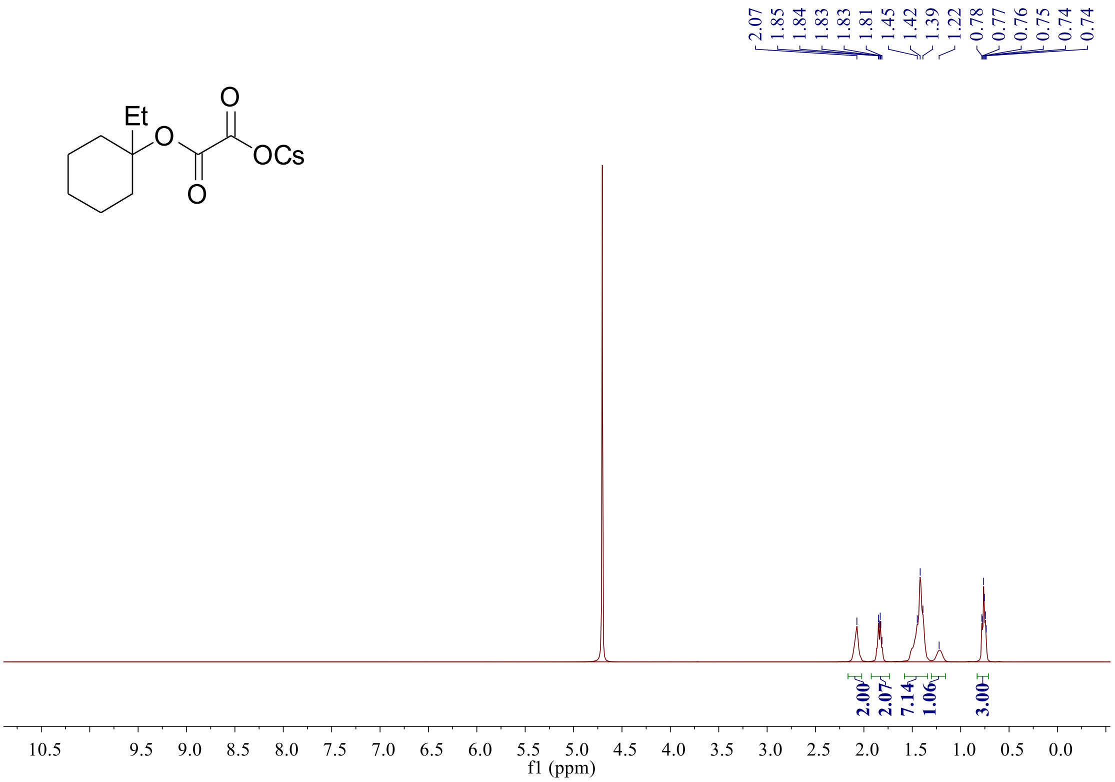


$^{13}$C NMR (150 MHz, $D_2O$)

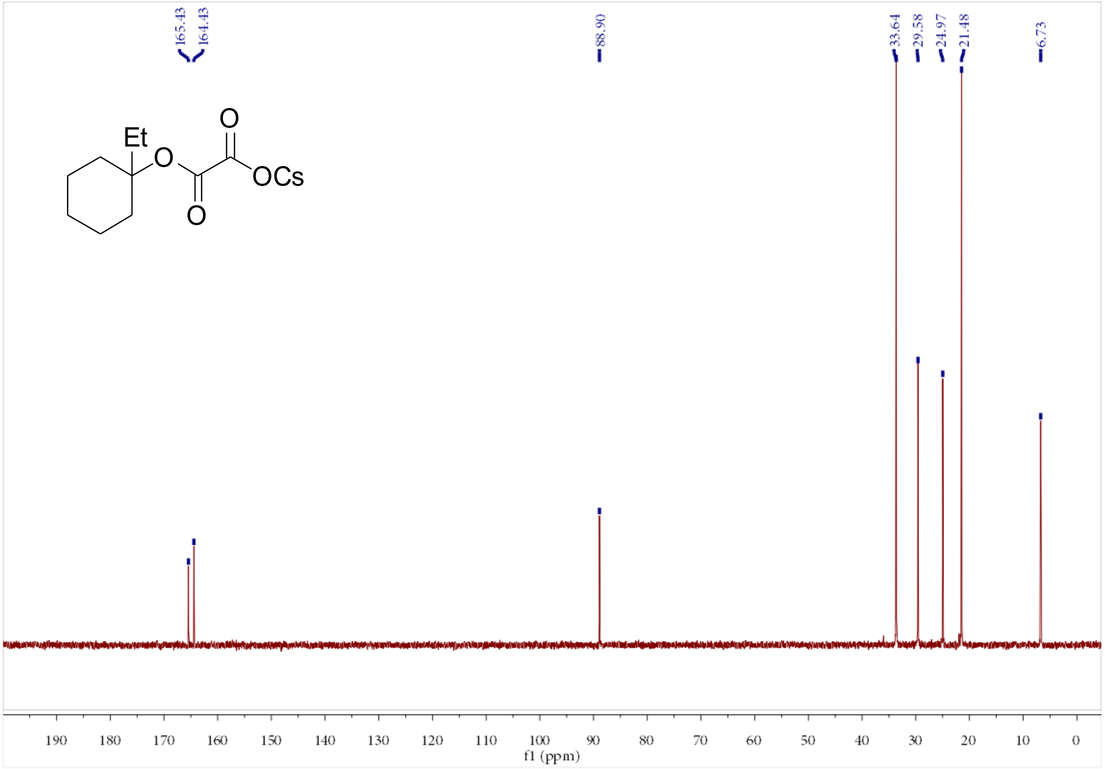

**Methyl (4-methyltetrahydro-2H-thiopyran-4-yl) oxalate (S17):**

$^{1}$H NMR (600 MHz, $CDCl_3$)

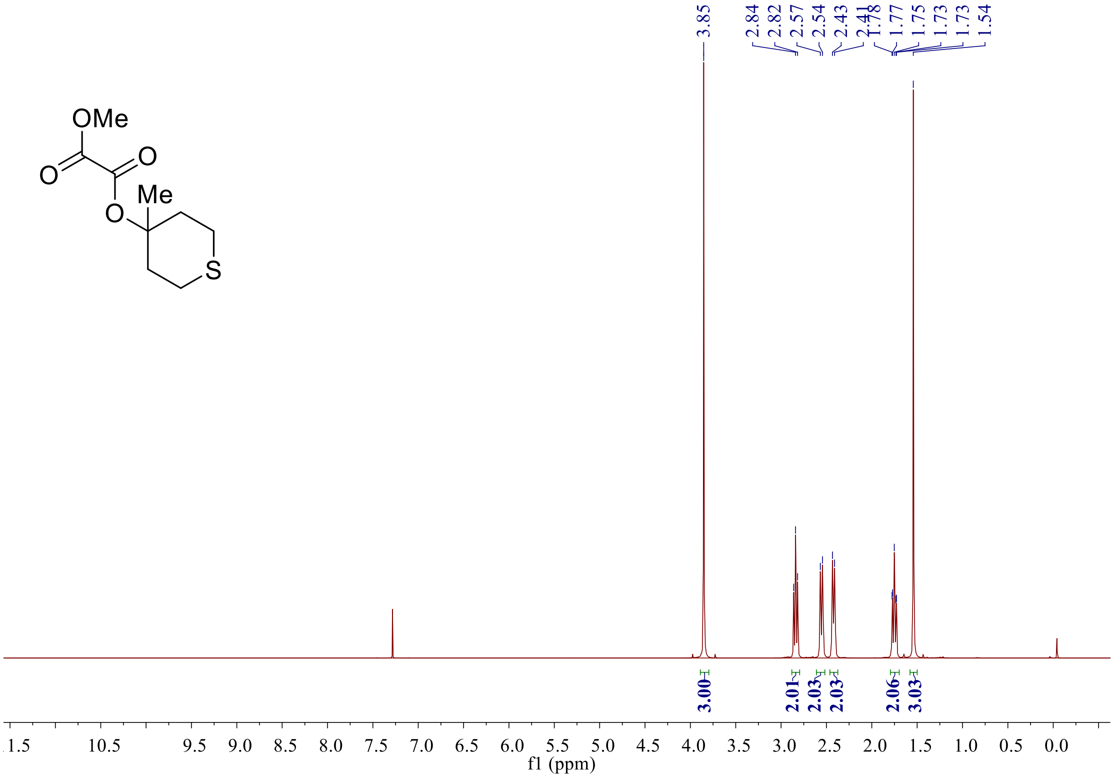


$^{13}$C NMR (150 MHz, $CDCl_3$)

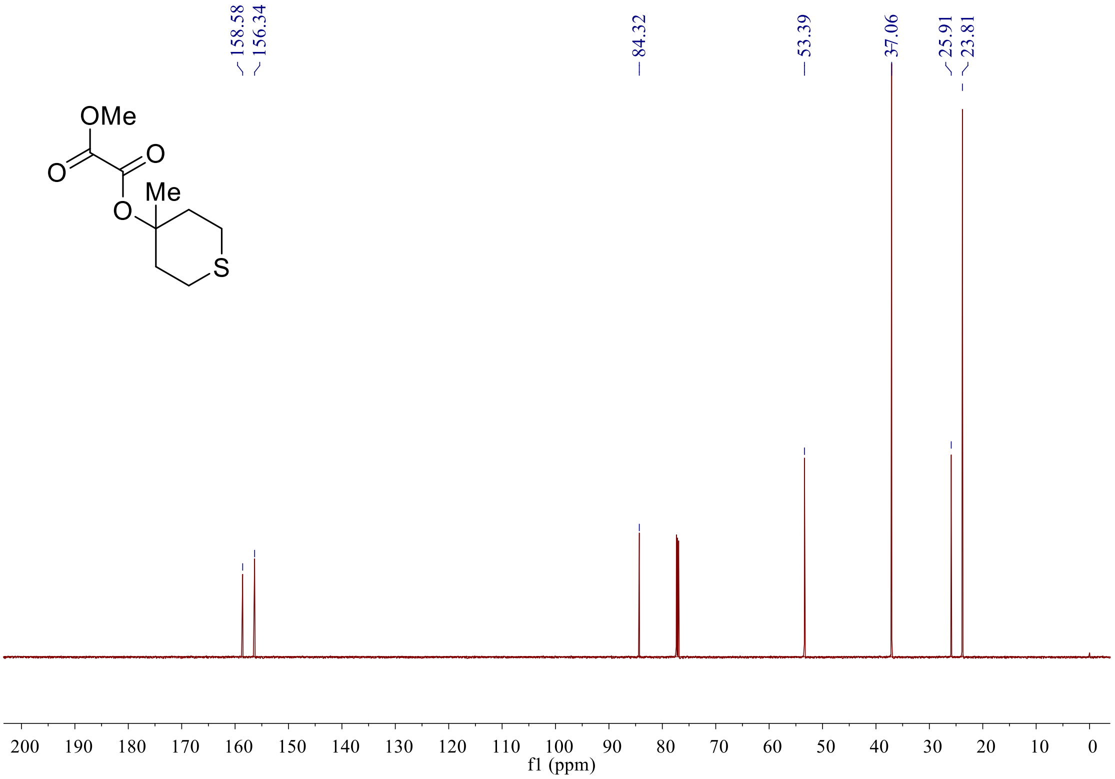

**Cesium 2-((4-methyltetrahydro-2H-pyran-4-yl)oxy)-2-oxoacetate (S18):**

$^{1}$H NMR (600 MHz, DMSO)

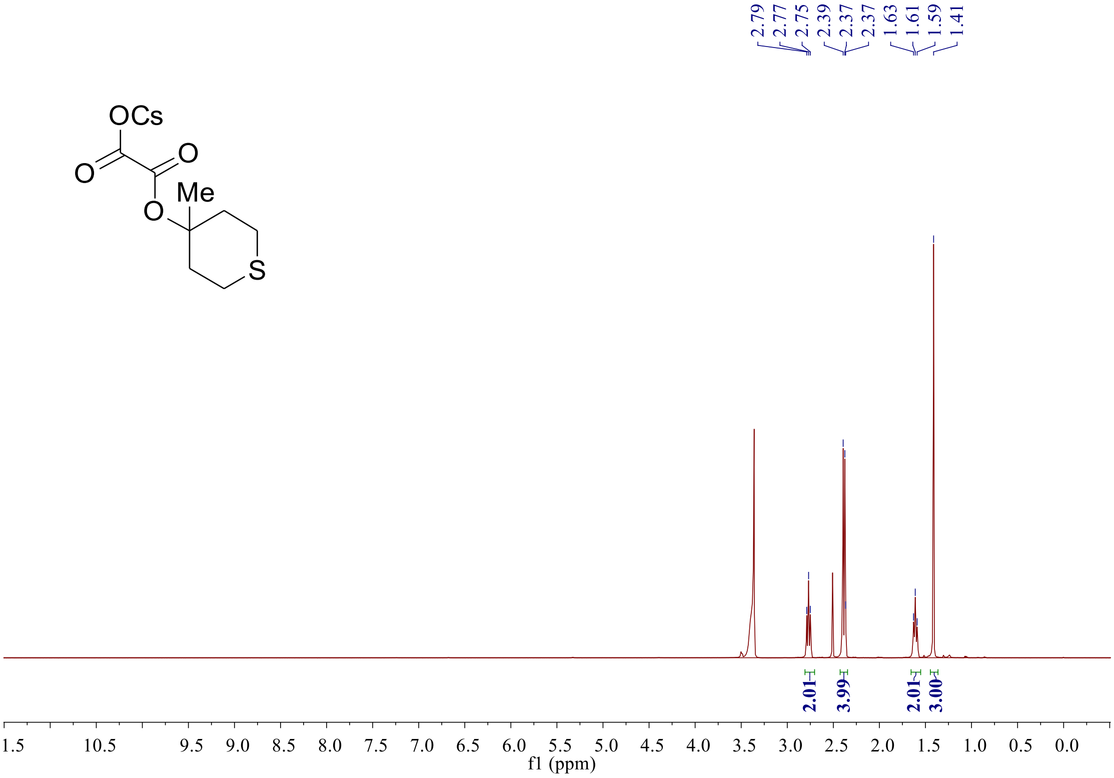


[3]C NMR (150 MHz, DMSO)

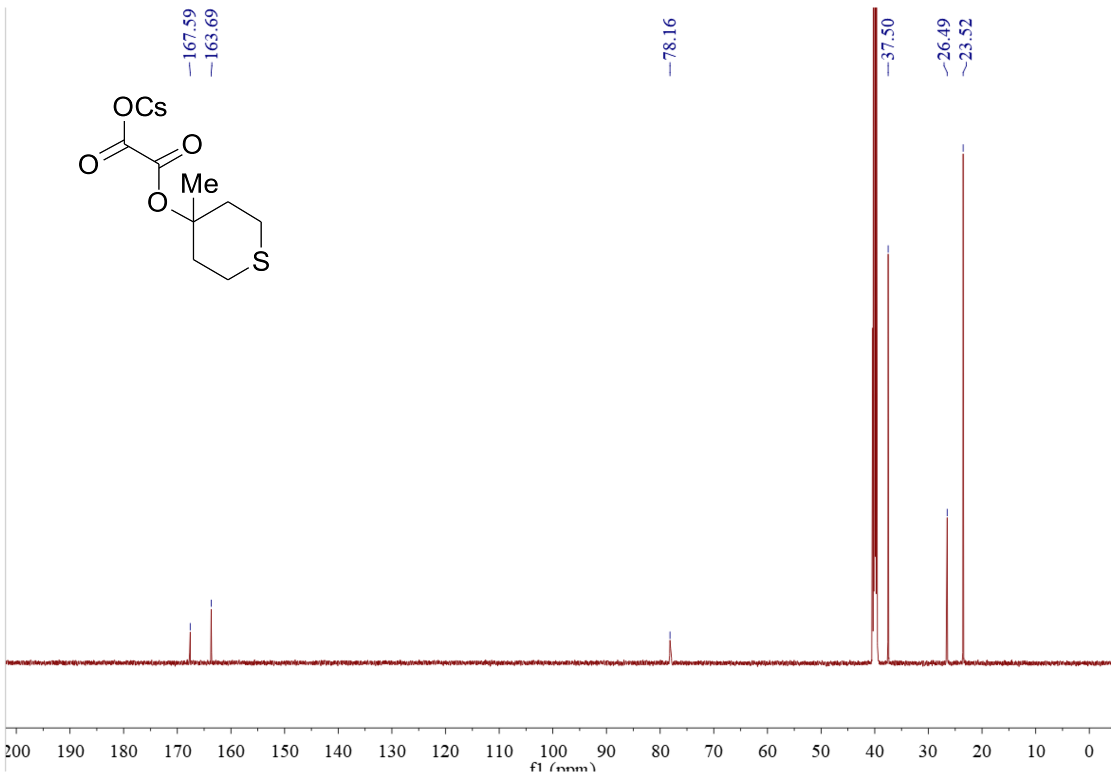

**Methyl (7-methyl-1,4-dioxaspiro[4.5]decan-7-yl) oxalate (S19):**

$^{1}$H NMR (600 MHz, $CDCl_3$)

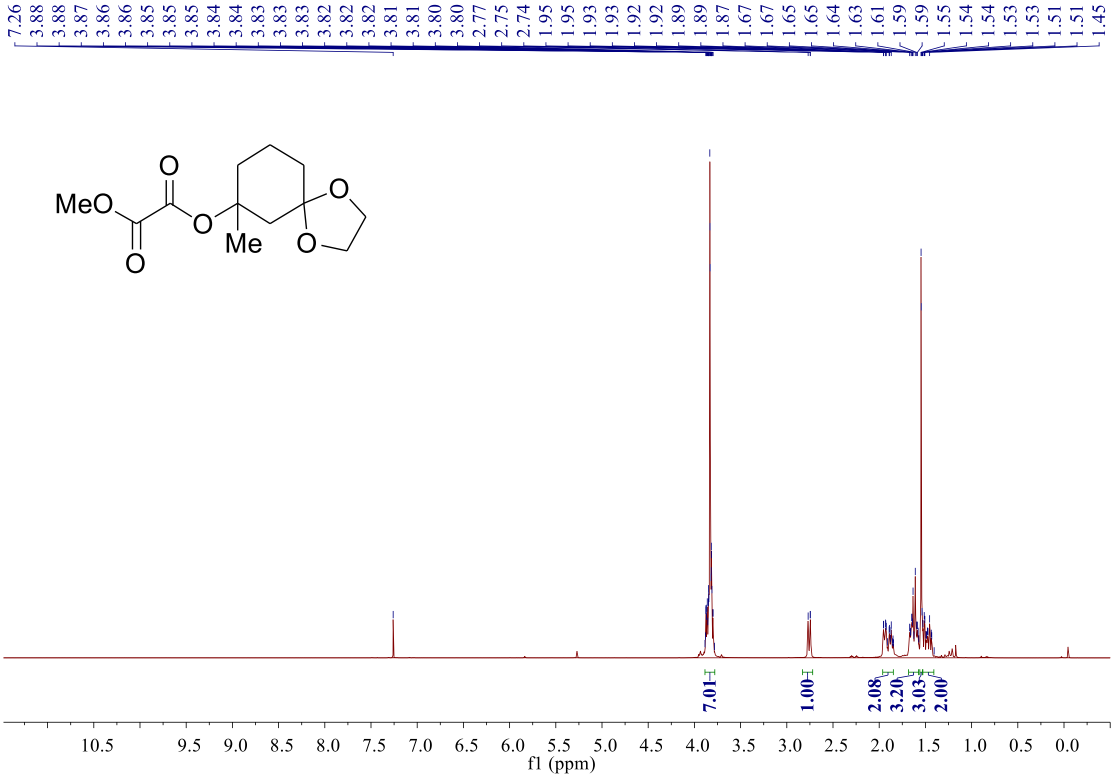


$^{13}$C NMR (150 MHz, $CDCl_3$)

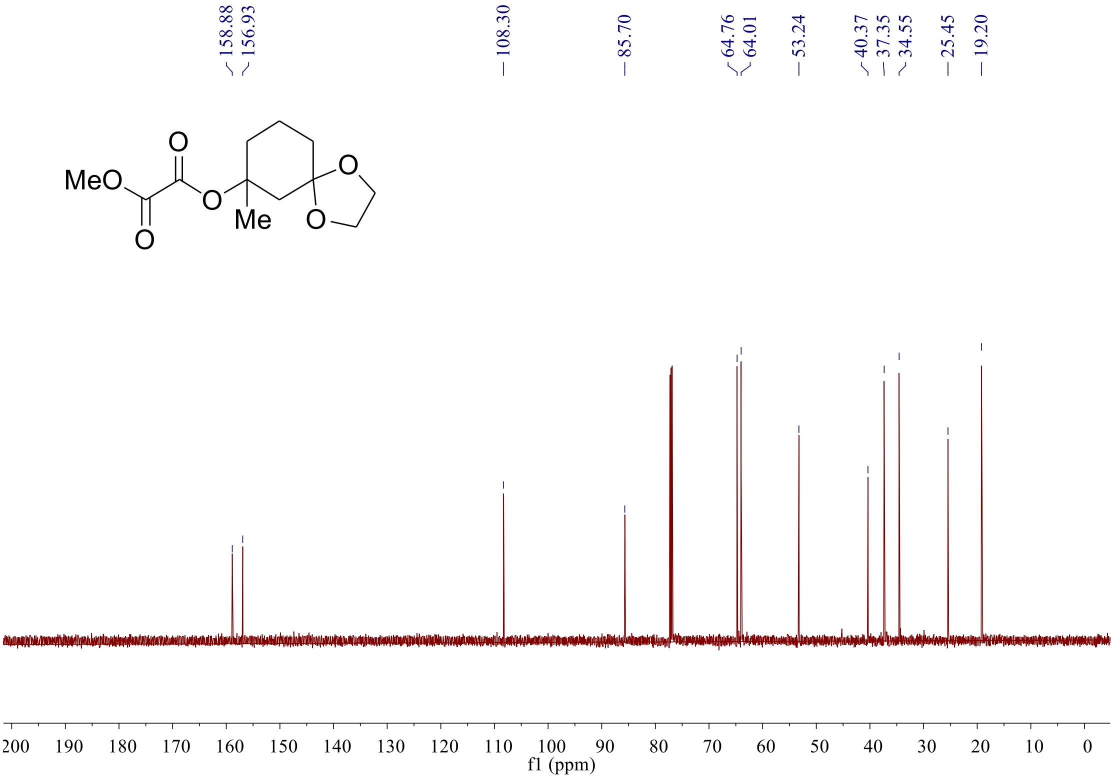

**Cesium 2-((7-methyl-1,4-dioxaspiro[4.5]decan-7-yl)oxy)-2-oxoacetate (S20):**

$^{1}$H NMR (600 MHz, DMSO)

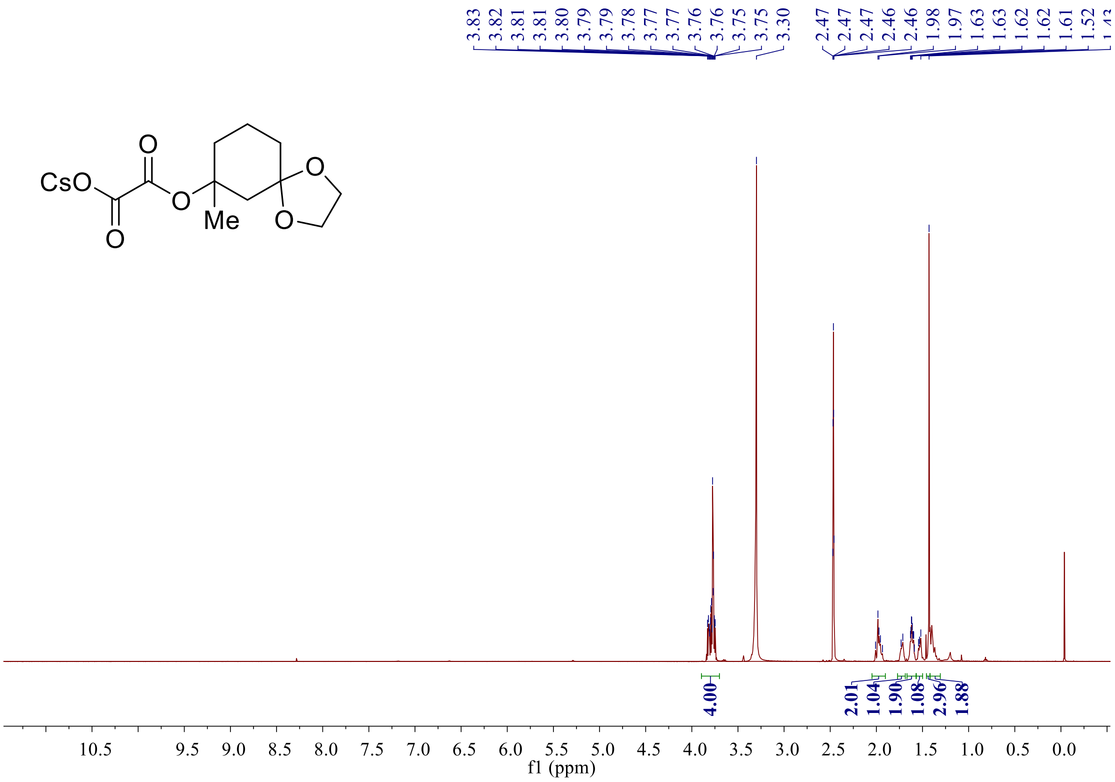


$^{13}$C NMR (150 MHz, DMSO)

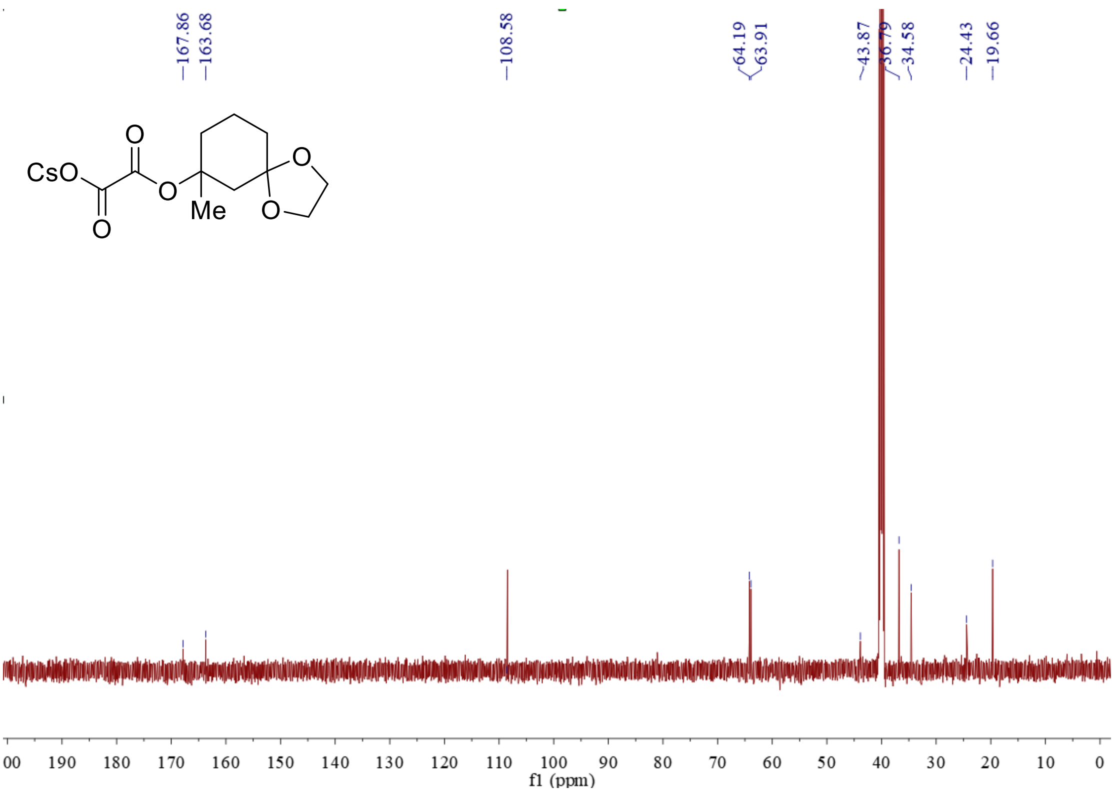

**Methyl (1-methyl-4-methylenecyclohexyl) oxalate(S21).**

$^1$H NMR (600 MHz, CDCl3)

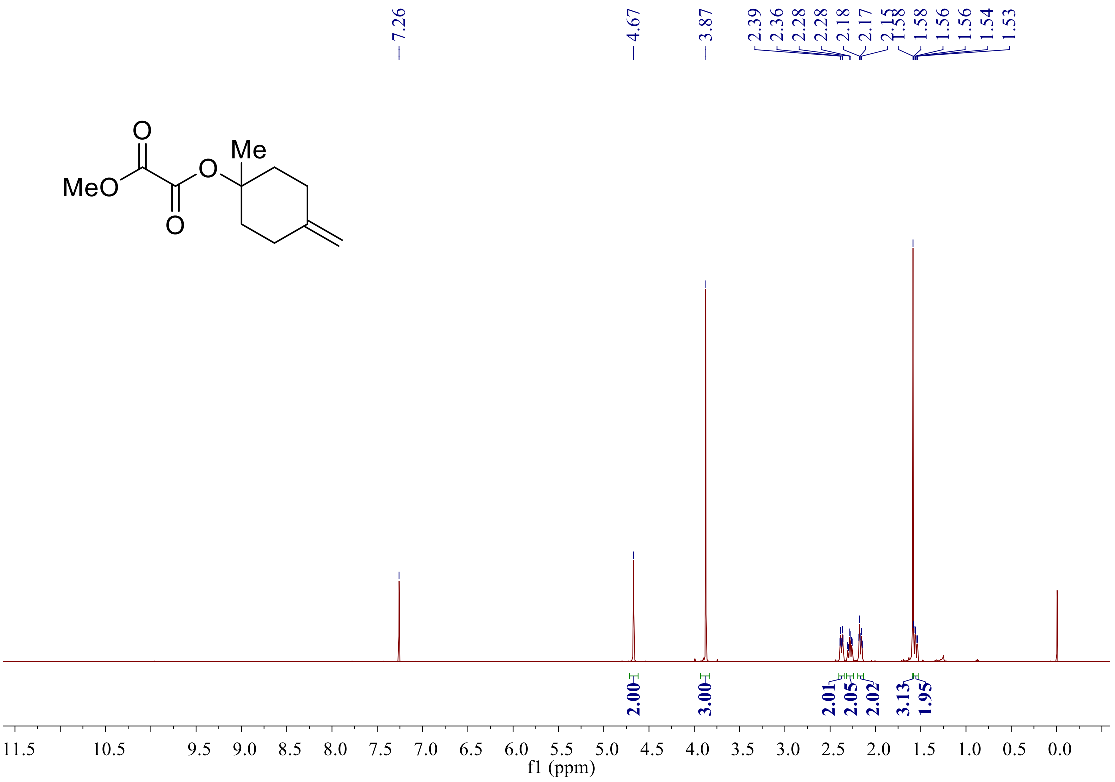


$^{13}$C NMR (150 MHz, $CDCl_3$)

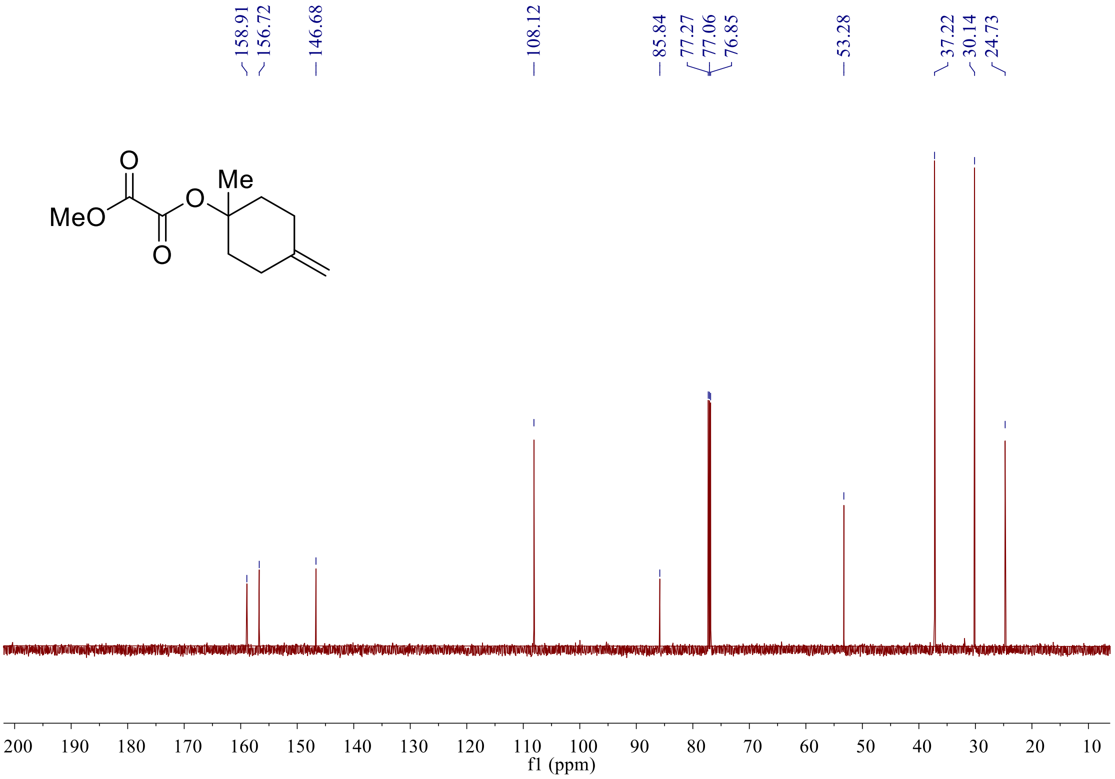

**Cesium 2-((1-methyl-4-methylenecyclohexyl)oxy)-2-oxoacetate(S22)**

$^{1}$H NMR (400 MHz, DMSO)

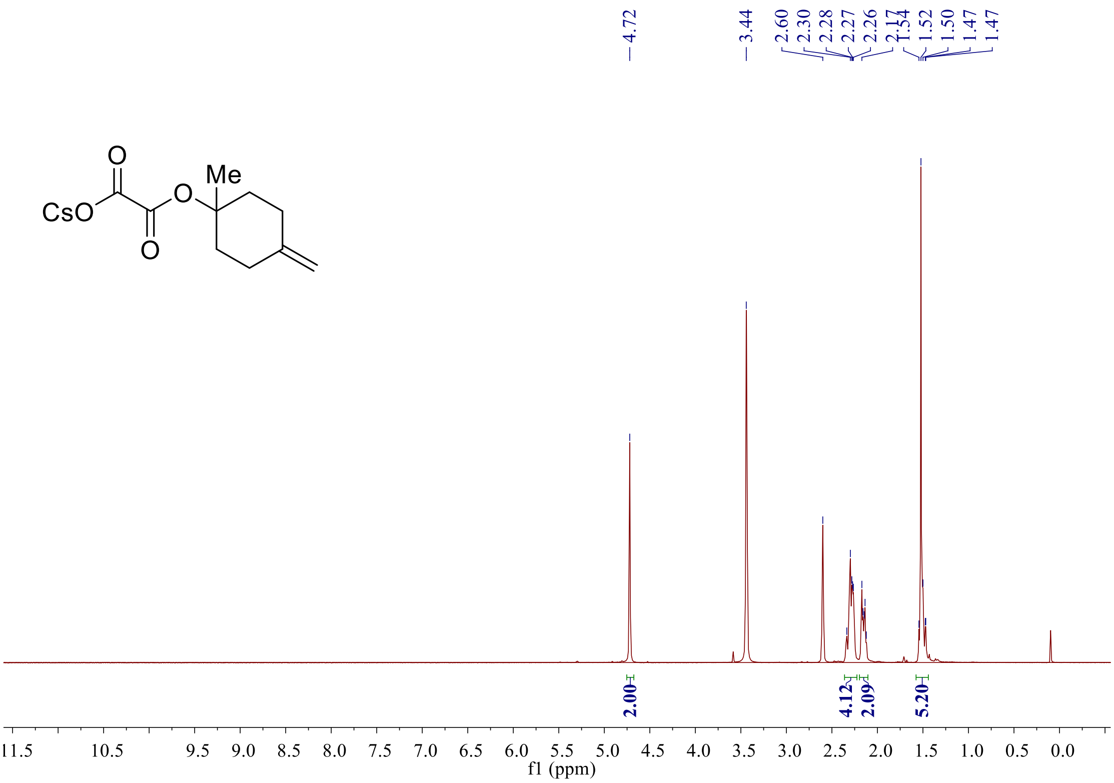

$^{13}$C NMR (100 MHz, DMSO)

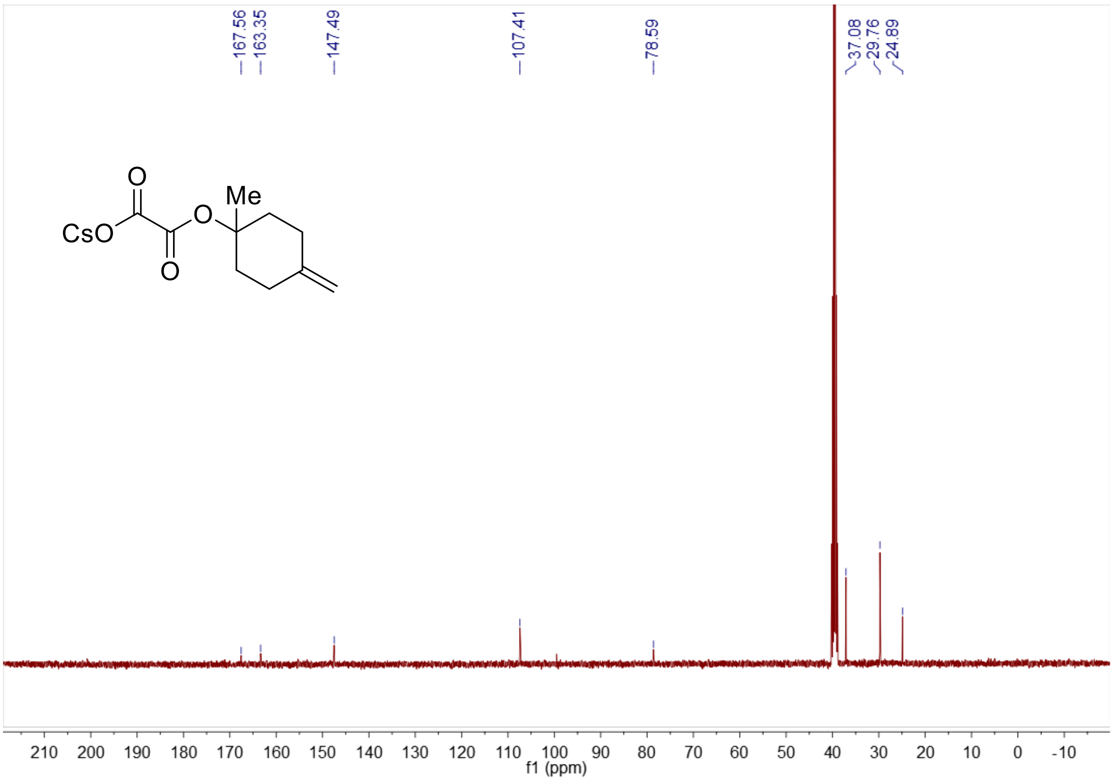

**(E)-3-(3-(4-cyanophenyl)-1-methylcyclohexyl)allyl benzoate (4):**

$^{1}$H NMR (600 MHz, $CDCl_3$)

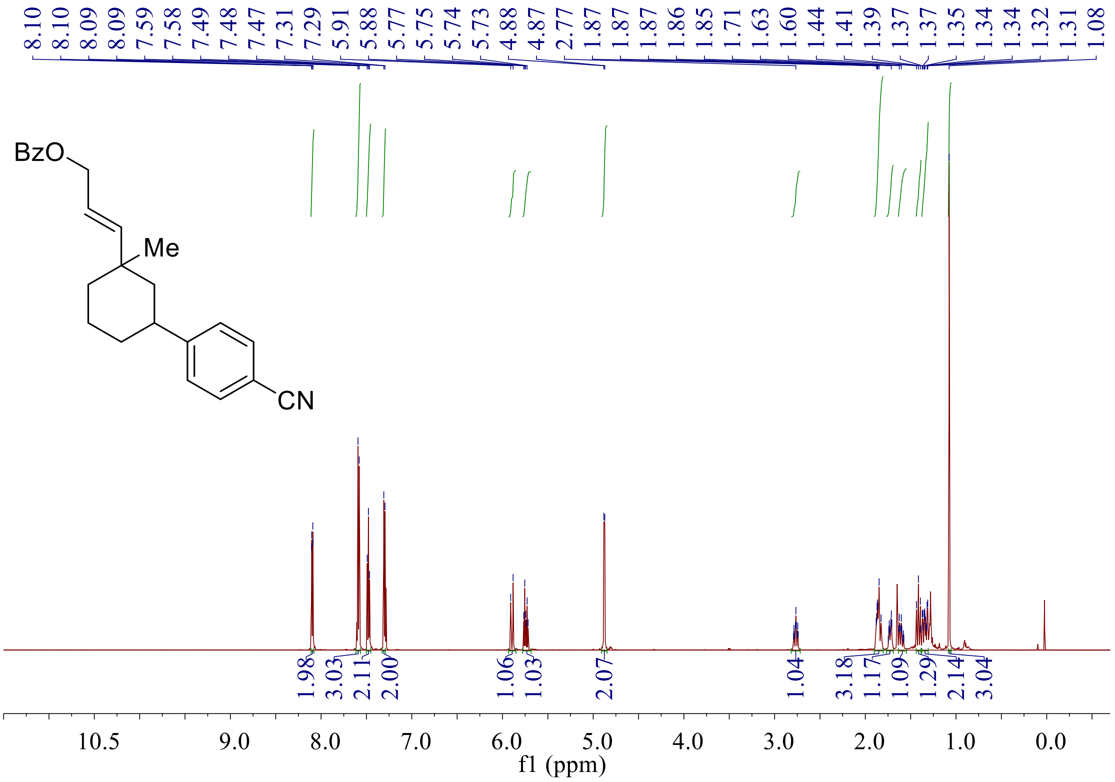


$^{13}$C NMR (150 MHz, $CDCl_3$)

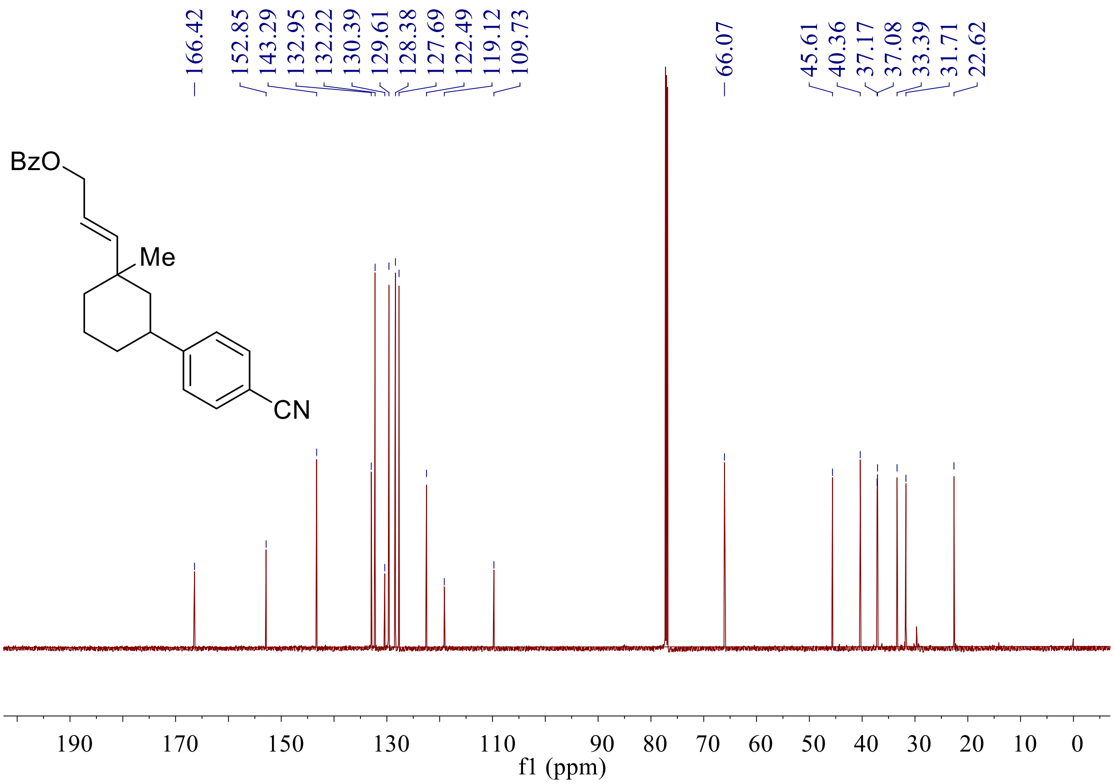

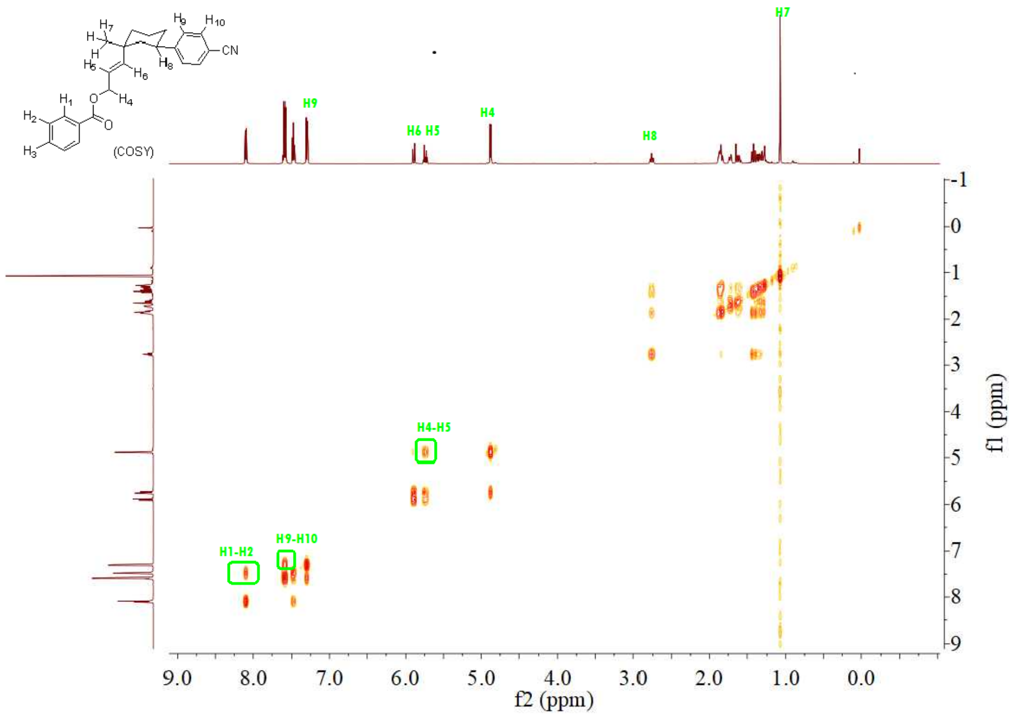


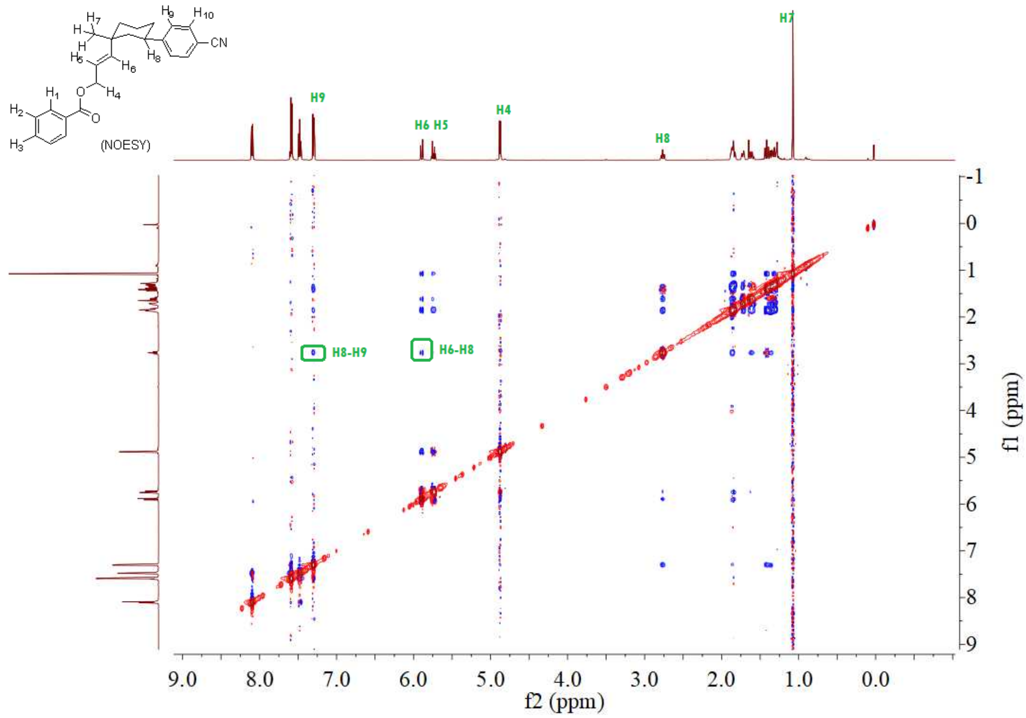


**(E)-3-(6-(4-cyanophenyl)-8-methyl-1,4-dioxaspiro[4.5]decan-8-yl)allyl benzoate (5):**

$^1$H NMR (600 MHz, $CDCl_3$)

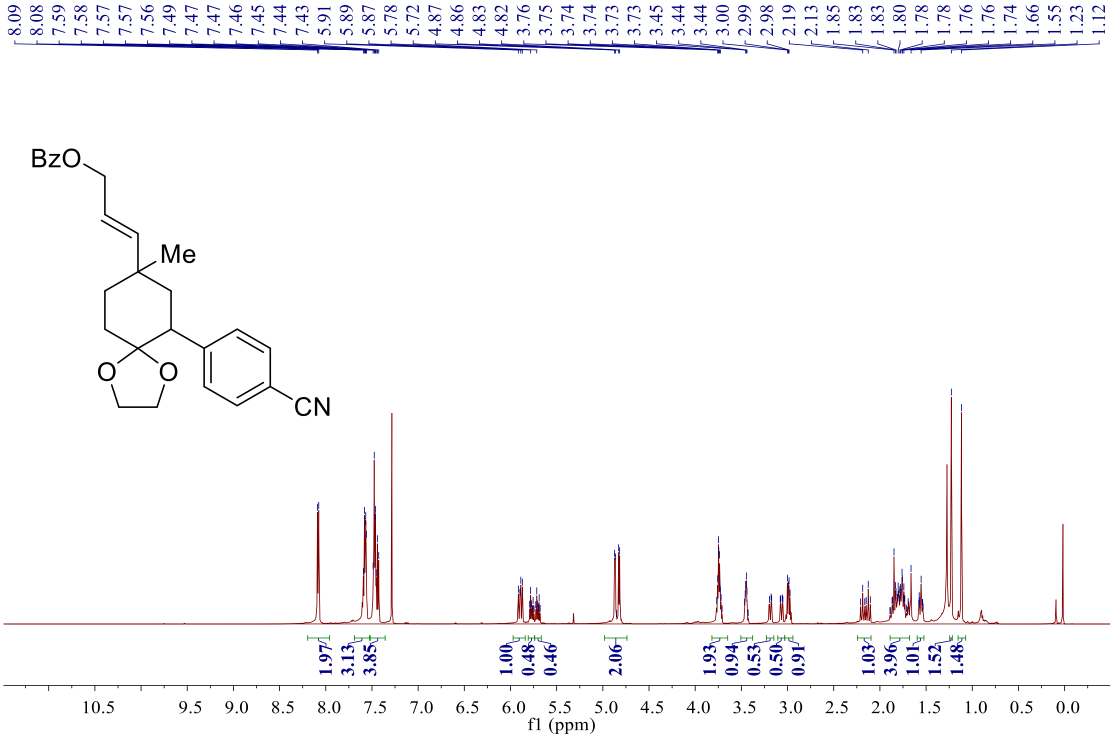


$^{13}C$ NMR (150 MHz, $CDCl_3$)

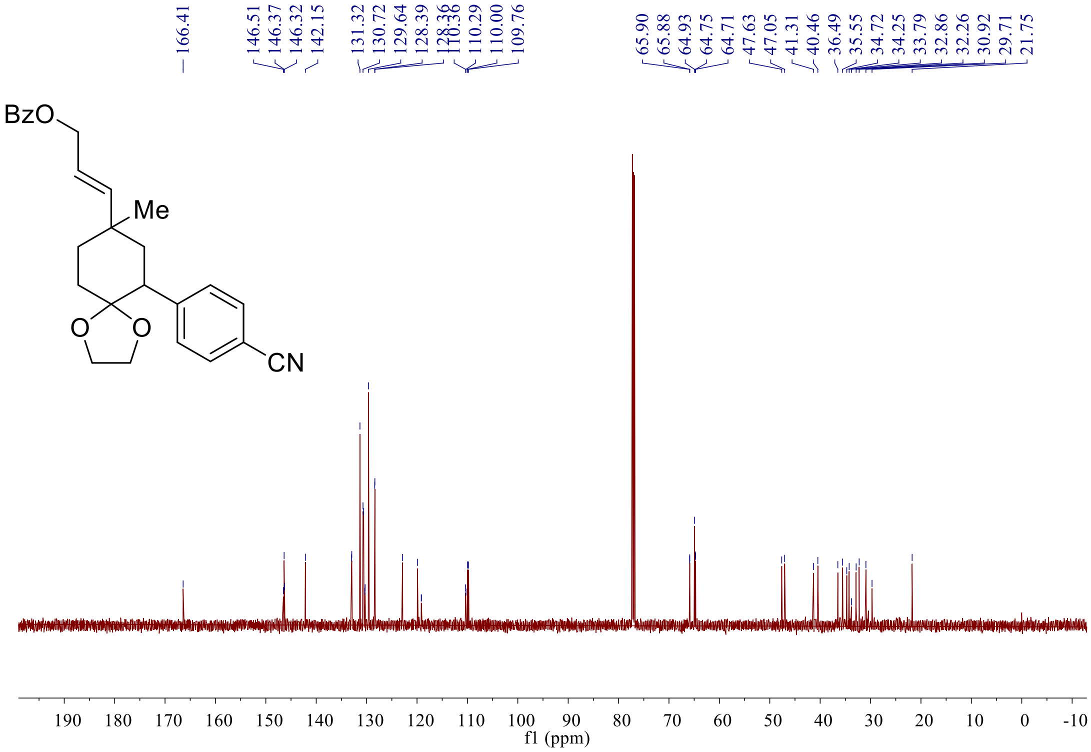


**(E)-3-(5-(4-cyanophenyl)-1,2-dimethylcyclohexyl)allyl benzoate(6)**

$^{1}H$ NMR (600 MHz, $CDCl_3$)

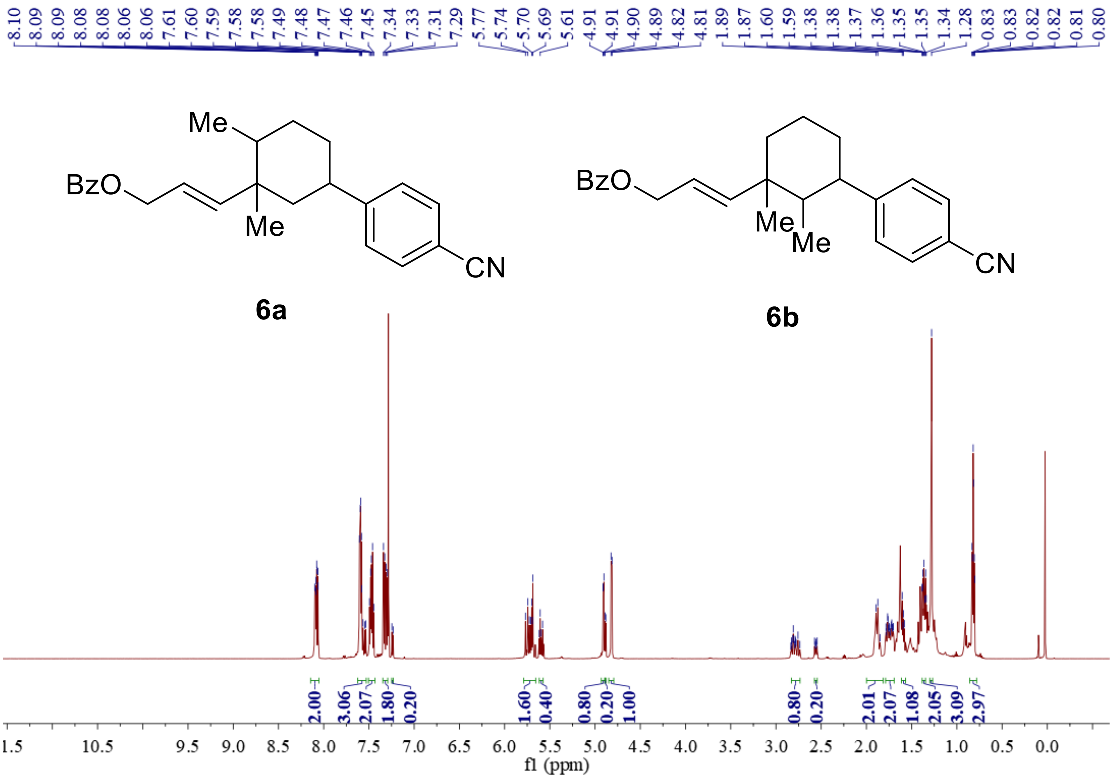

$^{13}$C NMR (150 MHz, CDCl$_3$)

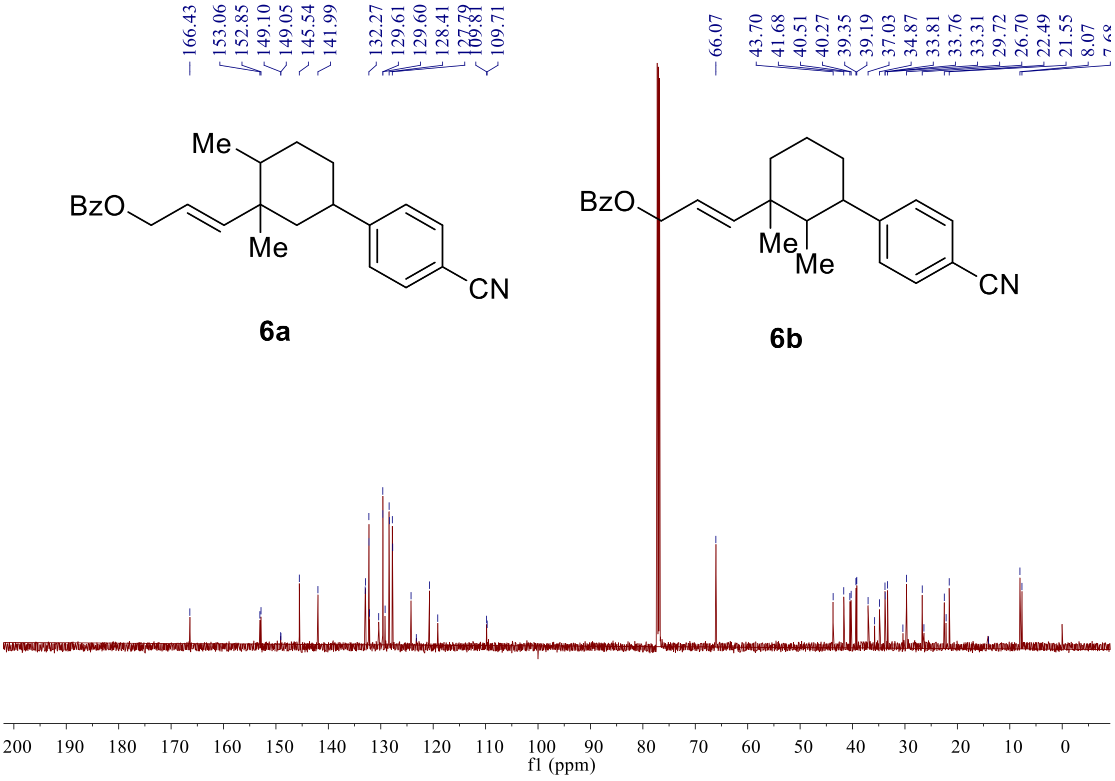

**(E)-3-(3-(4-cyanophenyl)-1,4-dimethylcyclohexyl)allyl benzoate(7)**

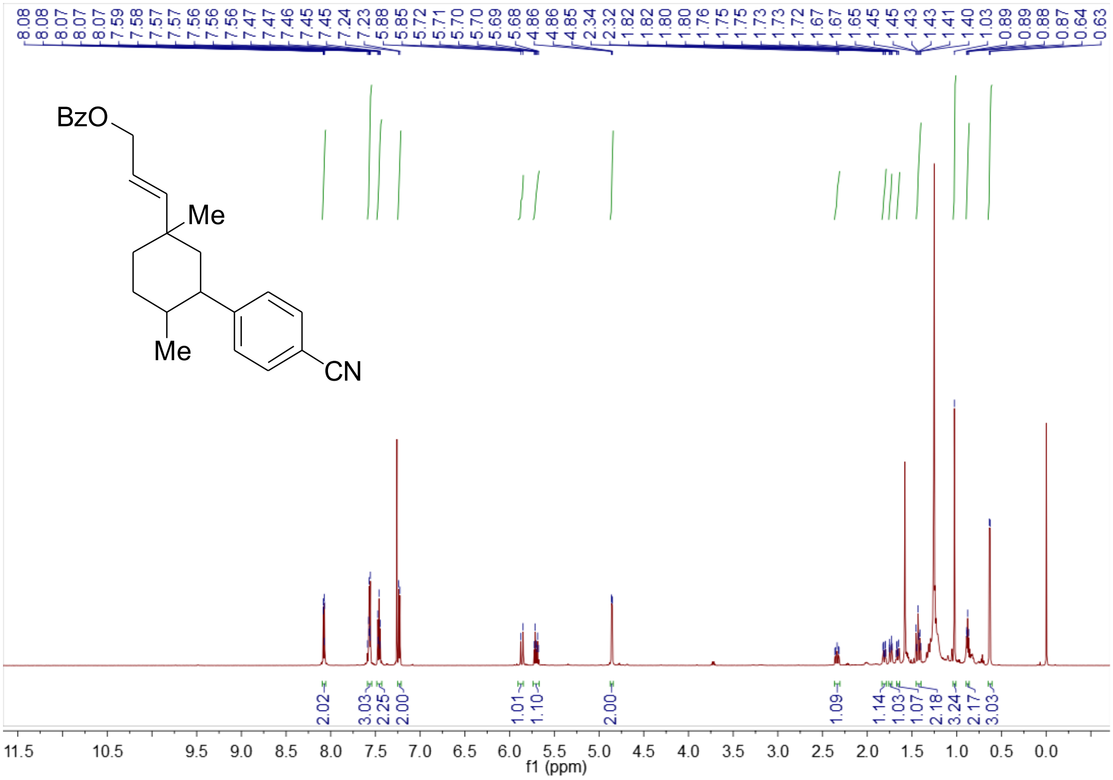
8.08
8.08
8.07
8.07
7.59
7.58
7.57
7.57
7.56
7.56
7.56
7.47
7.47
7.46
7.45
7.45
7.24
7.23
5.88
5.85
5.72
5.71
5.70
5.70
5.69
5.68
4.86
4.86
4.85
2.34
2.32
1.82
1.82
1.80
1.80
1.76
1.75
1.75
1.73
1.73
1.72
1.67
1.67
1.65
1.45
1.45
1.43
1.43
1.41
1.40
1.03
0.89
0.89
0.88
0.87
0.64
0.63
BzO
Me
Me
CN
2.02
3.03
2.25
2.00
1.01
1.10
2.00
1.09
1.14
1.03
1.07
2.18
3.24
2.17
3.03
f1 (ppm)

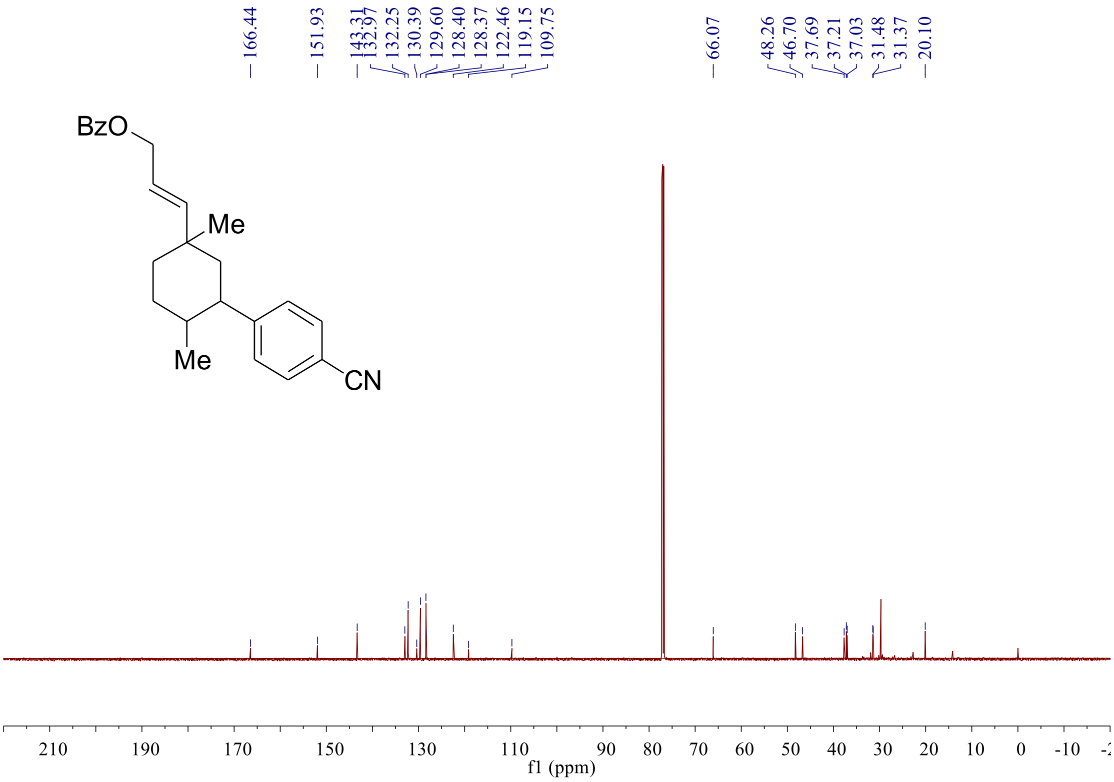
166.44
151.93
143.31
132.97
132.25
130.39
129.60
128.40
128.37
122.46
119.15
109.75
66.07
48.26
46.70
37.69
37.21
37.03
31.48
31.37
20.10
BzO
Me
Me
CN
f1 (ppm)

**3-(1,4-dimethylcyclohexyl)allyl benzoate (7’)**

$^{1}H$ NMR (600 MHz, $CDCl_3$)

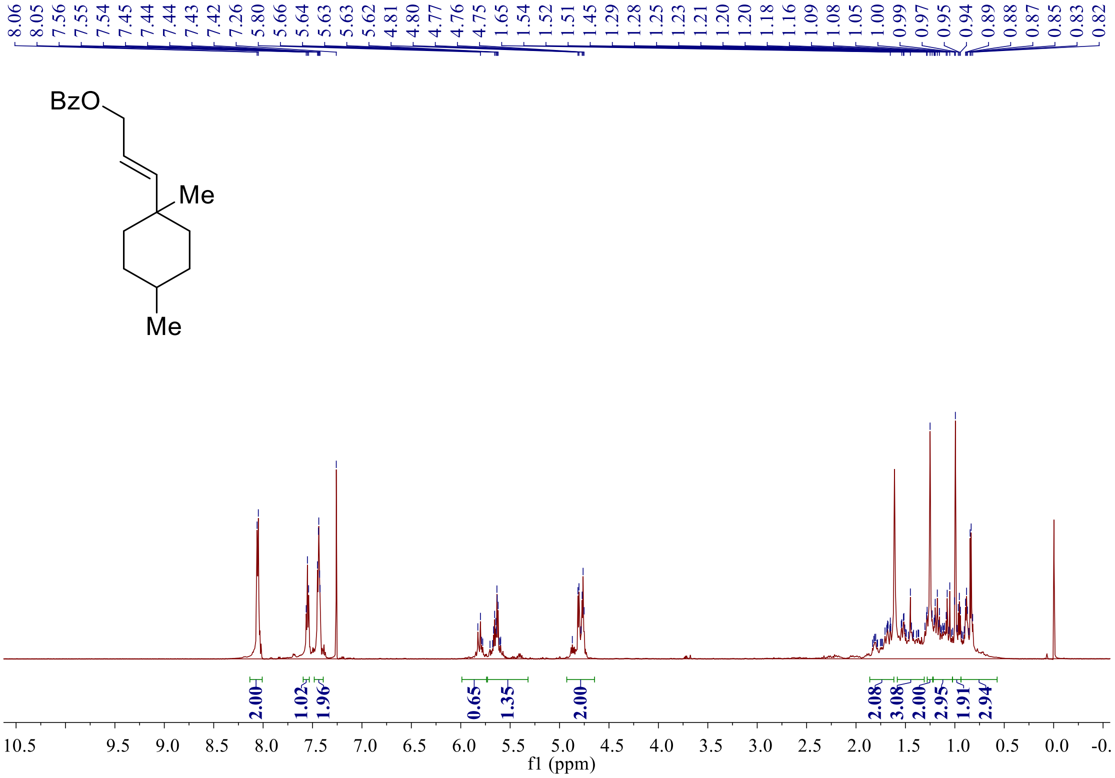


$^{13}C$ NMR (150 MHz, $CDCl_3$)

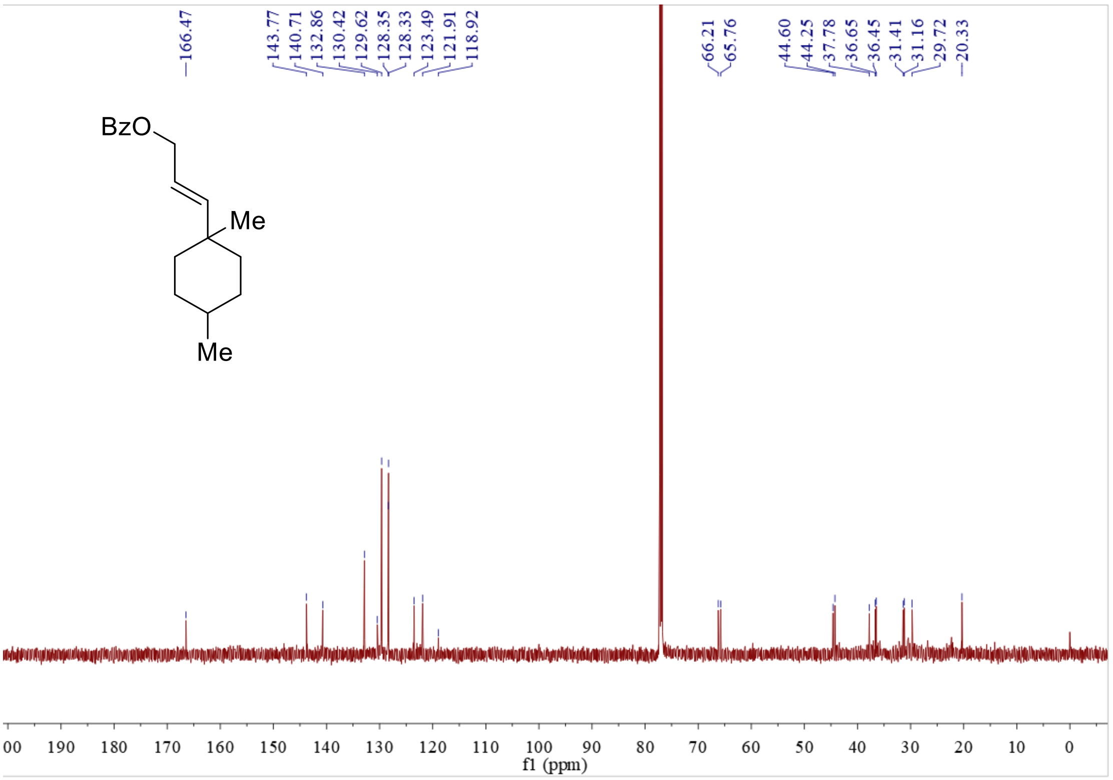

**(E)-3-(3-(4-cyanophenyl)-1-ethylcyclohexyl)allyl benzoate (8)**

$^{1}$H NMR (600 MHz, $CDCl_3$)

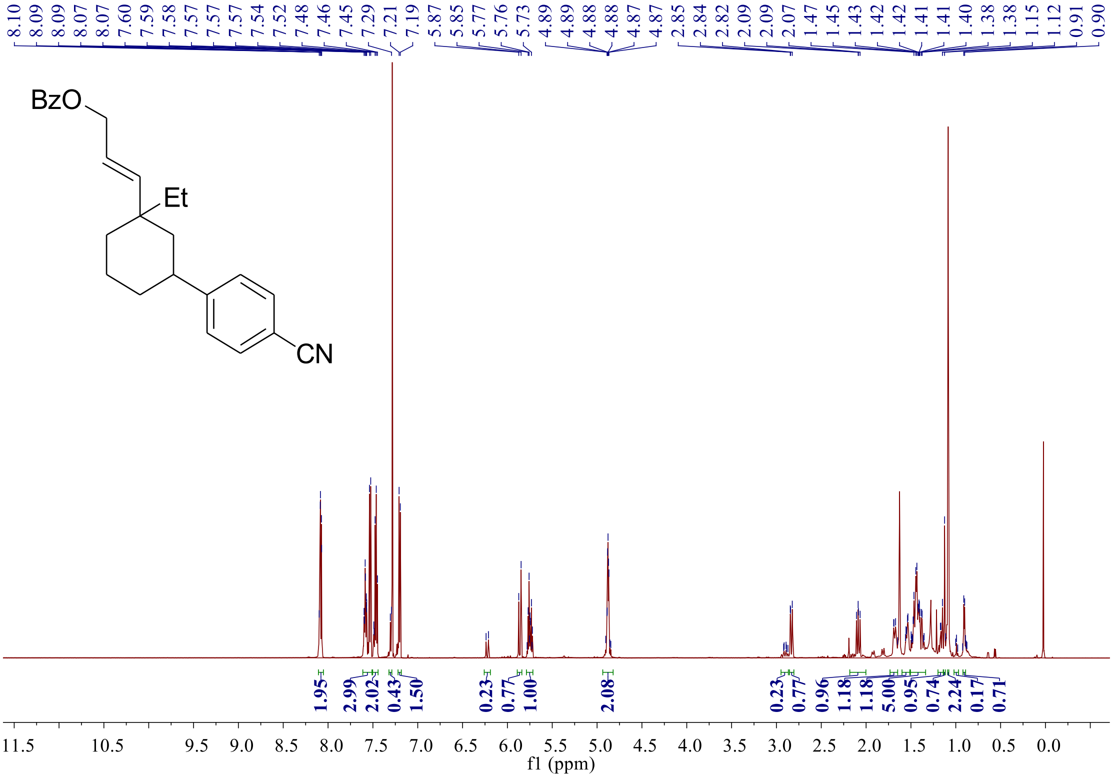


$^{13}$C NMR (150 MHz, $CDCl_3$)

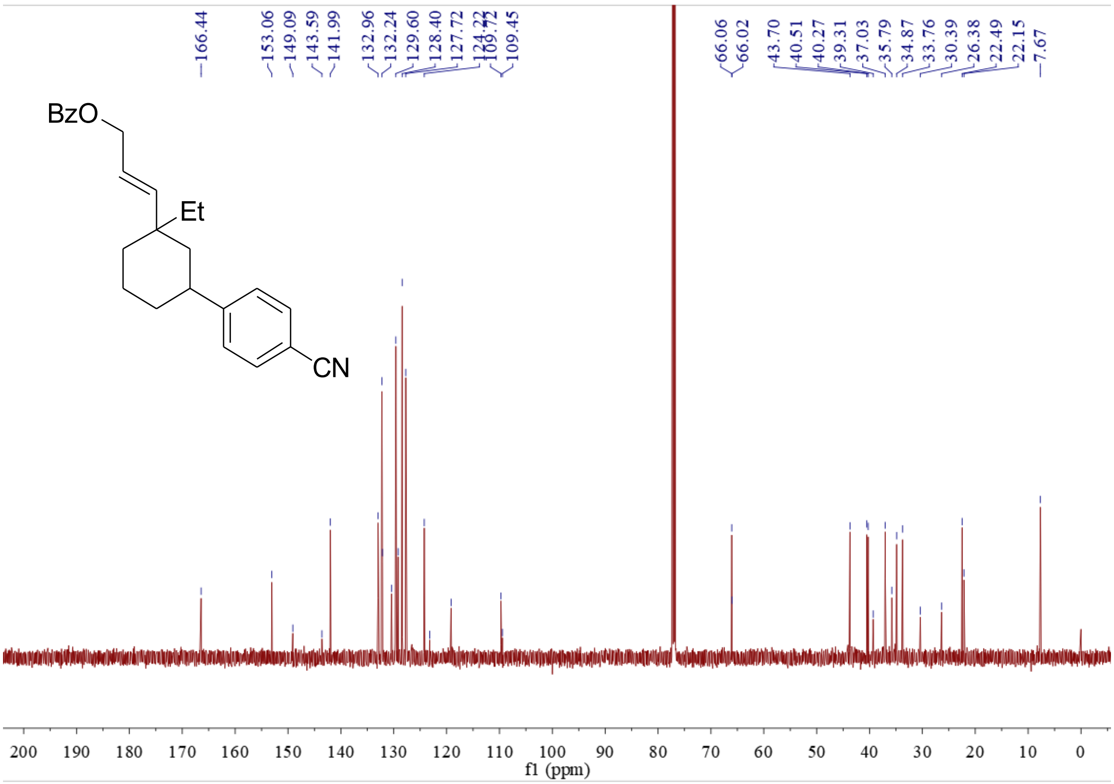

**(*E*)-3-(9-(4-cyanophenyl)-7-methyl-1,4-dioxaspiro[4.5]decan-7-yl)allyl benzoate(9)**

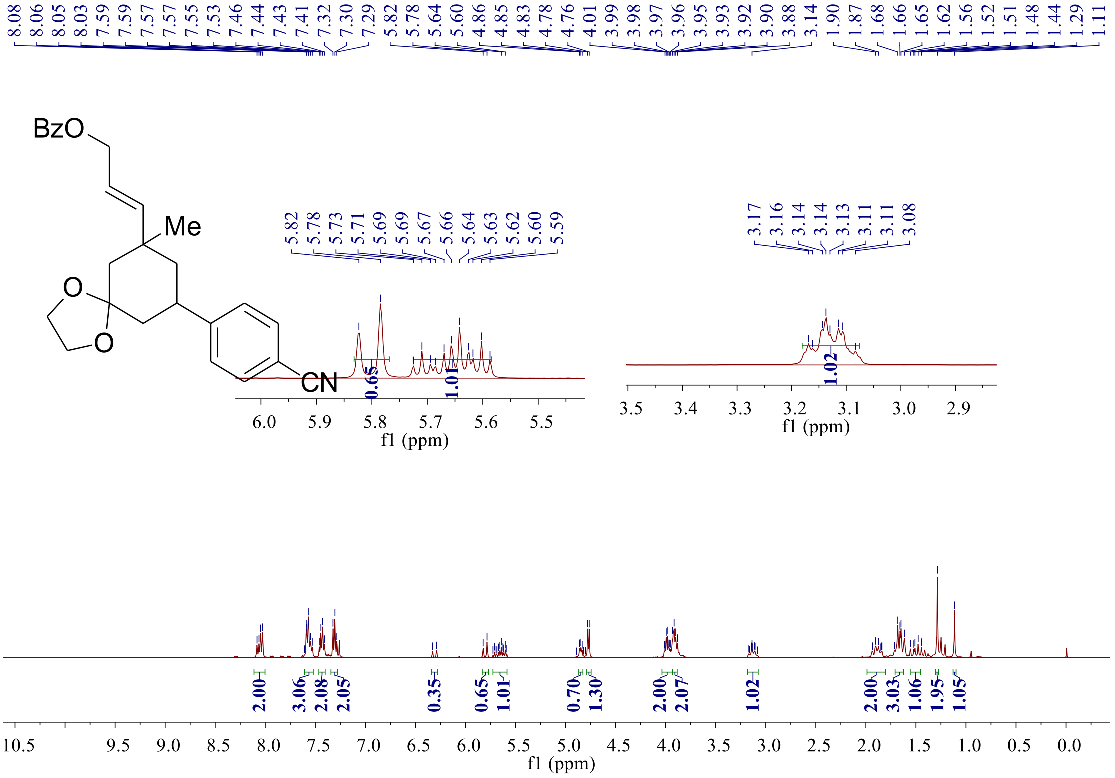


$^{13}$C NMR (150 MHz, $CDCl_3$)

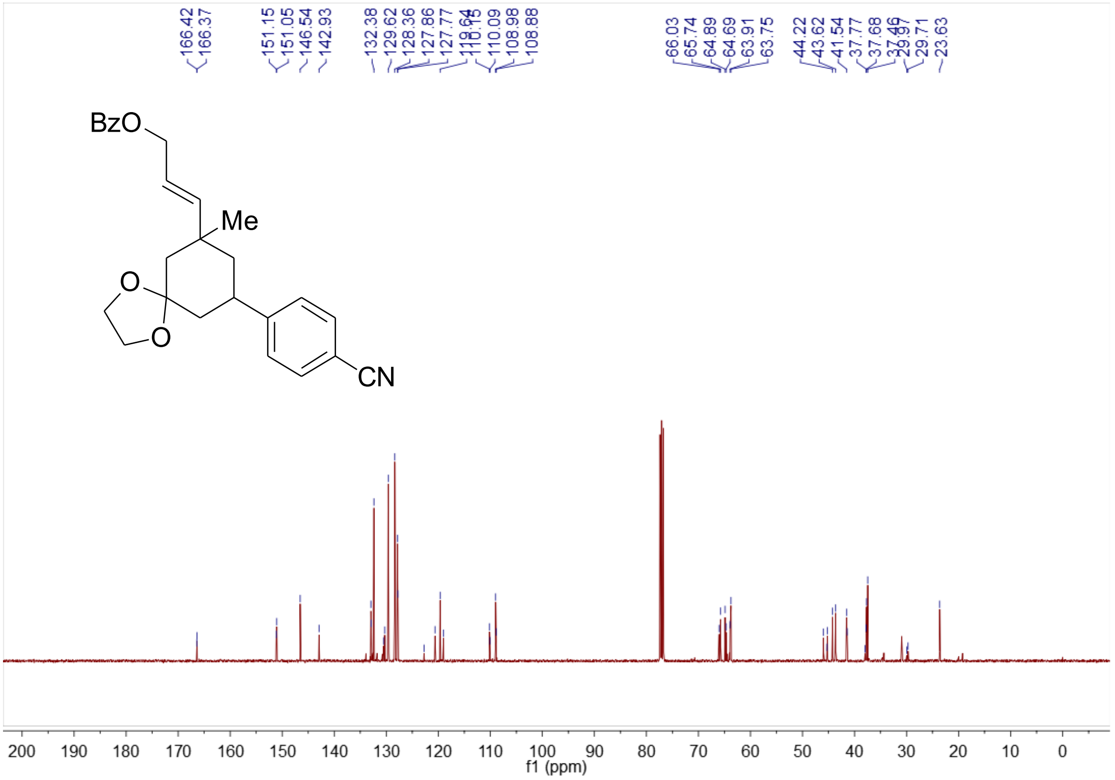

**(*E*)-3-(2-(4-cyanophenyl)-4-methyltetrahydro-2H-pyran-4-yl)allyl benzoate (10)**

$^{1}$H NMR (600 MHz, $CDCl_3$)

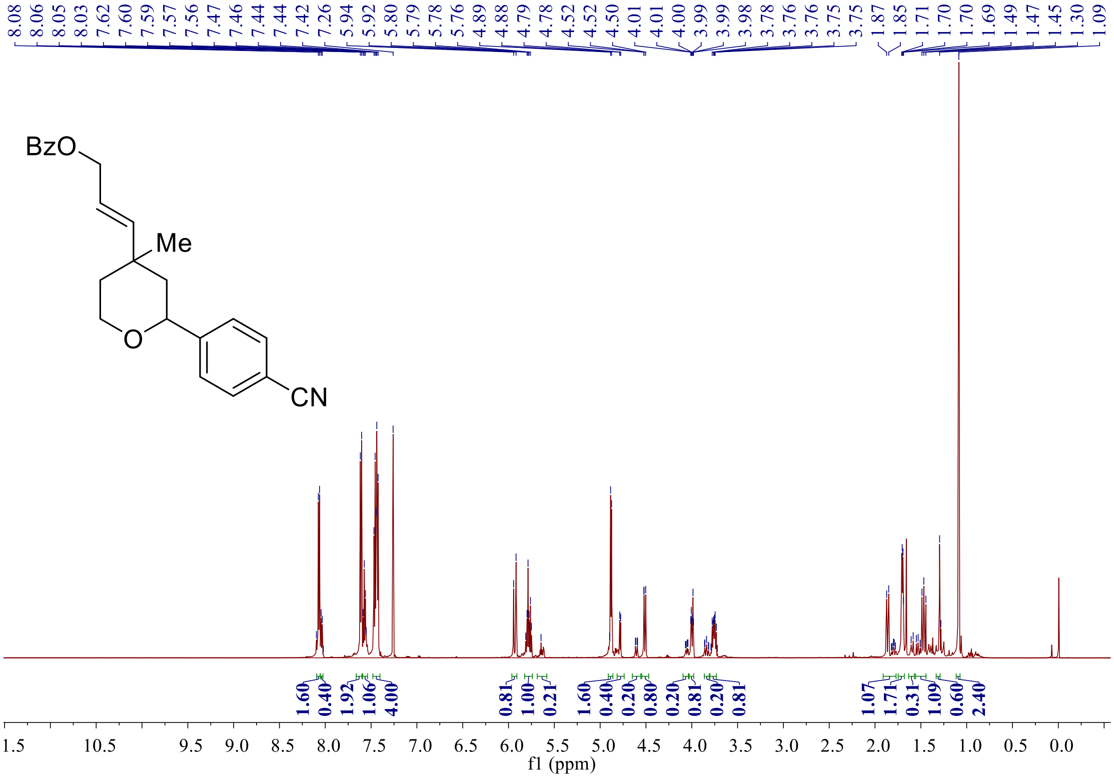


$^{13}$C NMR (150 MHz, $CDCl_3$)

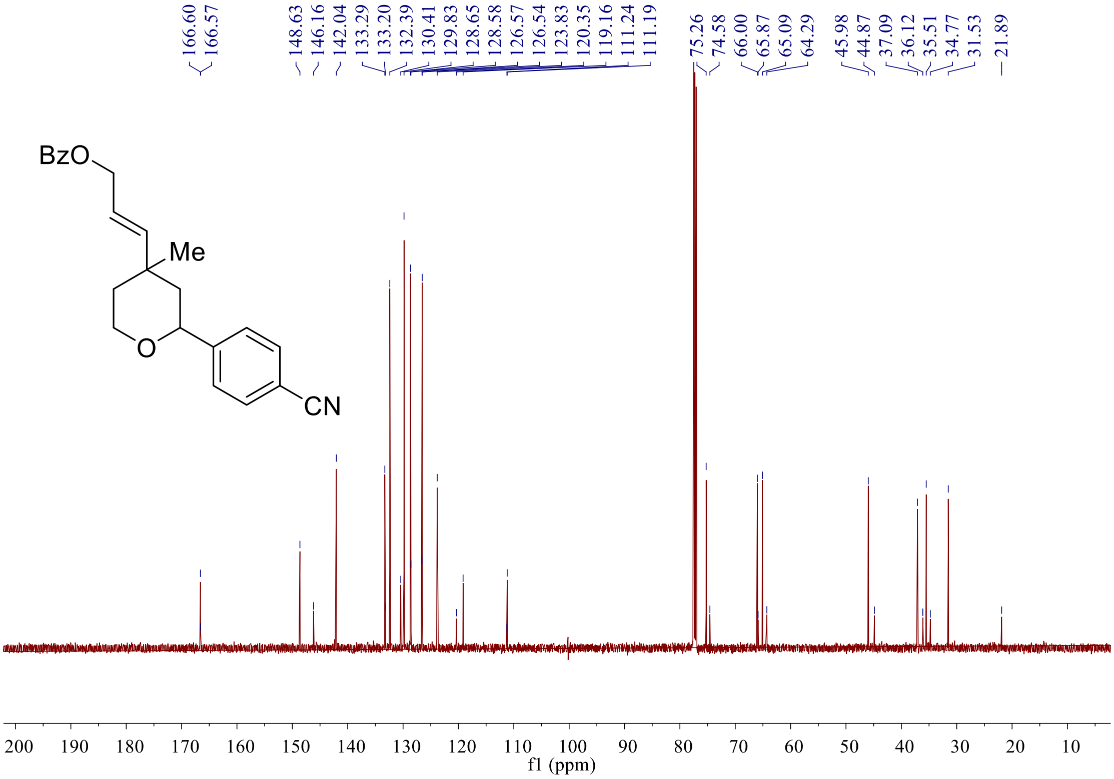

**(*E*)-3-(5-(4-cyanophenyl)-3-methyltetrahydro-2H-pyran-3-yl)allyl benzoate (11)**

$^1$H NMR (400 MHz, $CDCl_3$)

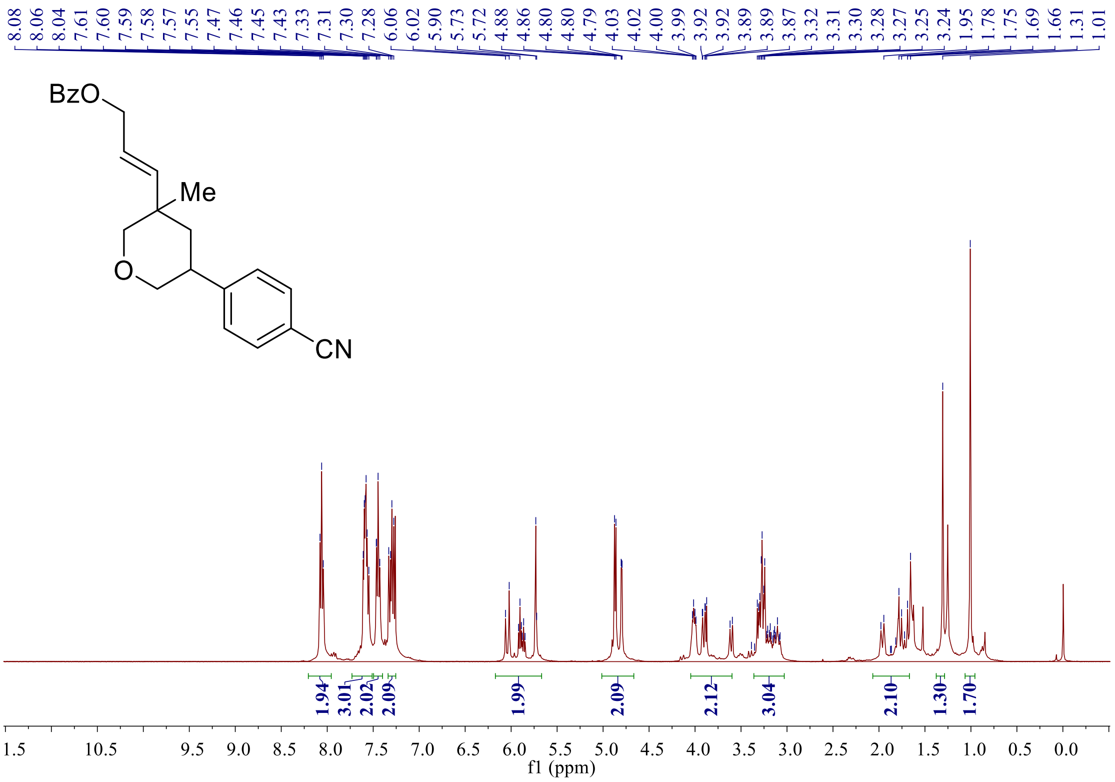

$^{13}$C NMR (100 MHz, $CDCl_3$)

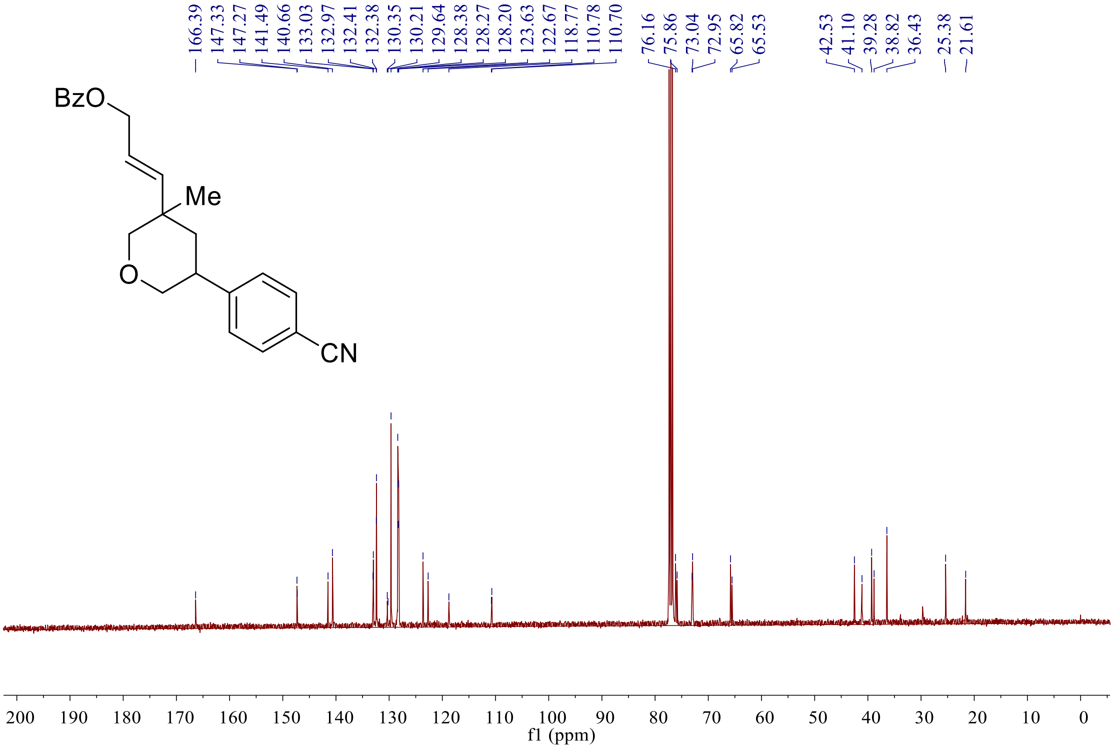

**(*E*)-3-(2-(4-cyanophenyl)-4-methyltetrahydro-2H-thiopyran-4-yl)allyl benzoate(12)**

$^{1}$H NMR (600 MHz, $CDCl_3$)

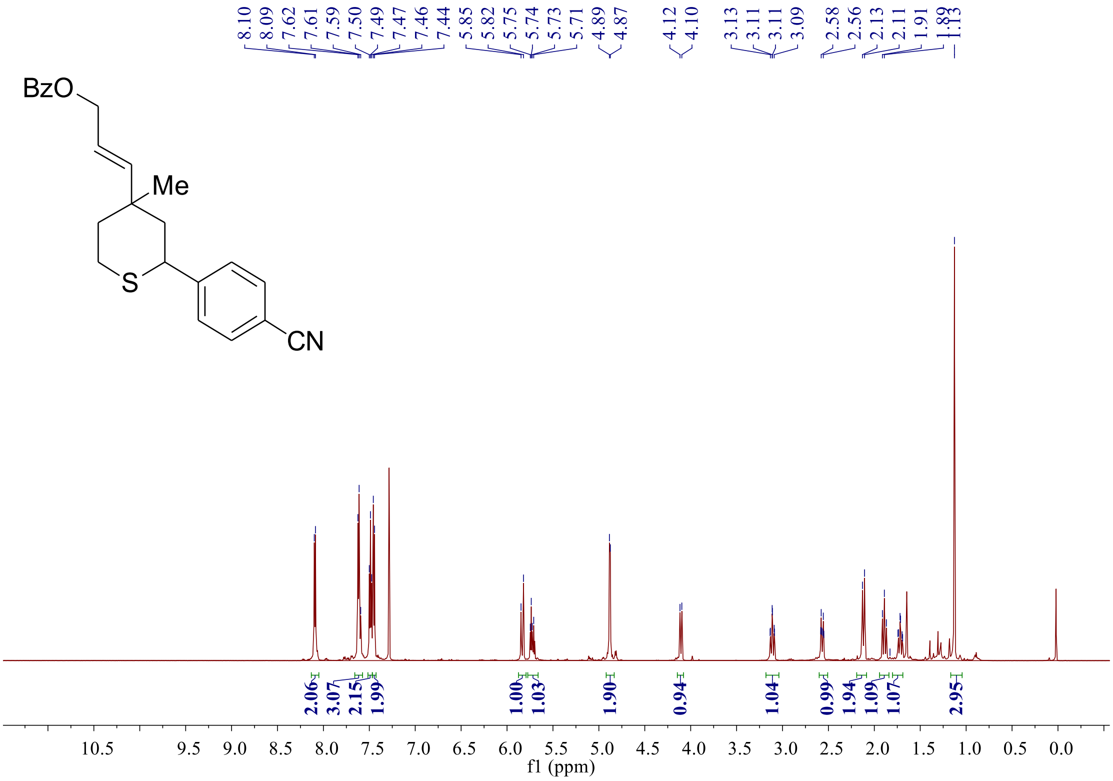


$^{13}$C NMR (150 MHz, $CDCl_3$)

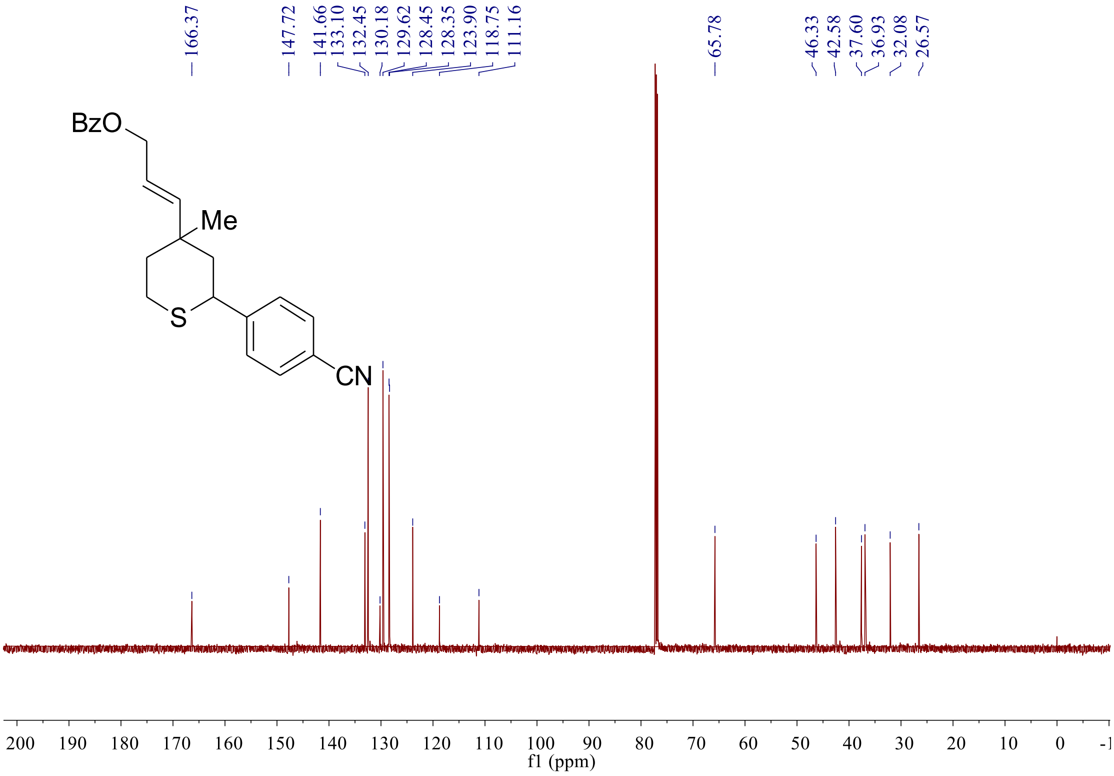

***Tert*-butyl (E)-4-(3-(benzoyloxy)prop-1-en-1-yl)-2-(4-cyanophenyl)-4-ethylpiperidine-1-carboxylate (13)**

$^{1}H$ NMR (600 MHz, $CDCl_3$)

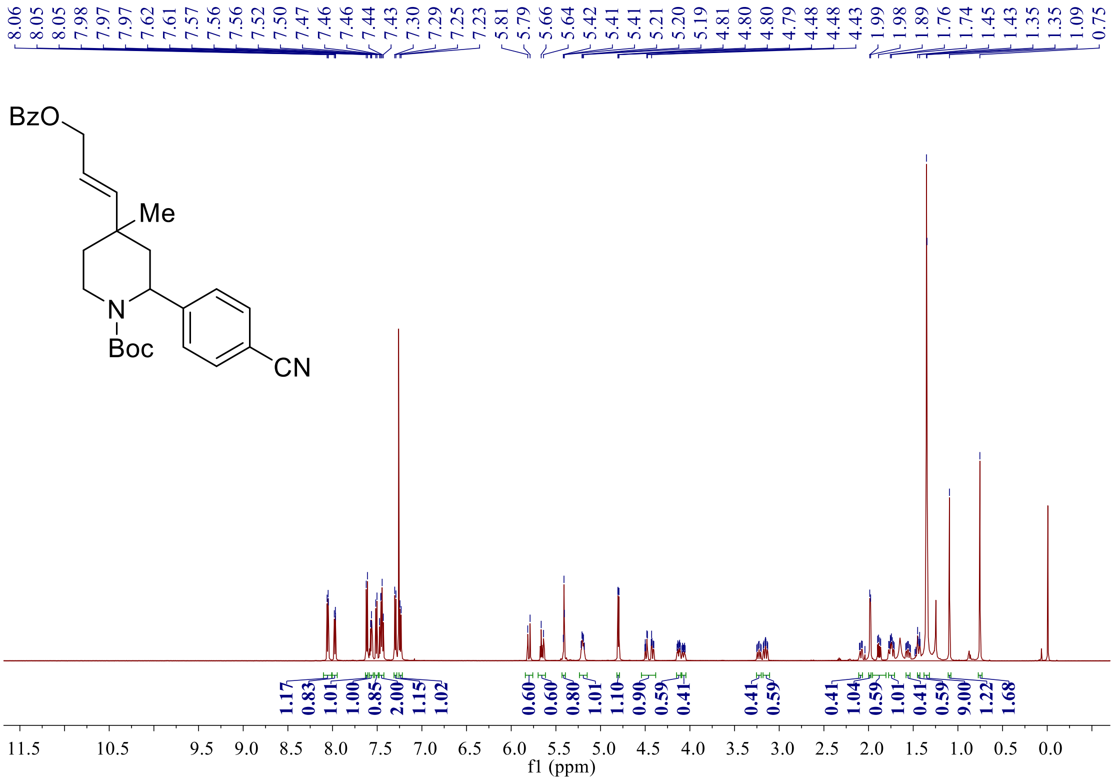


$^{13}C$ NMR (150MHz, $CDCl_3$)

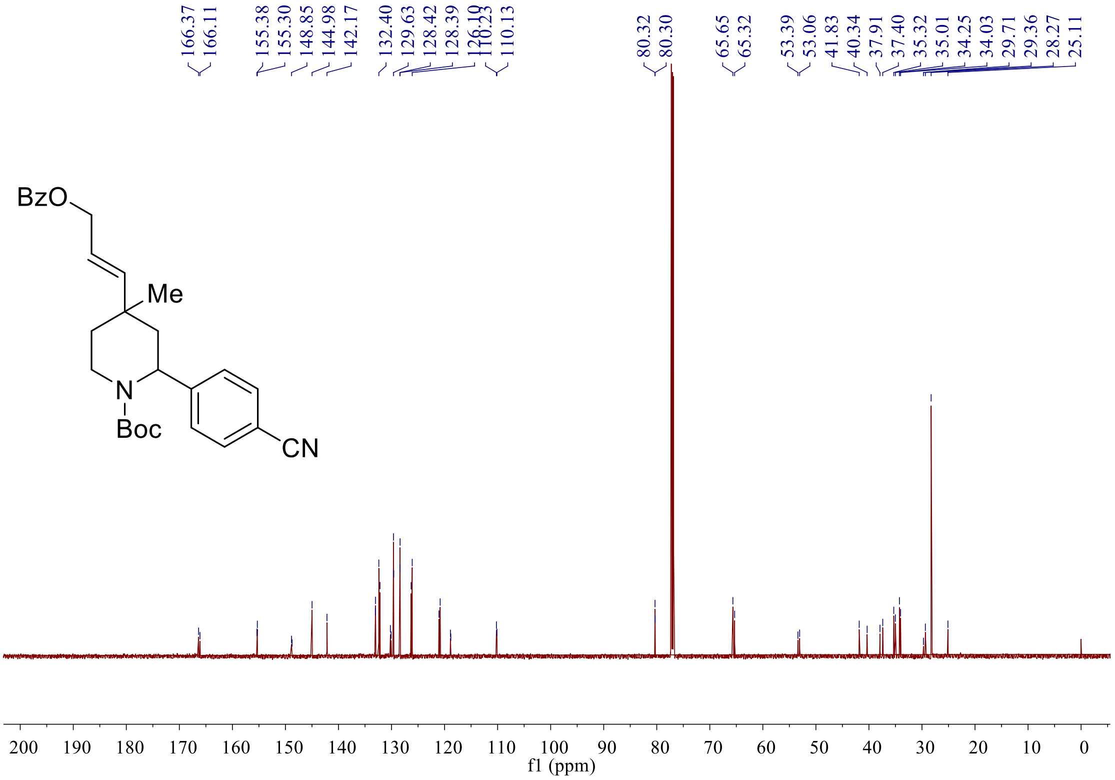

**(*E*)-3-(3-(4-cyanophenyl)-1-methylcyclopentyl)allyl benzoate(14)**

$^1$H NMR (600 MHz, $CDCl_3$)

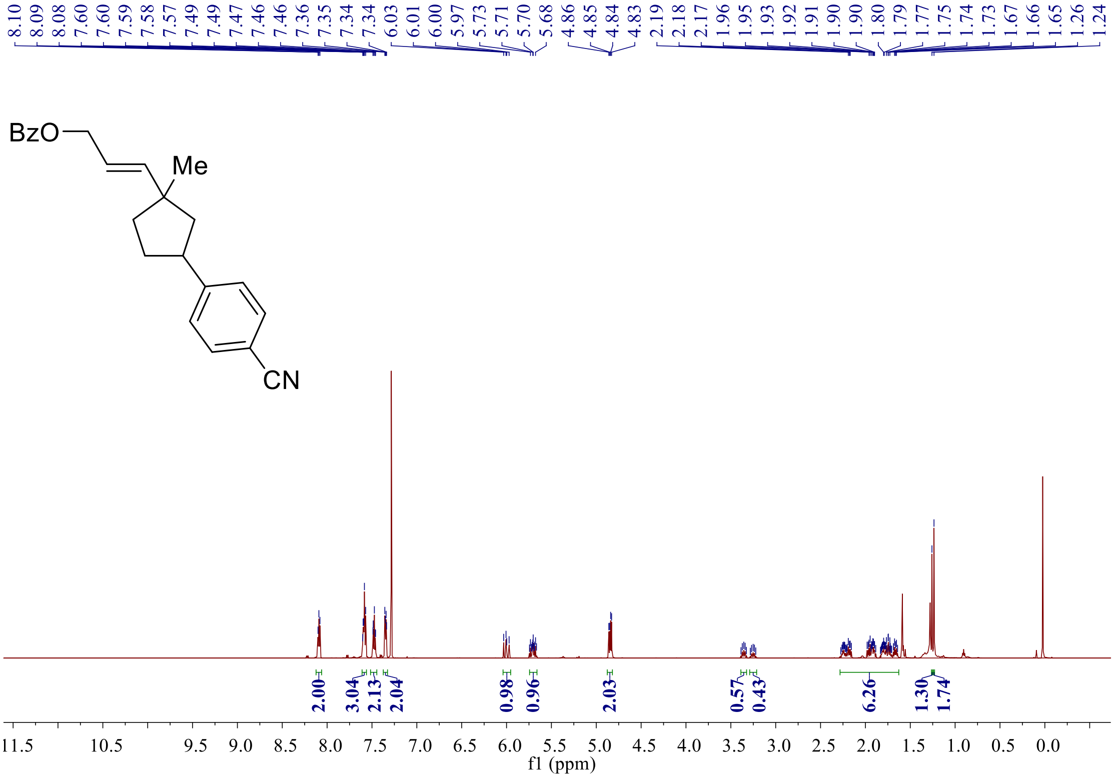


$^{13}$C NMR (150 MHz, $CDCl_3$)

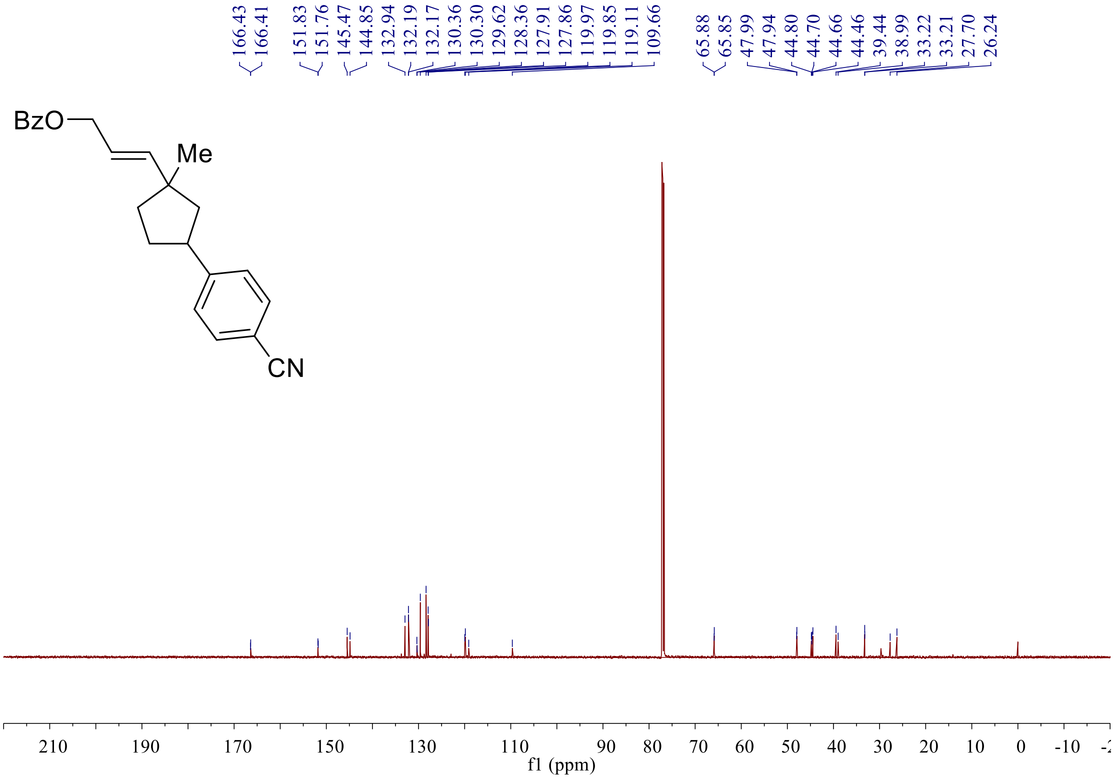

**Methyl (*E*)-4-((4-(3-(benzoyloxy)prop-1-en-1-yl)-4-methylcyclohex-1-en-1-yl)methyl)benzoate(15)**

$^1$H NMR (400 MHz, $CDCl_3$)

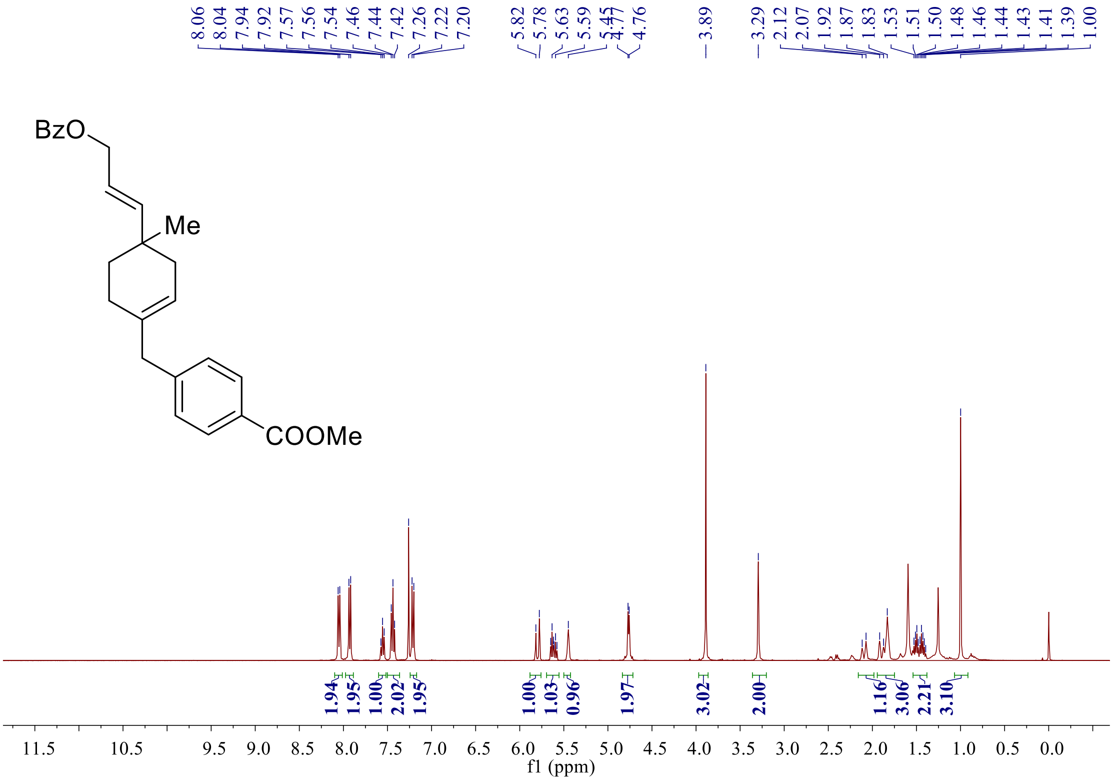


$^{13}$C NMR (100 MHz, $CDCl_3$)

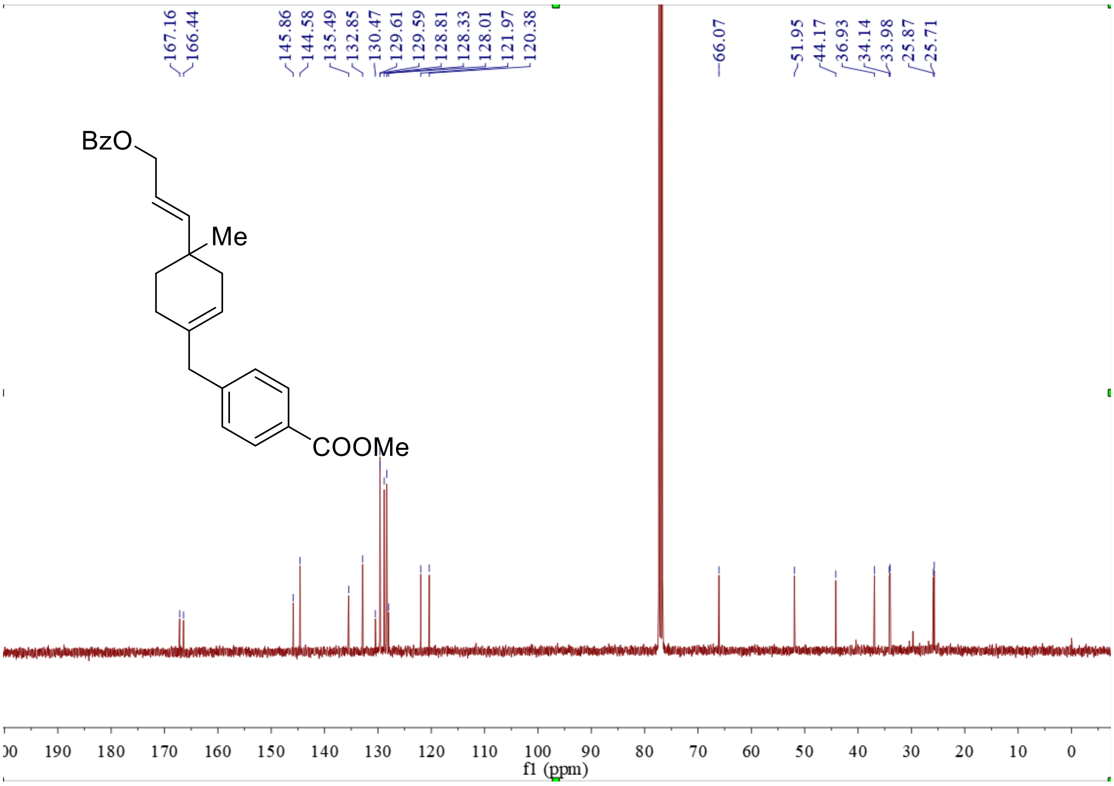

## (*E*)-3-(4-(4-formylbenzyl)-1-methylcyclohex-3-en-1-yl)allyl benzoate(16)

$^{1}$H NMR (400 MHz, $CDCl_3$)

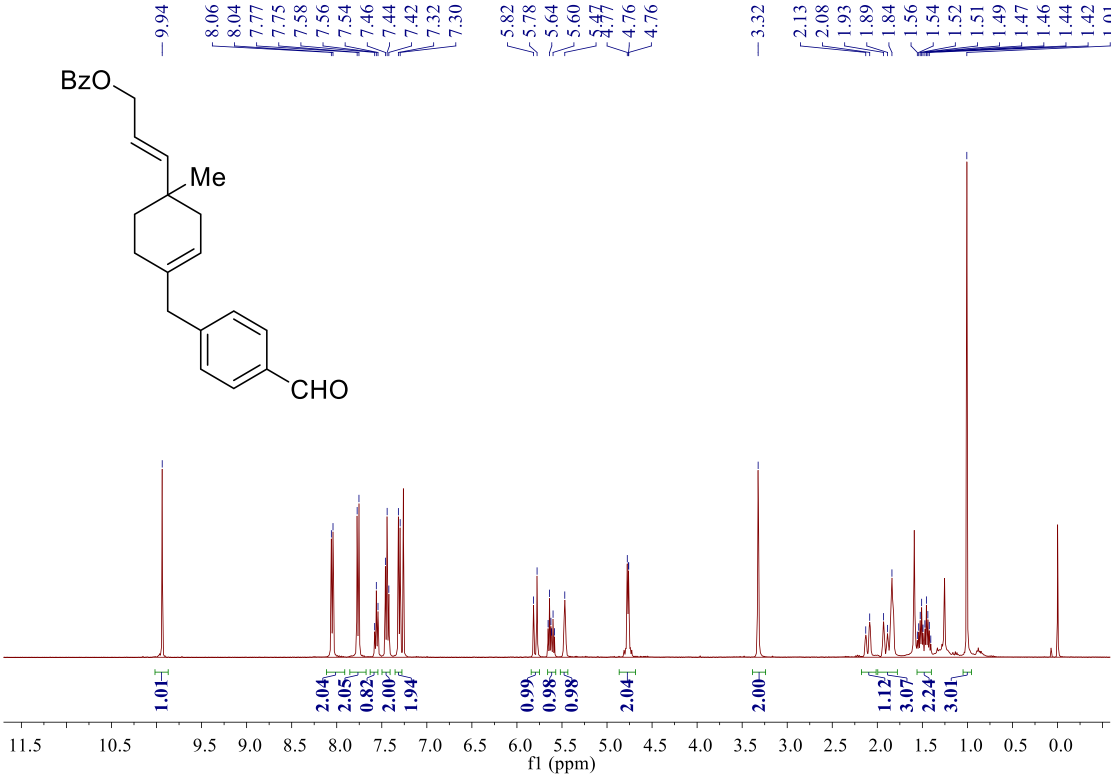


$^{13}$C NMR (100 MHz, $CDCl_3$)

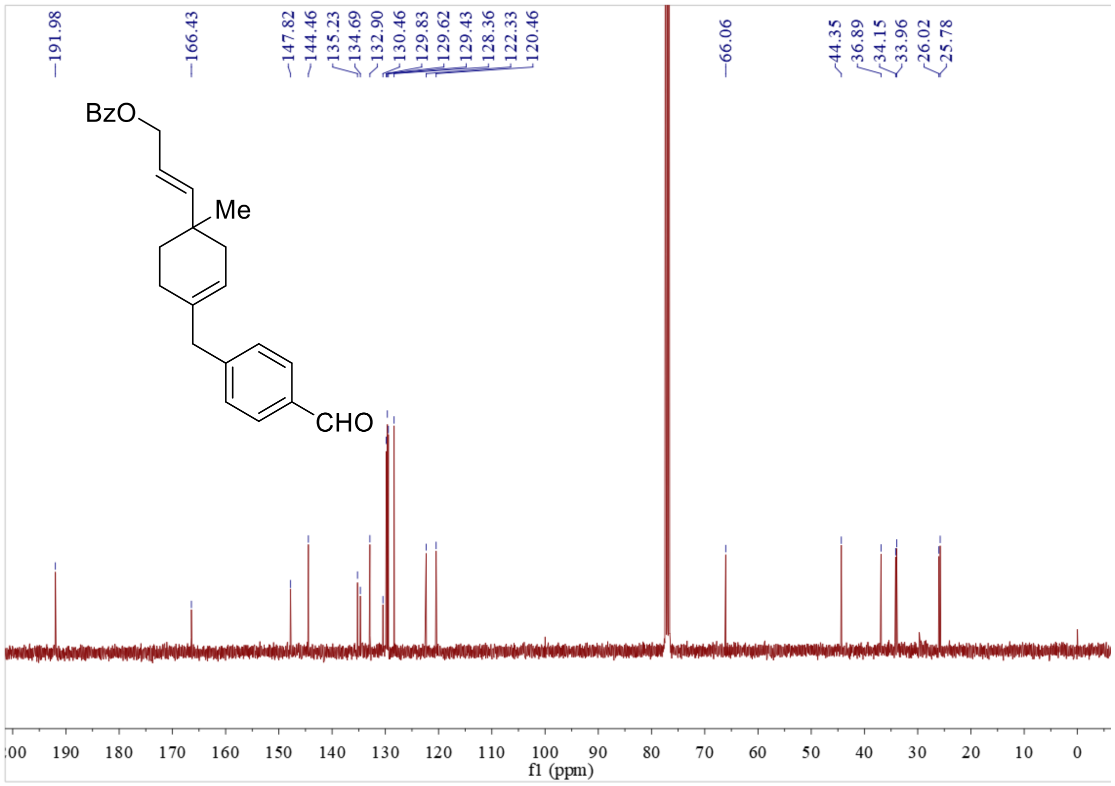

**(*E*)-3-(1-methyl-4-((2-methylpyridin-4-yl)methyl)cyclohex-3-en-1-yl)allyl benzoate (17)**

$^{1}H$ NMR (400 MHz, $CDCl_3$)

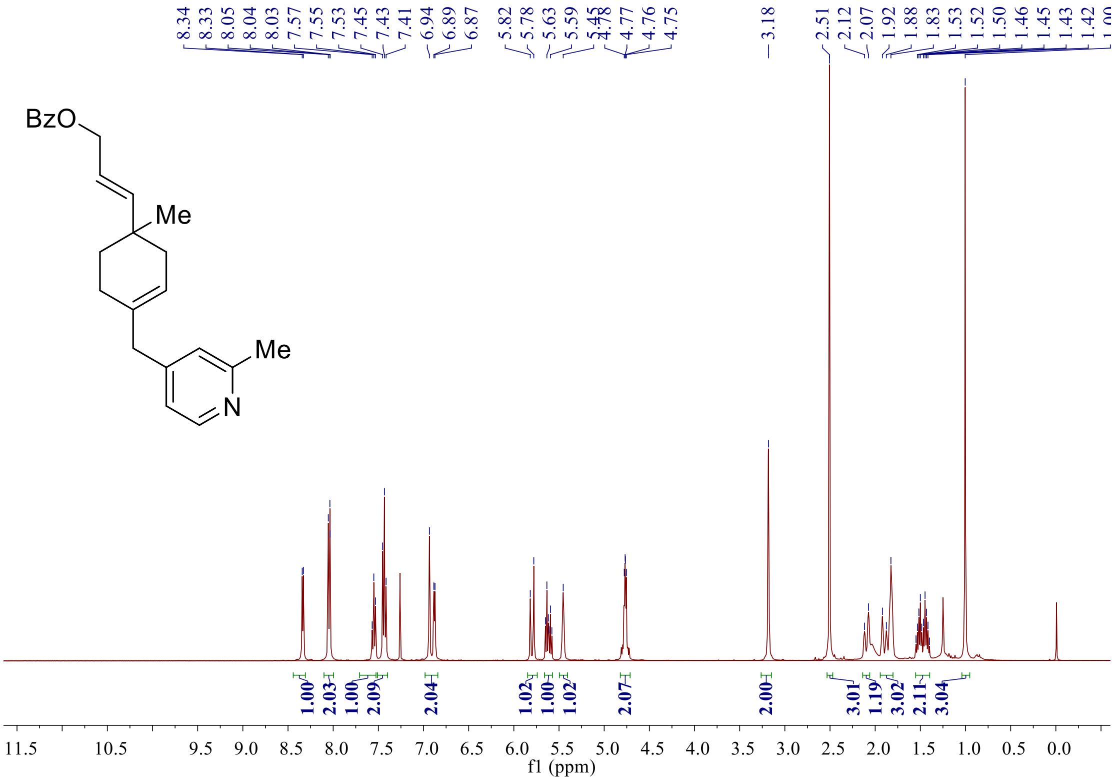


$^{13}C$ NMR (100 MHz, $CDCl_3$)

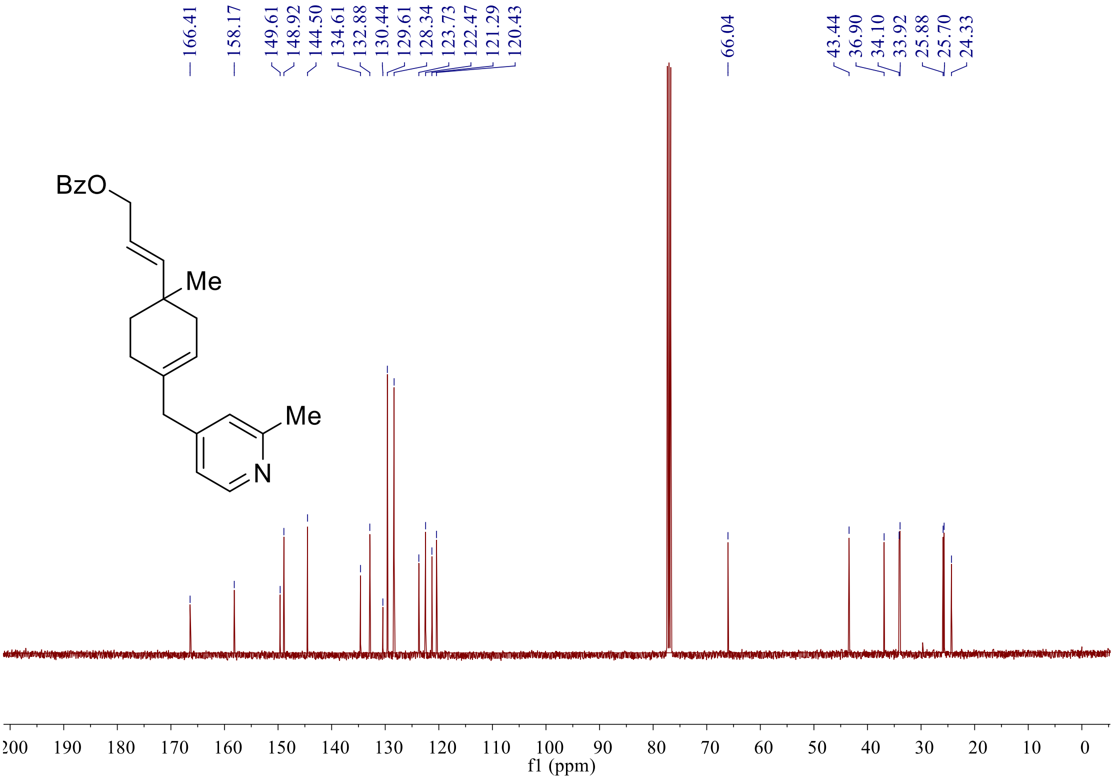

**(*E*)-3-(1-methyl-4-((2-(trifluoromethyl)pyridin-4-yl)methyl)cyclohex-3-en-1-yl)allyl benzoate (18)**

$^1$H NMR (400 MHz, CDCl$_3$)

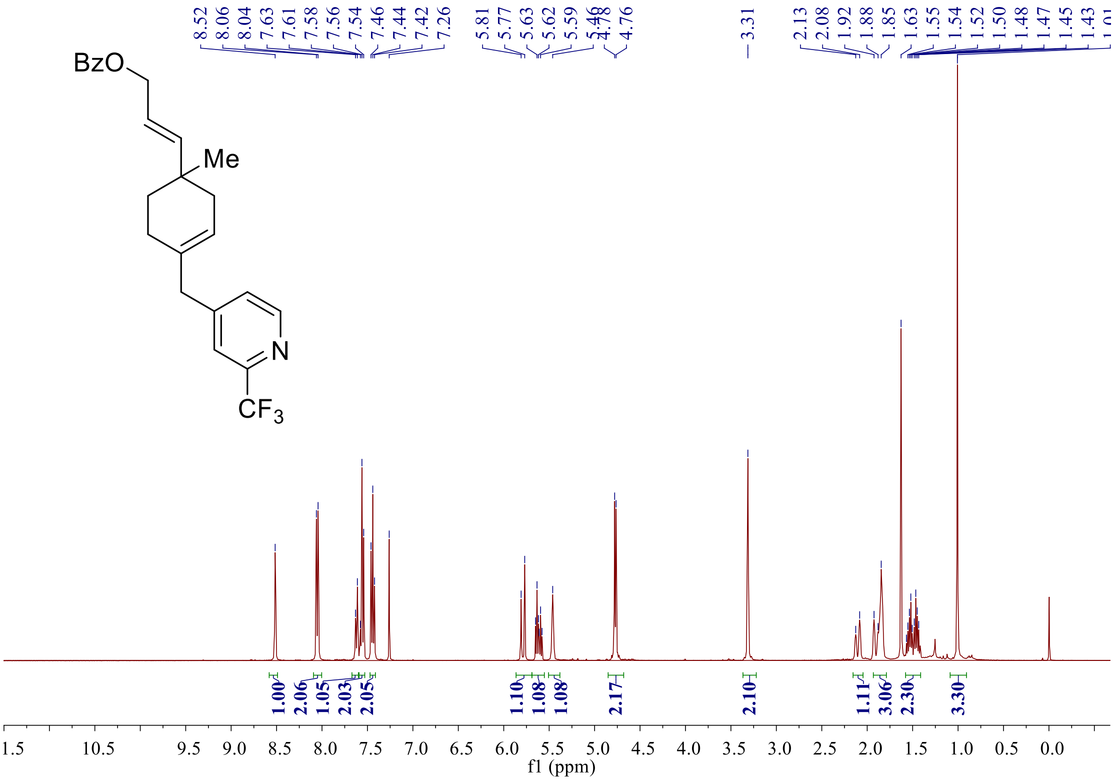


$^{19}$F NMR (377 MHz, CDCl$_3$)

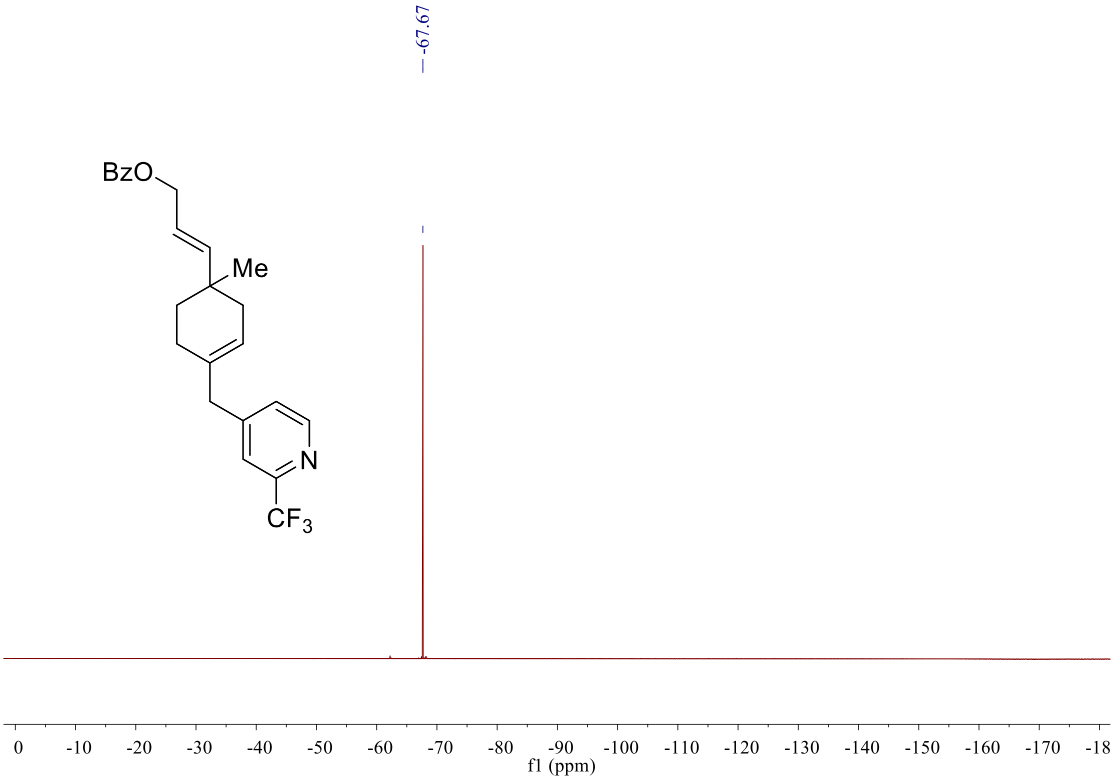

$^{13}C$ NMR (100 MHz, $CDCl_3$)

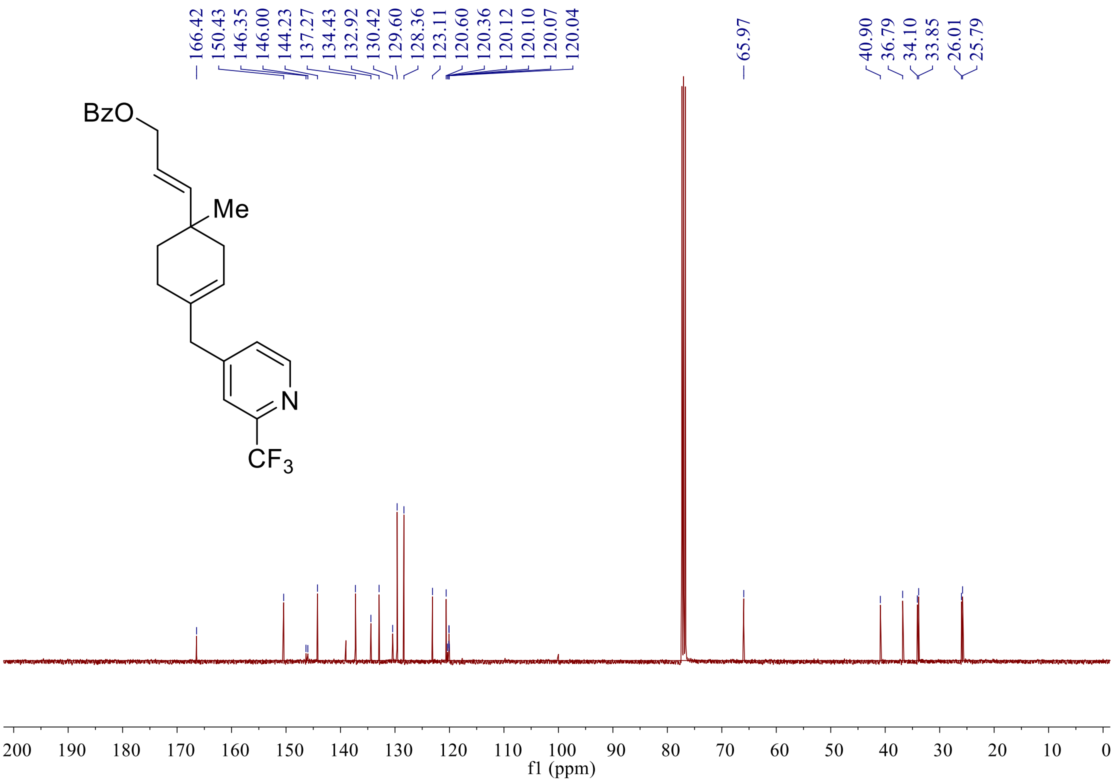


## (*E*)-3-(3-(4-cyanophenyl)-1-methylcyclohexyl)allyl 4-acetoxybenzoate (19)

$^{1}H$ NMR (400 MHz, $CDCl_3$)

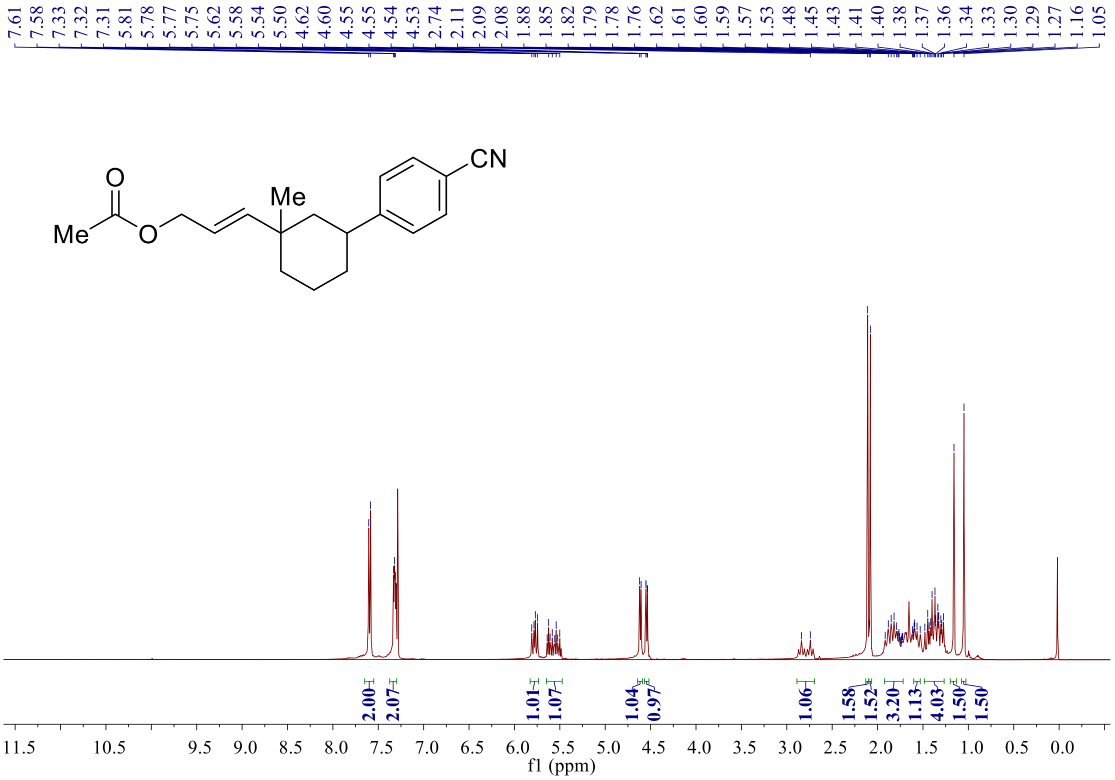

$^{13}C$ NMR (100 MHz, $CDCl_3$)

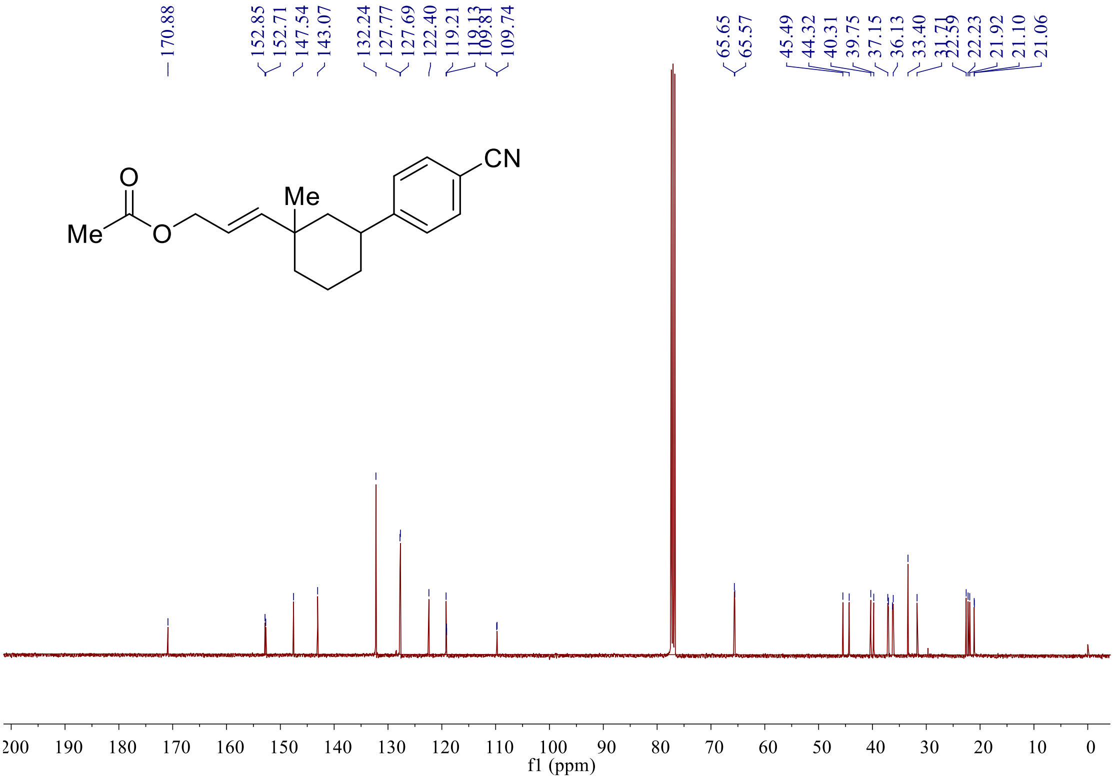


**(*E*)-3-(3-(4-cyanophenyl)-1-methylcyclohexyl)allyl cyclohexanecarboxylate(20)**

$^{1}H$ NMR (600 MHz, $CDCl_3$)

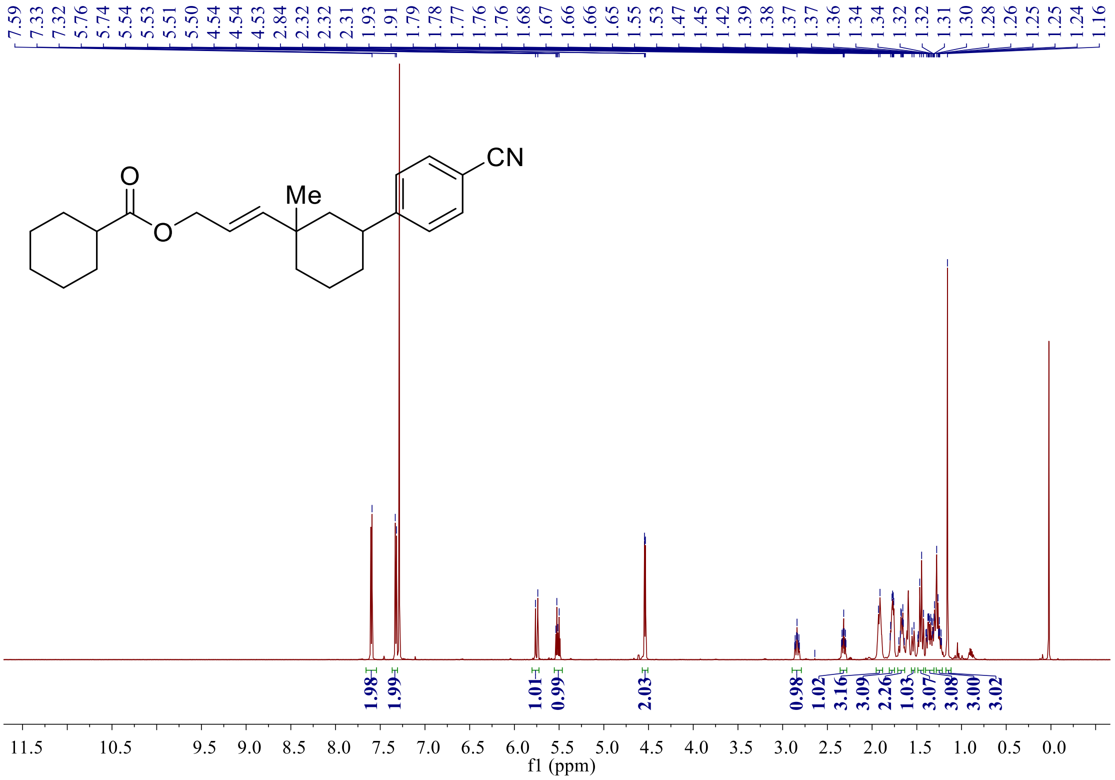

$^{13}C$ NMR (150 MHz, $CDCl_3$)

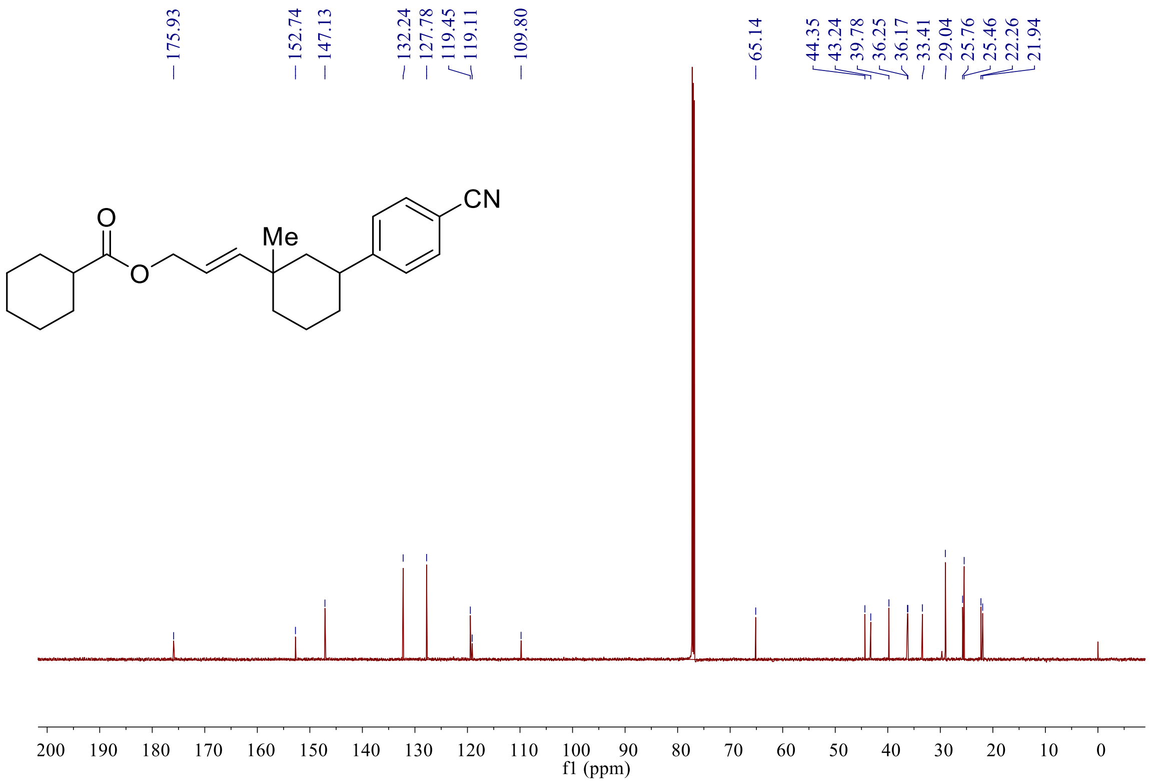


**(*E*)-3-(3-(4-cyanophenyl)-1-methylcyclohexyl)allyl 1-methyl-1H-indole-3-carboxylate(21)**

$^{1}H$ NMR (600 MHz, $CDCl_3$)

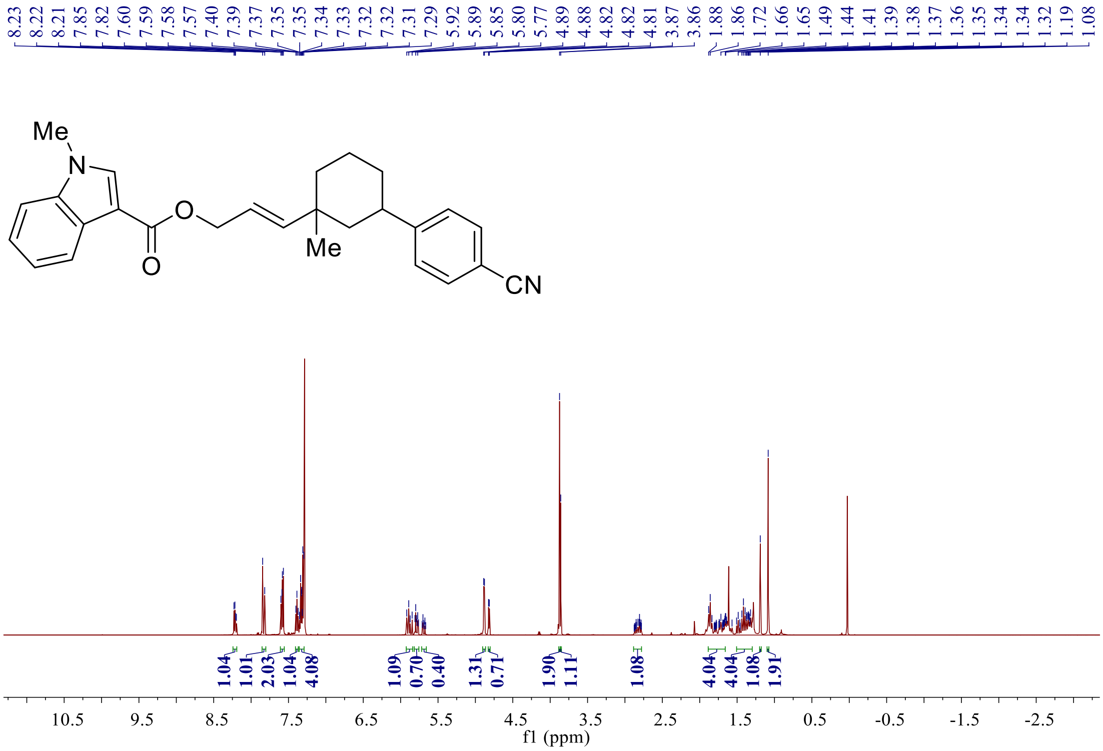

$^{13}C$ NMR (150 MHz, $CDCl_3$)

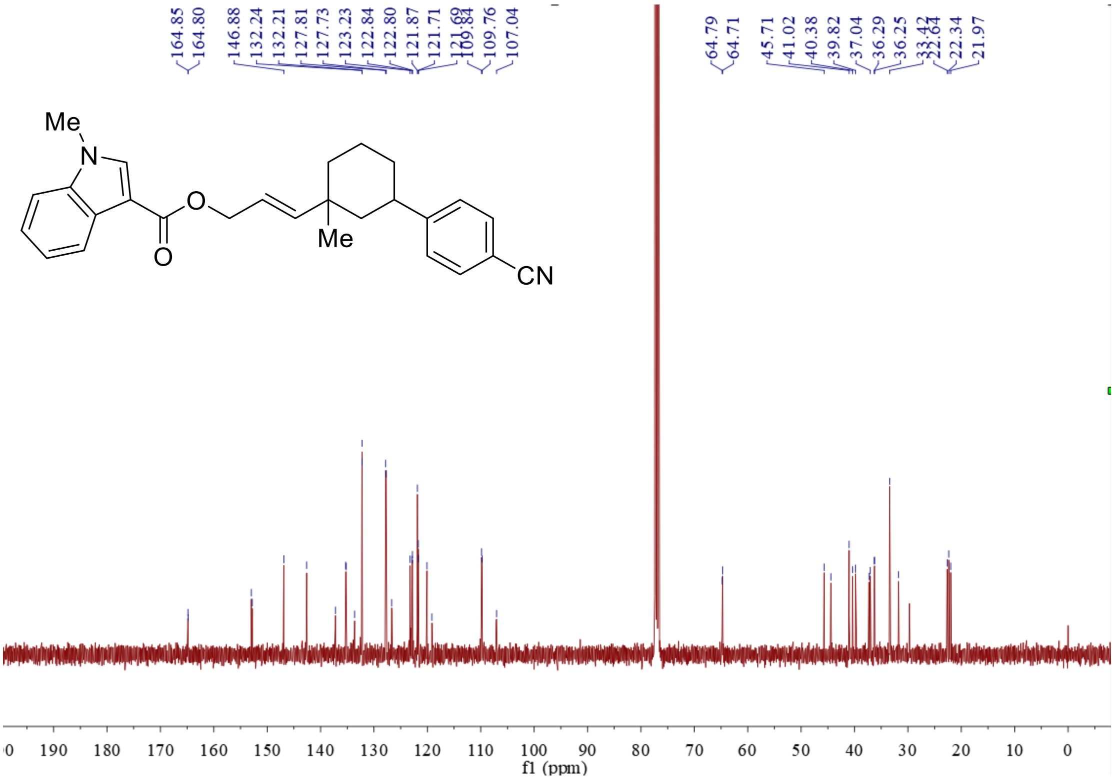


**(*E*)-3-(3-(4-cyanophenyl)-1-methylcyclohexyl)allyl isonicotinate (22)**

$^{1}H$ NMR (600 MHz, $CDCl_3$)

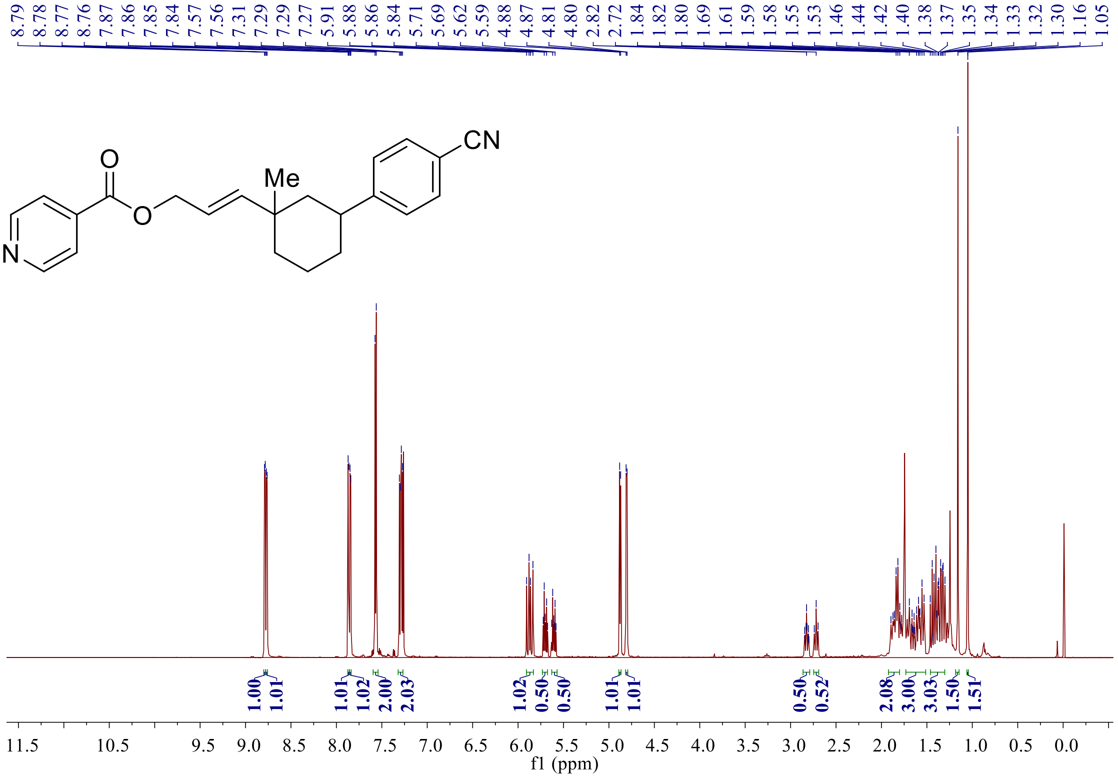

$^{13}C$ NMR (150 MHz, $CDCl_3$)

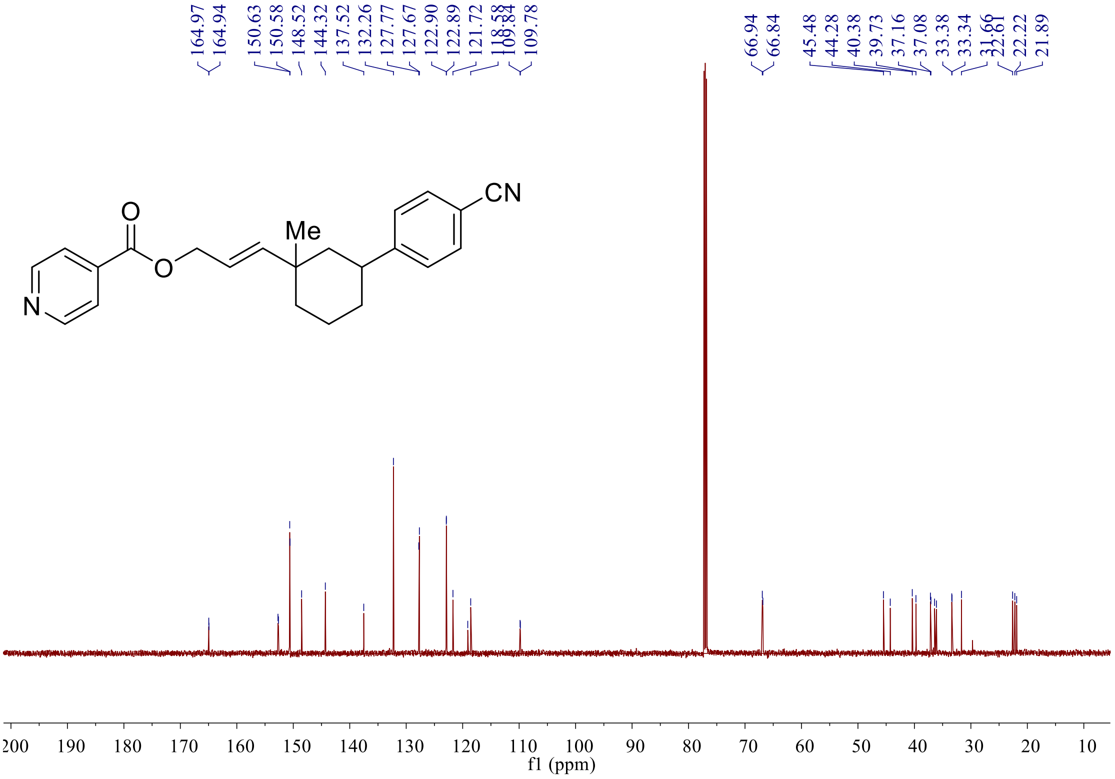


## (*E*)-3-(3-(4-cyanophenyl)-1-methylcyclohexyl)allyl 4-(tert-butyl)benzoate (23)

$^{1}H$ NMR (600 MHz, $CDCl_3$)

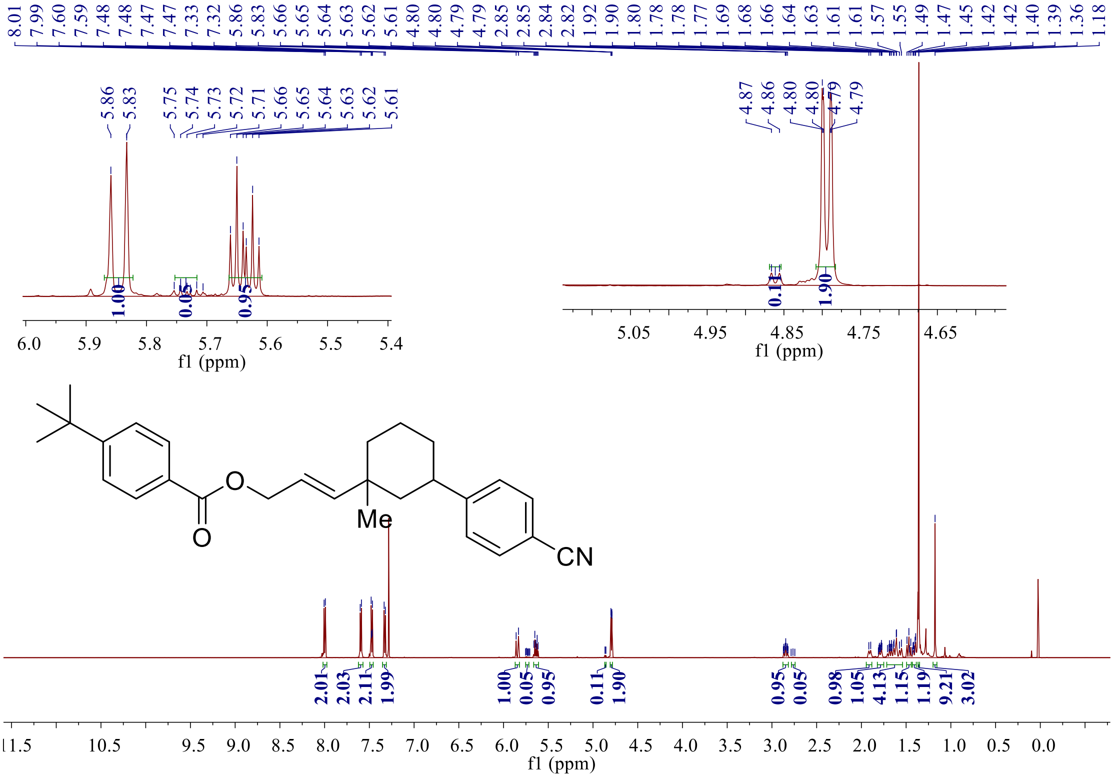

$^{13}C$ NMR (150 MHz, $CDCl_3$)

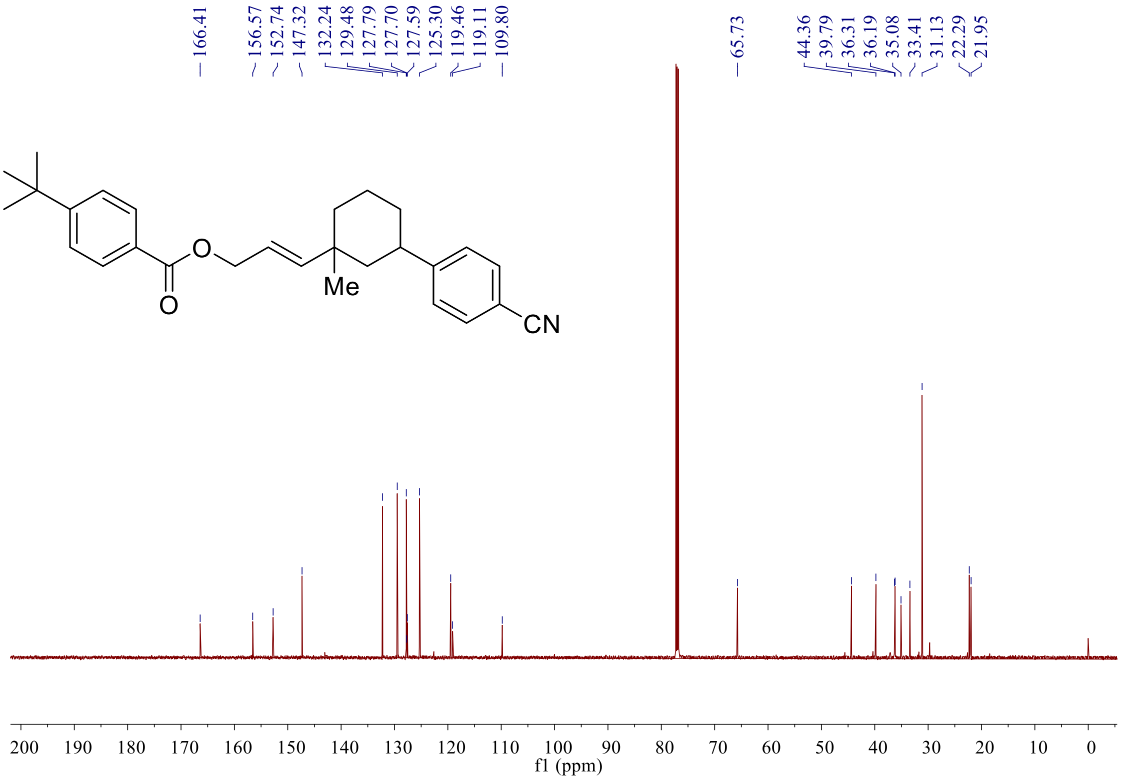


**(*E*)-3-(3-(4-cyanophenyl)-1-methylcyclohexyl)allyl 4-acetoxybenzoate (24)**

$^{1}H$ NMR (400 MHz, $CDCl_3$)

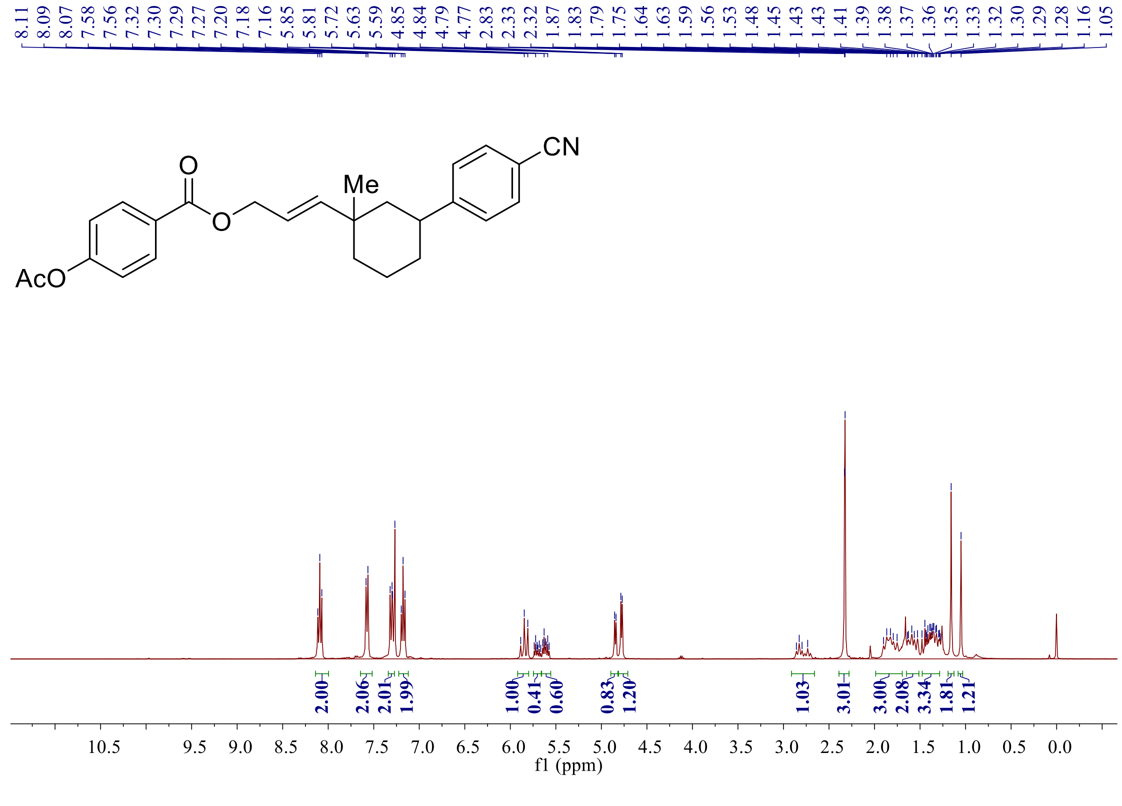

$^{13}$C NMR (100 MHz, $CDCl_3$)

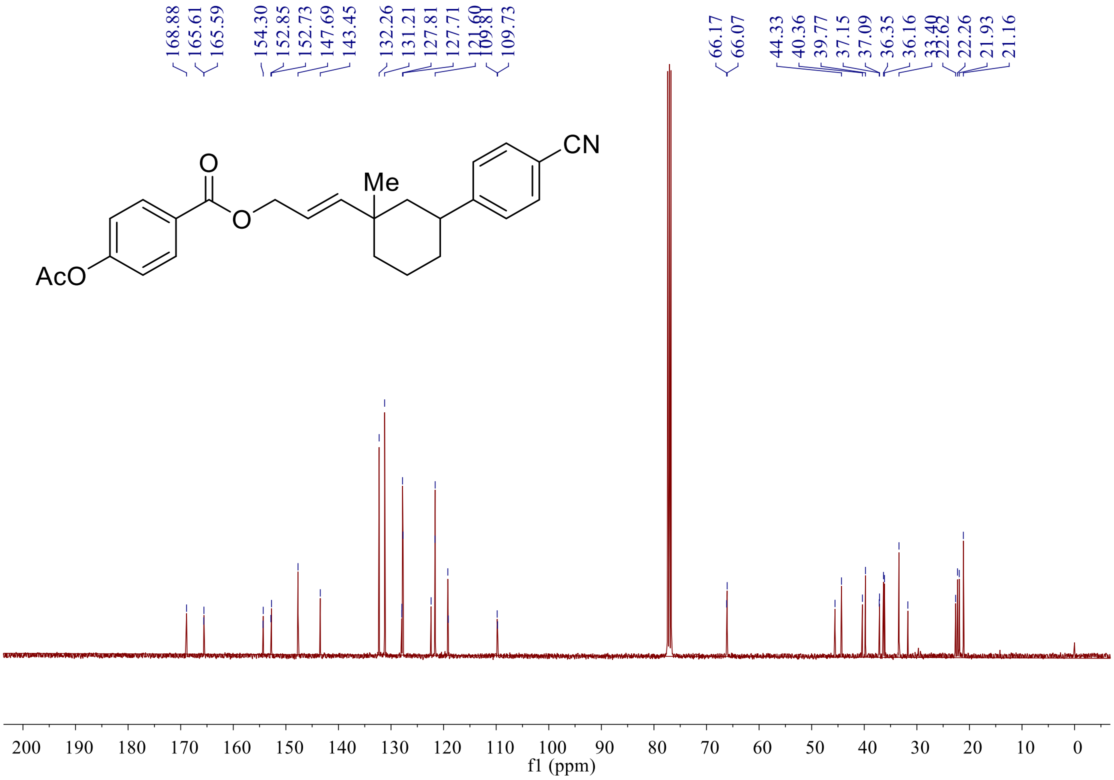


**(*E*)-3-(3-(4-cyanophenyl)-1-methylcyclohexyl)allyl 3,5-dimethylbenzoate (25)**

$^{1}$H NMR (600 MHz, $CDCl_3$)

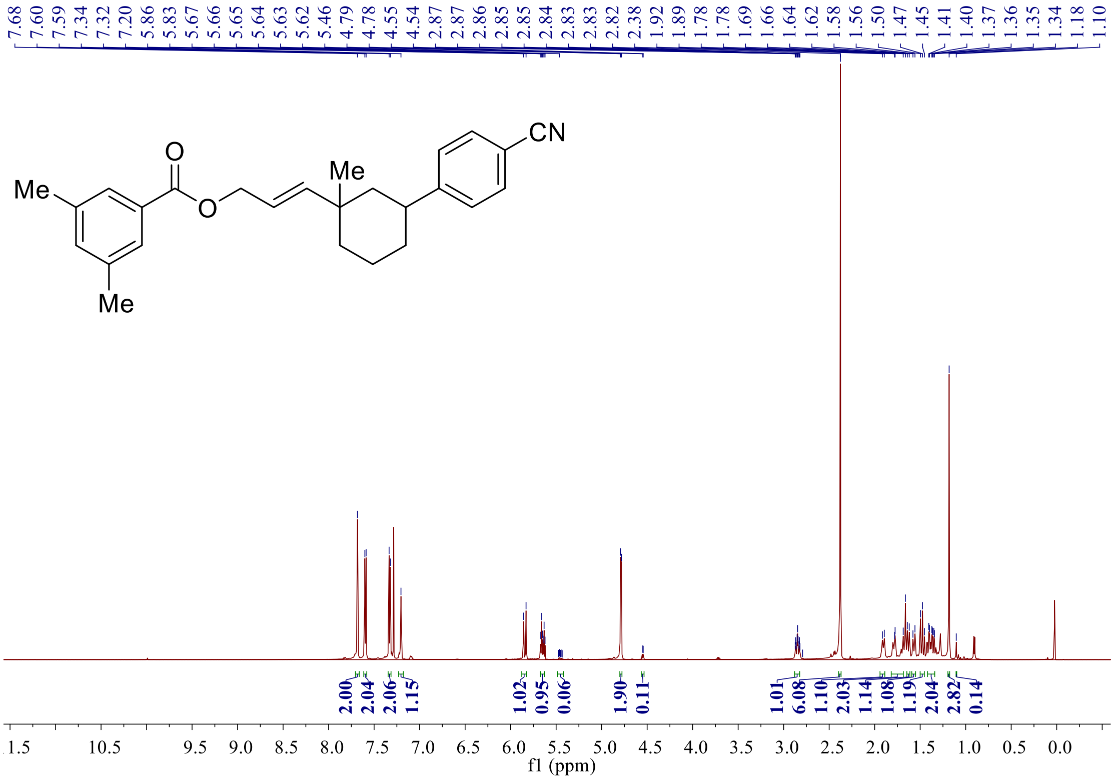

$^{13}$C NMR (150 MHz, $CDCl_3$)

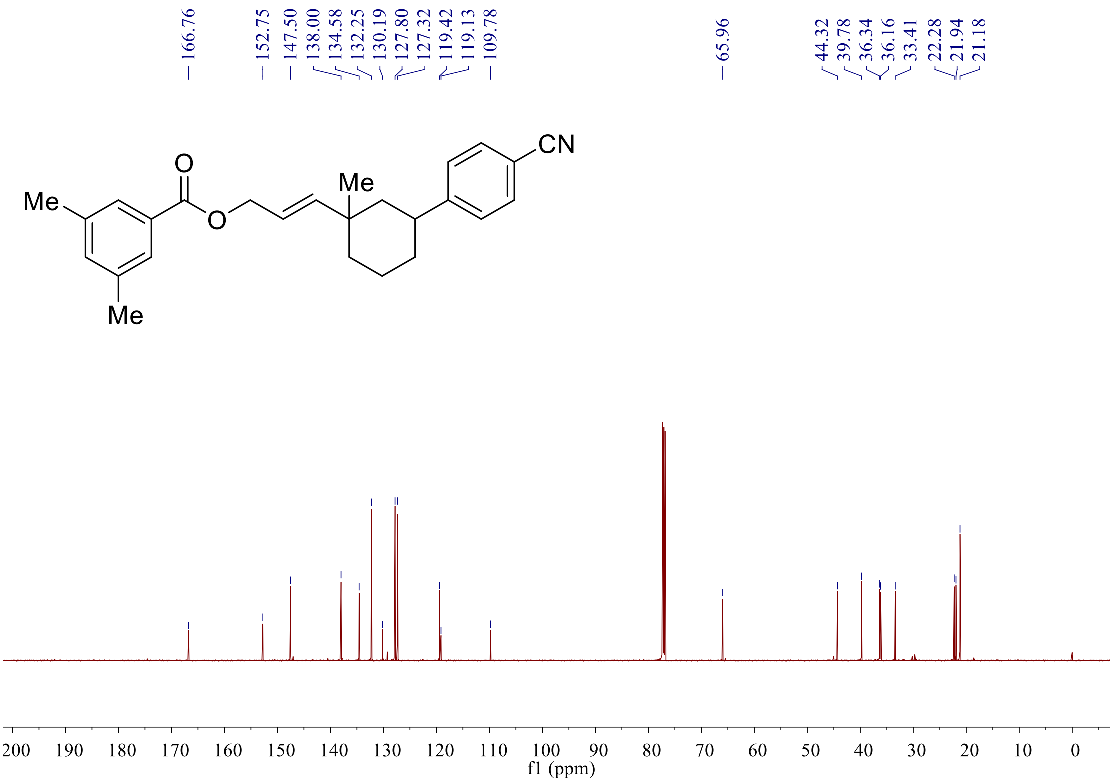


**(*E*)-4-(3-(3-(1,3-dioxoisoindolin-2-yl)prop-1-en-1-yl)-3-methyl cyclohexyl) benzonitrile(26)**

$^{1}$H NMR (600 MHz, $CDCl_3$)

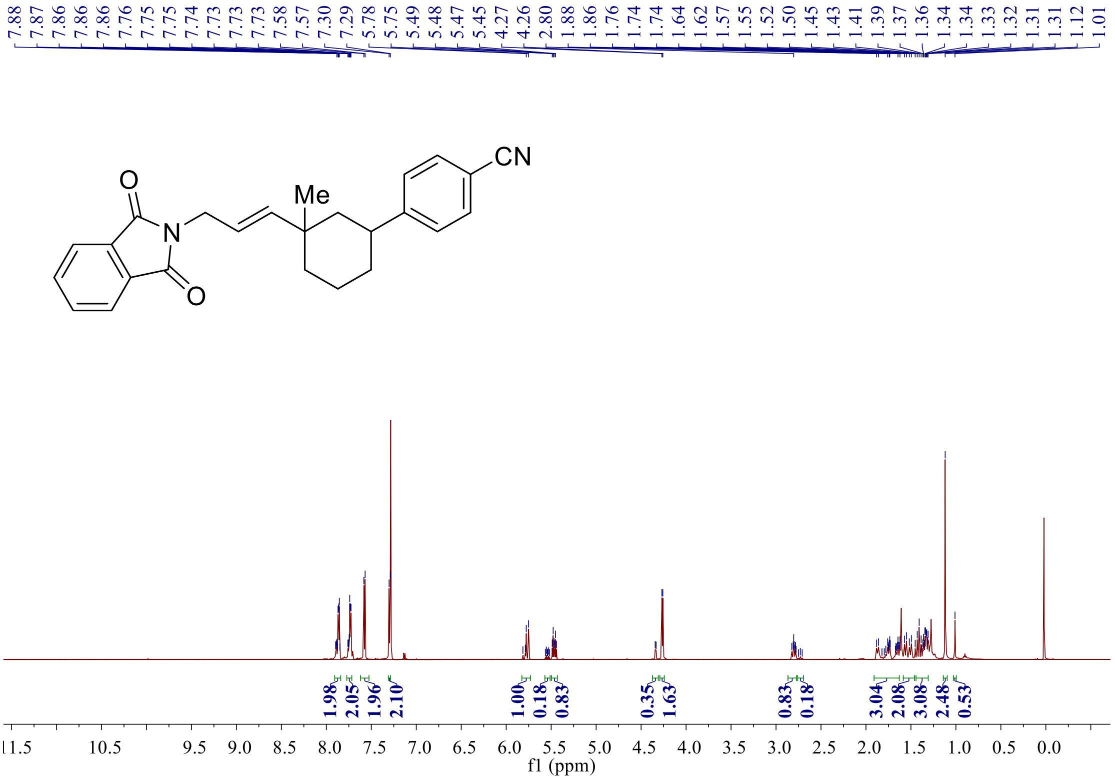

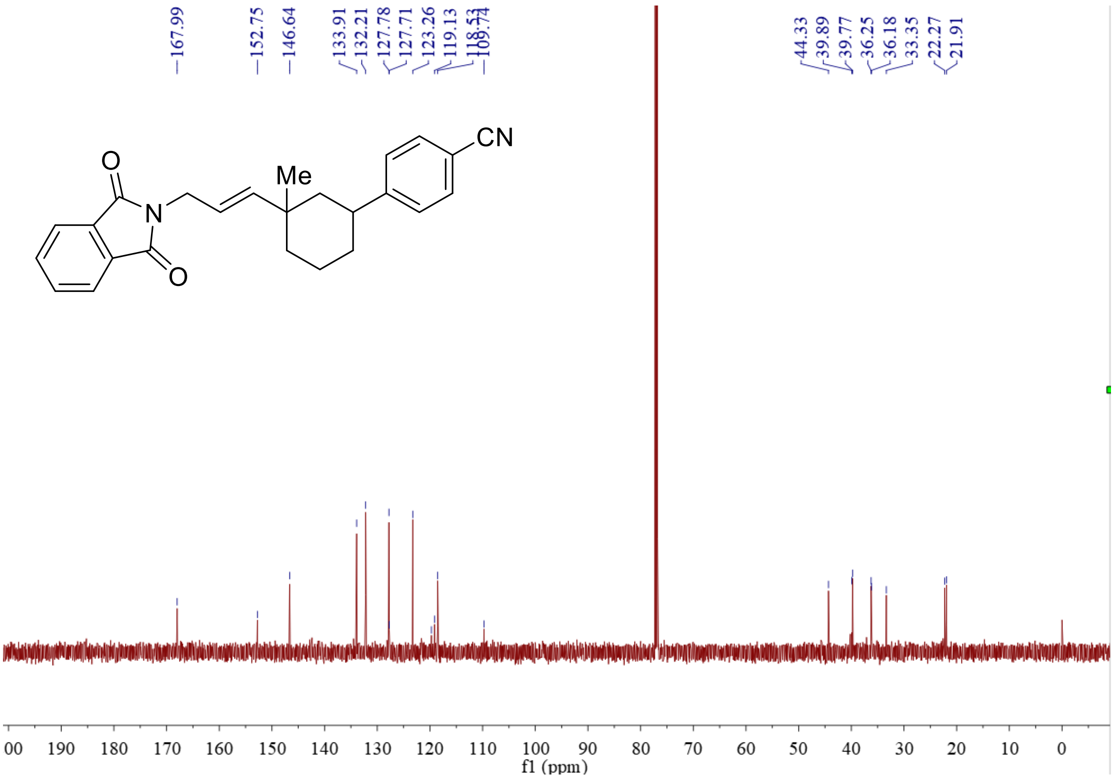


## (*E*)-3-(3-(4-cyanophenyl)-1-methylcyclohexyl)allyl 3-(4,5-diphenyloxazol-2-yl)propanoate(27)

$^1$H NMR (600 MHz, $CDCl_3$)

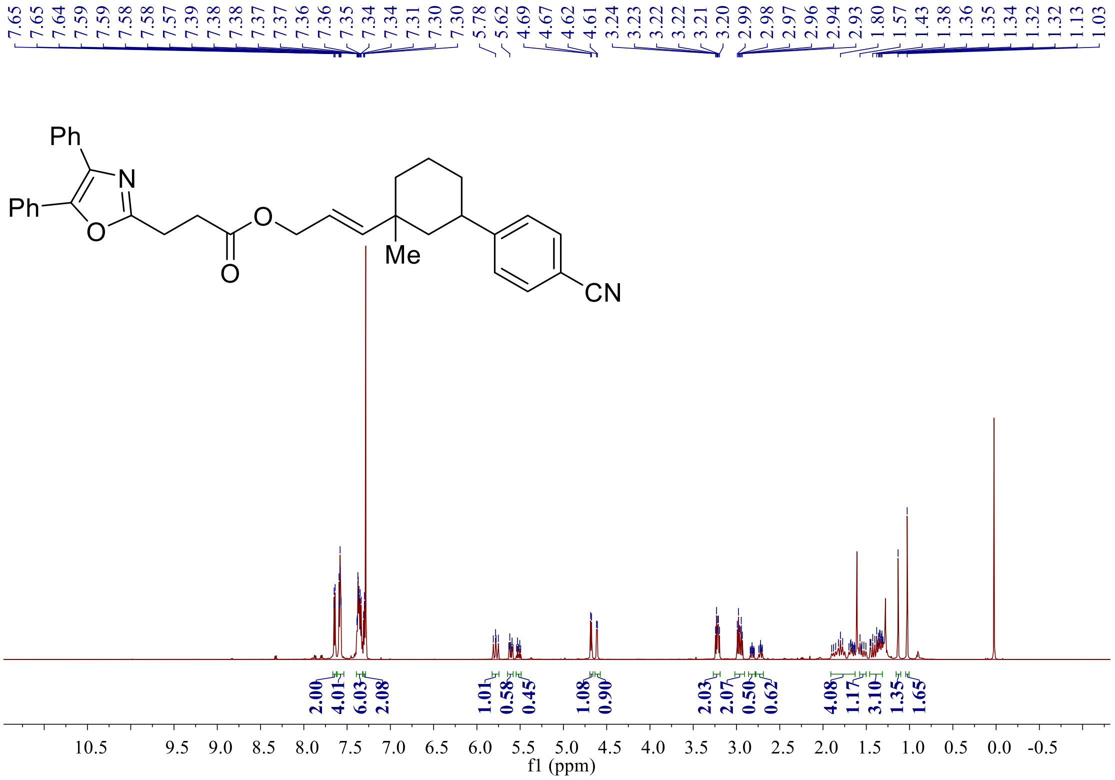

$^{13}$C NMR (150 MHz, $CDCl_3$)

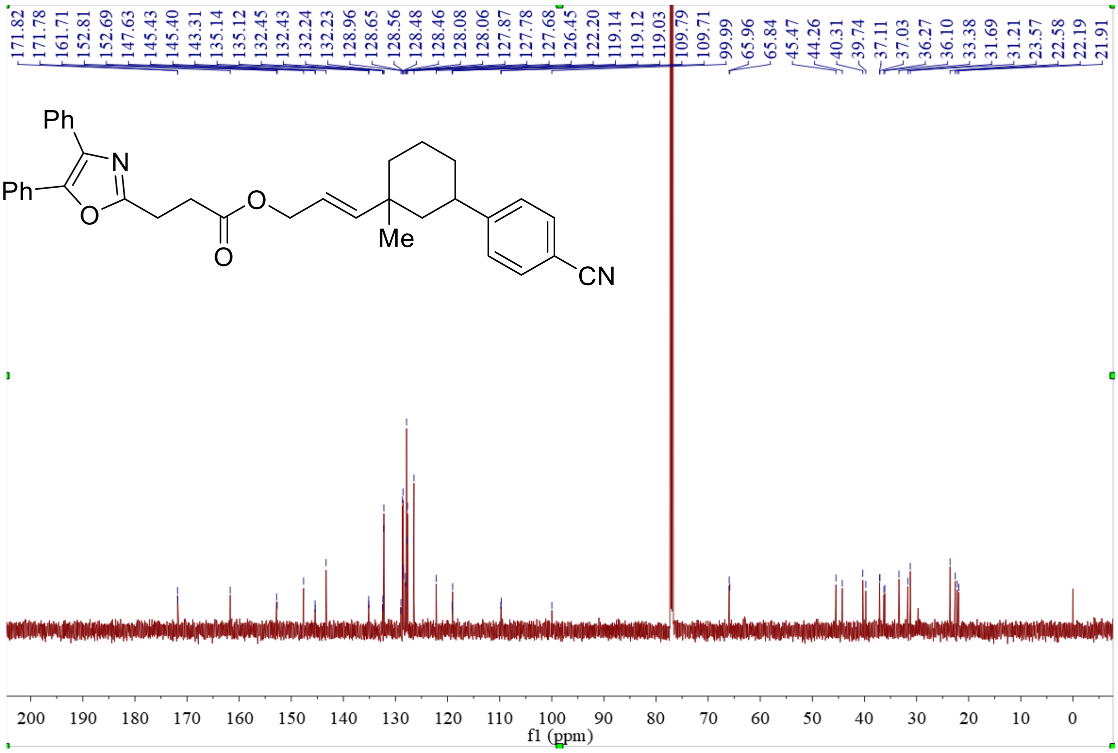


**(*E*)-4-(3-methyl-3-(3-phenoxyprop-1-en-1-yl)cyclohexyl)benzonitrile(28)**

$^{1}$H NMR (600 MHz, $CDCl_3$)

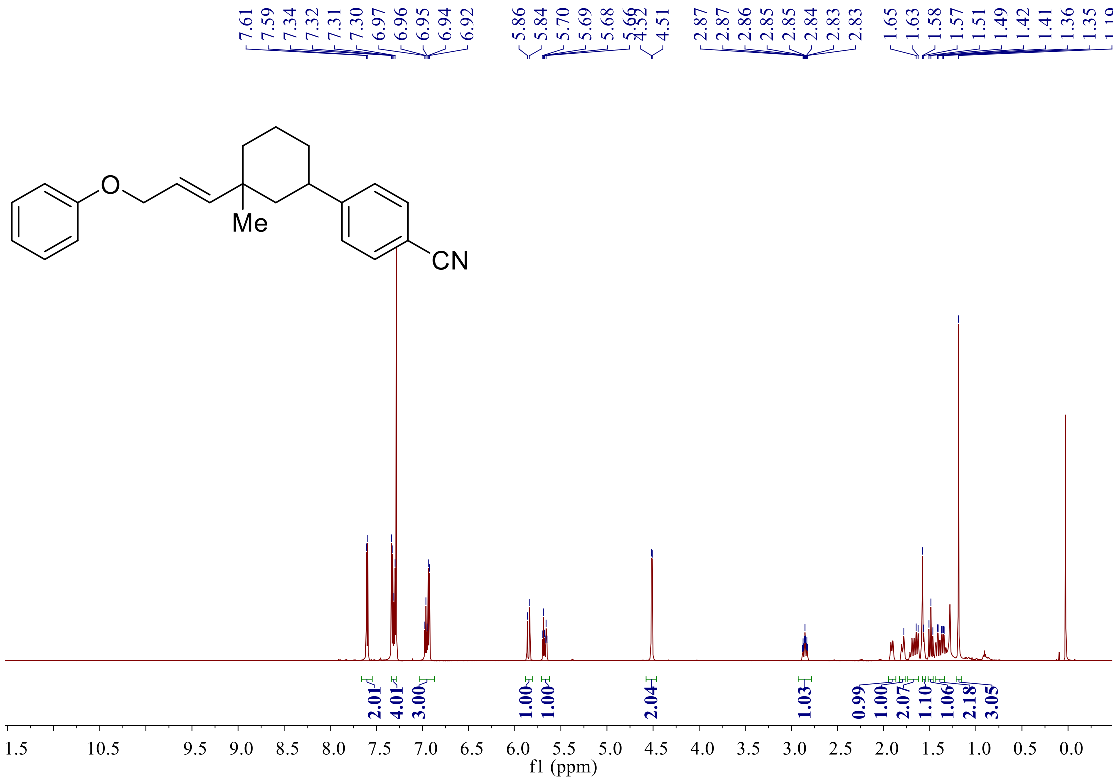

$^{13}C$ NMR (150 MHz, $CDCl_3$)

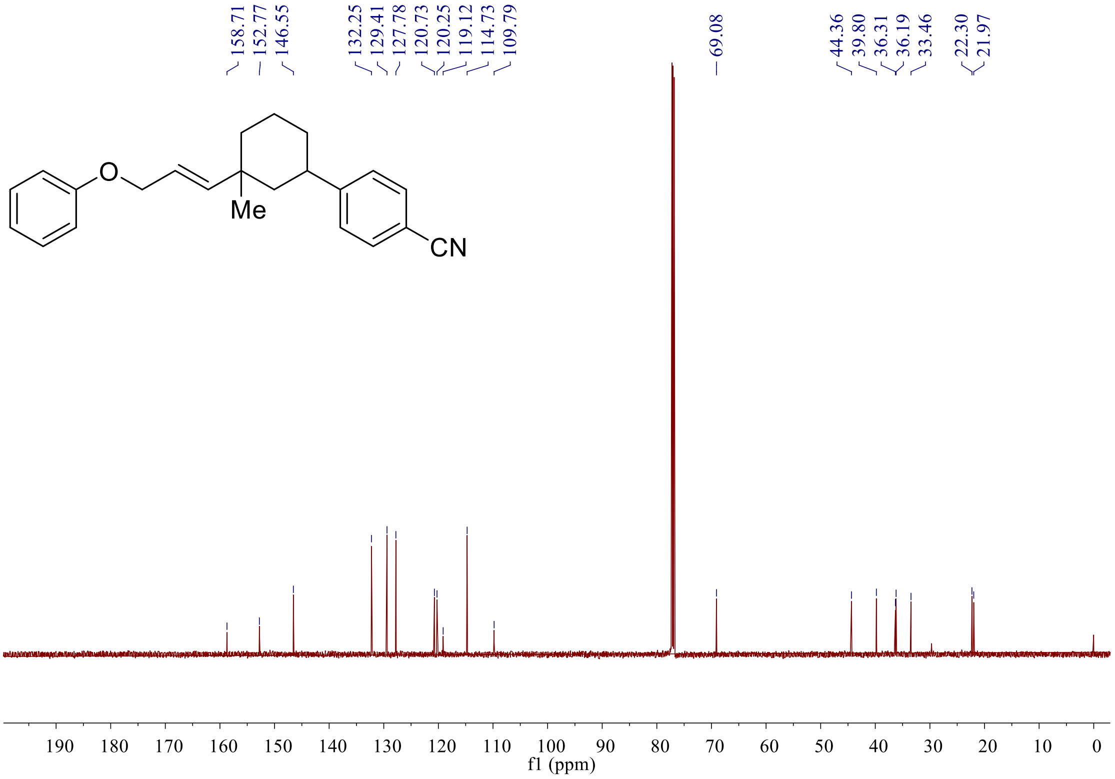


**(*E*)-3-(3-(4-cyanophenyl)-1-methylcyclohexyl)allyl 6-isopropyl-1,4a-dimethyl-1,2,3,4,4a,9,10,10a-octahydrophenanthrene-1-carboxylate (29)**

$^{1}H$ NMR (600 MHz, $CDCl_3$)

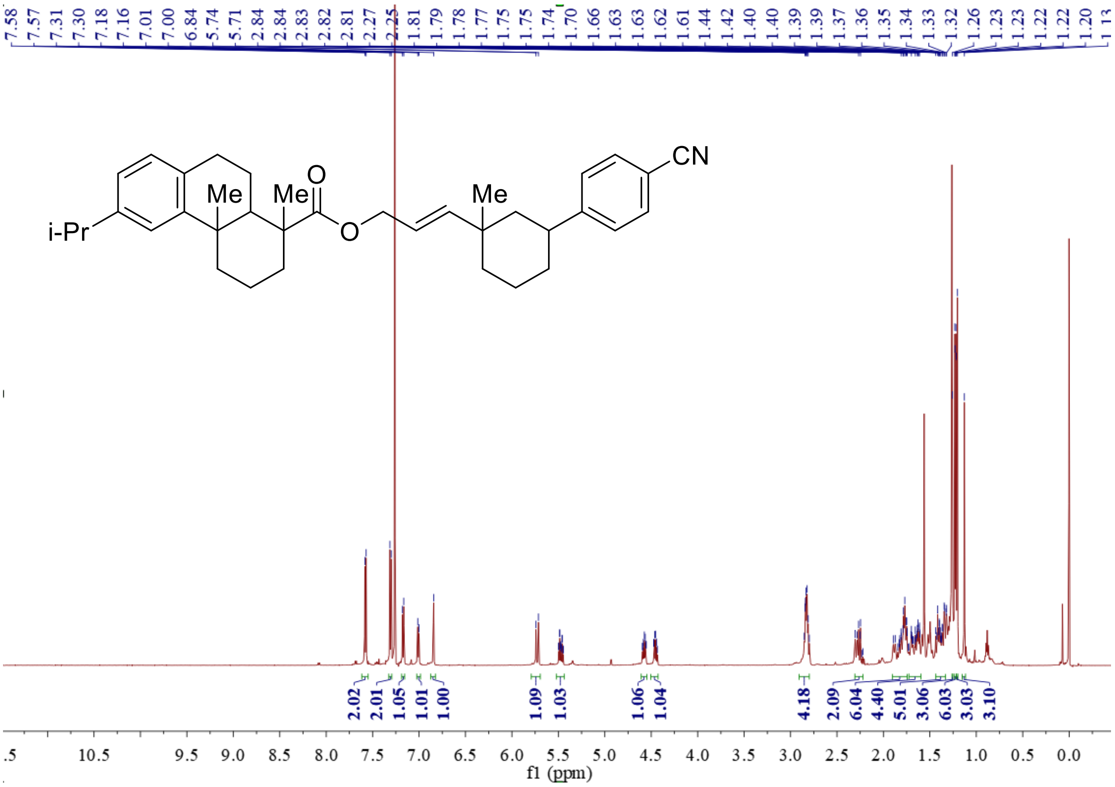

$^{13}$C NMR (150 MHz, $CDCl_3$)

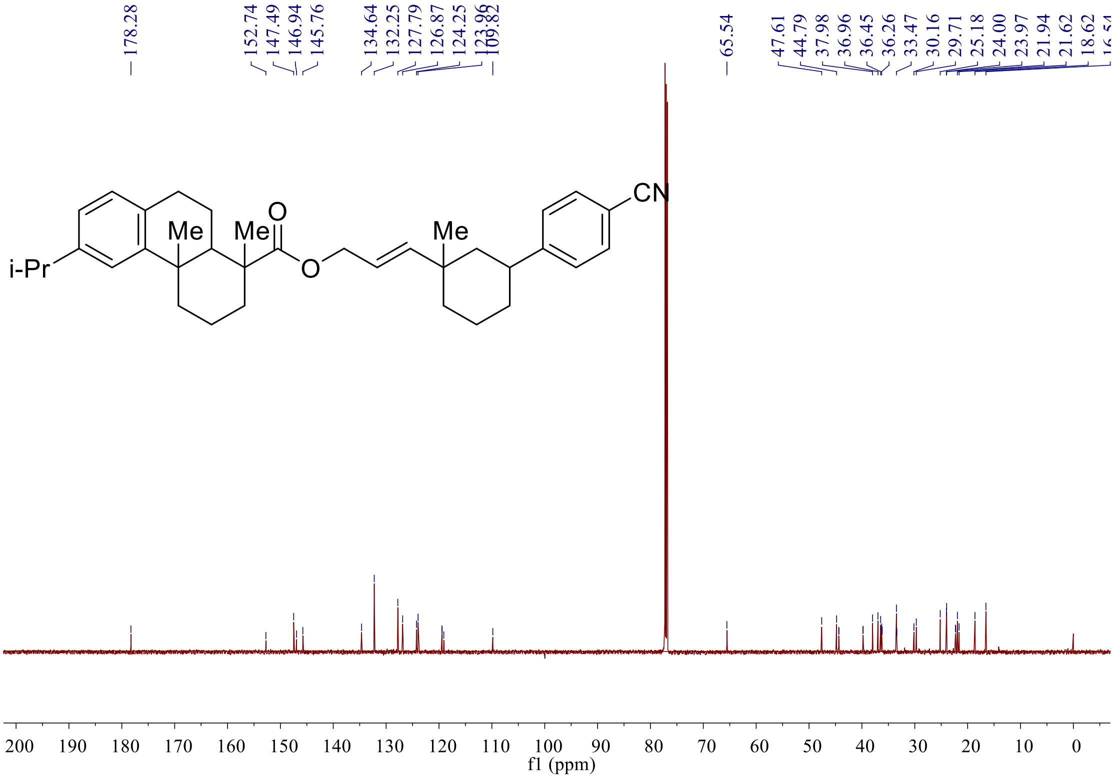


**(*E*)-3-(3-(4-acetylphenyl)-1-methylcyclohexyl)allyl benzoate (30)**

$^{1}$H NMR (400 MHz, $CDCl_3$)

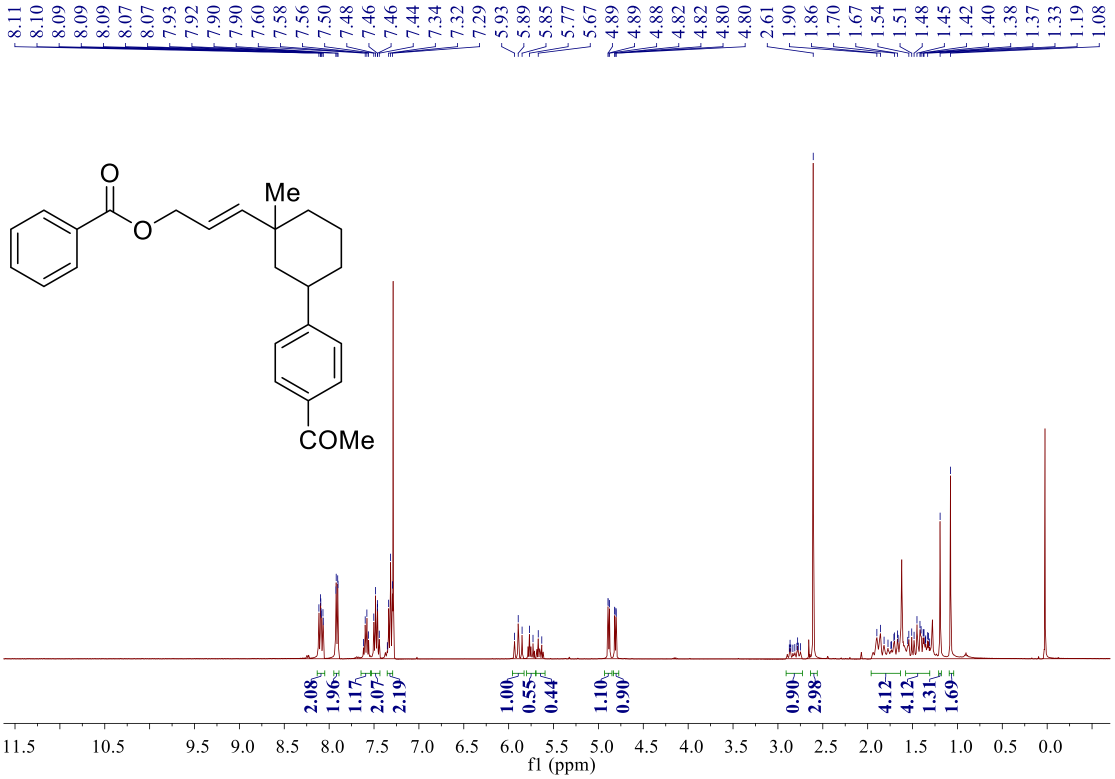

$^{13}C$ NMR (100 MHz, $CDCl_3$)

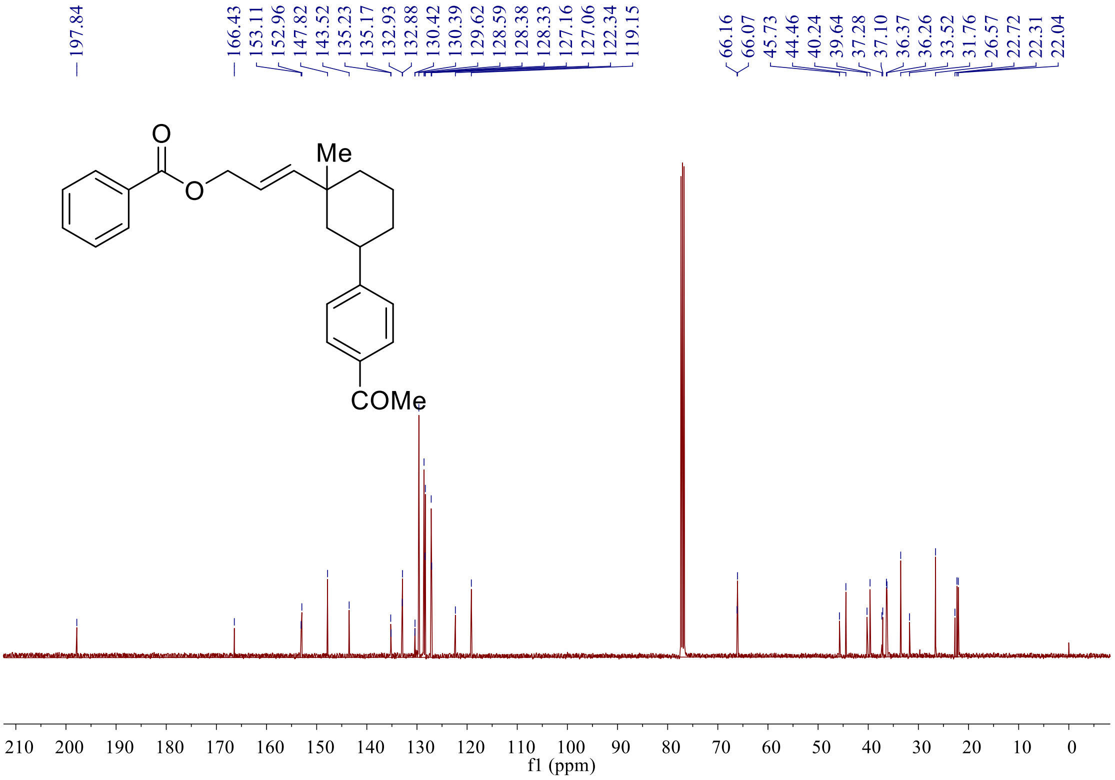


**Methyl (*E*)-4-(3-(3-(benzoyloxy)prop-1-en-1-yl)-3-methylcyclohexyl)benzoate(31)**

$^{1}H$ NMR (600 MHz, $CDCl_3$)

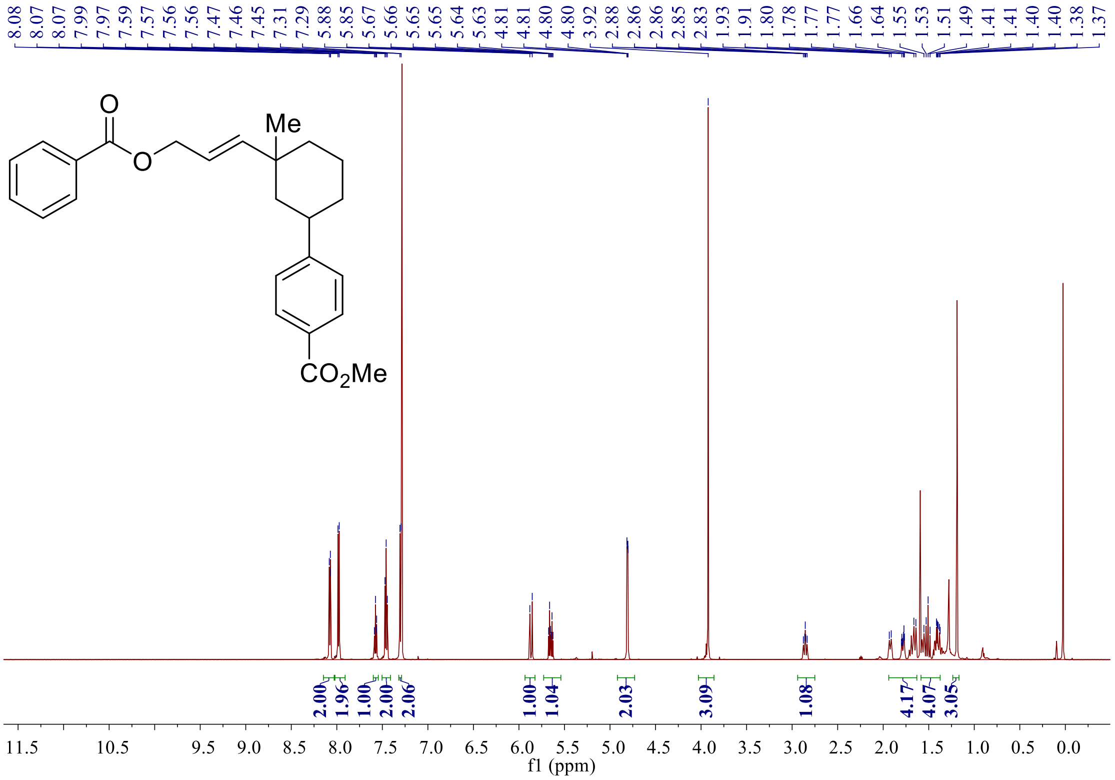

$^{13}$C NMR (150 MHz, $CDCl_3$)

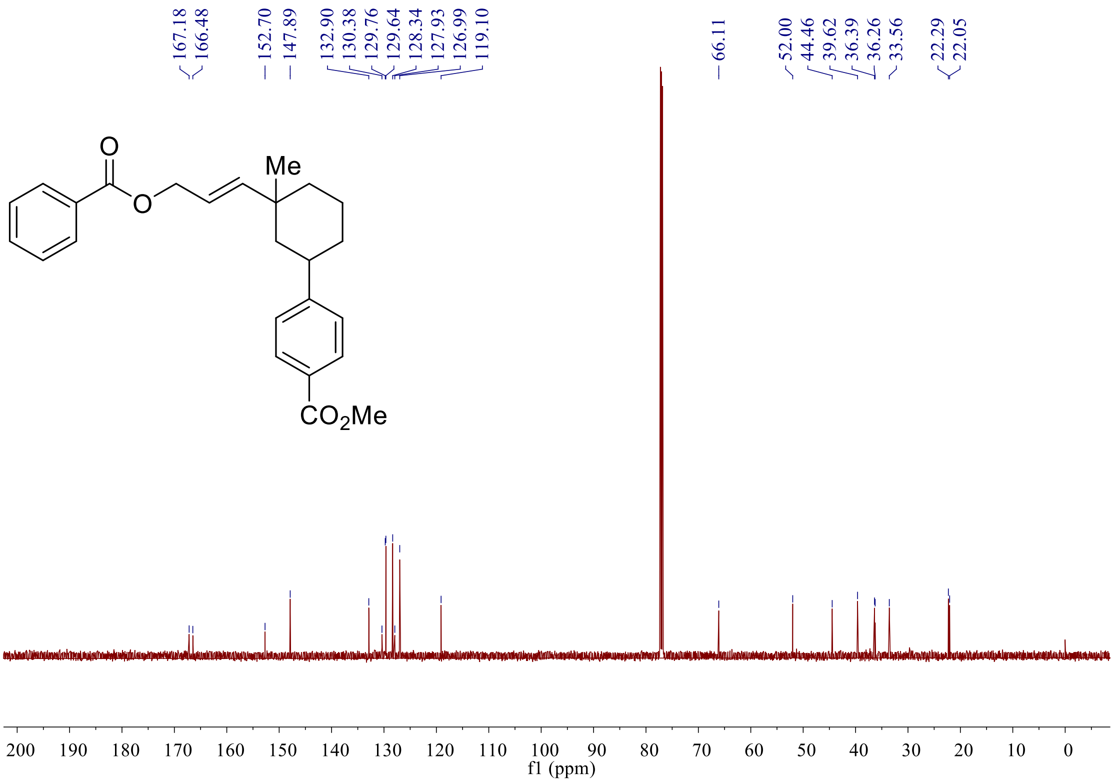


**Methyl (*E*)-4-(3-(3-(benzoyloxy)prop-1-en-1-yl)-3-methylcyclohexyl)benzoate**

**(*E*)-3-(3-(4-benzoylphenyl)-1-methylcyclohexyl)allyl benzoate(32)**

$^{1}$H NMR (600 MHz, $CDCl_3$)

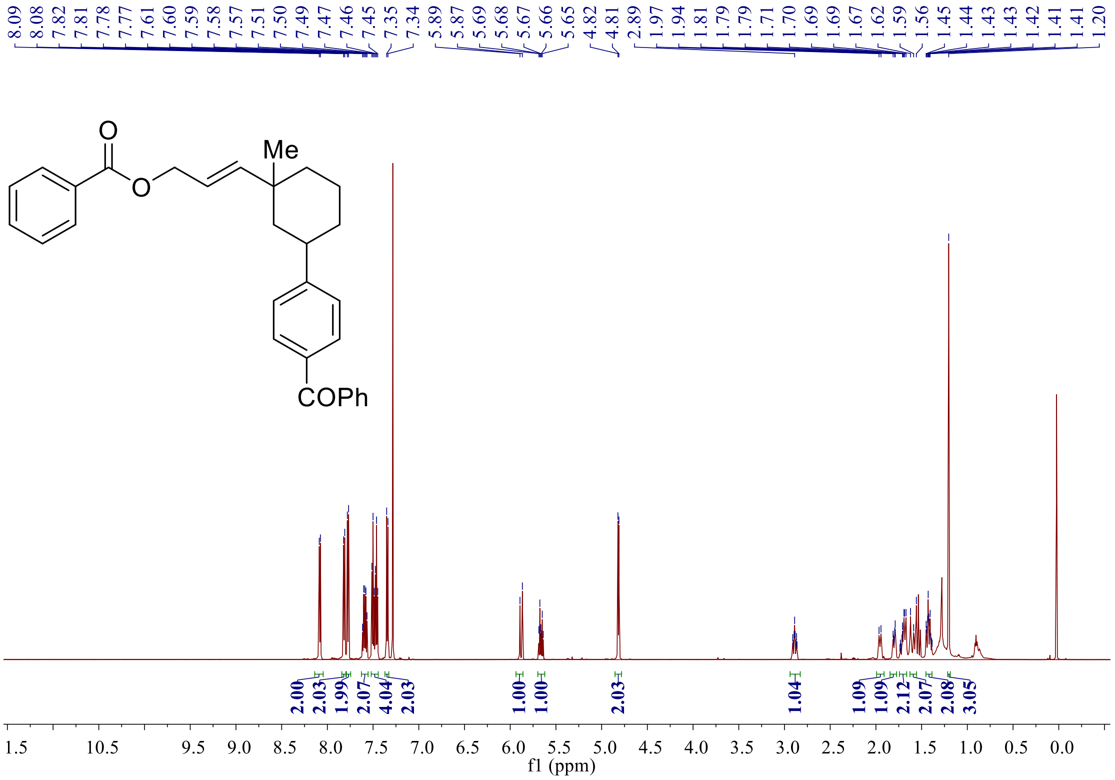

$^{13}$C NMR (150 MHz, $CDCl_3$)

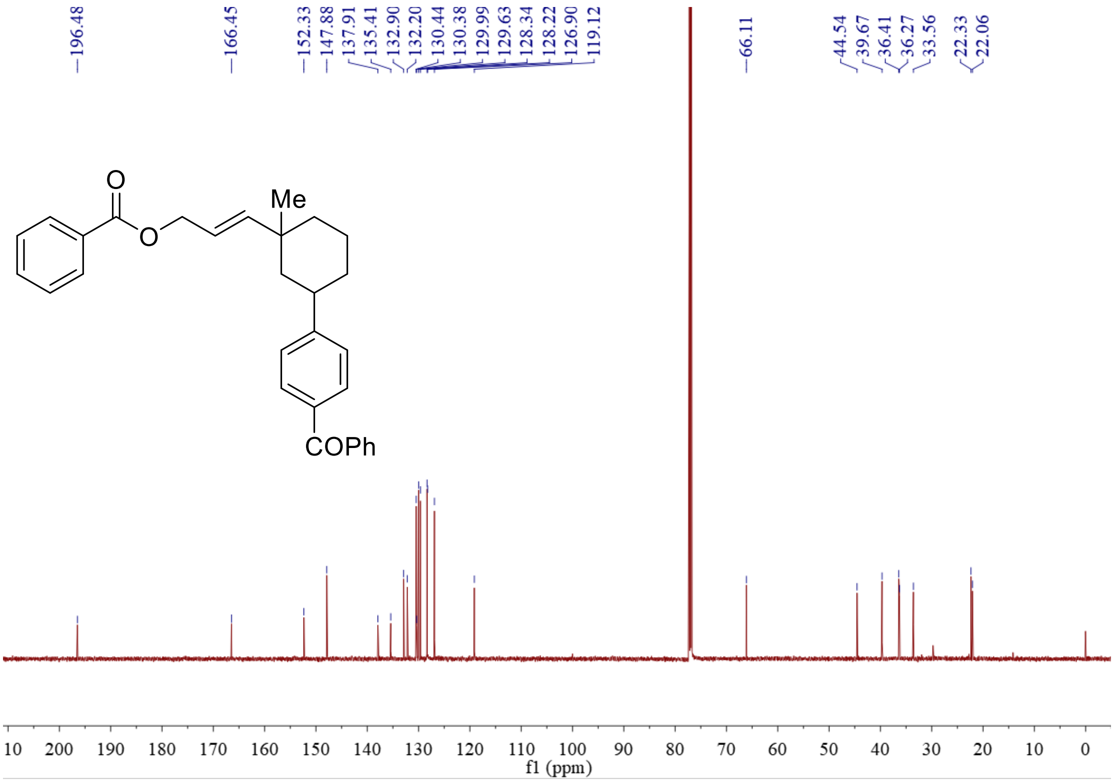


## (*E*)-3-(3-(4-formylphenyl)-1-methylcyclohexyl)allyl benzoate(33)

$^{1}$H NMR (600 MHz, $CDCl_3$)

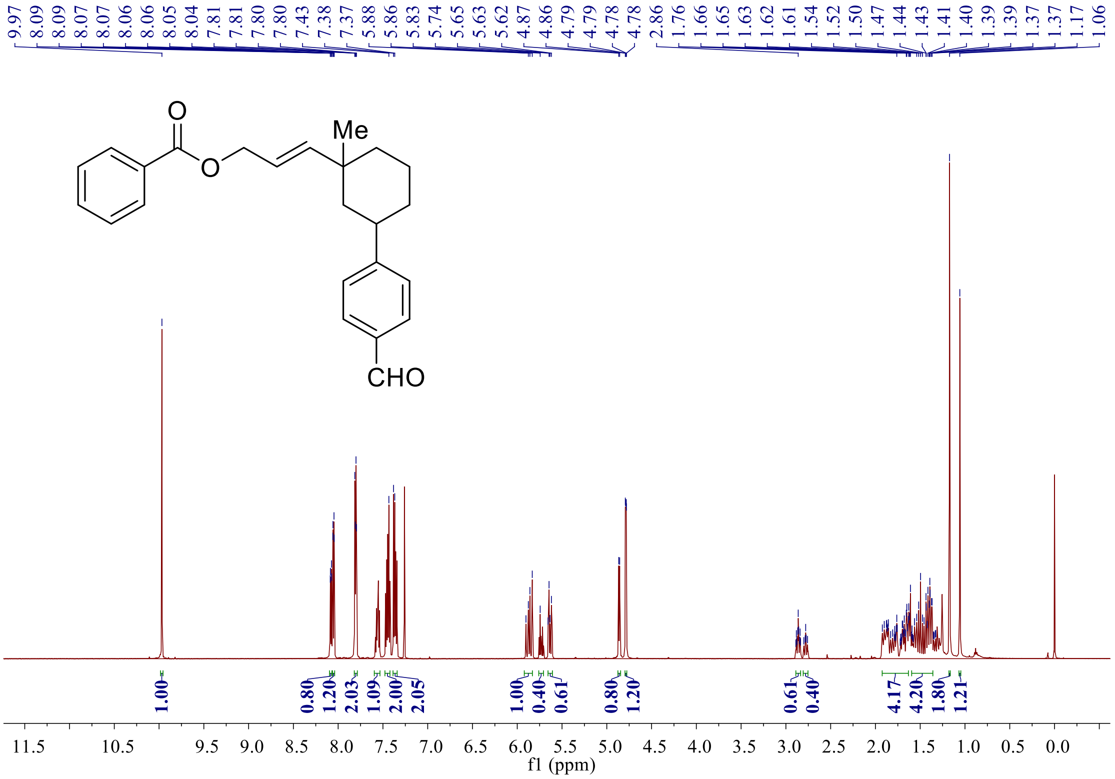

$^{13}C$ NMR (150 MHz, $CDCl_3$)

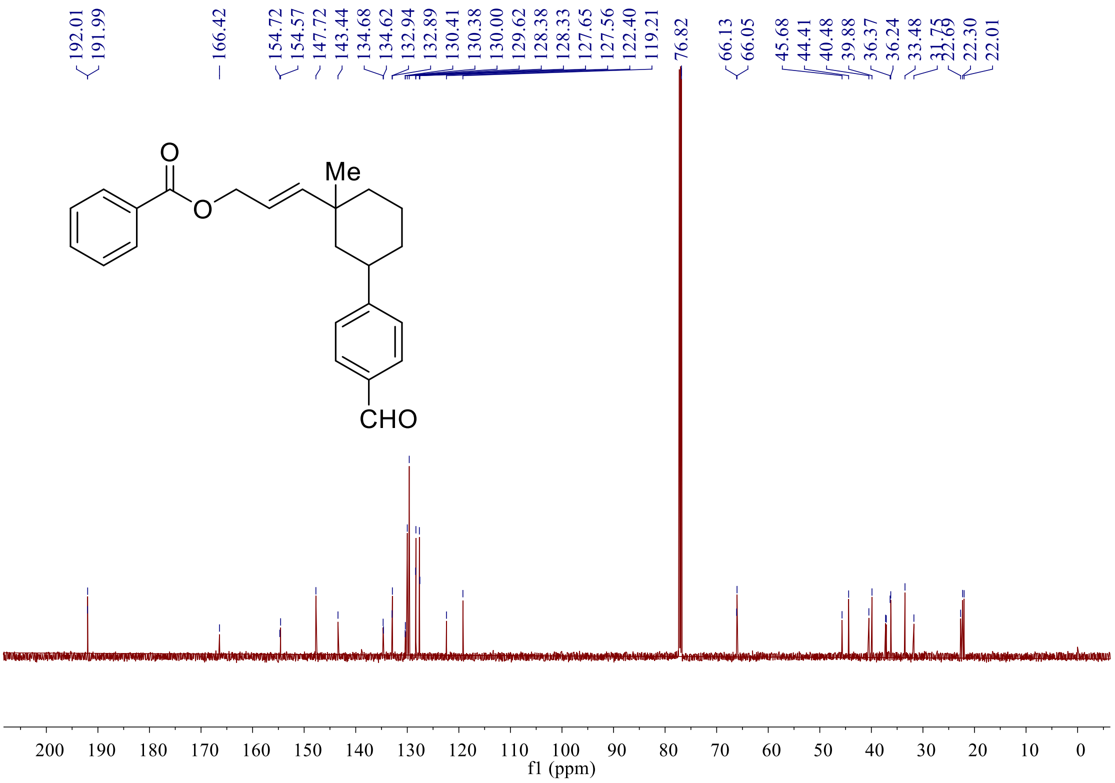


**(*E*)-3-(1-methyl-3-(4-(methylsulfonyl)phenyl)cyclohexyl)allyl benzoate (34)**

$^{1}H$ NMR (600 MHz, $CDCl_3$)

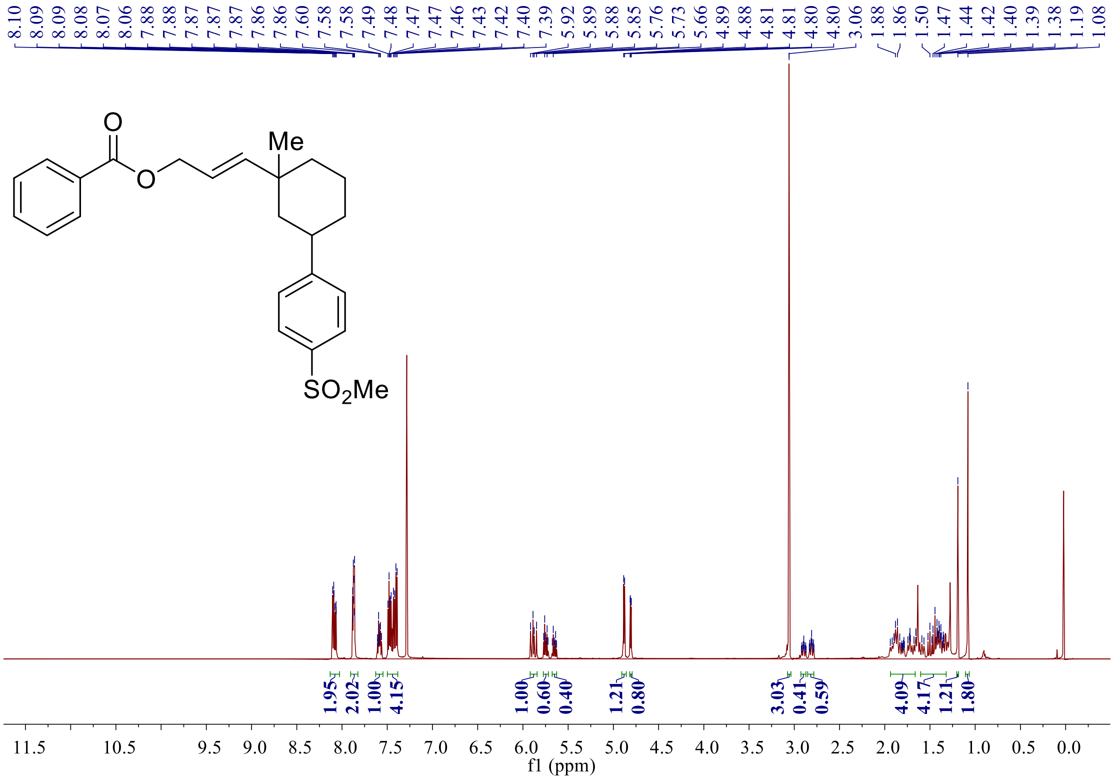

$^{13}C$ NMR (150 MHz, $CDCl_3$)

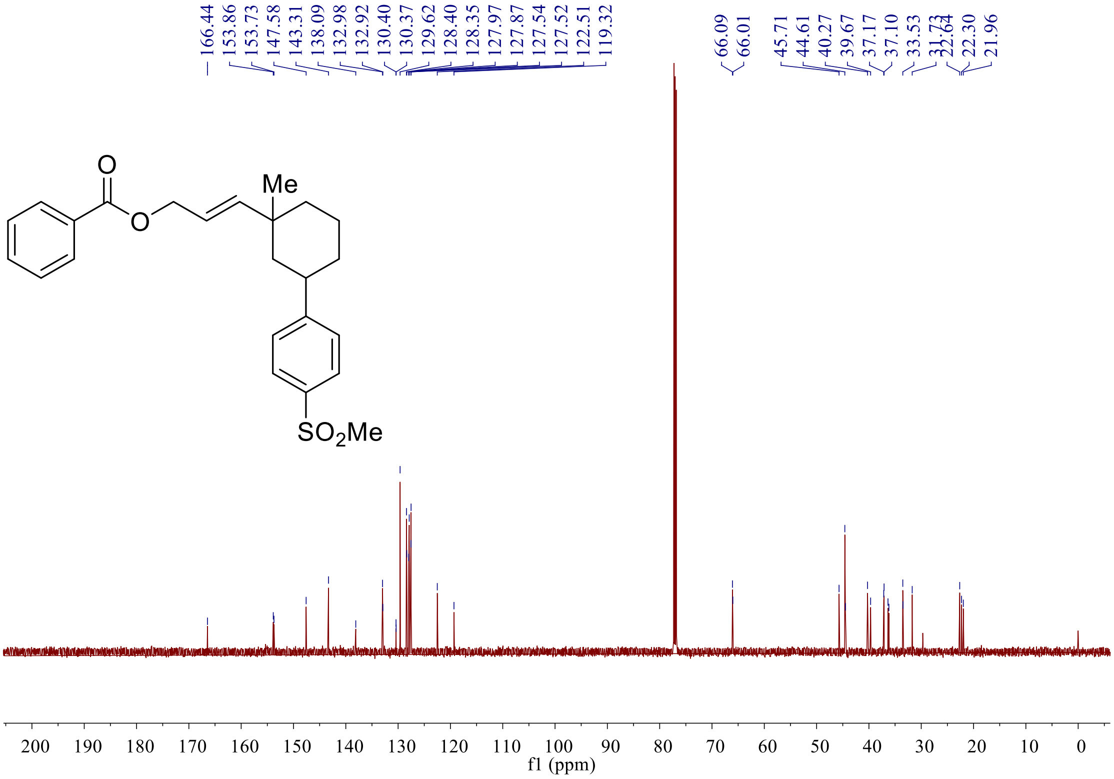


**(*E*)-3-(3-(3,5-bis(trifluoromethyl)phenyl)-1-methylcyclohexyl)allyl benzoate(35)**

$^{1}H$ NMR (600 MHz, $CDCl_3$)

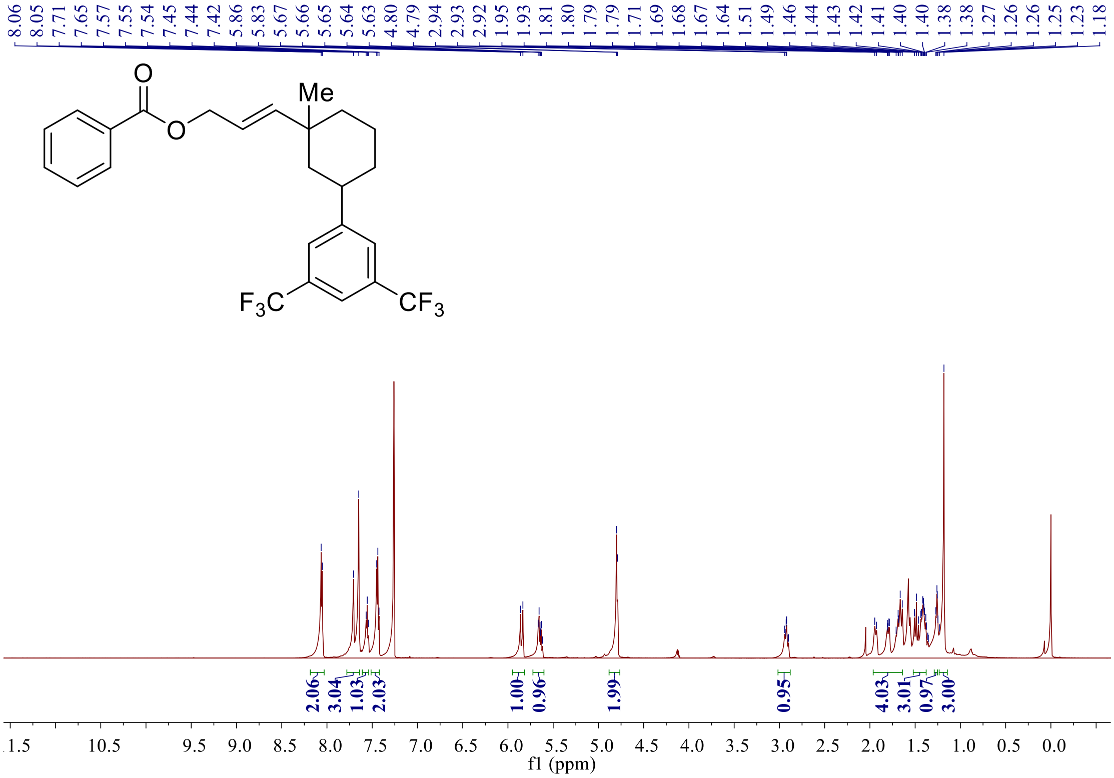

$^{19}$F NMR (377 MHz, $CDCl_3$)

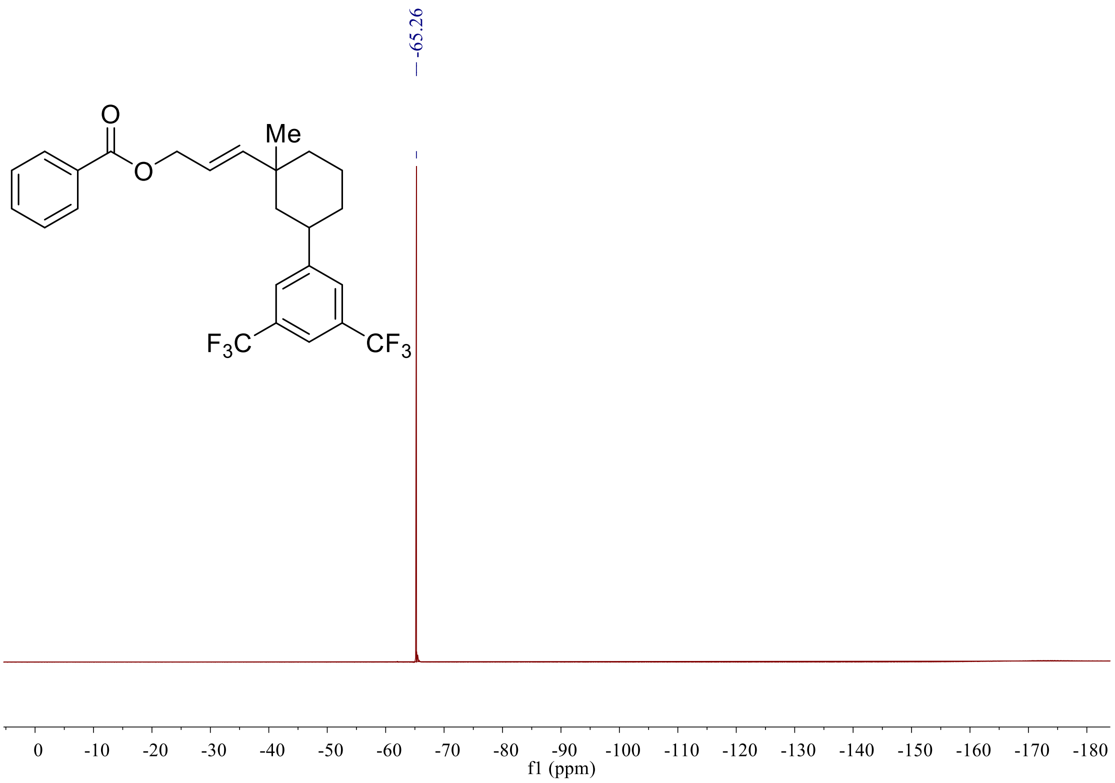


$^{13}$C NMR (150 MHz, $CDCl_3$)

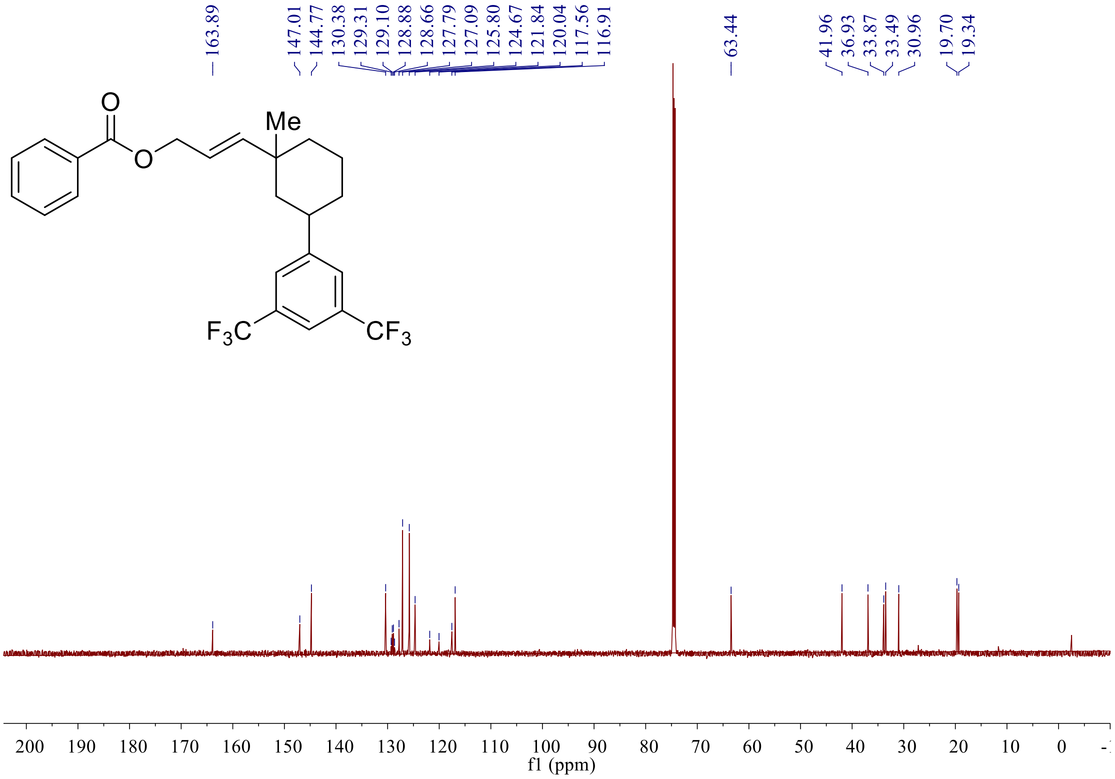

**(*E*)-3-(1-methyl-3-(pyridin-2-yl)cyclohexyl)allyl benzoate(36)**

$^1$H NMR (600 MHz, CDCl$_3$)

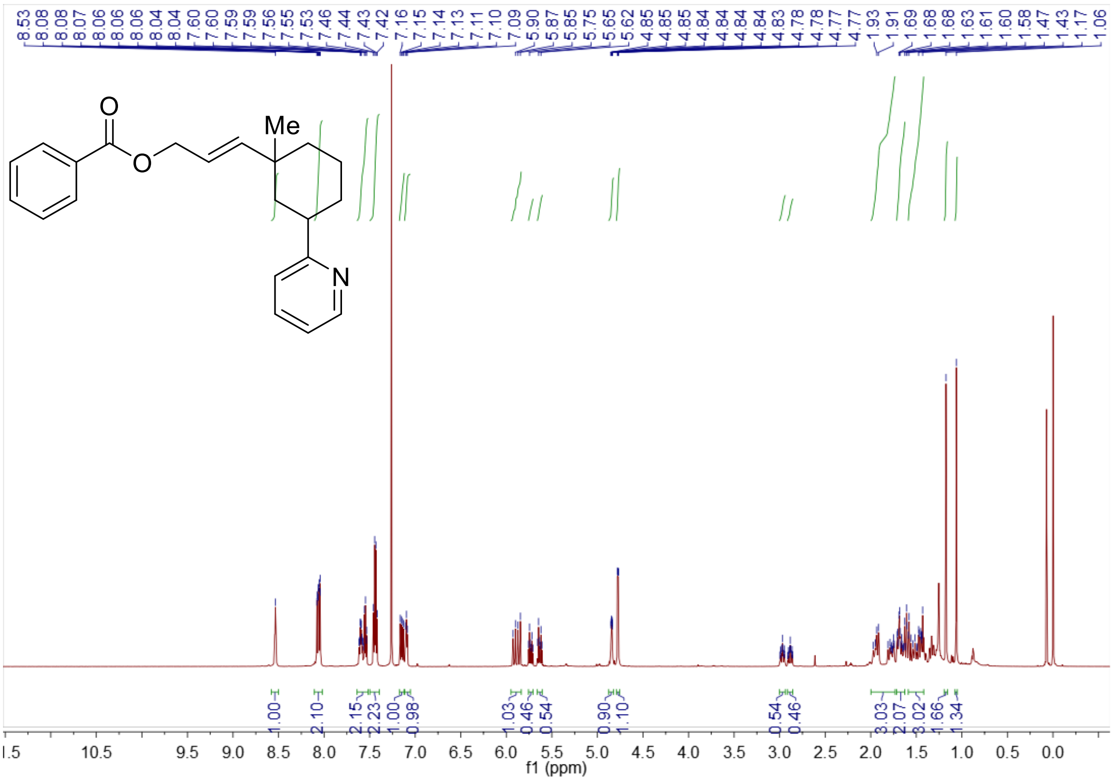

$^{13}$C NMR (150 MHz, CDCl$_3$)

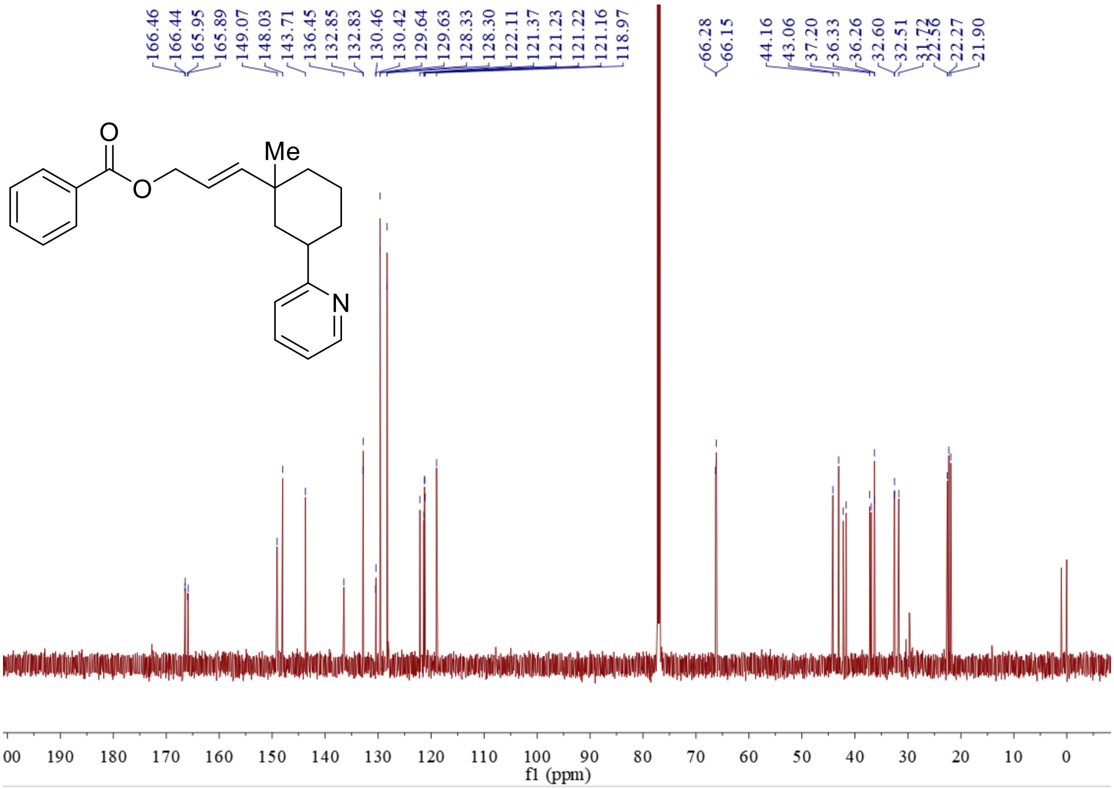

**(*E*)-3-(3-(6-methoxypyridin-2-yl)-1-methylcyclohexyl)allyl benzoate (37)**

$^1$H NMR (600 MHz, $CDCl_3$)

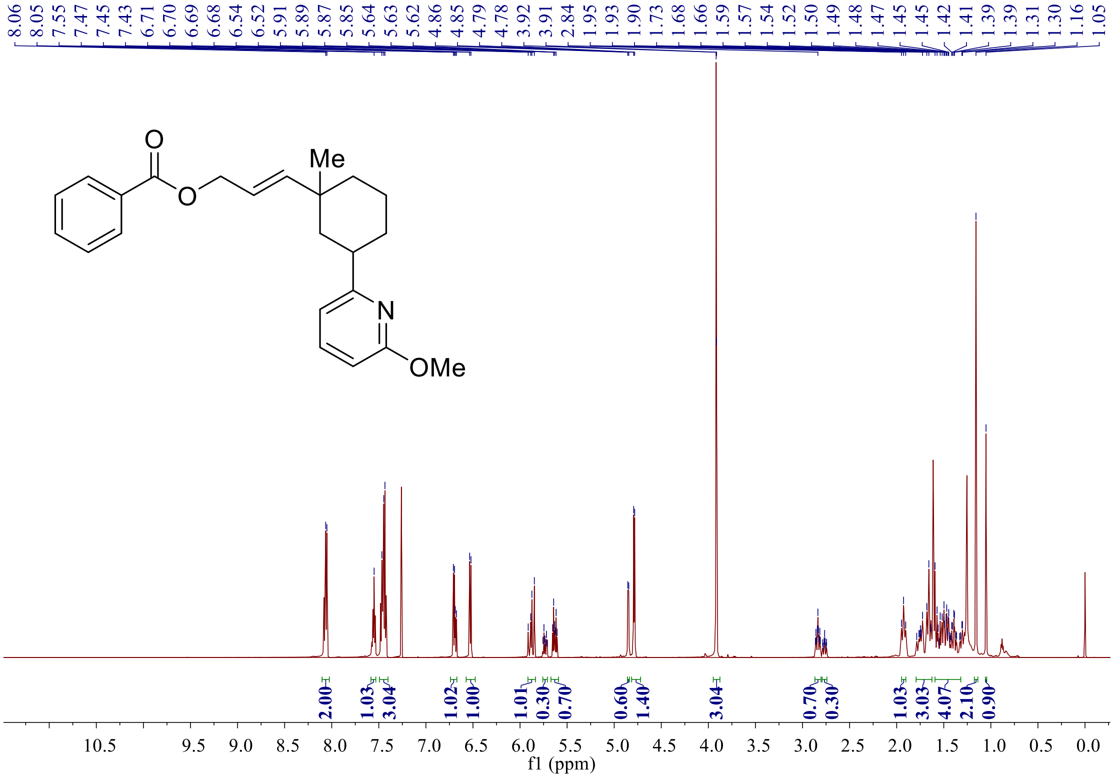


$^{13}$C NMR (150 MHz, $CDCl_3$)

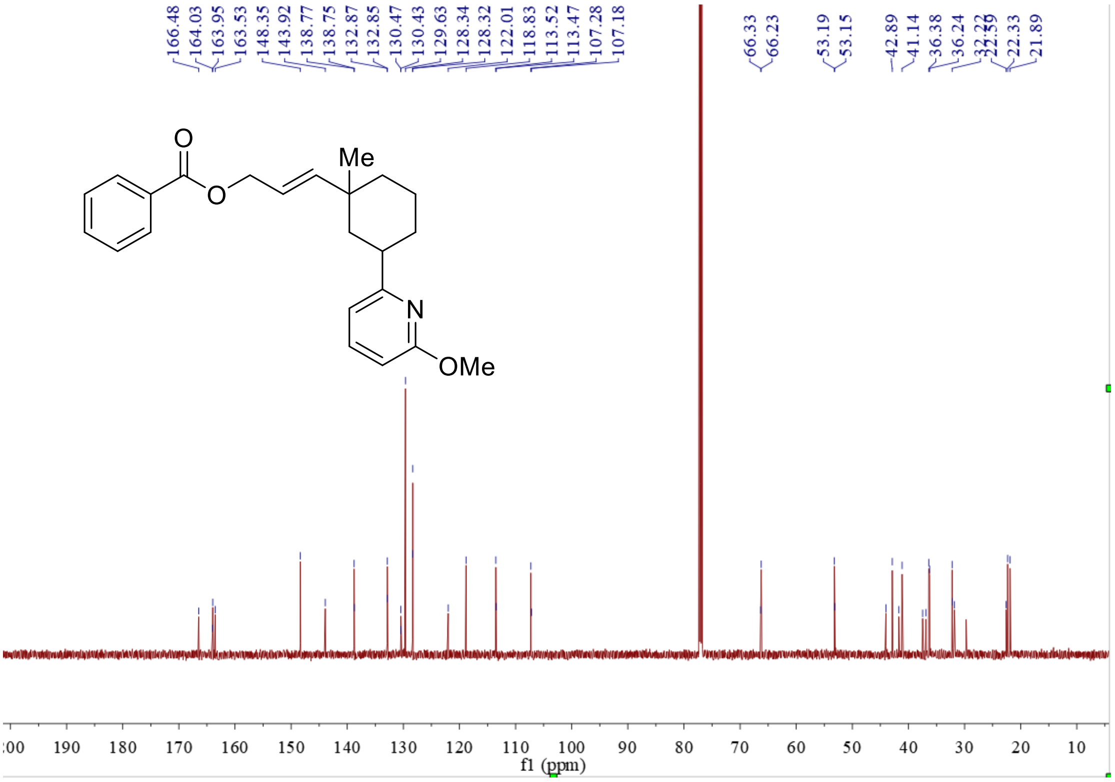

**(*E*)-3-(1-methyl-3-(5-(trifluoromethyl)pyridin-2-yl)cyclohexyl)allyl benzoate(38)**

H NMR (600 MHz, CDCl3

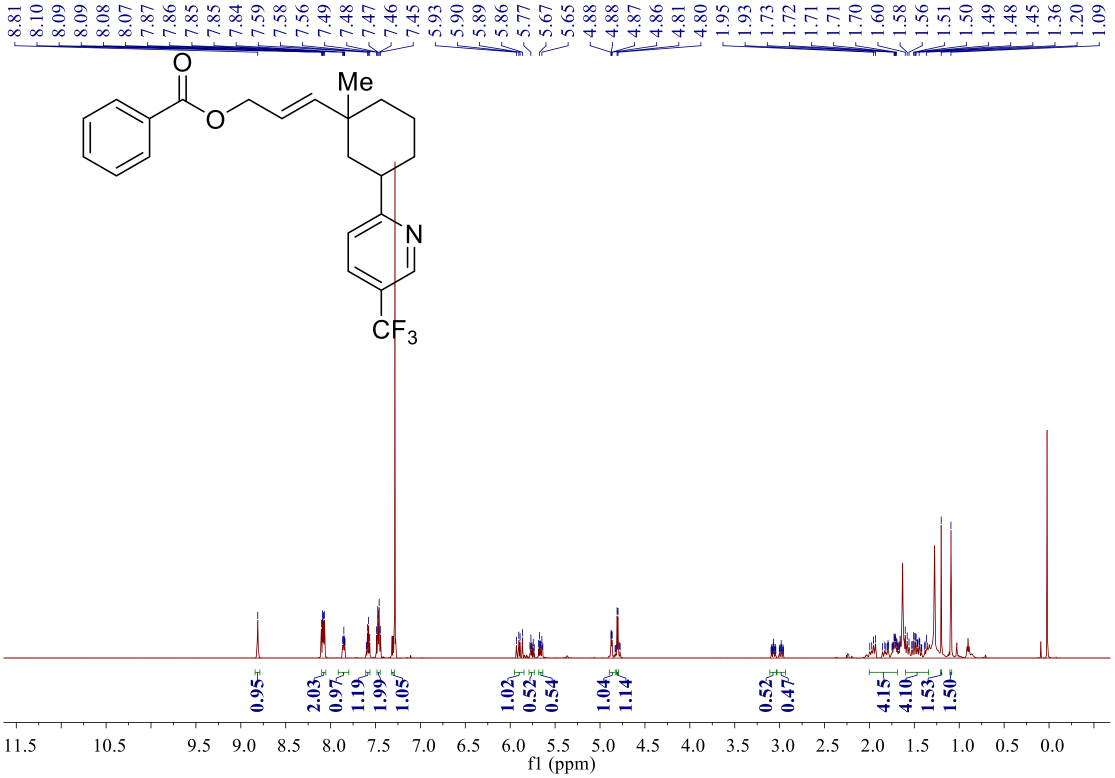

$^{13}$C NMR (150 MHz, CDCl3)

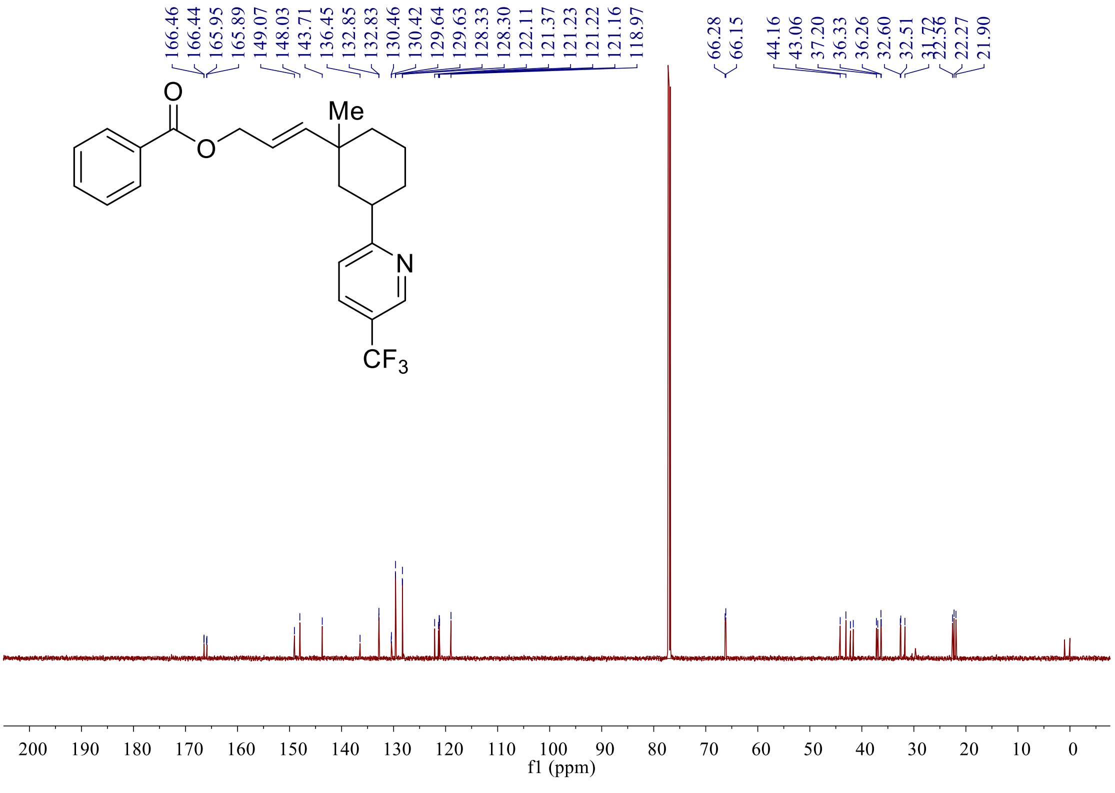

**(*E*)-3-(1-methyl-3-(p-tolyl)cyclohexyl)allyl benzoate(39)**

$^1$H NMR (600 MHz, $CDCl_3$)

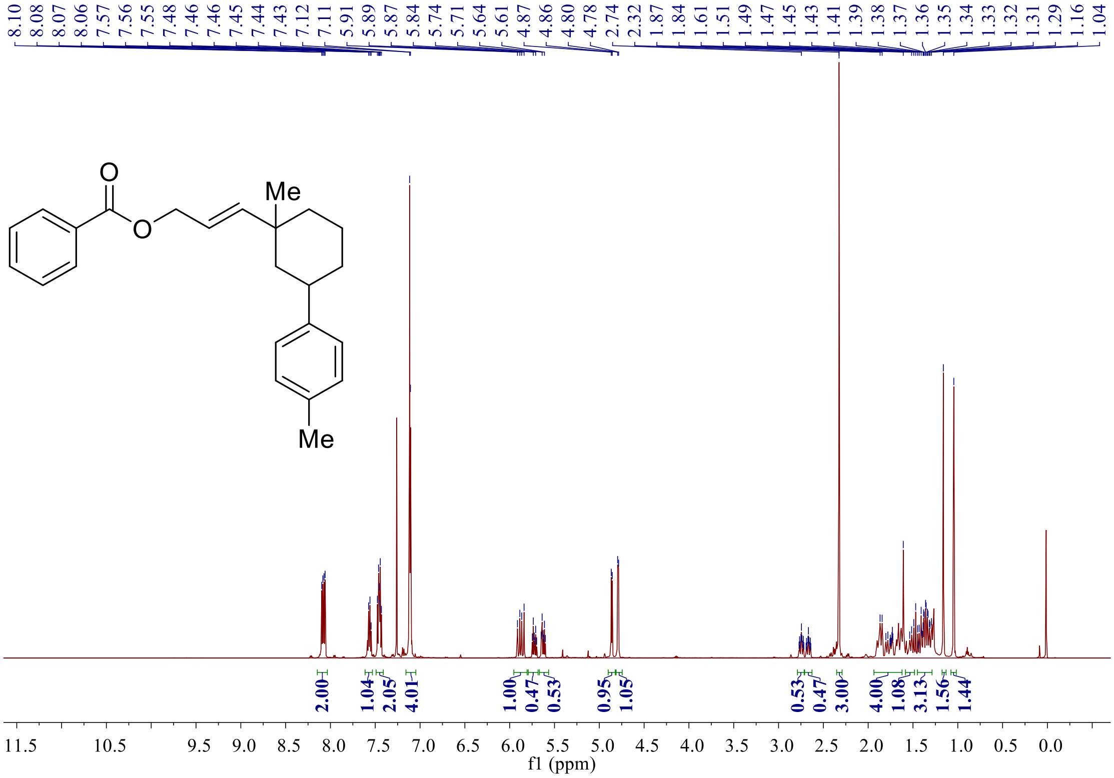

$^{13}$C NMR (150 MHz, $CDCl_3$)

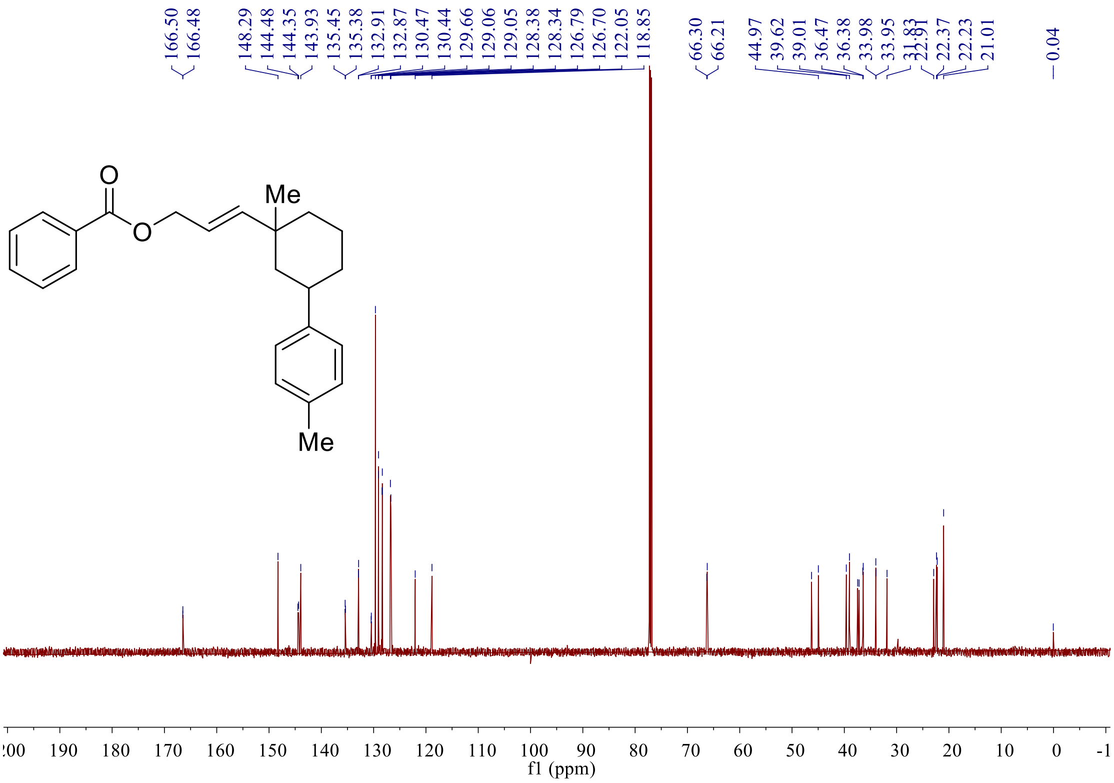

**(*E*)-3-(3-(4-methoxyphenyl)-1-methylcyclohexyl)allyl benzoate(40)**

$^1$H NMR (600 MHz, $CDCl_3$)

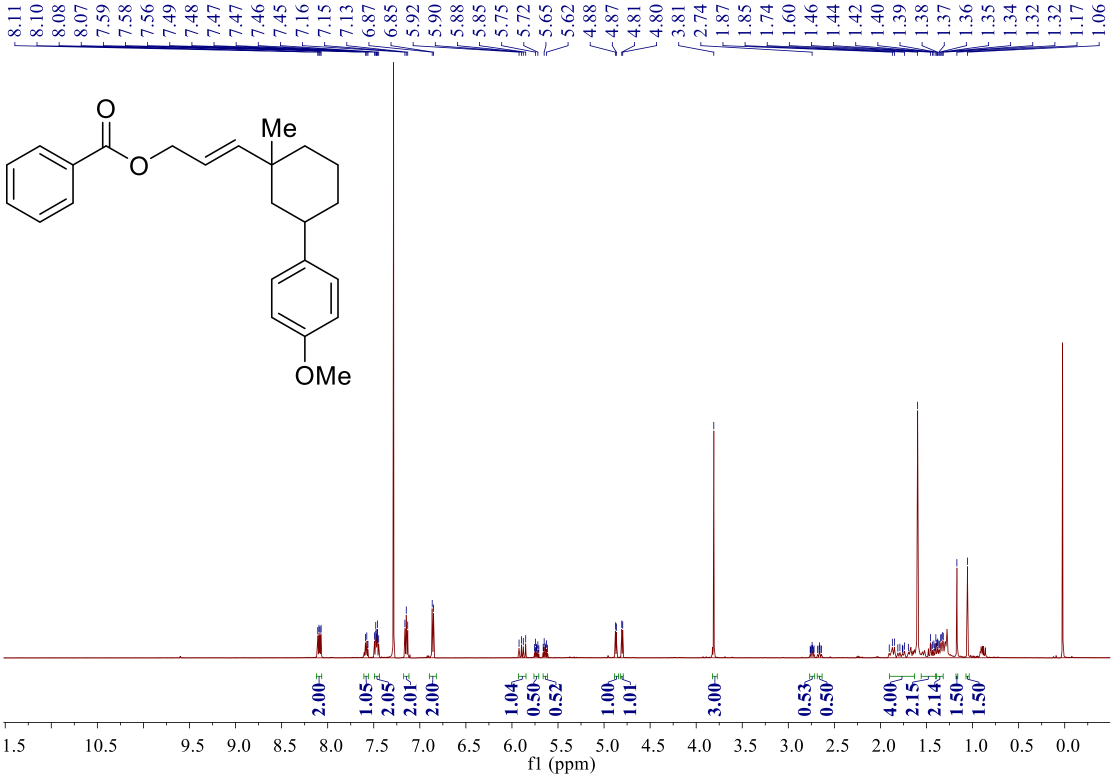


$^{13}$C NMR (150 MHz, $CDCl_3$)

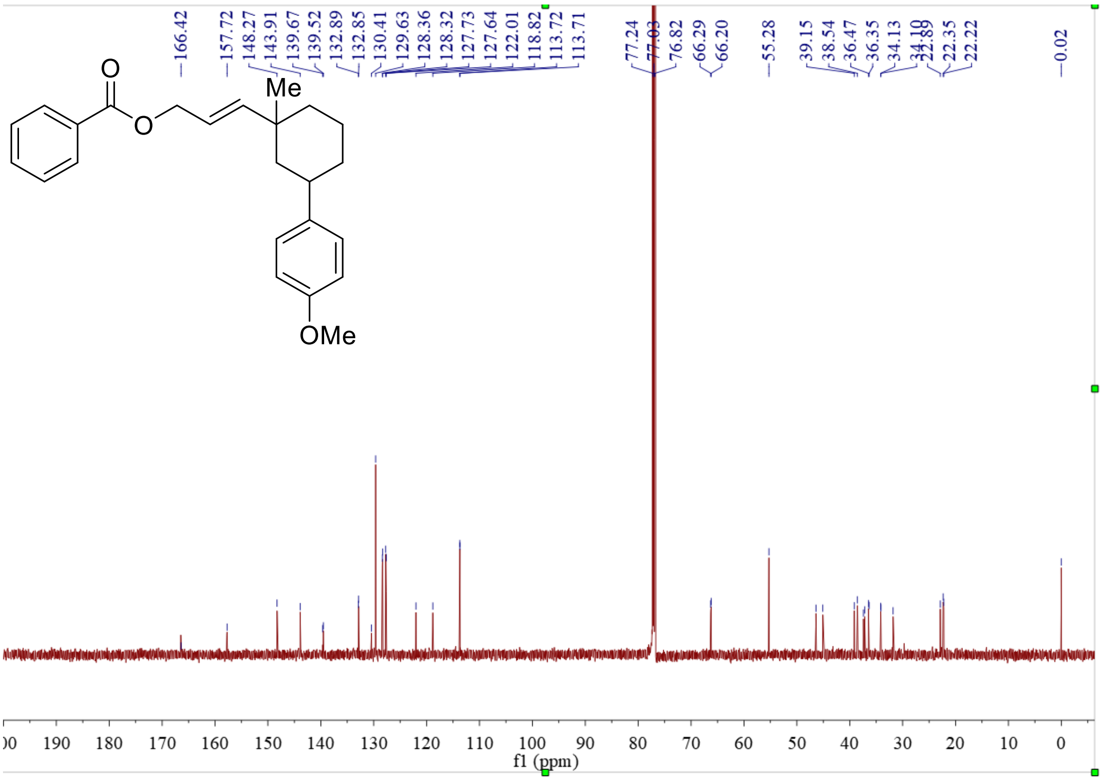

**(*E*)-3-(1-methyl-3-(pyridin-3-yl)cyclohexyl)allyl benzoate (41)**

$^{1}H$ NMR (600 MHz, $CDCl_3$)

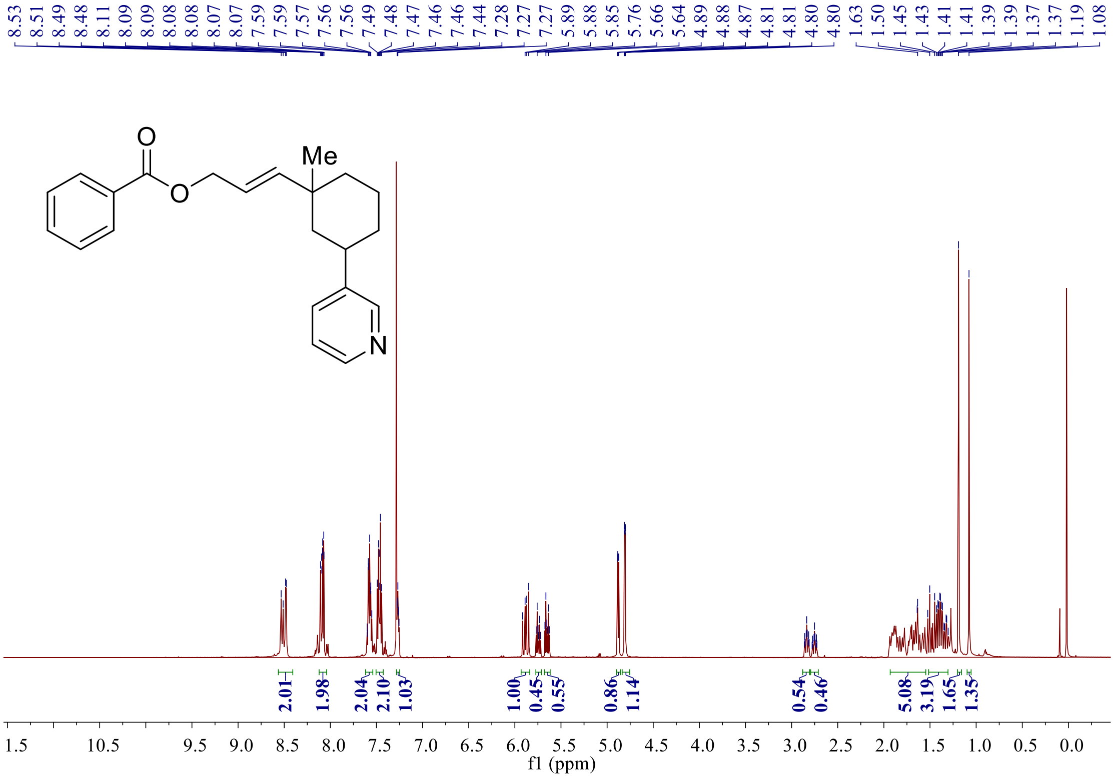

$^{13}C$ NMR (150 MHz, $CDCl_3$)

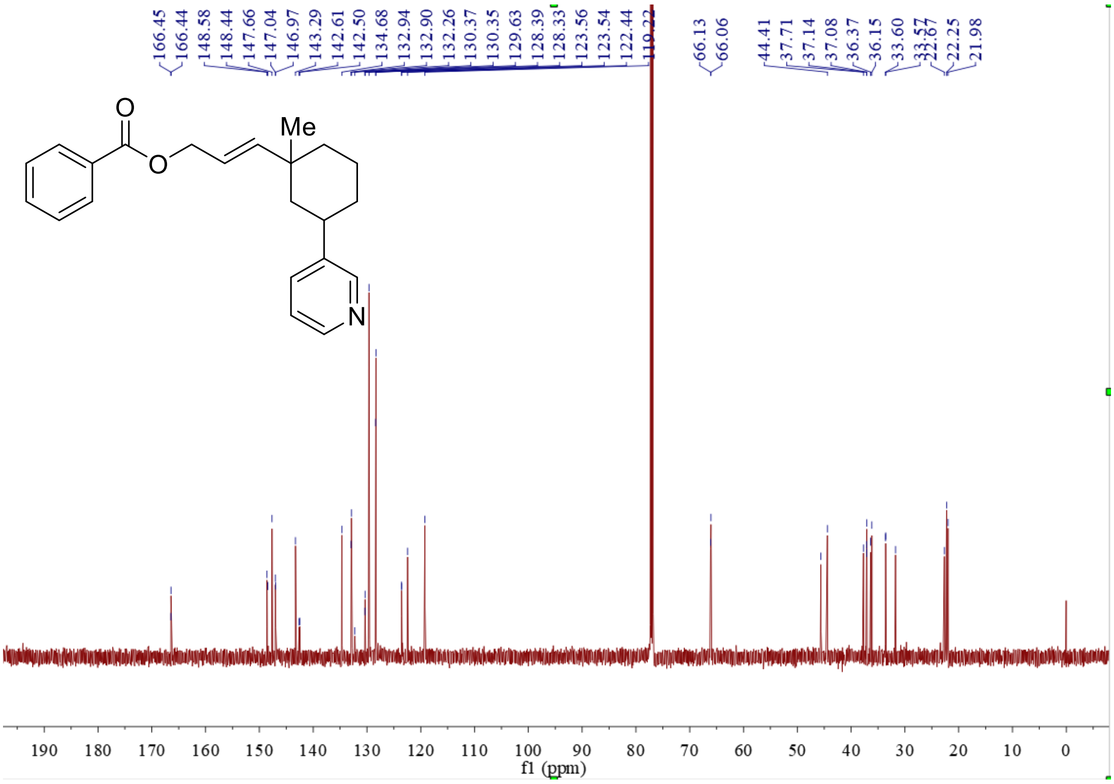

**(*E*)-3-(3-(benzo[d]thiazol-2-yl)-1-methylcyclohexyl)allyl benzoate(42)**

$^{1}$H NMR (600 MHz, $CDCl_3$)

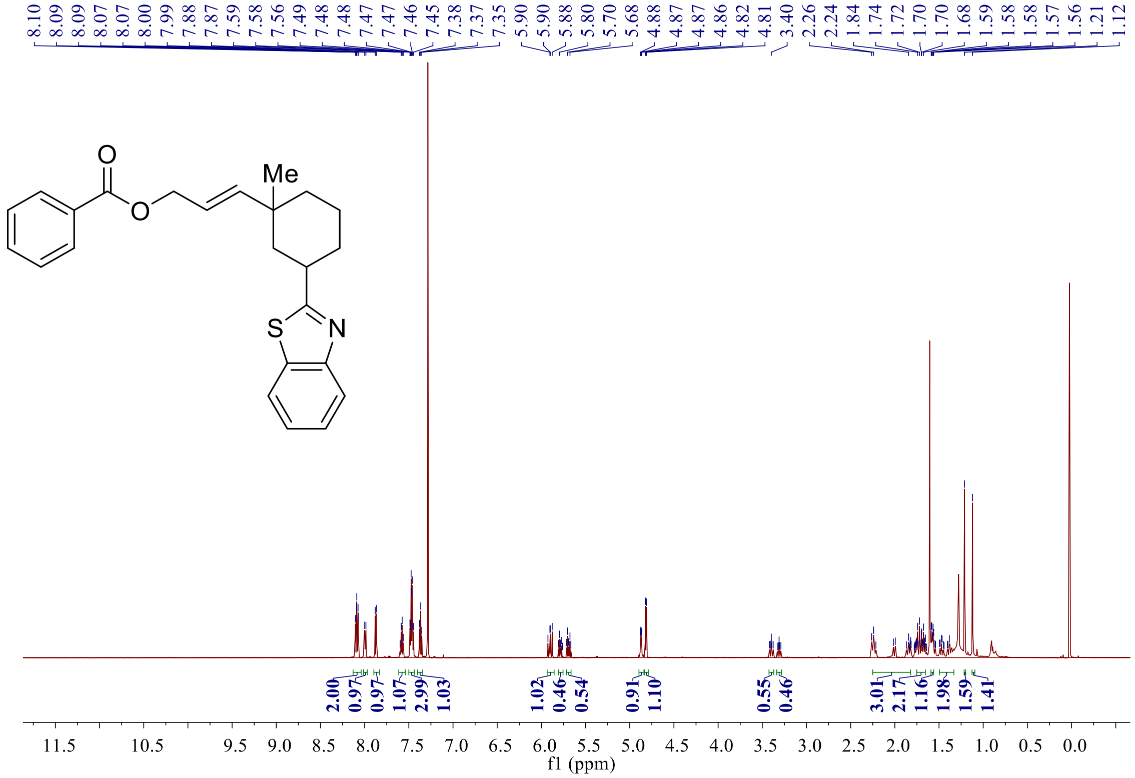


$^{13}$C NMR (150 MHz, $CDCl_3$)

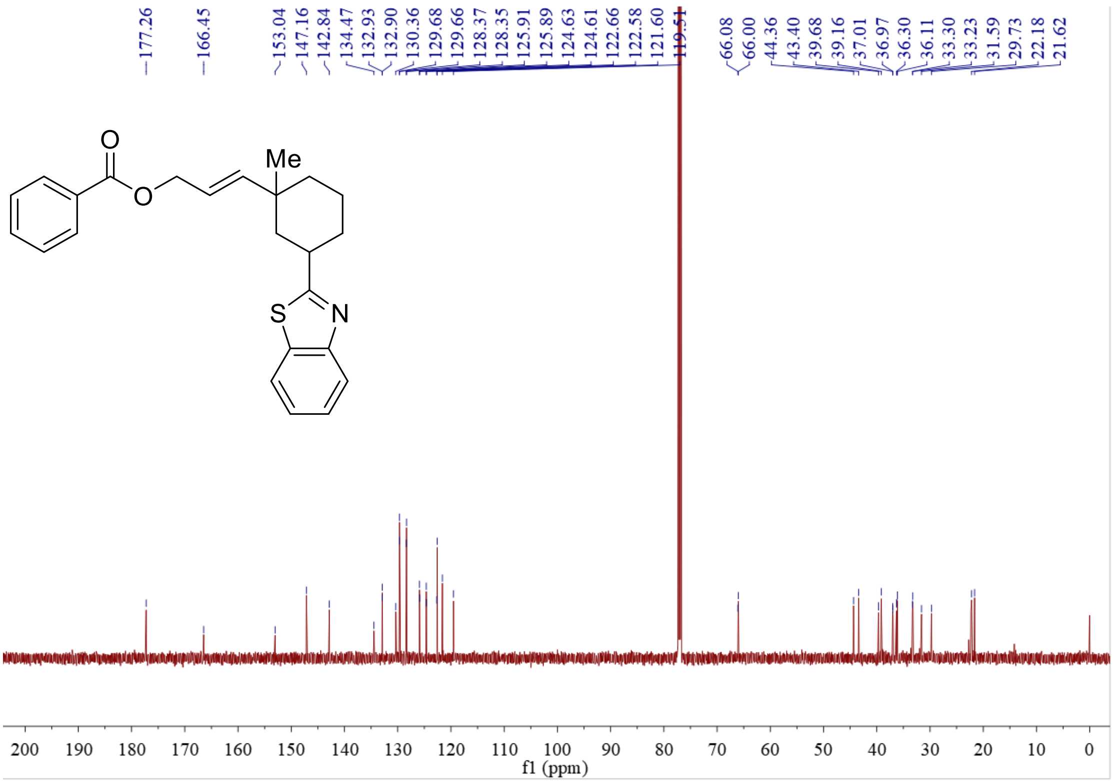

**3-(1-methyl-3-(pyridin-3-yl)cyclohexyl)propyl benzoate compound with methane (1:1) (43)**

$^{1}H$ NMR (600 MHz, $CDCl_3$)

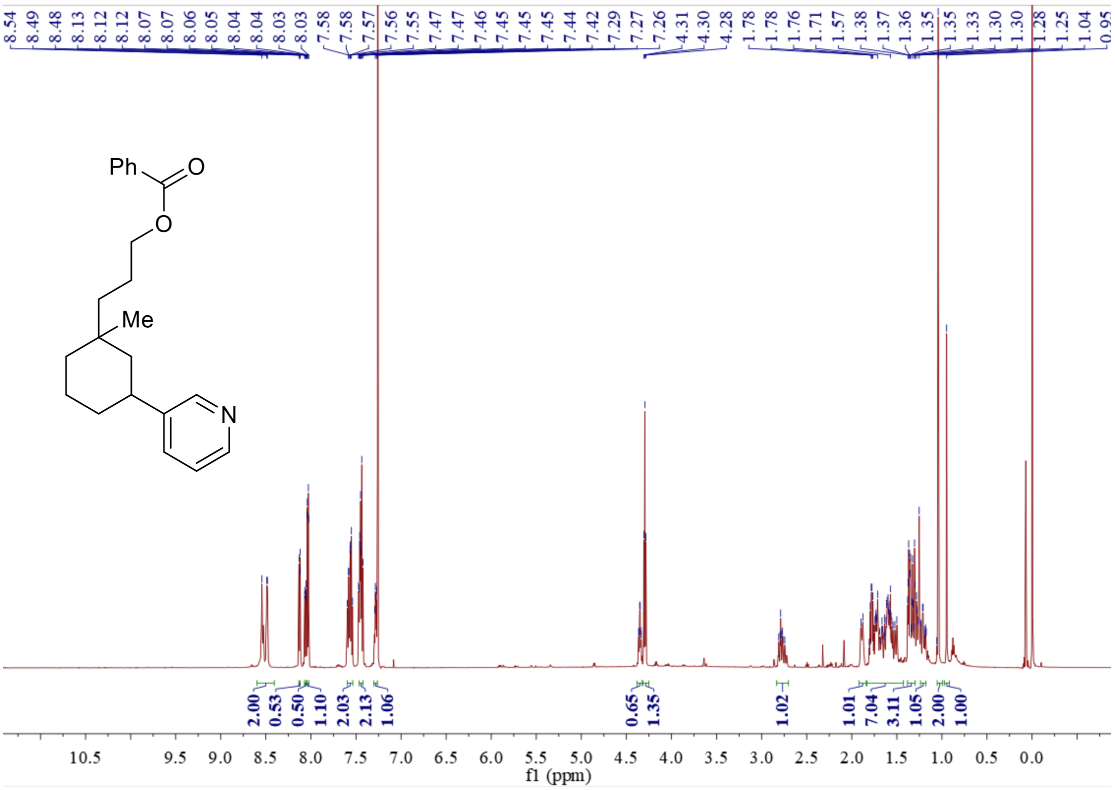


$^{13}C$ NMR (150 MHz, $CDCl_3$)

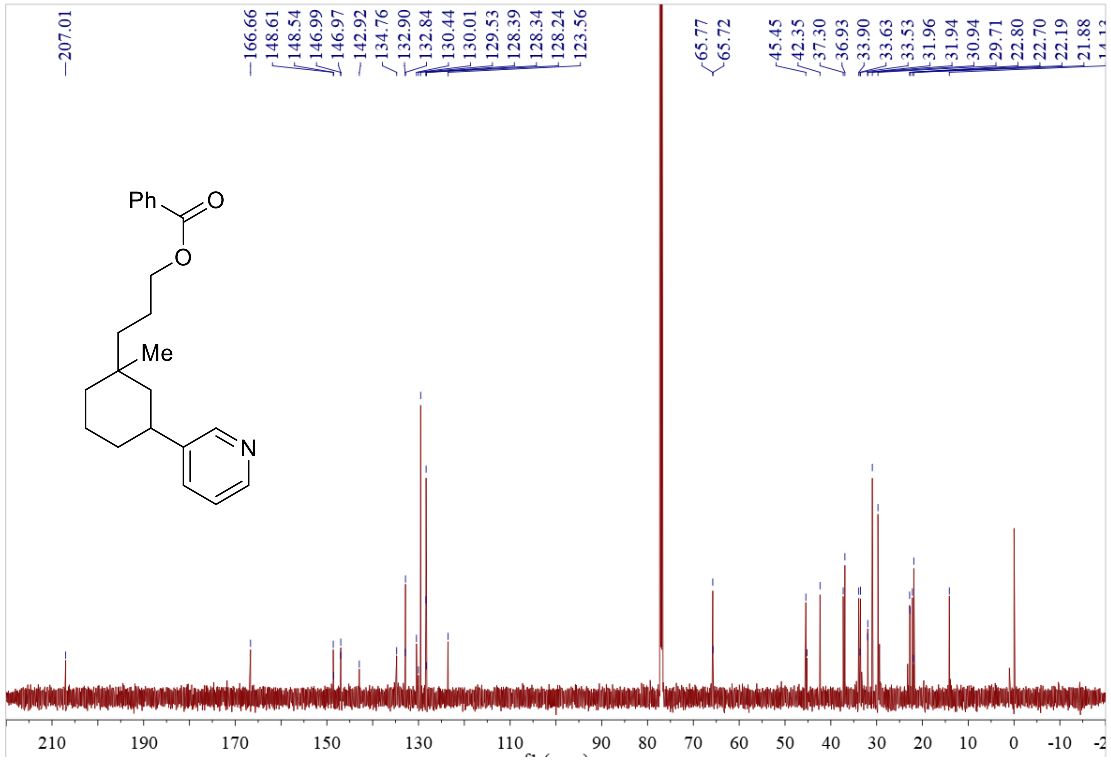

**(3-(3-(3-acetylphenyl)-1-methylcyclohexyl)oxiran-2-yl)methyl benzoate compound with methane (1:1) (44)**

$^{1}H$ NMR (600 MHz, $CDCl_3$)

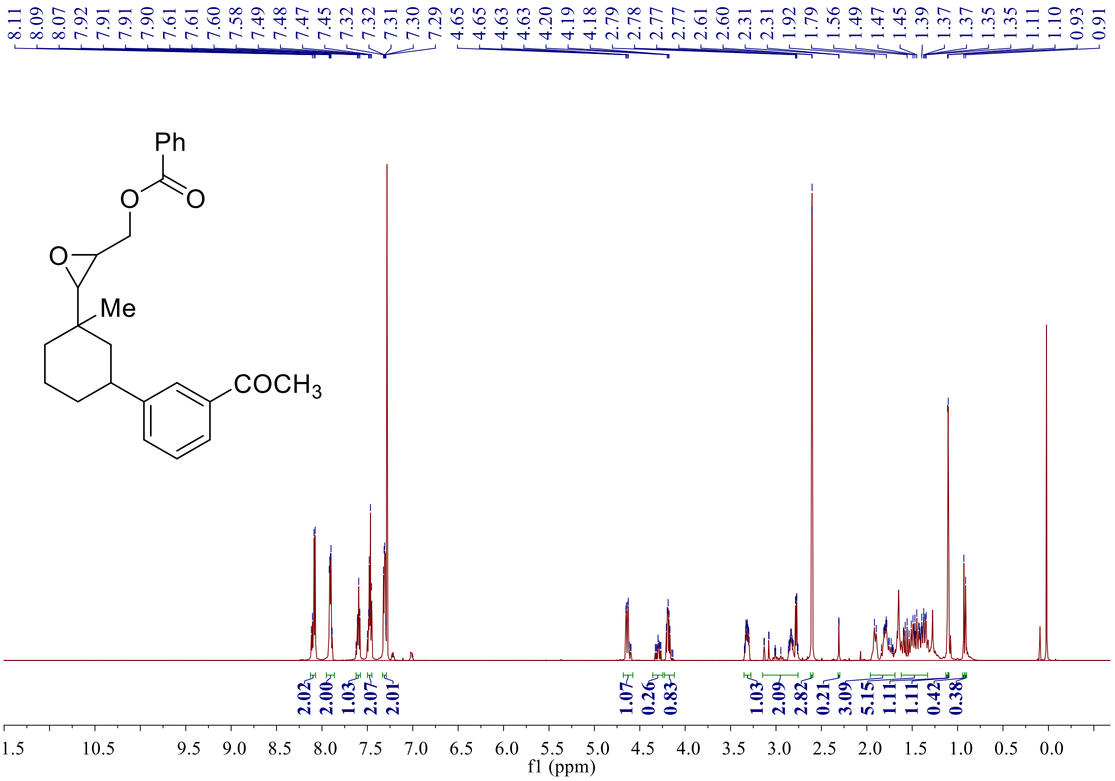


$^{13}C$ NMR (150 MHz, $CDCl_3$)

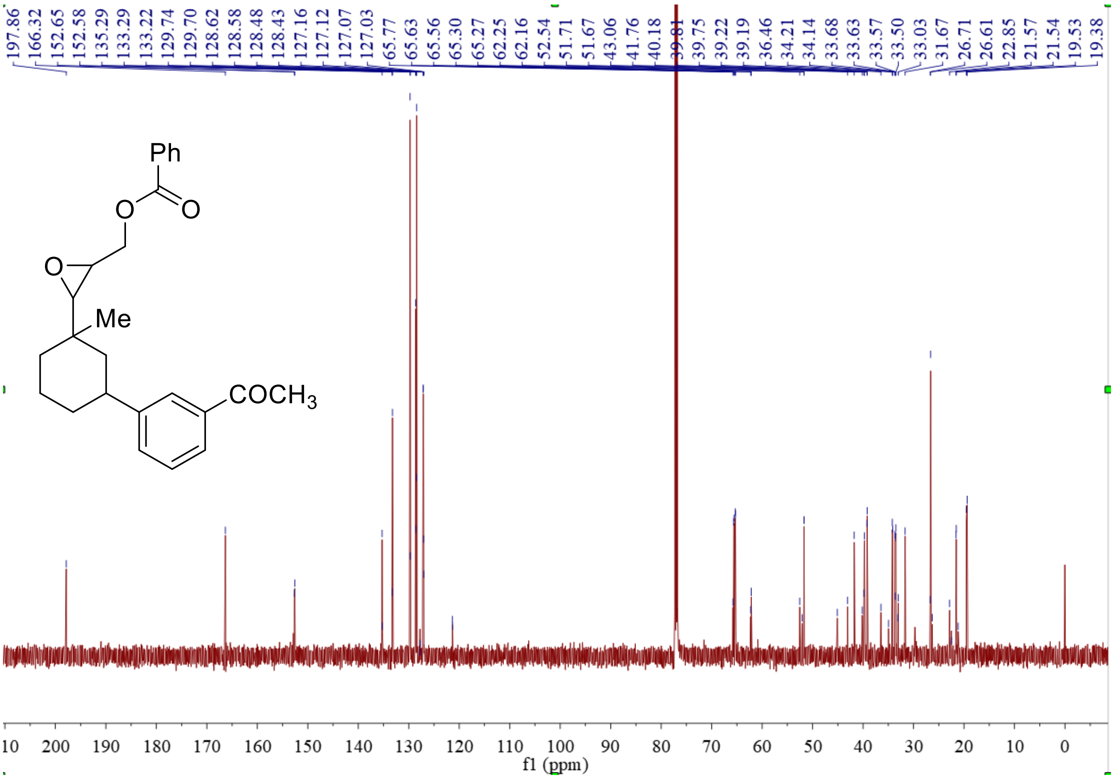

## 3-(4-acetylphenyl)-1-methylcyclohexane-1-carboxylic acid (46)

$^{1}$H NMR (600 MHz, $CDCl_3$)

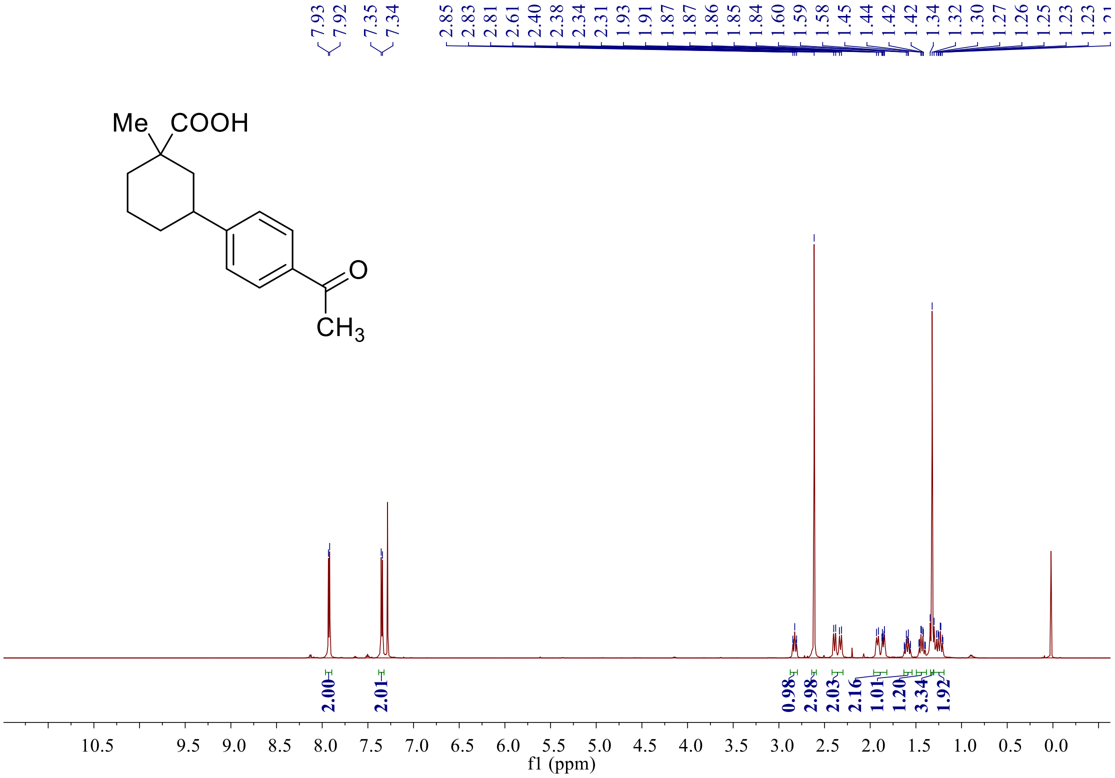


$^{13}$C NMR (150 MHz, $CDCl_3$)

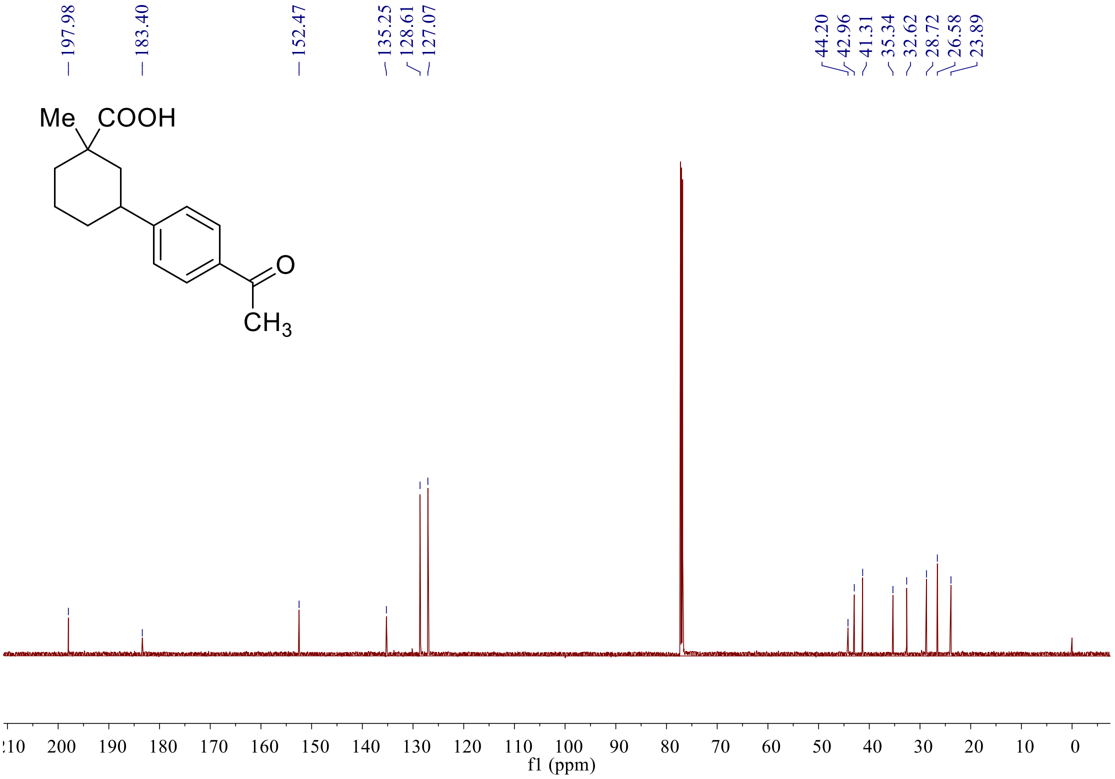

**1-(4-(3-(hydroxymethyl)-3-methylcyclohexyl)phenyl)ethan-1-ol (47)**

$^{1}$H NMR (600 MHz, $CDCl_3$)

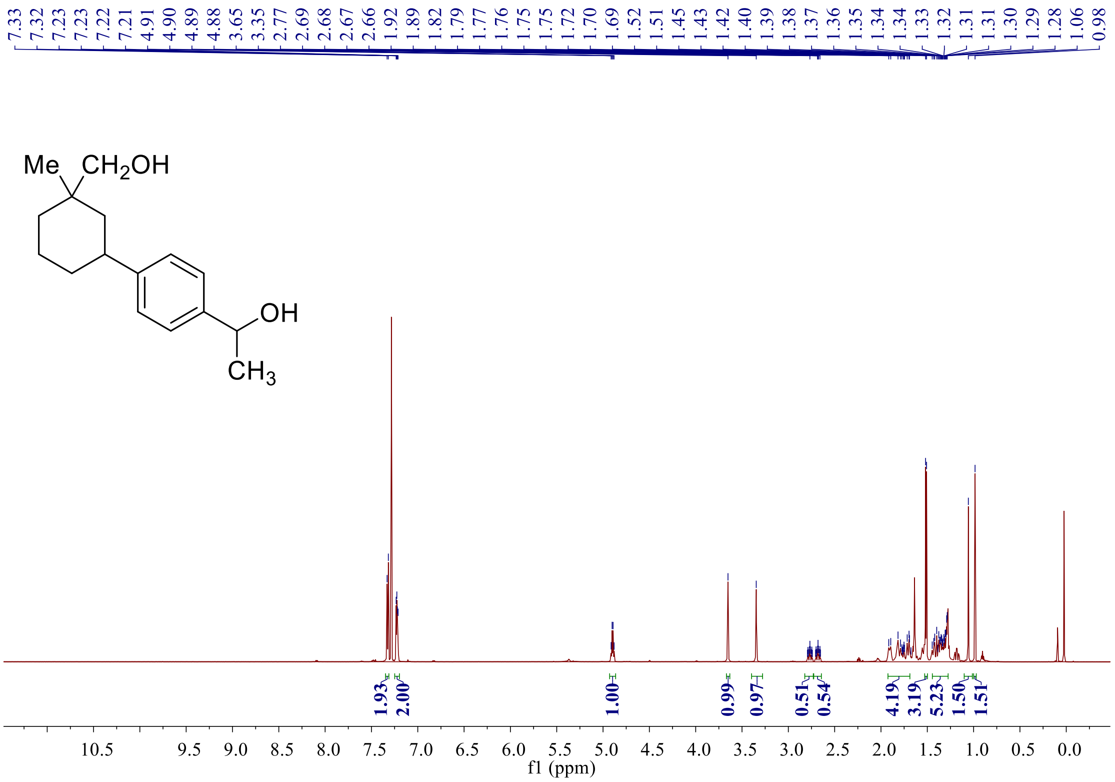

$^{13}$C NMR (150 MHz, $CDCl_3$)

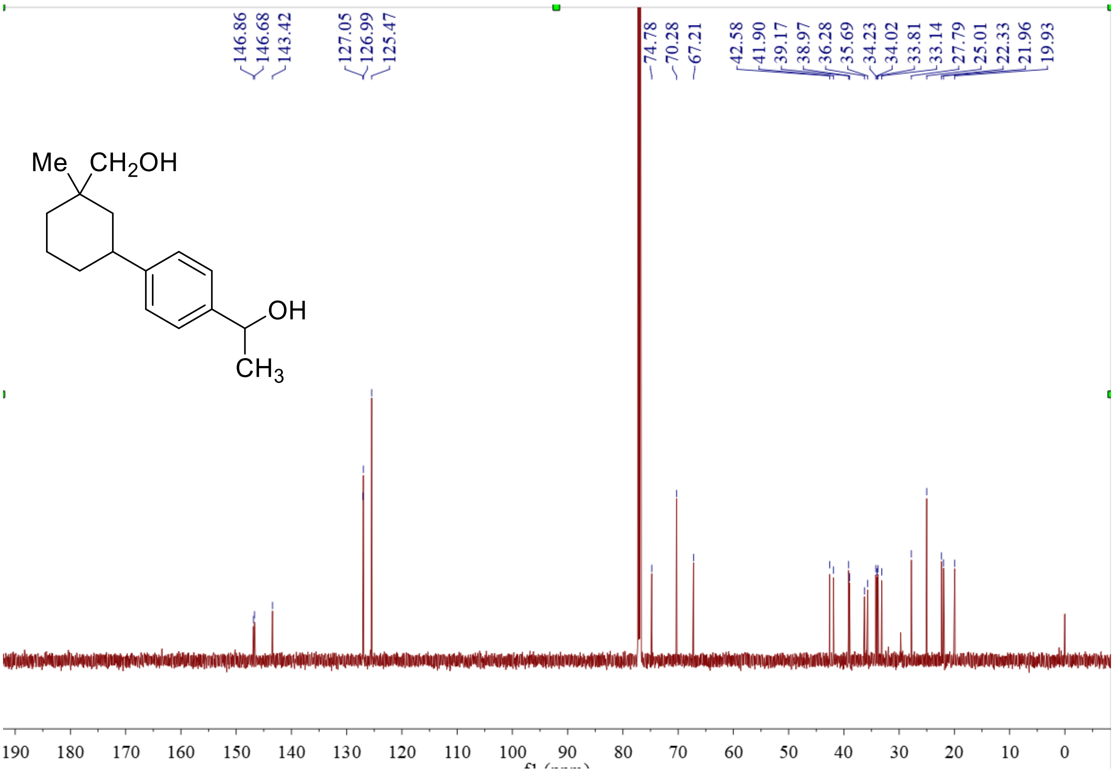

**4-(5-(1-methylcyclohexyl)penta-3,4-dien-1-yl)benzonitrile(48)**

$^{1}H$ NMR (600 MHz, $CDCl_3$)

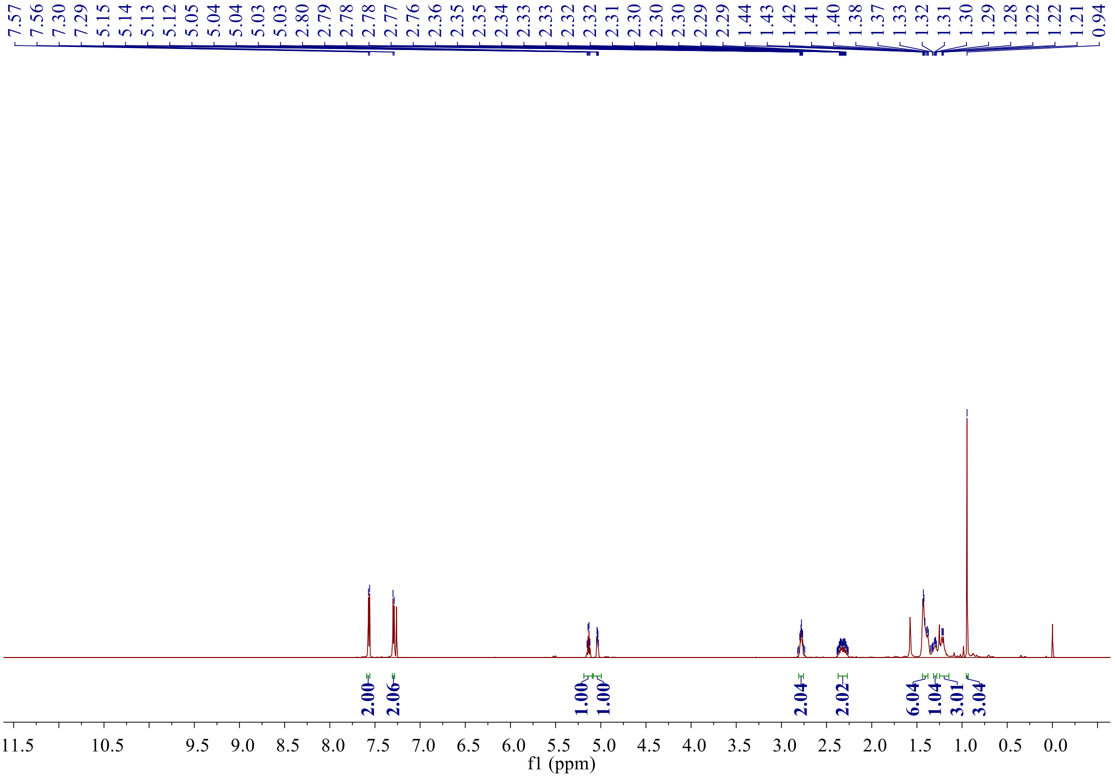


$^{13}C$ NMR (150 MHz, $CDCl_3$)

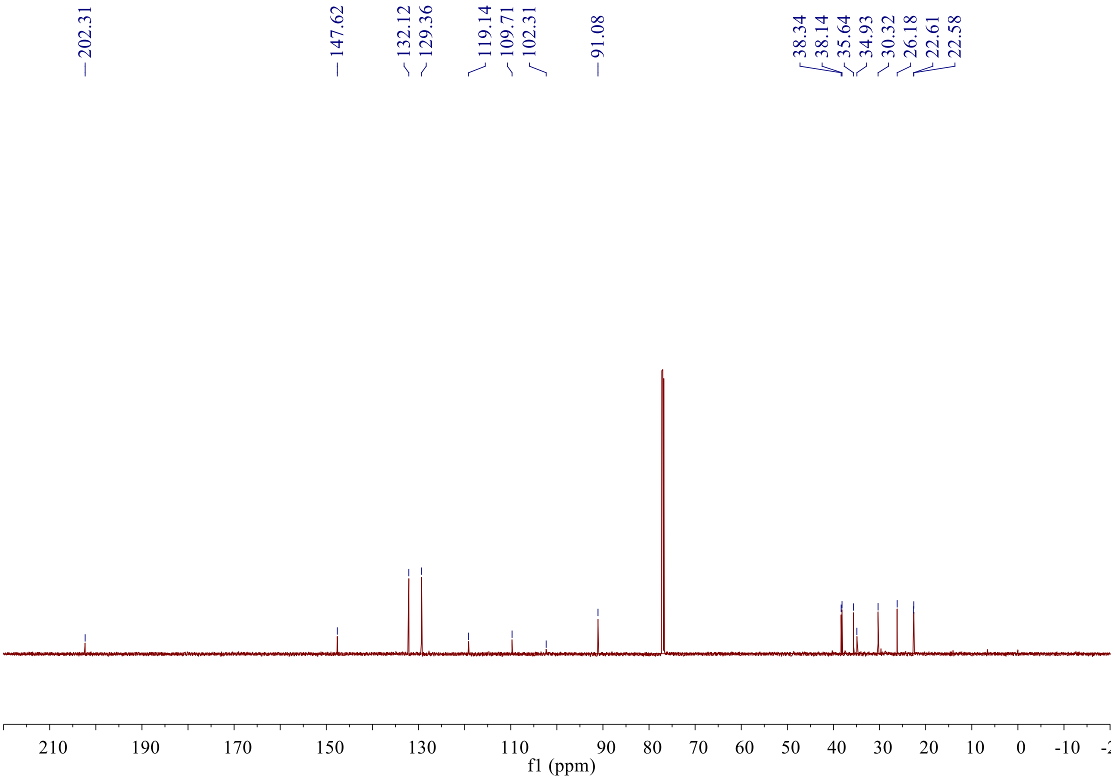

**4-(1-(4-(tert-butyl)phenyl)-2-(1-methylcyclohexyl)vinyl)benzonitrile(49)**

$^{1}$H NMR (600 MHz, CDCl$_3$)

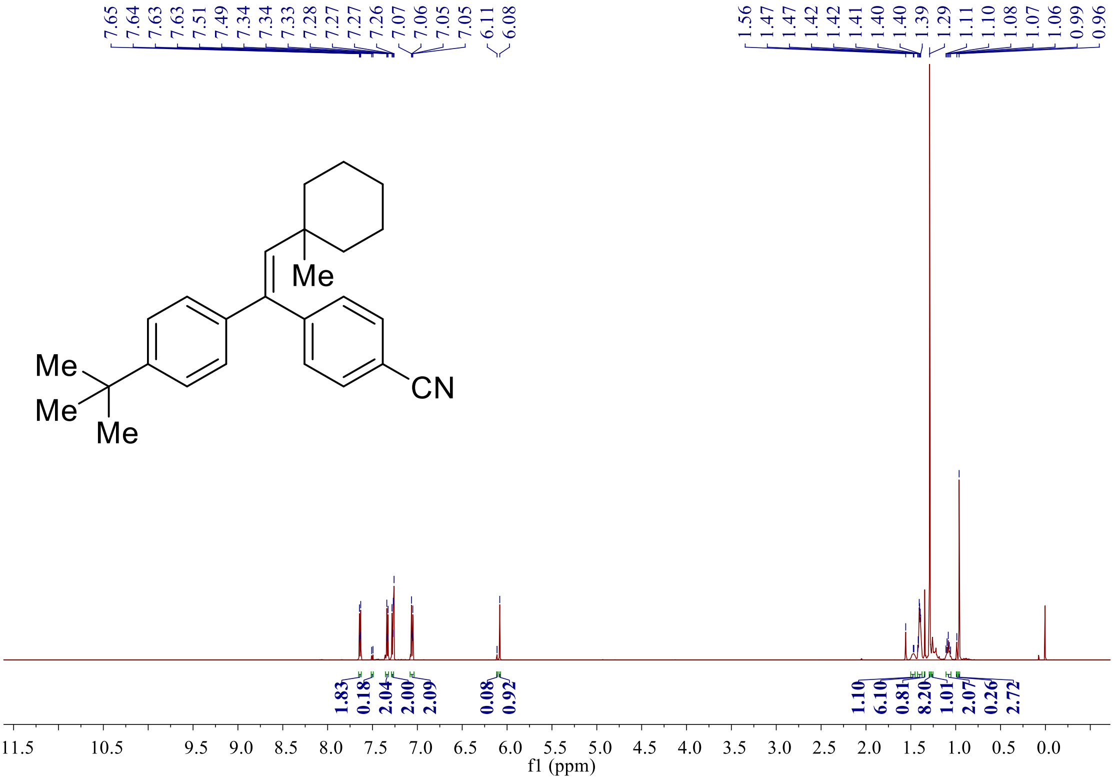


$^{13}$C NMR (150 MHz, CDCl$_3$)

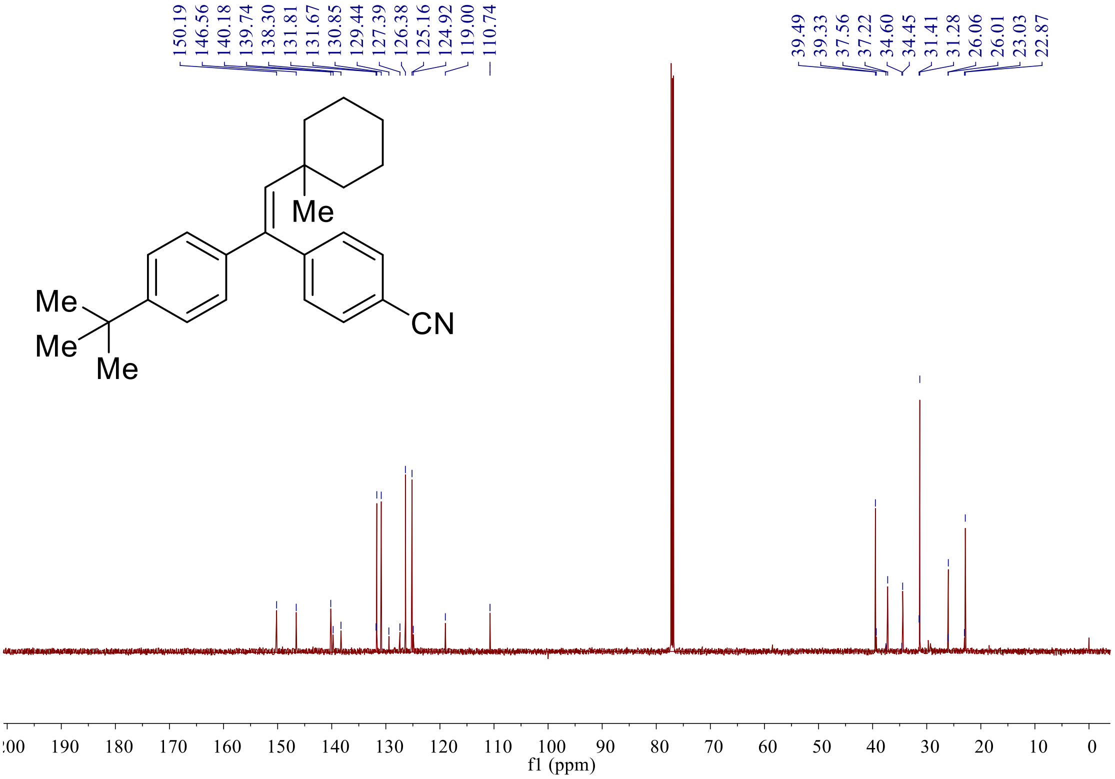

## Synthesis of (dtbpy)NiII(2-tolyl)I[S50]

$^1H$ NMR (400 MHz, Acetone)

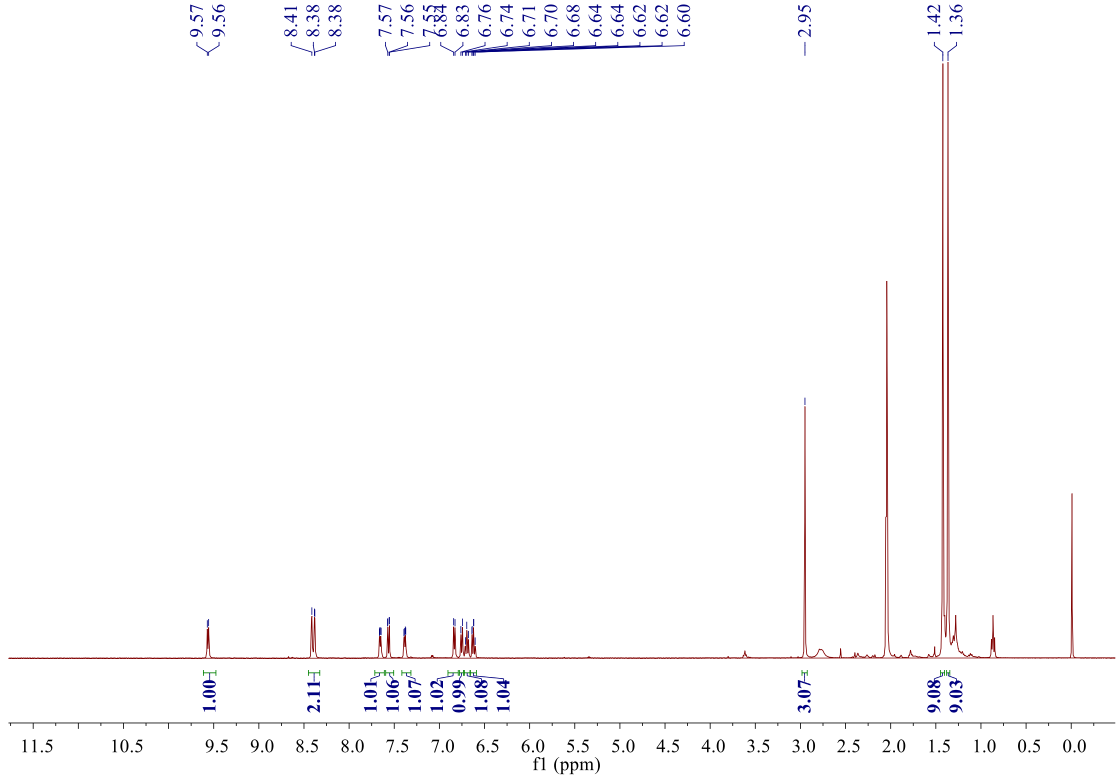


## (E)-3-(1-methylcyclohexyl)allyl isonicotinate (S23)

$^1H$ NMR (600 MHz, $CDCl_3$)

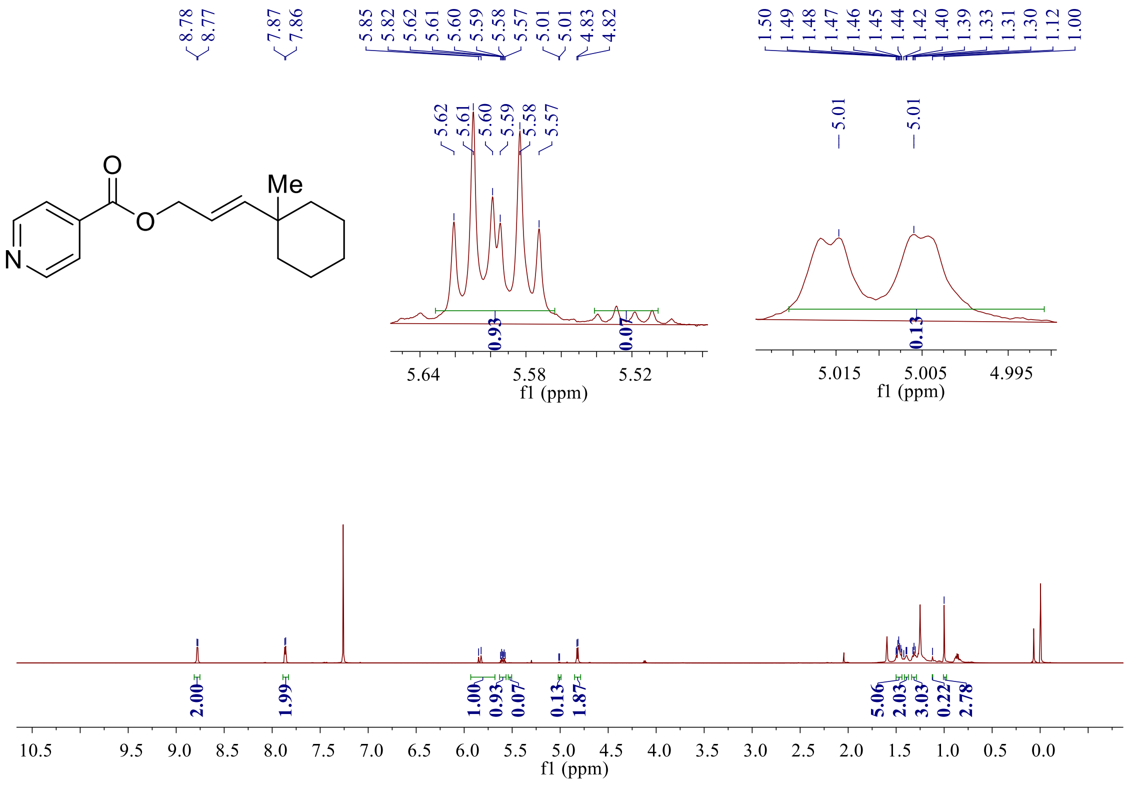

$^{13}$C NMR (150 MHz, $CDCl_3$)

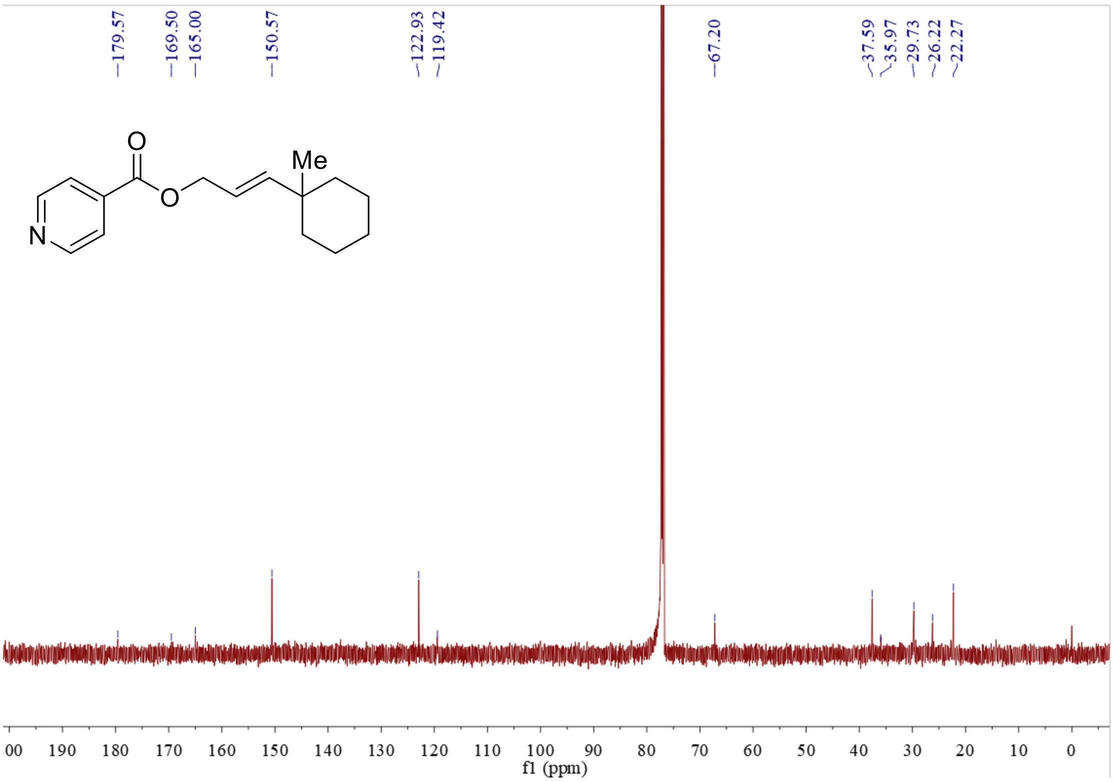

Supplement: SC-011-D0SC01471K-s001 [file SC-011-D0SC01471K-s001.pdf]
